# Radical-mediated direct C–H amination of arenes with secondary amines†
†Electronic supplementary information (ESI) available: Full experimental procedures, spectral characterisation and details of DFT calculations. See DOI: 10.1039/c8sc01747f


**DOI:** 10.1039/c8sc01747f

**Published:** 2018-07-11

**Authors:** Sebastian C. Cosgrove, John M. C. Plane, Stephen P. Marsden

**Affiliations:** a School of Chemistry , University of Leeds , Leeds LS2 9JT , UK . Email: s.p.marsden@leeds.ac.uk

## Abstract

Aryl dialkyl amines, valuable subunits of a wide range of effect chemicals, are accessed by intramolecular amination of aromatic C–H bonds employing UV photolysis of *N*-chloroamines. The reactions show good functional group tolerance and allow access to a range of fused and bridged polycyclic structures. The homogeneous reaction conditions allow for the one-pot conversion of secondary amines to their arylated derivatives. Experimental and theoretical evidence supports the involvement of electrophilic aminium radicals which react *via* direct *ortho*-attack on the arene.

## Abstract 2

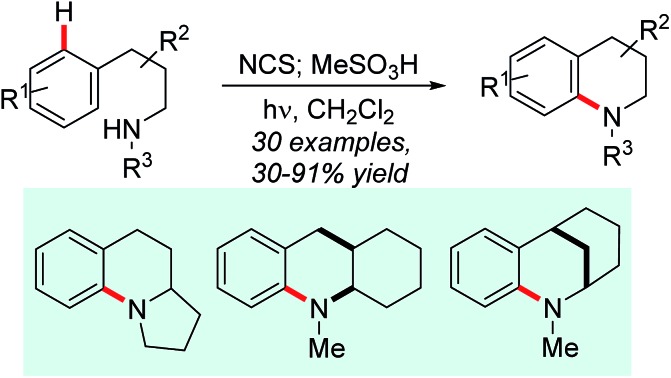
Direct radical-based substitution of aryl C–H bonds allows one-pot access to valuable polycyclic aryl amines from simple secondary amines.

## Introduction

Aromatic amines are fundamental components of a broad range of effect molecules including dyes, pharmaceuticals and agrochemicals.^1^ Studies within the pharmaceutical industry, for example, have shown that construction of aryl alkyl amines comprises *ca.* 5–10% of reactions carried out in the discovery phase^2^ and 5% of those undertaken in process development.^3^ Classically this motif is constructed by multi-step syntheses through *N*-alkylation of anilines or nucleophilic substitution of electron-poor haloarenes. Recent advances in metal-catalysed aminations of aromatic (pseudo)halides have been transformative,^4^ becoming one of the most significant reaction classes employed by industrial medicinal chemists.^5^ Nevertheless, the requirements for pre-functionalised aromatic (pseudo)halides and expensive precious metal catalysts and bespoke ligands are still limitations.

**Scheme 1:**
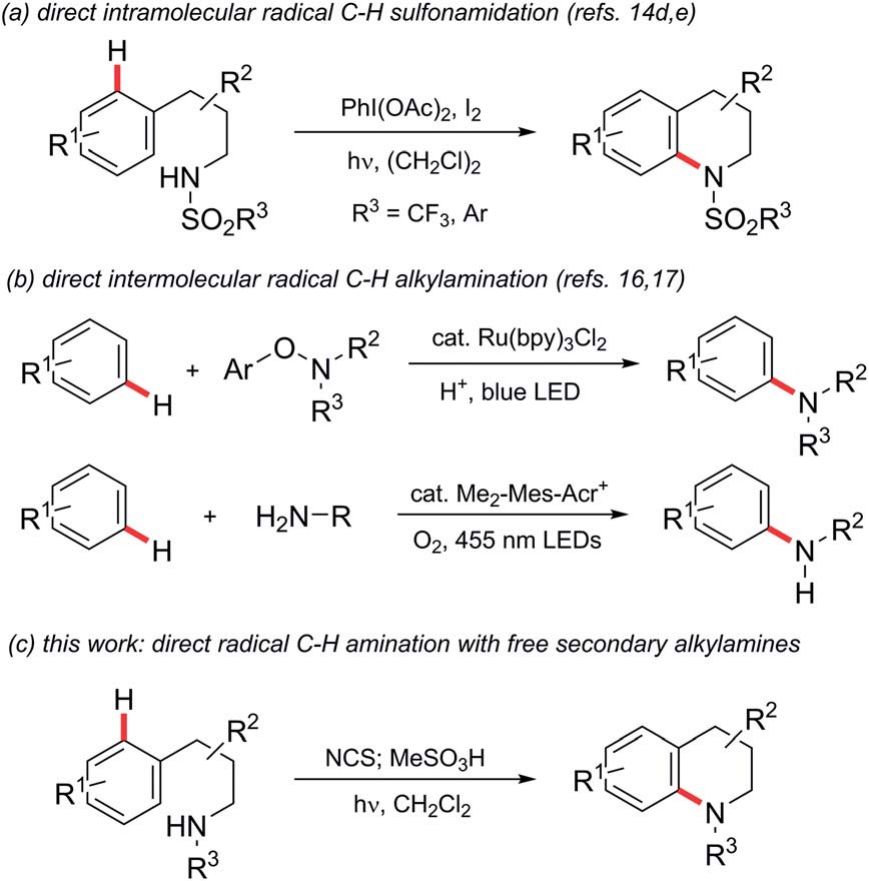
Direct radical C–H amination of arenes.

Interest in approaches to aromatic amines by the direct amination of aryl C–H bonds has therefore grown significantly. Metal-catalysed amination of substituted benzenes has been variously reported using electrophilic aminating species such as *O*-acyl-6 and *O*-sulfonylhydroxylamines,^7^ dioxazolones,^8^*N*-chloroamines,^9^ azides^10^ or amine derivatives in conjunction with oxidants,^11^ but in nearly all cases a coordinating group is required to direct C–H activation. The chemistry of nitrogen-centred radicals has seen a renaissance in recent years, and metal-catalysed intermolecular^12^ and photochemical/photoredox-mediated inter-13 and intramolecular^14^ methods for direct *N*-functionalisation of (hetero)arenes using such species have been reported,^15^ but are predominantly limited to the introduction of non-basic nitrogen substituents such as imides,12c,13c,d,f amides,13a,c phosphonamides14a,b or sulfonamides13b,g,h,14d,e (*e.g.*Scheme 1, panel a). There remains a significant need for a general direct amination of aromatic C–H bonds with simple alkylamine precursors. Outstanding progress has recently been made in this regard (Scheme 1, panel b): Leonori has demonstrated the amination of a range of mono- and bicyclic aromatics with secondary aminium radicals generated by photoredox-mediated homolysis of (2,4-dinitrophenyloxy)amines,^16^ while Nicewicz has demonstrated the intermolecular amination of (predominantly) electron-rich arenes with aminium radicals, generated directly from primary amines using acridinium photoredox catalysis coupled with aerobic oxidation.^17^

Despite these groundbreaking advances, a method for the direct arylation of secondary amines is still elusive: the aryloxyamine radical precursors employed successfully by Leonori require multi-step synthesis from secondary amines, while the direct functionalization reported by Nicewicz is thus far limited to primary amines.

Minisci and Kompa reported the direct amination of aromatics using aminium radicals generated from *N*-chloroamines in acidic media using, respectively, iron(ii) salts^18^ and UV photolysis.^19^ Despite the simplicity of these methods they have remarkably remained unexploited in the literature in the intervening 50 years.^20^ As noted elsewhere,^17^ the reasons for this are most likely due to the requirement for the use of preformed *N*-chloroamines which have a reputation as unstable/hazardous intermediates. Additionally, the reaction media were typically mixtures of concentrated sulfuric and acetic acid, which has limited scope as a medium for organic reactions and also precludes *in situ* generation of the radical precursors. Herein we describe the development of practical homogeneous conditions for *N*-chloroamine-mediated amination which: (a) allows us to demonstrate that the reaction has functional group tolerance, (b) allows access to a range of different polycyclic skeleta and, significantly, (c) facilitates the conversion of secondary amines to arylated derivatives in a single pot (Scheme 1, panel c). We also provide experimental and theoretical support for the involvement of electrophilic aminium radicals as intermediates.

## Results and discussion

Given the significance of the tetrahydroquinoline structure in a range of biologically active molecules,^21^ we began by examining the cyclisation of *N*-chloroamine **1a**, initially employing classical Hofmann–Löffler–Freytag (HLF) conditions in concentrated sulfuric acid^18^,^19^ (Table 1). Clean cyclisation to *N*-methyltetrahydroquinoline **2a** was observed in 81% yield (comparable to the non-photolytic literature variant mediated by FeCl_2_ (ref. 18b)), but as expected the viscous, heterogeneous reaction mixtures were difficult to process.

**Table 1:** Optimisation of the homogeneous amination reaction

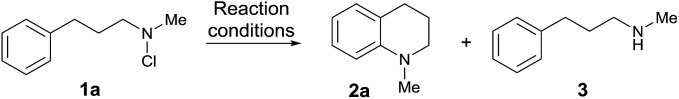			
Entry^*a*^	Reaction medium	Ratio **2** : **3**^*b*^	Yield **2a**^*c*^
1	c. H_2_SO_4_	100 : 0	81%
2^*d*^	c. H_2_SO_4_, FeCl_2_	n/a	81%
3	3 N HCl/MeOH	0 : 100	—
4	AcOH, 5 h	0 : 100	—
5	TFA, 5 h	55 : 27^*e*^	50%
6	MeSO_3_H/DCM (1 : 1)	100 : 0	80%
**7**	**MeSO**_**3**_ **H (10 eq.), DCM**	**100** : **0**	**91%**
8^*f*^	MeSO_3_H (10 eq.), DCM	0 : 100	—
9^*g*^	MeSO_3_H (10 eq.), DCM	25 : 75	17%

^*a*^Reaction conditions: **1a** (0.5 mmol.), 125W high pressure Hg-lamp, RT.

^*b*^Ratio of **2a** : **3**, determined by ^1^H NMR analysis.

^*c*^Isolated yield.

^*d*^Non-photolytic reaction using FeCl_2_ (result taken from ref. 18b).

^*e*^Plus 18% of chlorinated tetrahydroquinolines.

^*f*^In absence of UV or visible light.

^*g*^Using 24W visible light.

Amination was not observed in organic media such as acetic acid or methanolic HCl; in neat TFA amination occurred but ring-chlorinated products (presumably from competing electrophilic substitution pathways with the chloroamine as chlorinating agent^22^) were also observed. Reaction in mixtures of methanesulfonic acid and dichloromethane, however, gave clean conversion to **2a**, with best results found using 10 equivalents of acid (ESI†). Control experiments (entries 7, 8) confirmed the necessity for UV light irradiation.

We then applied the optimised conditions in an examination of the breadth and scope of the amination process, commencing with substitution in the non-aromatic ring (Table 2). Variation of the *N*-alkyl substituent was possible in products **2a–e**, with the tolerance of the removable *N*-benzyl substituent in **2b** being noteworthy from a synthetic perspective. Substitution in the aliphatic backbone of substrates **1** is also tolerated, allowing variously for incorporation of alkyl, alkenyl, aryl and heteroatom functionality at C2, 3 or 4 of the tetrahydroquinoline products **2f–n**. With longer alkyl chains on either the nitrogen atom or in the backbone, the potential for competing aliphatic C–H functionalisation through classical HLF chemistry exists, and indeed products of this process (<25%) accompanied the formation of the *N*-hexyl-substituted **2d**, lowering the isolated yield. However, the presence of abstractable hydrides in the carbon backbone was less problematic, illustrated by the relatively clean formation of the natural product angustureine^23^**2j** bearing a 2-pentyl substituent in 69% yield. Additionally, the potential for scission of the aminium radical intermediate where a radical-stabilising substituent is present on the beta-carbon18*b* means that 3-aryltetrahydroquinolines are not formed in preparatively useful yield.

**Table 2:** Substrate scope in the saturated heterocycle

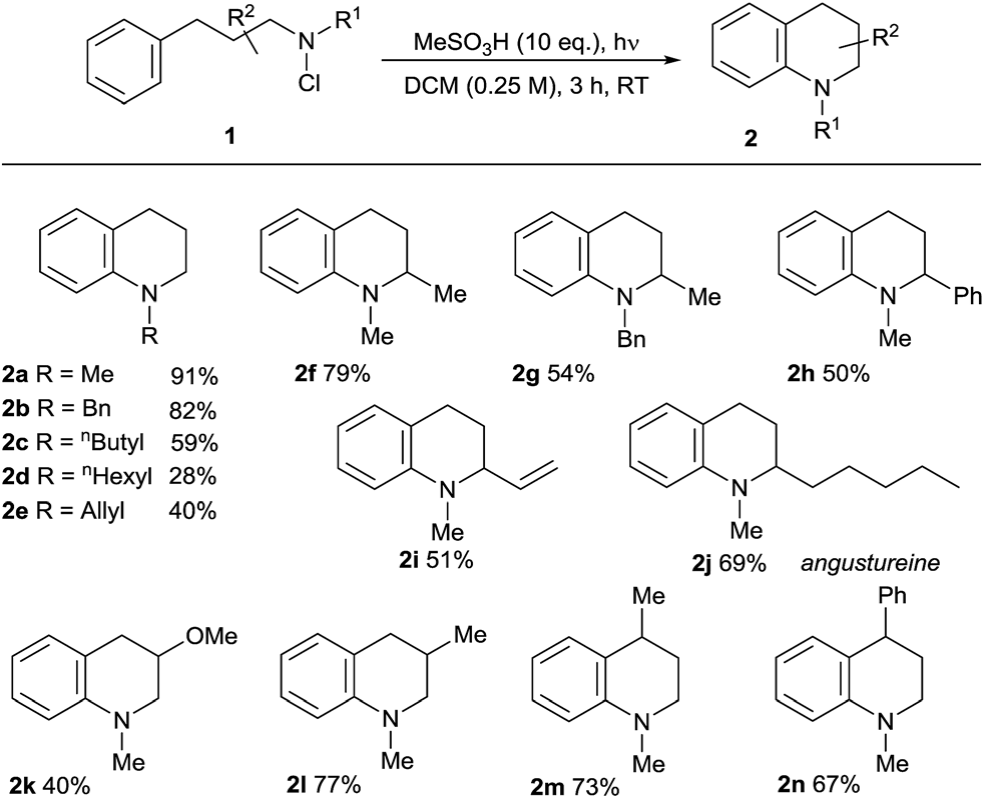

The scope of the reaction in terms of the aromatic partner was also investigated (Table 3). Both alkyl and aryl substituents are tolerated in the *ortho*-, *meta*- and *para*-positions of **1**. The effective cyclisation of *ortho*-substituted substrates notably leads to the effective formation of challenging contiguously trisubstituted arene structures **2r/v**. Alkyl and aromatic substituents are well tolerated, but the presence of an electron-withdrawing *para*-trifluoromethyl substitutent results in a lower conversion to **2q**, supporting the electrophilic character of the aminating species. We found that electron-rich arene-containing substrates (*e.g.* methoxyphenyl) were not tolerated, resulting in complex mixtures and evidence of direct electrophilic aromatic chlorination, presumably by the chloroamine agent itself.^22^ As expected, the use of *meta*-substituted substrates **1w/x** gives rise to a mixture of regioisomeric products with a moderate preference for the formation of the less-hindered C6-isomeric product. Most notably, the amination reaction demonstrates tolerance towards both halide substituents in **2s**, **t**, **x** and a boronate ester function in **2u**, which is synthetically significant, given the potential utility of both functionalities in downstream cross-coupling chemistries.

**Table 3:** Substrate scope in the aromatic ring

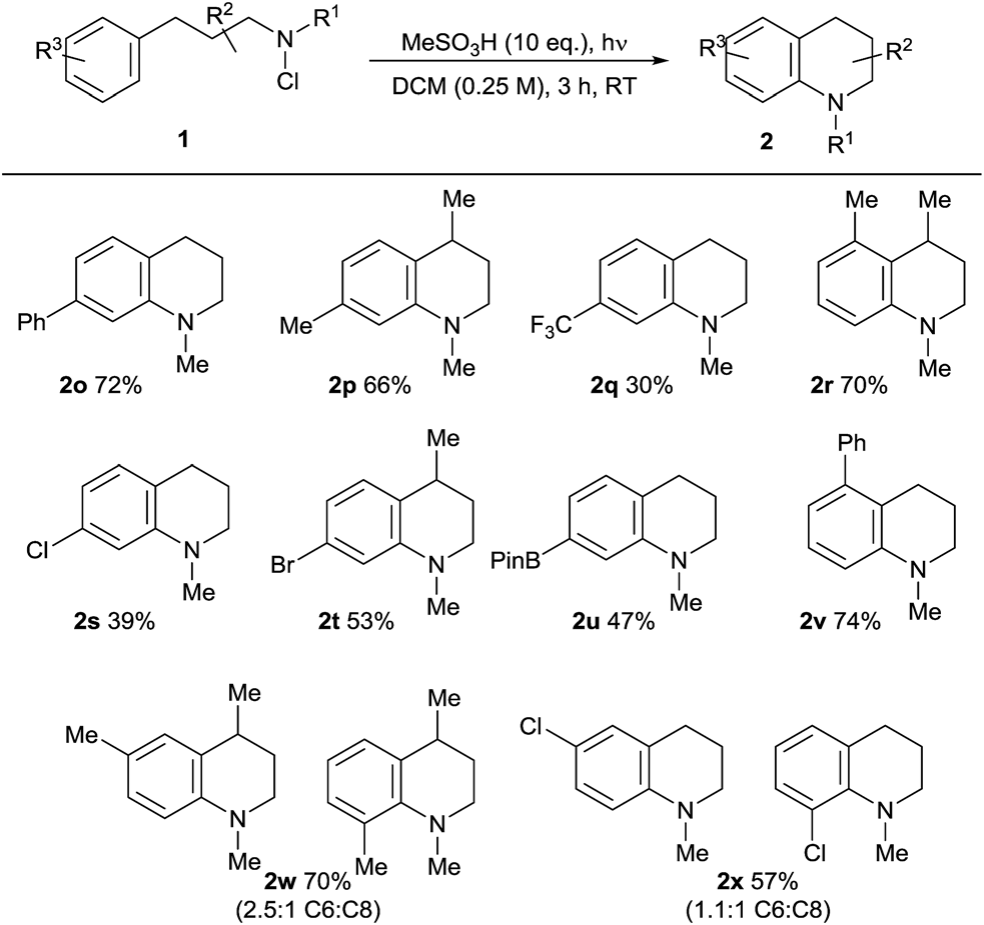

**Scheme 2:**
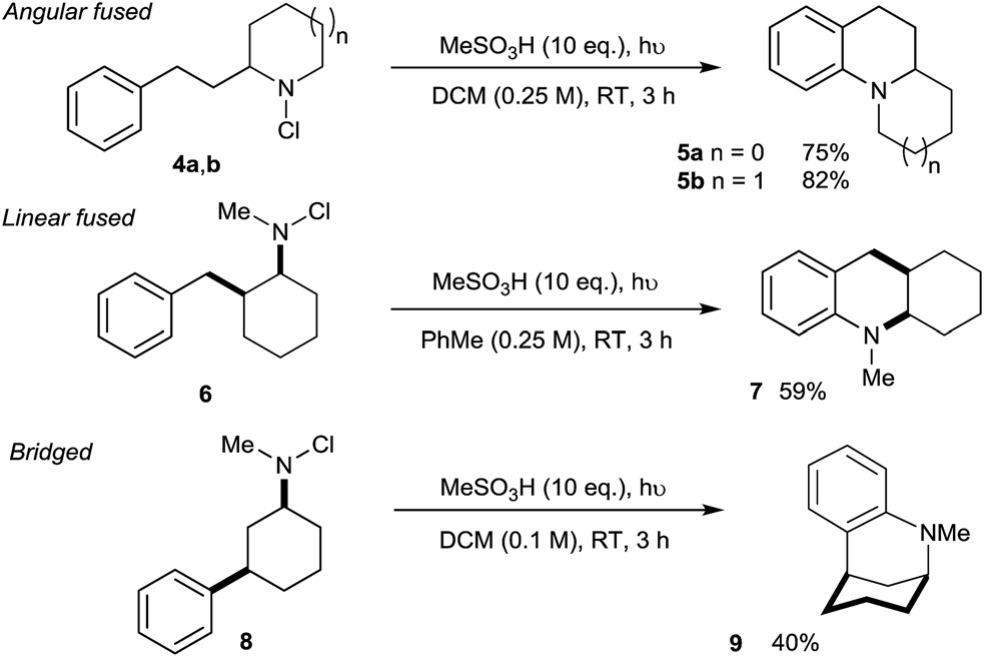
Tricyclic skeleta accessible through direct C–H amination.

We next assessed the potential of the direct amination in the preparation of diverse polycyclic assemblies (Scheme 2). We were pleased to find that the use of *N*-chloroamines **4a/b** derived from cyclic secondary amines lead to the highly effective synthesis of angularly-fused tricyclic amines **5a/b**, while the linearly-fused tricycle **7** was accessed efficiently from 2-benzylcyclohexylamine-derived **6**. Most remarkably, the *N*-chloroamine of the 3-phenylcyclohexylamine **8** underwent cyclisation to give bridged skeleton **9** (a framework found in complex alkaloids such as sespenine^24^), despite the requirement for a highly unfavourable 1,3-diaxial disposition of the reacting substituents in this conformationally-unbiased substrate.

**Scheme 3:**
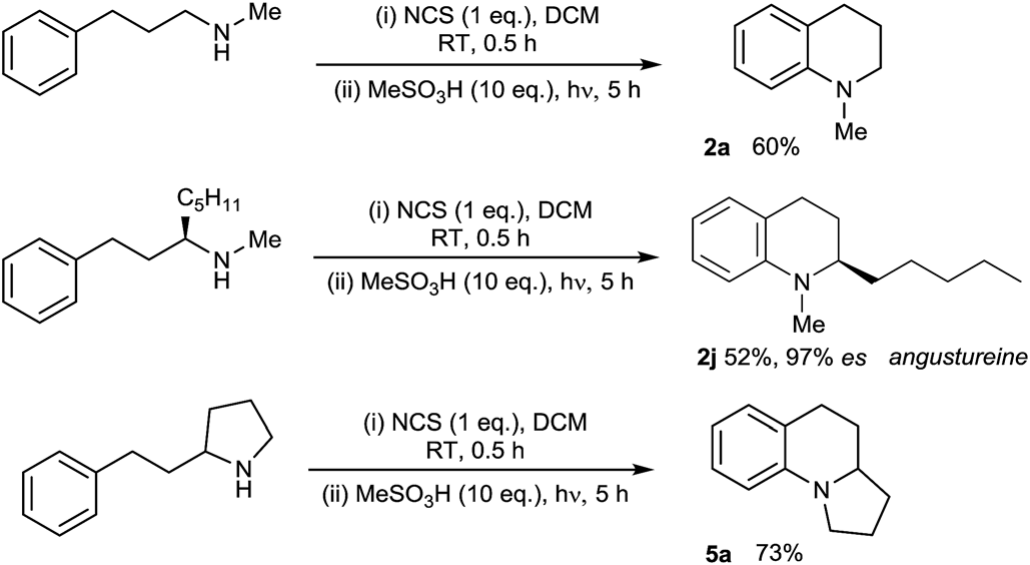
Direct one-pot arylation of secondary amines.

With the substrate scope of the protocol established, we next turned our attention to the development of a protocol for the direct amination of secondary amines in a single vessel. We were very pleased to find that this could be effected simply by treatment of the parent secondary amine with *N*-chlorosuccinimide (NCS) in dichloromethane followed by addition of MsOH and irradiation with UV light (Scheme 2). The presence of the succinimide by-product does not appear to adversely affect the direct amination, with isolated yields of the simple tetrahydroquinoline **2a**, the alkaloid angustureine **2j**, and the angularly-fused tricycle **5a** comparable to the two-step process (separate *N*-chlorination/amination) in both cases. The formation of enantioenriched **2j** proceeds with extremely high stereochemical fidelity, as predicted. This protocol constitutes the first examples of the one-pot metal-free arylation of secondary amines, and the operational simplicity of the method (obviating the need for prior functionalization of the nitrogen) should enable further synthetic applications of the method (Scheme 3).

**Scheme 4:**
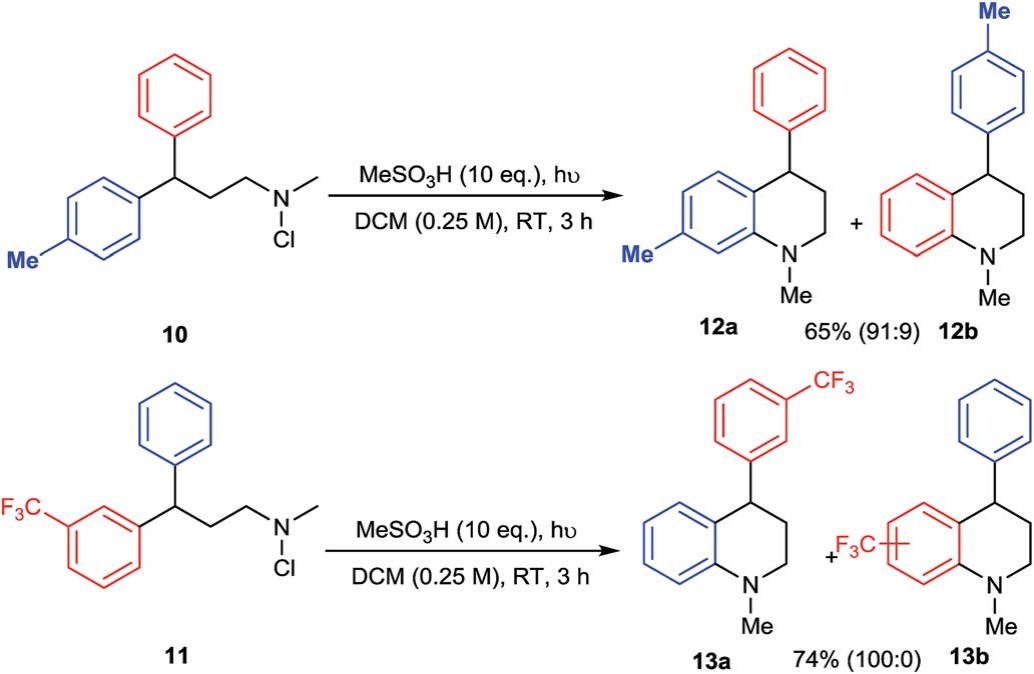
Intramolecular competition experiments.

The mechanism of amination by photolysis of *N*-chloroamines has been proposed to involve the intermediacy of protonated aminium radicals,12*d* but we sought to provide further evidence for the precise pathway to the tetrahydroquinoline products. The electrophilic nature of the aminating species was probed by internal competition experiments between differentially-substituted 3,3-diarylpropylamine substrates **10** and **11** (Scheme 4). In competition between a phenyl and a 4-methylphenyl substituent, amination of the more electron-rich ring in **10** predominates, favouring **12a** by a factor of 10 : 1, whereas in competition with a 3-trifluoromethyl group exclusive amination of the phenyl ring of **11** is observed, giving **13a**. Both results support a strongly electrophilic nature for the aminating species.

**Fig. 1:**
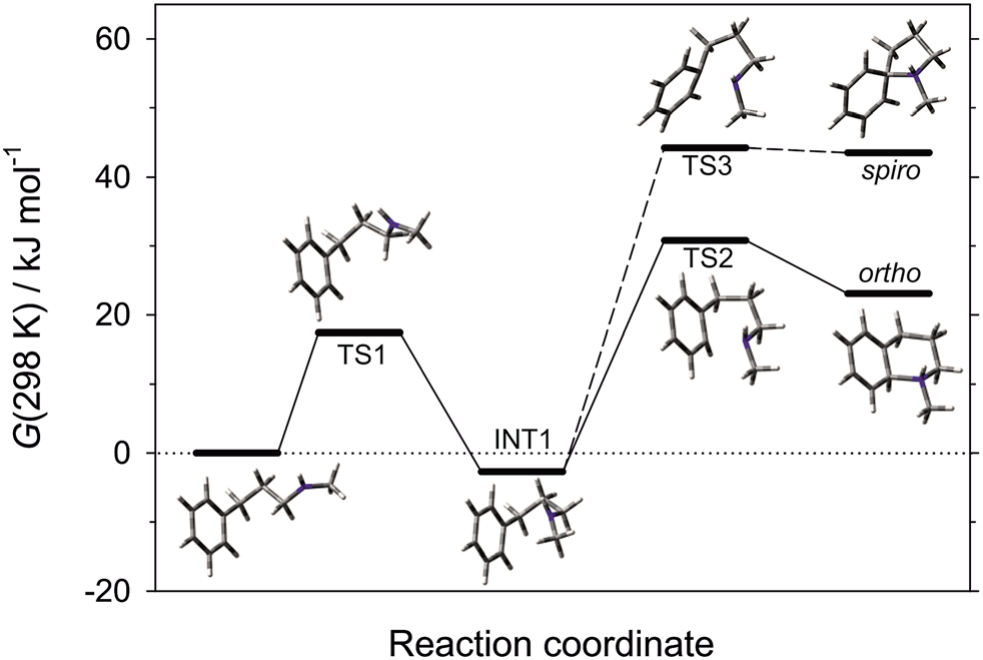
Energy plot for the cyclisation modes of aminium radical derived from **1a**.

The pathway of the cyclisation reaction was further probed by DFT calculations (B3LYP method with the 6-311+G(2d,p) triple zeta basis set; solvation by dichloromethane included by the Polarisable Continuum Model; see ESI† for full details). Although the ultimate product is that of *ortho*-amination, the intramolecular addition of radicals to arenes can arise through 5-membered *spiro*-addition or direct 6-membered *ortho*-addition modes, and examples of each manifold are known for heteroatom-centred radicals.^25^ Our calculations revealed that cyclisation of the neutral *N*-methyl-3-phenylpropan-1-aminyl radical in both *spiro*- and *ortho*-addition modes was, as expected, highly endergonic (Δ*G*(298 K) = 67 and 54 kJ mol^–1^, respectively), and was discounted. The cyclisations of the corresponding protonated aminium radicals were energetically more reasonable, consistent with the known rate enhancement in 1,5-hydrogen atom transfer^26^ and cyclisation to alkenes^27^ for aminium *versus* aminyl radicals. Of the two cyclisation pathways, the transition state for the *ortho*-cyclisation mode was found to be 14.2 kJ mol^–1^ more favourable than the *spiro*-mode and we conclude that this is the mode of attack (Fig. 1).

**Scheme 5:**
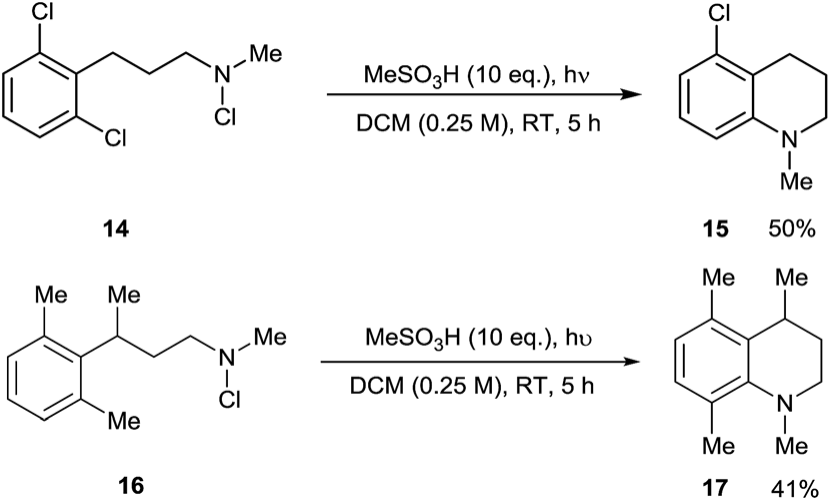
Substitution and rearrangement reactions of 2,6-disubstituted arene substrates.

In light of this, we examined the behaviour of 2,6-disubstituted substrates in which both *ortho*-positions are blocked. 2,6-Dichlorophenyl substrate **14** underwent formal C–Cl amination to give **15**, presumably *via ortho*-cyclisation and *ipso*-radical substitution. This constitutes a metal-free equivalent to Buchwald-Hartwig and Ullman amination reactions, occurring under acidic conditions which contrast with the basic conditions used in such processes. With 2,6-dimethylphenyl substrate **16**, the contiguously tetrasubstituted arene **17** was unexpectedly formed. This compound presumably arises by 1,2-methyl migration in the delocalised cyclohexadienyl radical cationic (Wheland) intermediate derived from this. Both of these reaction manifolds may find further synthetic application (Scheme 5).

## Conclusions

In summary, direct amination of aromatic compounds by photolytically-generated aminium radicals is revealed as an effective and functional group tolerant method for the construction of the valuable aryl dialkyl amine function. The reaction allows access to a range of polycyclic skeleta including bridged variants of relevance to complex alkaloids. The development of homogeneous reaction conditions for the amination allows for the direct one-pot conversion of secondary amines to their arylated derivatives under metal-free radical-based conditions. The mechanism has been probed experimentally and theoretically, with direct *ortho*-addition of electrophilic aminium radicals favoured. The functional group tolerance of the reaction coupled with the ability to access diverse polycyclic ring systems supports future applications in target synthesis.

## Conflicts of interest

There are no conflicts to declare.

## Supplementary Material

Supplementary information
