# Supplementary material for: Radical-mediated direct C–H amination of arenes with secondary amines
Source: Chem Sci. 2018 Jul 11;9(32):6647–52. doi: 10.1039/c8sc01747f (PMC6115623; doi:10.1039/c8sc01747f)
Supplement: Supplementary file 1 [file SC-009-C8SC01747F-s001.pdf]

Electronic Supplementary Material (ESI) for Chemical Science.
This journal is © The Royal Society of Chemistry 2018

Supporting Information - Contents

Radical-Mediated Direct C-H Amination of Arenes with Secondary Amines

S. C. Cosgrove, J. M. C. Plane, S. P. Marsden*

Supporting Information - Contents 1

Supporting information 2

1.1 General Experimental 2

1.2 Preparation of amines and characterisation data for intermediates 3

1.3 *N*-Chloroamine data 42

1.4 Tetrahydroquinoline data........ 57

1.5 One-pot procedures 74

1.6 Angustureine 11 stereoselective total synthesis 74

1.7 ^{1}H and ^{13}C NMR spectra for *N*-chloroamines 77

1.8 ^{1}H and ^{13}C NMR spectra for tetrahydroquinolines 106

1.9 NMR/HPLC traces for asymmetric synthesis of angustureine 11 138

1.10 DFT Calculations 140

1.11 Bibliography 148

Supporting information

1.1 General Experimental

Water-sensitive reactions were performed in oven- or flame-dried glassware cooled under nitrogen before use. Solvents were removed under reduced pressure using a Büchi rotary evaporator and a Vacuubrand PC2001 Vario diaphragm pump.

All other solvents and reagents were of analytical grade and used as supplied. Commercially available starting materials were obtained from Sigma–Aldrich, Alfa Aesar and Fluorochem.

Flash column chromatography was carried out using silica (35-70 µm particles). Thin layer chromatography was carried out on commercially available pre-coated aluminium plates (Merck silica 2 8 8 0 Kieselgel 60F254).

Analytical LC-MS was performed using a system comprising of a Bruker HCT Ultra ion trap mass spectrometer equipped with electrospray ionization and an Agilent 1200 series LC made up of, a high vacuum degasser, a binary pump, a high performance autosampler, an autosampler thermostat, a thermostated column compartment and diode array detector. The system used a Phenomenex Luna C18 50 × 2 mm 5 micron column and elution was effected with a binary gradient of two solvent systems: MeCN/H_2O + 0.1% Formic acid or MeCN/H_2O.

Proton and carbon NMR spectra were recorded on a Bruker Avance DPX 300, Avance 500, AV-3 400 or DRX 500 or JEOL ECA600II spectrometer using an internal deuterium lock. Carbon NMR spectra were recorded with composite pulse decoupling using the watts 16 pulse sequence. DEPT, COSY, HMQC and HMBC pulse sequences were routinely used to aid the assignment of spectra. Chemical shifts are quoted in parts per million downfield of tetramethylsilane, and coupling constants (J) are given in Hz. NMR spectra were recorded at 300 K unless otherwise stated.

Melting points were determined on a Reichert hot stage microscope and are uncorrected.

Infrared spectra were recorded on a Bruker alpha FT-IR spectrometer using a “platinum ATR” accessory and are reported in wavenumbers (cm^{-1}).

Nominal mass spectrometry was routinely performed on a Bruker HCT Ultra spectrometer using electrospray (+) ionization. Nominal and accurate mass spectrometry using electrospray ionisation was carried in the School of Chemistry at the University of Leeds, using a Bruker MaXis Impact spectrometer.

Photochemical reactions were conducted using a quartz immersion well reactor and 125 W medium pressure mercury lamp supplied by Photochemical Reactors Ltd.

1.2 Preparation of amines and characterisation data for intermediates

Route A

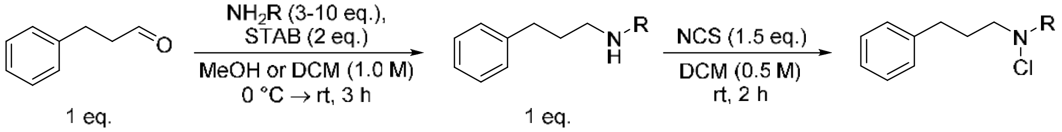

General Procedure A: Reductive amination

To a stirred solution of carbonyl compound (1.0 eq.) in anhydrous MeOH or DCM (1.0 M) at 0 °C was added amine (3-10 eq.) and the reaction mixture was stirred for 15 mins. To this was added sodium trisacetoxyborohydride (2.0 eq.) portionwise, and the reaction mixture was warmed to rt, stirred for 3 h then the reaction was quenched with sat. aqueous $NaHCO_3$ (3 vol). The aqueous phase was extracted with EtOAc (× 3) and the combined organics were dried over Na_2SO_4 and concentrated *in vacuo*. Purification afforded the desired product.

General Procedure B: Chlorination reaction

Following a modified procedure by De Luca *et al.*,[1] to a stirred solution of amine (1.0 eq.) in DCM (0.20 M) at rt in a flask covered by aluminium foil was added NCS (1.5 eq.) portionwise and the reaction mixture was stirred for 3 h then concentrated *in vacuo*. Purification by flash chromatography afforded the desired product.

N-Methyl-3-phenylpropan-1-amine 3

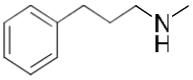

Following general procedure A, using hydrocinnamaldehyde (6.71 g, 50.0 mmol) and $MeNH_2$ (50 mL of an 8.0 M solution in EtOH, 10.0 eq.) afforded the title compound (7.45 g, 49.9 mmol, 99%) as a colourless oil. The data is in accordance with the literature.[2]

^{1}H NMR (300 MHz, $CDCl_3$) δ 7.32-7.24 (2H, m, ArH), 7.22-7.15 (3H, m, ArH), 2.70-2.57 (4H, m, includes 2H, m, propyl H_2-C3; and 2H, m, propyl H_2-C1), 2.43 (3H, s, NCH_3), 1.87-1.76 (2H, m, propyl H_2-C2);

^{13}C NMR (75 MHz, $CDCl_3$) δ 142.2 (C_q), 128.4 (2 × C, ArC), 128.3 (2 × C, ArC), 125.7 (ArC), 51.7 (propyl C1), 36.5 (NCH_3), 33.6 (propyl C3), 31.6 (propyl C2);

IR ν_{max} (neat)/cm^{-1}: 3308 (N-H), 2932, 2854, 1603, 1495, 1472, 1453, 1112;

HRMS (ESI): $C_{10}H_{16}N$ [M+H$^+$]: calculated 150.1277, found 150.1289.

N-Benzyl-3-phenylpropan-1-amine

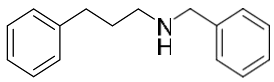

Following general procedure A, using hydrocinnamaldehyde (1.00 mL, 7.45 mmol) and benzylamine (2.44 mL, 22.4 mmol). Purification by flash chromatography on silica gel, eluting with a gradient of 25-100% EtOAc in hexane afforded the title compound (899 mg, 3.99 mmol, 54%) as a yellow oil. The data is in accordance with the literature.[3]

^{1}H NMR (300 MHz, $CDCl_3$) δ 7.50-7.14 (10H, m, ArH), 3.85 (2H, s, NCH_2Ph), 2.82-2.65 (4H, m, includes 2H, m, propyl H_2-C1; and 2H, m, propyl H_2-C3), 1.99-1.85 (2H, m, propyl H_2-C2);

^{13}C NMR (75 MHz, $CDCl_3$) δ 142.2 (C_q), 140.5 (C_q), 128.4 (4 × C, ArC), 128.3 (4 × C, ArC), 128.1 (ArC), 126.9 (ArC), 54.0 (NCH_2Ph), 48.9 (propyl C1), 33.6 (propyl C3), 31.7 (propyl C2);

IR ν_{max} (neat)/cm^{-1}: 3025, 2927, 2856, 2813, 1602, 1494, 1452, 1171;

HRMS (ESI): $C_{16}H_{20}N$ [M+H$^+$]: calculated 226.1590, found 226.1595.

N-(3-Phenylpropyl)butan-1-amine

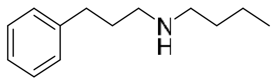

Following general procedure A, using hydrocinnamaldehyde (0.98 mL, 7.45 mmol) and *n*-butylamine (3.69 mL, 37.3 mmol). Purification by SCX cartridge afforded the *title compound* (1.02 g, 5.33 mmol, 72%) as a yellow oil.

^{1}H NMR (500 MHz, $CDCl_3$) δ 7.32-7.23 (2H, m, ArH), 7.22-7.15 (3H, m, ArH), 2.69-2.61 (4H, m, includes 2H, m, propyl H_2-C3; and 2H, m, propyl H_2-C1), 2.59 (2H, t, *J* = 7.6, butyl H_2-C1), 1.87-1.77 (2H, m, propyl H_2-C2), 1.50-1.42 (2H, m, butyl H_2-C2), 1.38-1.29 (2H, m, butyl H_2-C3), 0.91 (3H, t, *J* = 7.3, butyl H_3-C4);

^{13}C NMR (75 MHz, $CDCl_3$) δ 142.2 (C_q), 128.4 (2 × C, ArC), 128.3 (2 × C, ArC), 125.7 (ArC), 49.8 (butyl C1), 49.6 (propyl C1), 33.7 (propyl C3), 32.4 (butyl C2), 31.8 (propyl C2), 20.5 (butyl C3), 14.0 (butyl C4);

IR ν_{max} (neat)/cm^{-1}: 3026, 2955, 2927, 2858, 1496, 1454, 1128, 697;

HRMS (ESI): $C_{13}H_{22}N$ [M+H$^+$]: calculated 192.1747, found 192.1750.

N-(3-Phenylpropyl)hexan-1-amine

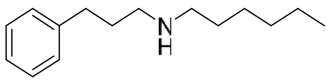

Following general procedure A, using hydrocinnamaldehyde (1.00 mL, 7.45 mmol) and *n*-hexylamine (4.92 mL, 37.3 mmol). Purification by flash chromatography on silica gel, eluting

with a gradient of 25-100% EtOAc in hexane afforded the *title compound* (260 mg, 1.19 mmol, 16%) as a colourless oil.

^{1}H NMR (300 MHz, $CDCl_3$) δ 7.32-7.23 (2H, m, ArH), 7.21-7.16 (3H, m, ArH), 2.70-2.54 (6H, m, includes 2H, m, propyl H_2-C1; 2H, m, propyl H_2-C3; 2H, m, hexyl H_2-C1), 1.88-1.74 (2H, m, propyl H_2-C2), 1.51-1.41 (2H, m, hexyl H_2-C2), 1.37-1.21 (6H, m, hexyl H_2-C3-5), 0.93-0.83 (3H, m, hexyl H_3-C6);

^{13}C NMR (75 MHz, $CDCl_3$) δ 142.2 (C_q), 128.4 (2 × C, ArC), 128.3 (2 × C, ArC), 125.7 (ArC), 50.1 (CH_2), 49.6 (CH_2), 33.7 (CH_2), 31.8 (2 × C, CH_2), 30.2 (CH_2), 27.1 (CH_2), 22.6 (CH_2), 14.0 (CH_3);

IR ν_{max} (neat)/cm^{-1}: 3026, 2925, 2855, 1603, 1495, 1454, 1129, 687;

HRMS (ESI): $C_{15}H_{26}N$ [M+H$^+$]: calculated 220.2060, found 220.2063.

N-(3-Phenylpropyl)prop-2-en-1-amine

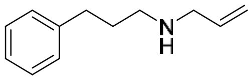

Following general procedure A, using hydrocinnamaldehyde (0.98 mL, 7.45 mmol) and allylamine (2.79 mL, 37.3 mmol). Purification by flash chromatography on silica gel, eluting with a gradient of 50-100% EtOAc in hexane afforded the *title compound* (624 mg, 3.56 mmol, 48%) as a colourless oil.

^{1}H NMR (500 MHz, $CDCl_3$) δ 7.32-7.23 (2H, m, ArH), 7.23-7.15 (3H, m, ArH), 5.91 (1H, ddt, *J* = 16.8, 10.3, 6.0, propenyl H-C2), 5.17 (1H, dd, *J* = 16.8, 1.5, propenyl H_{trans}-C3), 5.09 (1H, dd, *J* = 10.3, 1.5, propenyl H_{cis}-C3), 3.25 (2H, dt, *J* = 6.0, 1.3, propenyl H_2-C1), 2.70-2.61 (4H, m, includes 2H, m, propyl H_2-C1; 2H, m, propyl H_2-C3), 1.88-1.80 (2H, m, propyl H_2-C2);

^{13}C NMR (125 MHz, $CDCl_3$) δ 142.3 (C_q), 137.1 (propenyl C2), 128.5 (2 × C, ArC), 128.5 (2 × C, ArC), 125.9 (ArC), 115.9 (propenyl C3), 52.6 (propenyl C1), 49.1 (propyl C1), 33.8 (propyl C3), 31.9 (propyl C2);

IR ν_{max} (neat)/cm^{-1}: 3063, 3026, 2927, 2857, 2812, 1643 (C=C), 1603, 1453;

HRMS (ESI): $C_{12}H_{18}N$ [M+H$^+$]: calculated 176.1434, found 176.1432.

Route B

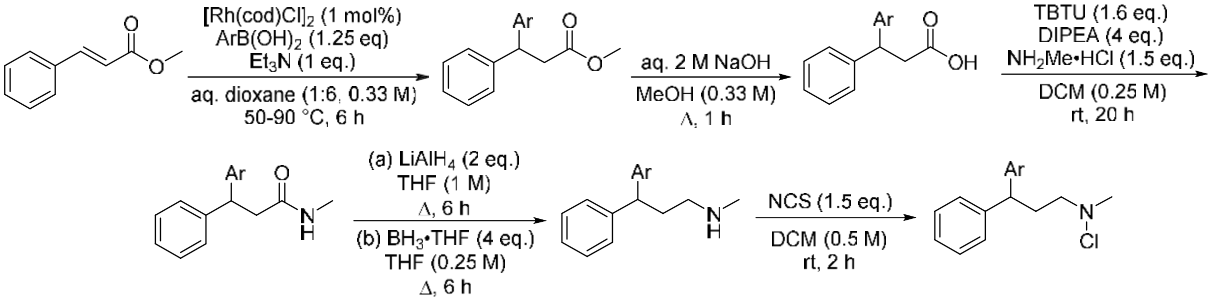

General Procedure C: Rh(I)-catalysed 1,4-conjugate addition

Following a procedure by Miyaura *et al.*,[4] to a stirred solution of $[Rh(cod)Cl]_2$ (1 mol%) and $ArB(OH)_2$ (1.25-1.50 eq.) in degassed aqueous dioxane (6:1, 0.33 M) was added a solution of α,β-unsaturated carbonyl compound (1.0 eq.) in aqueous dioxane and freshly distilled and degassed Et_3N (1.0 eq.) simultaneously. The reaction mixture was heated at 50 °C for 6 h, after which it was cooled to rt, concentrated *in vacuo* and purified by flash chromatography on silica gel to afford the desired product.

General Procedure D: Ester hydrolysis

A solution of ester (1.0 eq.) in MeOH and 2 M aqueous NaOH (0.33 M, 1:1 v/v) was heated at reflux for 1 h. The reaction mixture was then cooled to rt and diluted with 2 M aqueous HCl (2 vol). The aqueous phase was extracted with EtOAc (× 3) and the combined organic extracts were washed with sat. brine solution (2 vol), dried over Na_2SO_4 and concentrated *in vacuo*. Purification afforded the desired product.

General Procedure E: TBTU mediated peptidic coupling

To a solution of acid (1.0 eq.) in anhydrous DCM (0.25 M) was added TBTU (1.6 eq.), DIPEA (4.0 eq.) and NH_2Me•HCl (1.5 eq.). The reaction mixture was stirred at rt for 20 h then was quenched with sat. aqueous $NaHCO_3$ solution (1 vol) and the phases separated. The aqueous phase was extracted with DCM (× 3) and the combined organic extracts were washed with sat. brine solution (1 vol), dried over Na_2SO_4 and concentrated *in vacuo*. Purification afforded the desired product.

General Procedure F: $LiAlH_4$ reduction

To a stirred suspension of $LiAlH_4$ (2.0-4.0 eq.) in THF (1.0 M) at 0 °C was added a solution of reactant in THF (0.5 M) dropwise. The reaction mixture was then heated at reflux for 2-6 h. The reaction mixture was cooled to 0 °C the quenched sequentially with the dropwise addition of H_2O (1.0 eq.), 2 M aqueous NaOH (1.0 eq.) and H_2O (5.0 eq.) then stirred for 1 h at rt until the reaction

mixture had turned colourless. The resultant slurry was dried over Na_2SO_4, filtered through a pad of Celite and the pad of Celite was washed with EtOAc. The filtrate was concentrated *in vacuo*. Purification afforded the desired products.

General Procedure G: Borane reduction

To a stirred solution of amide (1.0 eq.) in THF (0.25 M) at 0 °C was added a solution of BH_3 (4.0 eq.) dropwise. The reaction mixture was stirred at 0 °C for 15 mins, then heated to reflux and stirred for 6 h, after which it was cooled to 0 °C and the reaction was quenched with the dropwise addition of 4 M aqueous NaOH (1 vol). The phases were then separated, and the aqueous phase was extracted with EtOAc (× 3) then the combined organic extracts were washed with sat. brine solution (1 vol), dried over Na_2SO_4 and concentrated *in vacuo*. Purification afforded the desired product.

Methyl 3,3-diphenylpropanoate

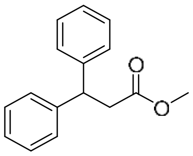

Following general procedure C, using methyl *trans*-cinnamate (1.22 g, 7.5 mmol) and $PhB(OH)_2$ (1.14 g, 9.38 mmol). Purification by flash chromatography on silica gel, eluting with 10% EtOAc in hexane afforded the title compound (1.73 g, 7.20 mmol, 96%) as a bright yellow oil. The data is in accordance with the literature.[5]

^{1}H NMR (300 MHz, $CDCl_3$) δ 7.35-7.15 (10H, m, ArH), 4.57 (1H, t, *J* = 8.0, propyl H-C3), 3.59 (3H, s, OCH_3), 3.08 (2H, d, *J* = 8.0, propyl H_2-C2);

^{13}C NMR (75 MHz, $CDCl_3$) δ 172.3 (propyl C1), 143.5 (2 × C, C_q), 128.6 (4 × C, ArC), 127.6 (4 × C, ArC), 126.5 (2 × C, ArC), 51.7 (OCH_3), 47.0 (propyl C3), 40.6 (propyl C2);

IR ν_{max} (neat)/cm^{-1}: 3340, 3028, 2951, 1732 (C=O), 1637, 1493, 1430, 1253;

HRMS (ESI): $C_{16}H_{16}O_2Na$ [M+Na$^+$]: calculated 263.1043, found 263.1045.

3,3-Diphenylpropanoic acid

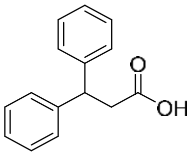

Following general procedure D, using methyl 3,3-diphenylpropanoate (1.50 g, 6.24 mmol) afforded the title compound (1.41 g, 6.23 mmol, 99%) as a colourless solid, which was used without further purification. A small quantity was crystallised from 9:1 hexane-EtOAc. The data is in accordance with the literature.[6]

M.p 155-158 °C, colourless needles, hexane-EtOAc;

^{1}H NMR (300 MHz, MeOD) δ 7.31-7.23 (8H, m, ArH), 7.20-7.12 (2H, m, ArH), 4.50 (1H, t, *J* = 8.0, propyl H-C3), 3.04 (2H, d, *J* = 8.0, propyl H_2-C2);

^{13}C NMR (75 MHz, MeOD) δ 175.6 (propyl C1), 145.3 (2 × C, C_q), 129.5 (4 × C, ArC), 128.8 (4 × C, ArC), 127.4 (2 × C, ArC), 48.5 (propyl C3), 41.5 (propyl C2);

IR ν_{max} (neat)/cm^{-1}: 3027 (O-H), 2910, 1695 (C=O), 1597, 1493, 1427, 1268, 919;

HRMS (ESI): $C_{15}H_{14}O_2Na$ [M+Na$^+$]: calculated 249.0886, found 249.0886.

N-Methyl-3,3-diphenylpropanamide

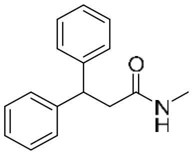

Following general procedure E, using 3,3-diphenylpropanoic acid (1.25 g, 5.52 mmol). Purification by flash chromatography on silica gel, eluting with 50% EtOAc in hexane afforded the *title compound* (1.27 g, 5.31 mmol, 96%) as an amorphous solid.

^{1}H NMR (300 MHz, $CDCl_3$) δ 7.26-7.06 (10H, m, ArH), 5.16 (1H, br. s, NH), 4.50 (1H, t, *J* = 7.8, propyl H-C3), 2.80 (2H, d, *J* = 7.8, propyl H_2-C2), 2.56 (3H, app. d, *J* = 4.9, NCH_3);

^{13}C NMR (75 MHz, $CDCl_3$) δ 171.7 (propyl C1), 143.7 (2 × C, C_q), 128.6 (4 × C, ArC), 127.7 (4 × C, ArC), 126.5 (2 × C, ArC), 47.3 (propyl C3), 43.3 (propyl C2), 26.3 (NCH_3);

IR ν_{max} (neat)/cm^{-1}: 3341 (N-H), 3030, 2941, 1638 (C=O), 1601, 1550, 1492, 745;

HRMS (ESI): $C_{16}H_{17}NONa$ [M+Na$^+$]: calculated 262.1202, found 262.1203.

N-Methyl-3,3-diphenylpropan-1-amine

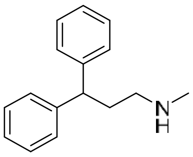

Following general procedure F, using *N*-methyl-3,3-diphenylpropanamide (1.00 g, 4.18 mmol, 1.0 eq.). Purification by flash chromatography on silica gel, eluting with EtOAc then 5% MeOH in DCM, afforded the title compound (574 mg, 2.55 mmol, 61%) as a colourless gum.

^{1}H NMR (300 MHz, $CDCl_3$) δ 7.32-7.23 (8H, m, ArH), 7.21-7.14 (2H, m, ArH), 4.01 (1H, t, *J* = 7.8, propyl H-C3), 2.55 (2H, t, *J* = 7.4, propyl H_2-C1), 2.39 (3H, s, NCH_3), 2.26 (2H, app. dd, *J* = 14.9, 7.4, propyl H_2-C2);

^{13}C NMR (75 MHz, $CDCl_3$) δ 144.7 (2 × C, C_q), 128.5 (4 × C, ArC), 127.8 (4 × C, ArC), 126.2 (2 × C, ArC), 50.4 (propyl C1), 49.0 (propyl C3), 36.3 (NCH_3), 35.5 (propyl C2);

IR ν_{max} (neat)/cm^{-1}: 3060, 2931 (N-H), 2843, 2791, 1599, 1493, 1469, 1030;

HRMS (ESI): $C_{16}H_{20}N$ [M+H$^+$]: calculated 226.1590, found 226.1590.

Methyl 3-phenyl-3-(3-(trifluoromethyl)phenyl)propanoate

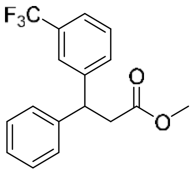

Following general procedure C, using methyl *trans*-cinnamate (1.22 g, 7.50 mmol) and *m*-$CF_3PhB(OH)_2$ (1.78 g, 9.38 mmol). Purification by flash chromatography on silica gel, eluting with a gradient of 25-50% DCM in hexane afforded the title compound (1.01 g, 3.28 mmol, 44%) as a colourless gum.

^{1}H NMR (300 MHz, $CDCl_3$) δ 7.52-7.38 (4H, m, ArH), 7.35-7.27 (2H, m, ArH), 7.25-7.19 (3H, m, ArH), 4.62 (1H, t, *J* = 8.0, propyl H-C3), 3.59 (3H, s, OCH_3), 3.08 (2H, d, *J* = 7.9, propyl H_2-C2);

^{13}C NMR (125 MHz, $CDCl_3$) δ 171.8 (propyl C1), 144.4 (ArC), 142.5 (ArC), 131.1 (ArC), 130.9 (q, *J* = 32.1, C_q), 129.0 (ArC), 128.8 (2 × C, ArC), 127.6 (2 × C, ArC), 126.9 (ArC), 124.4 (q, *J* = 3.8, ArC), 124.1 (q, *J* = 272.4, CF_3), 123.5 (q, *J* = 3.8, ArC), 51.7 (CH_3), 46.8 (propyl C3), 40.4 (propyl C2)**;**

IR ν_{max} (neat)/cm^{-1}: 3030, 2954, 1736 (C=O), 1600, 1495, 1438, 1326, 1119;

HRMS (ESI): $C_{17}H_{15}F_3O_2Na$ [M+Na$^+$]: calculated 331.0916, found 331.0920.

3-Phenyl-3-(3-(trifluoromethyl)phenyl)propanoic acid

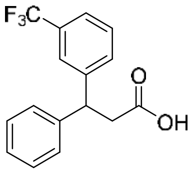

Following general procedure D, using methyl 3-phenyl-3-(3-(trifluoromethyl)phenyl)propanoate (900 mg, 2.92 mmol). Crystallisation of the crude solid from a 1:1 hexane-EtOAc mixture afforded the *title compound* (551 mg, 1.87 mmol, 64%) as a colourless crystalline solid.

M.p 88-90 °C, crystalline solid, hexane-EtOAc;

^{1}H NMR (300 MHz, $CDCl_3$) δ 7.55-7.16 (9H, m, ArH), 4.58 (1H, t, *J* = 7.9, propyl H-C3), 3.10 (2H, t, *J* = 7.9, propyl H_2-C2);

^{13}C NMR (125 MHz, $CDCl_3$) δ 177.5 (propyl C1), 144.2 (ArC), 142.2 (ArC), 131.0 (ArC), 131.0 (q, *J* = 32.1, C_q), 129.1 (ArC), 128.9 (2 × C, ArC), 127.5 (2 × C, ArC), 127.0 (ArC), 124.4 (q, *J* = 3.8, ArC), 124.0 (q, *J* = 272.4, CF_3), 123.6 (q, *J* = 3.8, ArC), 46.4 (propyl C3), 40.2 (propyl C2);

IR ν_{max} (neat)/cm^{-1}: 3064 (O-H), 2910, 1704 (C=O), 1598, 1498, 1449, 1429, 1406;

HRMS (ESI): $C_{16}H_{13}F_3O_2Na$ [M+Na$^+$]: calculated 317.0760, found 317.0759.

N-Methyl-3-phenyl-3-(3-(trifluoromethyl)phenyl)propanamide

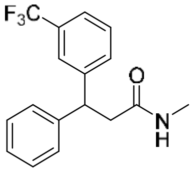

Following general procedure E, using 3-phenyl-3-(3-(trifluoromethyl)phenyl)propanoic acid (940 mg, 3.21 mmol). Purification by flash chromatography on silica gel, eluting with 25% EtOAc in pentane afforded the *title compound* (648 mg, 2.11 mmol, 66%) as a colourless oil.

^{1}H NMR (500 MHz, $CDCl_3$) δ 7.51-7.36 (4H, m, ArH), 7.33-7.27 (2H, m, ArH), 7.24-7.17 (3H, m, ArH), 5.42 (1H, s, NH), 4.68 (1H, t, *J* = 7.7, propyl H-C3), 2.88 (2H, app. dd, *J* = 7.7, 3.3, propyl H_2-C2), 2.66 (3H, app. d, *J* = 4.8, NCH_3);

^{13}C NMR (125 MHz, $CDCl_3$) δ 171.1 (propyl C1), 144.8 (C_q), 142.8 (C_q), 131.4 (ArC), 130.8 (q, *J* = 32.0, C_q), 129.0 (ArC), 128.7 (2 × C, ArC), 127.7 (2 × C, ArC), 126.9 (ArC), 124.2 (q, *J* = 3.8, ArC), 124.1 (q, *J* = 272.4, CF_3), 123.4 (q, *J* = 3.8, ArC), 47.0 (propyl C3), 42.9 (propyl C2), 26.2 (NCH_3);

IR ν_{max} (neat)/cm^{-1}: 3285 (N-H), 3090, 3030, 2945, 1640 (C=O), 1562, 1495, 1411;

HRMS (ESI): $C_{17}H_{16}F_3NO$ [M+H$^+$]: calculated 308.1257, found 308.1244.

N-Methyl-3-phenyl-3-(3-(trifluoromethyl)phenyl)propan-1-amine

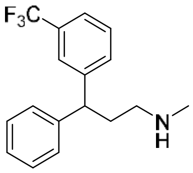

Following general procedure G, using *N*-methyl-3-phenyl-3-(3-(trifluoromethyl)phenyl)propanamide (640 mg, 2.08 mmol). Purification by SCX cartridge afforded the *title compound* (201 mg, 0.69 mmol, 33%) as a yellow oil.

^{1}H NMR (500 MHz, $CDCl_3$) δ 7.21-7.10 (4H, m, ArH), 7.07-6.92 (5H, m, ArH), 3.85 (1H, t, *J* = 7.8, propyl H-C3), 2.28 (2H, t, *J* = 7.2, propyl H_2-C1), 2.14 (3H, s, NCH_3), 2.05-1.95 (2H, m, propyl H_2-C2);

^{13}C NMR (125 MHz, $CDCl_3$) δ 145.8 (C_q), 143.7 (C_q), 131.2 (ArC), 130.7 (q, *J* = 32.0, C_q), 128.9 (ArC), 128.7 (2 × C, ArC), 127.8 (2 × C, ArC), 126.6 (ArC), 124.4 (q, *J* = 3.8, ArC), 124.2 (q, *J* = 272.1, CF_3), 123.1 (q, *J* = 3.5, ArC), 50.1 (propyl C1), 48.8 (propyl C3), 36.3 (NCH_3), 35.4 (propyl C2);

IR ν_{max} (neat)/cm^{-1}: 3028, 2934, 2850, 2797, 1599, 1494, 1474, 1325;

HRMS (ESI): $C_{17}H_{19}F_3N$ [M+H$^+$]: calculated 294.1464, found 294.1463.

Methyl 3-phenyl-3-(*p*-tolyl)propanoate

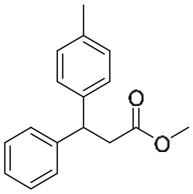

Following general procedure C, using methyl *trans*-cinnamate (1.22g, 7.50 mmol) and *p*-MePhB(OH)$_2$ (1.28 g, 9.38 mmol). Purification by flash chromatography on silica gel, eluting with 25% DCM in pentane afforded the title compound (625 mg, 2.46 mmol, 33%) as a pale yellow oil. The data is in accordance with the literature.[7]

^{1}H NMR (300 MHz, $CDCl_3$) δ 7.31-7.06 (9H, m, ArH), 4.52 (1H, t, *J* = 8.0, propyl H-C3), 3.58 (3H, s, OCH_3), 3.05 (2H, d, *J* = 8.0, propyl H_2-C2), 2.29 (3H, s, $ArCH_3$);

^{13}C NMR (75 MHz, $CDCl_3$) δ 172.3 (propyl C1), 143.7 (C_q), 140.5 (C_q), 136.1 (C_q), 129.2 (2 × C, ArC), 128.5 (2 × C, ArC), 127.6 (2 × C, ArC), 127.5 (2 × C, ArC), 126.5 (ArC), 51.6 (OCH_3), 46.6 (propyl C3), 40.6 (propyl C2), 21.0 ($ArCH_3$);

IR ν_{max} (neat)/cm^{-1}: 3026, 2951, 2921, 1734 (C=O), 1601, 1494, 1434, 1254;

HRMS (ESI): $C_{17}H_{18}O_2Na$ [M+Na$^+$] calculated 277.1199, found 277.1203.

3-Phenyl-3-(*p*-tolyl)propanoic acid

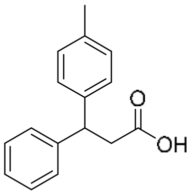

Following general procedure D, using methyl 3-phenyl-3-(*p*-tolyl)propanoate (2.00 g, 7.86 mmol) afforded the *title compound* (1.82 g, 7.57 mmol, 96%) as a colourless solid, which was used without further purification. A small quantity was crystallised from hexane:EtOAc (19:1).

M.p 141-145 °C, colourless microcrystalline solid, hexane-EtOAc;

^{1}H NMR (300 MHz, $CDCl_3$) δ 7.31-7.05 (9H, m, ArH), 4.49 (1H, t, *J* = 7.9, propyl H-C3), 3.07 (2H, d, *J* = 7.9, propyl H_2-C2), 2.30 (3H, s, $ArCH_3$);

^{13}C NMR (75 MHz, $CDCl_3$) δ 177.0 (propyl C1), 143.5 (C_q), 140.2 (C_q), 136.2 (C_q), 129.3 (2 × C, ArC), 128.6 (2 × C, ArC), 127.5 (2 × C, ArC), 127.4 (2 × C, ArC), 126.5 (ArC), 46.3 (propyl C3), 40.3 (propyl C2), 21.0 ($ArCH_3$);

IR ν_{max} (neat)/cm^{-1}: 3272 (O-H), 3094, 3019, 2929, 1636 (C=O), 1567, 1492, 1435;

HRMS (ESI): $C_{16}H_{16}O_2Na$ [M+Na$^+$]: calculated 263.1043, found 263.1045.

N-Methyl-3-phenyl-3-(*p*-tolyl)propanamide

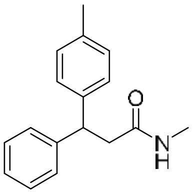

Following general procedure E, using 3-phenyl-3-(*p*-tolyl)propanoic acid (1.35 g, 5.62 mmol). Purification by flash chromatography on silica gel, eluting with 50% EtOAc in pentane afforded the *title compound* (975 mg, 3.85 mmol, 69%) as an amorphous solid.

^{1}H NMR (500 MHz, $CDCl_3$) δ 7.29-7.15 (5H, m, ArH), 7.12 (2H, d, *J* = 8.1, ArH), 7.08 (2H, d, *J* = 8.1, ArH), 5.38 (1H, br. s, NH), 4.54 (1H, t, *J* = 7.8, propyl H-C3), 2.87 (2H, d, *J* = 7.8, propyl H_2-C2), 2.64 (3H, d, *J* = 4.7, NCH_3), 2.29 (3H, s, $ArCH_3$);

^{13}C NMR (125 MHz, $CDCl_3$) δ 171.8 (propyl C1), 144.0 (C_q), 140.7 (C_q), 136.0 (C_q), 129.2 (2 × C, ArC), 128.5 (2 × C, ArC), 127.6 (2 × C, ArC), 127.5 (2 × C, ArC), 126.4 (ArC), 46.9 (propyl C3), 43.3 (propyl C2), 26.2 (NCH_3), 20.9 ($ArCH_3$);

IR ν_{max} (neat)/cm^{-1}: 3272, 3094, 3062, 3020, 2929, 1636 (C=O), 1567, 1512;

HRMS (ESI): $C_{17}H_{20}NO$ [M+H$^+$]: calculated 254.1539, found 254.1534.

N-Methyl-3-phenyl-3-(*p*-tolyl)propan-1-amine

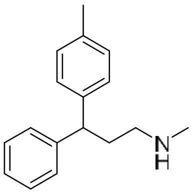

Following general procedure F, using *N*-methyl-3-phenyl-3-(*p*-tolyl)propanamide (750 mg, 2.96 mmol) afforded the *title compound* (684 mg, 2.86 mmol, 97%) as a yellow oil.

^{1}H NMR (500 MHz, $CDCl_3$) δ 7.32-7.22 (4H, m, ArH), 7.19-7.06 (5H, m, ArH), 3.99 (1H, t, *J* = 7.8, propyl H-C3), 2.58-2.52 (2H, m, propyl H_2-C1), 2.40 (3H, s, NCH_3), 2.31 (3H, s, $ArCH_3$), 2.28-2.22 (2H, m, propyl H_2-C2);

^{13}C NMR (125 MHz, $CDCl_3$) δ 145.0 (C_q), 141.8 (C_q), 135.6 (C_q), 129.1 (2 × C, ArC), 128.4 (2 × C, ArC), 127.7 (2 × C, ArC), 127.6 (2 × C, ArC), 126.1 (ArC), 50.5 (propyl C1), 48.7 (propyl C3), 36.4 (propyl C2), 35.7 (NCH_3), 20.9 ($ArCH_3$);

IR ν_{max} (neat)/cm^{-1}: 3024, 2925, 2862, 2792, 1600, 1512, 1493, 1450;

HRMS (ESI): $C_{17}H_{22}N$ [M+H$^+$]: calculated 240.1747, found 240.1743.

Route C

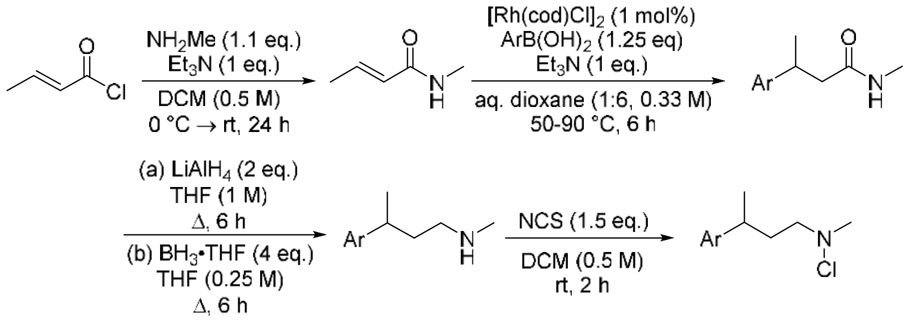

(*E*)-*N*-Methylbut-2-enamide

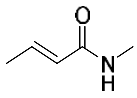

Following a procedure by Greaney *et* al.,[8] to a stirred solution of methylamine (5.50 mL of a 40% w/w in H_2O, 1.1 eq.) and Et_3N (7.67 mL, 50 mmol, 1 eq.) in DCM (100 mL) at 0 °C, was added crotonyl chloride (4.79 mL, 50 mmol, 1 eq.) dropwise. The reaction mixture was stirred at 0 °C for 30 min, then warmed to rt and stirred for 24 h. The reaction was quenched with sat. aqueous $NaHCO_3$ (50 mL) and the phases separated. The organic phase was dried over Na_2SO_4 and concentrated *in vacuo*. Purification by flash chromatography on silica gel, eluting with EtOAc afforded the title compound (3.46 g, 34.9 mmol, 70%) as a colourless solid. A small quantity was crystallised from hexane.

M.p 72-75 °C, colourless microcrystalline solid, hexane;

^{1}H NMR (500 MHz, $CDCl_3$) δ 6.83 (1H, dq, *J* = 15.1, 6.9, butenyl H-C3), 5.78 (1H, dq, *J* = 15.1, 1.6, butenyl H-C2), 5.41 (1H, s, NH), 2.86 (3H, d, *J* = 3.9, NCH_3), 1.84 (3H, dd, *J* = 6.9, 1.6, butenyl H_3-C4);

^{13}C NMR (100 MHz, $CDCl_3$) δ 166.9 (butenyl C1), 139.5 (butenyl C3), 125.1 (butenyl C2), 26.3 (NCH_3), 17.7 (butenyl C4);

IR ν_{max} (neat)/cm^{-1}: 3269 (N-H), 3092, 2961, 2943, 2916, 1666 (C=O), 1625, 1563;

HRMS (ESI): $C_5H_{10}ON$ [M+H$^+$]: calculated 100.0757, found 100.0754.

N-Methyl-3-phenylbutanamide

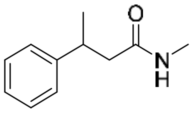

Following general procedure C, using *N*-methylcrotonamide (500 mg, 5.04 mmol) and $PhB(OH)_2$ (768 mg, 6.30 mmol). Purification by flash chromatography on silica gel, eluting with a gradient

of 25-50% EtOAc in hexane afforded the title compound (461 mg, 2.60 mmol, 52%) as a colourless solid. A small quantity was crystallised from hexane-EtOAc (9:1).

M.p 60-62 °C, colourless crystalline solid, hexane-EtOAc;

^{1}H NMR (300 MHz, $CDCl_3$) δ 7.36-7.27 (2H, m, ArH), 7.24-7.16 (3H, m, ArH), 5.22 (1H, br. s, NH), 3.37-3.24 (1H, m, butyl H-C3), 2.71 (3H, d, *J* = 4.8, NCH_3), 2.49-2.32 (2H, m, butyl H_2-C2), 1.31 (3H, d, *J* = 7.0, butyl H_3-C4);

^{13}C NMR (75 MHz, $CDCl_3$) δ 172.3 (butyl C1), 146.0 (C_q), 128.6 (2 × C, ArC), 126.7 (2 × C, ArC), 126.4 (ArC), 45.8 (butyl C2), 36.9 (butyl C3), 26.2 (NCH_3), 21.6 (butyl C4);

IR ν_{max} (neat)/cm^{-1}: 3298 (N-H), 3029, 2961, 2919, 1639 (C=O), 1564, 1494, 1298;

HRMS (ESI): $C_{11}H_{16}NO$ [M+H$^+$]: calculated 178.1226, found 178.1225.

N-Methyl-3-phenylbutan-1-amine

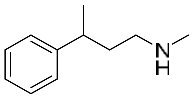

Following general procedure F, using *N*-methyl-3-phenylbutanamide (400 mg, 2.26 mmol) afforded the *title compound* (275 mg, 1.68 mmol, 74%) as a yellow oil.

^{1}H NMR (300 MHz, $CDCl_3$) δ 7.34-7.26 (2H, m, ArH), 7.22-7.14 (3H, m, ArH), 2.85-2.70 (1H, m, butyl H-C3), 2.57-2.42 (2H, m, butyl H_2-C1), 2.37 (3H, s, NCH_3), 1.83-1.72 (2H, m, butyl H_2-C2), 1.26 (3H, d, *J* = 7.0, butyl H_3-C4);

^{13}C NMR (75 MHz, $CDCl_3$) δ 147.2 (C_q), 128.4 (2 × C, ArC), 126.9 (2 × C, ArC), 126.0 (ArC), 50.4 (butyl C1), 38.3 (butyl C2), 38.0 (NCH_3), 36.5 (butyl C3), 22.5 (butyl C4);

IR ν_{max} (neat)/cm^{-1}: 3026, 2958 (N-H), 2925, 1603, 1543, 1493, 1473, 1376;

HRMS (ESI): $C_{11}H_{18}N$ [M+H$^+$]: calculated 164.1434, found 164.1432.

N-Methyl-3-(*o*-tolyl)butanamide

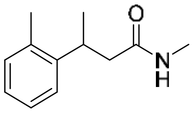

Following general procedure C, using *N*-methylcrotonamide (500 mg, 5.04 mmol) and *o*-MePhB(OH)$_2$ (857 mg, 6.30 mmol). Purification by flash chromatography on silica gel, eluting with a gradient of 25-50% EtOAc in hexane afforded the *title compound* (422 mg, 2.21 mmol, 44%) as a yellow gum.

^{1}H NMR (500 MHz, $CDCl_3$) δ 7.20-7.06 (4H, m, ArH), 5.35 (1H, s, NH), 3.62-3.53 (1H, m, butyl H-C3), 2.72 (1H, d, *J* = 4.8, NCH_3), 2.45 (1H, dd, *J* = 14.0, 6.6, butyl H_a-C2), 2.39-2.29 (4H, m, includes 3H, s, $ArCH_3$, and 1H, m, butyl H_b-C2), 1.26 (3H, d, *J* = 6.9, butyl H_3-C4);

^{13}C NMR (125 MHz, $CDCl_3$) δ 172.4 (butyl C1), 144.2 (C_q), 135.5 (C_q), 130.5 (ArC), 126.2 (ArC), 126.0 (ArC), 124.9 (ArC), 44.8 (butyl C2), 31.8 (butyl C3), 26.2 (NCH_3), 21.2 (butyl C4), 19.4 ($ArCH_3$);

IR ν_{max} (neat)/cm^{-1}: 3303 (N-H), 3062, 2965, 2877, 1638 (C=O), 1559, 1458, 1363;

HRMS (ESI): $C_{12}H_{17}NONa$ [M+Na$^+$]: calculated 214.1202, found 214.1207.

N-Methyl-3-(*o*-tolyl)butan-1-amine

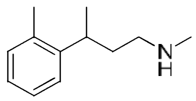

Following general procedure F, using *N*-methyl-3-(*o*-tolyl)butanamide (350 mg, 1.83 mmol). Purification by SCX cartridge the *title compound* (270 mg, 1.52 mmol, 83%) as a yellow oil.

^{1}HNMR (300 MHz, $CDCl_3$) δ 7.24-7.04 (4H, m, ArH), 3.13-2.99 (1H, m, butyl H-C3), 2.59-2.43 (2H, m, butyl H_2-C1), 2.39 (3H, s, NCH_3), 2.33 (3H, s, $ArCH_3$), 1.90-1.68 (2H, m, butyl H_2-C2), 1.22 (3H, d, *J* = 6.9, butyl H_3-C4);

^{13}C NMR (75 MHz, $CDCl_3$) δ 145.4 (C_q), 135.2 (C_q), 130.2 (ArC), 126.2 (ArC), 125.6 (ArC), 125.2 (ArC), 50.4 (butyl C1), 37.8 (butyl C2), 36.5 (NCH_3), 32.5 (butyl C3), 21.8 (butyl C4), 19.6 ($ArCH_3$);

IR ν_{max} (neat)/cm^{-1}: 3018, 2959 (N-H), 2927, 2788, 1489, 1456, 1375, 1123;

HRMS (ESI): $C_{12}H_{20}N$ [M+H$^+$]: calculated 178.1590, found 178.1587.

N-Methyl-3-(*m*-tolyl)butanamide

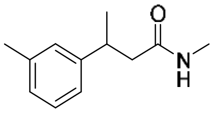

Following general procedure C, using *N*-methylcrotonamide (500 mg, 5.04 mmol) and *m*-MePhB$(OH)_2$ (1.03 g, 7.56 mmol). Purification by flash chromatography on silica gel, eluting with 50% EtOAc in pentane afforded the *title compound* (598 mg, 3.13 mmol, 62%) as a yellow gum.

^{1}H NMR (500 MHz, $CDCl_3$) δ 7.21 (1H, t, *J* = 7.5, ArH), 7.08-7.01 (3H, m, ArH), 5.36 (1H, s, NH), 3.28 (1H, h, *J* = 7.1, butyl H-C3), 2.74 (3H, d, *J* = 4.8, NCH_3), 2.46 (1H, dd, *J* = 14.0, 7.2, butyl H_a-C2), 2.39 (1H, dd, *J* = 14.0, 7.7, butyl H_b-C2), 2.36 (3H, s, $ArCH_3$), 1.32 (3H, d, *J* = 7.0, butyl H_3-C4);

^{13}C NMR (125 MHz, $CDCl_3$) δ 172.3 (butyl C1), 146.1 (C_q), 138.1 (C_q), 128.5 (ArC), 127.6 (ArC), 127.1 (ArC), 123.7 (ArC), 45.8 (butyl C2), 36.9 (butyl C3), 26.1 (NCH_3), 21.6 (butyl C4), 21.4 ($ArCH_3$);

IR ν_{max} (neat)/cm^{-1}: 3302 (N-H), 2961, 2905, 1637 (C=O), 1608, 1586, 1554, 1489;

HRMS (ESI): $C_{12}H_{18}NO$ [M+H$^+$]: calculated 192.1383, found 192.1381.

N-Methyl-3-(*m*-tolyl)butan-1-amine

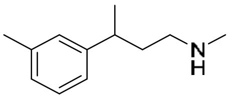

Following general procedure F, using *N*-methyl-3-(*m*-tolyl)butanamide (450 mg, 2.35 mmol). Purification by SCX cartridge the *title compound* (399 mg, 2.25 mmol, 96%) as a yellow oil.

^{1}H NMR (500 MHz, $CDCl_3$) δ 7.21-7.15 (1H, m, ArH), 7.04-6.96 (3H, m, ArH), 2.77-2.69 (1H, m, butyl H-C3), 2.55-2.42 (2H, m, butyl H_2-C1), 2.38 (3H, s, NCH_3), 2.34 (3H, s, $ArCH_3$), 1.83-1.72 (2H, m, butyl H_2-C2), 1.25 (3H, d, *J* = 7.0, butyl H_3-C4);

^{13}C NMR (125 MHz, $CDCl_3$) δ 147.1 (C_q), 137.8 (C_q), 128.2 (ArC), 127.6 (ArC), 126.7 (ArC), 123.9 (ArC), 50.3 (butyl C1), 38.2 (butyl C2), 37.9 (butyl C3), 36.3 (NCH_3), 22.5 (butyl C4), 21.4 ($ArCH_3$);

IR ν_{max} (neat)/cm^{-1}: 2958, 2923, 2869, 2792, 1606, 1454, 1375, 1306;

HRMS (ESI): $C_{12}H_{20}N$ [M+H$^+$]: calculated 178.1590, found 178.1588.

N-Methyl-3-(*p*-tolyl)butanamide

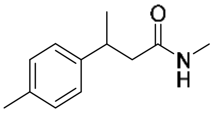

Following general procedure C, using *N*-methylcrotonamide (500 mg, 5.04 mmol) and *p*-MePhB(OH)$_2$ (1.03 g, 7.56 mmol). Purification by flash chromatography on silica gel, eluting with 50% EtOAc in pentane afforded the *title compound* (564 mg, 2.95 mmol, 59%) as a colourless solid. A small quantity was crystallised from hexane.

M.p 86-87 °C, colourless needles, hexane;

^{1}H NMR (500 MHz, $CDCl_3$) δ 7.11 (4H, s, ArH), 5.19 (1H, s, NH), 3.26 (1H, h, *J* = 7.1, butyl H-C3), 2.71 (3H, d, *J* = 4.9, NCH_3), 2.42 (1H, dd, *J* = 14.0, 7.3, butyl H_a-C2), 2.36 (1H, dd, *J* = 14.0, 7.5, butyl H_b-C2), 2.32 (3H, s, $ArCH_3$), 1.29 (3H, d, *J* = 7.0, butyl H_3-C4);

^{13}C NMR (125 MHz, $CDCl_3$) δ 172.4 (butyl C1), 142.9 (C_q), 135.9 (C_q), 129.2 (2 × C, ArC), 126.6 (2 × C, ArC), 45.9 (butyl C2), 36.5 (butyl C3), 26.2 (NCH_3), 21.7 ($ArCH_3$), 21.0 (butyl C4);

IR ν_{max} (neat)/cm^{-1}: 3306 (N-H), 2972, 2960, 2916, 2875, 1637 (C=O), 1550, 1514;

HRMS (ESI): $C_{12}H_{17}NaNO$ [M+Na$^+$]: calculated 214.1202, found 214.1210.

N-Methyl-3-(*p*-tolyl)butan-1-amine

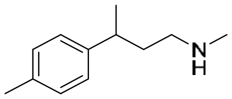

Following general procedure F, using *N*-methyl-3-(*p*-tolyl)butanamide (450 mg, 2.35 mmol). Purification by SCX cartridge the *title compound* (384 mg, 2.25 mmol, 96%) as a yellow oil.

^{1}H NMR (500 MHz, $CDCl_3$) δ 7.15-7.04 (4H, m, ArH), 2.78-2.70 (1H, m, butyl H-C3), 2.56-2.42 (2H, m, butyl H_2-C1), 2.38 (3H, s, NCH_3), 2.32 (3H, s, $ArCH_3$), 1.82-1.70 (2H, m, butyl H_2-C2), 1.24 (3H, d, *J* = 7.0, butyl H_3-C4);

^{13}C NMR (125 MHz, $CDCl_3$) δ 144.2 (C_q), 135.4 (C_q), 129.0 (2 × C, ArC), 126.7 (2 × C, ArC), 50.4 (butyl C1), 38.3 (butyl C2), 37.5 (butyl C3), 36.4 (NCH_3), 22.6 (butyl C4), 20.9 ($ArCH_3$);

IR ν_{max} (neat)/cm^{-1}: 2957, 2923, 2869, 2790, 1514, 1451, 1373, 1114;

HRMS (ESI): $C_{12}H_{20}N$ [M+H$^+$]: calculated 178.1590, found 178.1592.

3-(4-Bromophenyl)-*N*-methylbutanamide

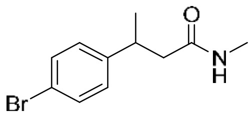

Following general procedure C, using *N*-methylcrotonamide (500 mg, 5.04 mmol) and *p*-BrPhB(OH)$_2$ (1.52 g, 7.56 mmol). Purification by automated flash chromatography on silica gel, eluting with a gradient 25-100% EtOAc in hexane afforded the *title compound* (536 mg, 2.09 mmol, 42%) as a colourless solid. A small quantity was crystallised from hexane-EtOAc (9:1).

M.p 108-111 °C, colourless microcrystalline solid, hexane-EtOAc;

^{1}H NMR (500 MHz, $CDCl_3$) δ 7.41 (2H, d, *J* = 8.4, ArH), 7.09 (2H, d, *J* = 8.4, ArH), 5.26 (1H, s, NH), 3.34-3.23 (1H, m, butyl H-C3), 2.72 (3H, d, *J* = 4.8, NCH_3), 2.36 (2H, d, *J* = 7.4, butyl H_2-C2), 1.28 (3H, d, *J* = 7.0, butyl H_3-C4);

^{13}C NMR (125 MHz, $CDCl_3$) δ 171.8 (butyl C1), 145.0 (C_q), 131.6 (2 × C, ArC), 128.5 (2 × C, ArC), 120.0 (C_q), 45.5 (butyl C2), 36.3 (butyl C3), 26.2 (NCH_3), 21.4 (butyl C4);

IR ν_{max} (neat)/cm^{-1}: 3299 (N-H), 2962, 2931, 2874, 1634 (C=O), 1556, 1487, 1402;

HRMS (ESI): $C_{11}H_{15}{}^{79}BrNO$ [M+H$^+$]: calculated 256.0332, found 256.0330.

3-(4-Bromophenyl)-*N*-methylbutan-1-amine

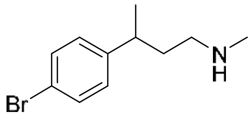

Following general procedure G, using 3-(4-bromophenyl)-*N*-methylbutanamide (400 mg, 1.56 mmol). Purification by SCX cartridge afforded the *title compound* (281 mg, 1.16 mmol, 74%) as a colourless oil.

^{1}H NMR (500 MHz, $CDCl_3$) δ 7.40 (2H, d, *J* = 8.4, ArH), 7.06 (2H, d, *J* = 8.4, ArH), 2.79-2.70 (1H, m, butyl H-C3), 2.51-2.40 (2H, m, butyl H_2-C1), 2.37 (3H, s, NCH_3), 1.77-1.69 (2H, m, butyl H_2-C2), 1.23 (3H, d, *J* = 7.0, butyl H_3-C4);

^{13}C NMR (125 MHz, $CDCl_3$) δ 146.2 (C_q), 131.4 (2 × C, ArC), 128.7 (2 × C, ArC), 119.5 (C_q), 50.2 (butyl C1), 38.2 (butyl C2), 37.4 (butyl C3), 36.5 (NCH_3), 22.4 (butyl C4);

IR ν_{max} (neat)/cm^{-1}: 2959, 2925, 2871, 2792, 1621, 1548, 1486, 1454;

HRMS (ESI): $C_{11}H_{17}{}^{79}BrN$ [M+H$^+$]: calculated 242.0539, found 242.0537.

(*S*,*E*)-2-Methyl-*N*-(3-phenylpropylidene)propane-2-sulfinamide

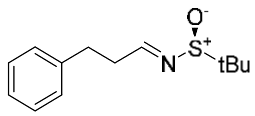

To an oven-dried flask flushed with N_2 was added anhydrous $CuSO_4$ (7.98 g, 50.0 mmol, 2.0 eq.) and (*S*)-(−)-*tert*-butylsulfinamide (3.03 g, 25.0 mmol, 1.0 eq.). A solution of hydrocinnamaldehyde (3.62 mL, 27.5 mmol, 1.1 eq.) in DCM (30 mL) was added and the reaction mixture was stirred at rt for 16 h. The reaction mixture was filtered through a pad of Celite, the Celite was washed with DCM (400 mL) and the filtrate was collected and concentrated *in vacuo* to afford the title compound (5.93 g, 25.0 mmol, quant.) as a yellow oil.

^{1}H NMR (500 MHz, $CDCl_3$) δ 8.12 (1H, t, *J* = 4.3, propylidyl H-C1), 7.31-7.25 (2H, m, ArH), 7.23-7.16 (3H, m, ArH), 3.01-2.94 (2H, m, propylidyl H_2-C3), 2.90-2.84 (2H, m, propylidyl H_2-C2), 1.13 (9H, s, $C(CH_3)_3$);

^{13}C NMR (125 MHz, $CDCl_3$) δ 168.6 (propylidyl C1), 140.5 (C_q), 128.7 (2 × C, ArC), 128.5 (2 × C, ArC), 126.4 (ArC), 56.7 (C_q), 37.6 (propylidyl C3), 31.5 (propylidyl C2), 22.4 (3 × C, $(CH_3)_3$);

IR ν_{max} (neat)/cm^{-1}: 3027, 2959, 2926, 2867, 1622 (N=C), 1497, 1455, 1391;

HRMS (ESI): $C_{13}H_{20}NOS$ [M+H$^+$]: calculated 238.1260, found 238.1258.

General Procedure H: Grignard addition to sulfinimine 13

To a stirred solution of sulfinimine **13** (1.0 eq.) in THF (0.5 M) at –78 °C was added a solution of Grignard reagent (1.5 eq.) dropwise. The reaction mixture was stirred at –78 °C for 2 h after which it was warmed to rt and quenched with sat. aqueous $NaHCO_3$ solution (1 vol). The phases were separated and the aqueous phase was extracted with EtOAc (× 3), then the combined organic extracts were dried over Na_2SO_4 and concentrated *in vacuo*. Purification afforded the desired product.

General Procedure I: *N*-Alkylation and deprotection

To a stirred solution of sulfinamide (1.0 eq.) in THF (0.25 M) at 0 °C was added LiHMDS (1.0 M solution in THF, 1.0 eq.) dropwise. After 1 h MeI (2.0 eq.) was added portionwise and the reaction mixture was warmed to rt and stirred for 2 h. The reaction mixture was quenched with H_2O (1 vol) and the phases were separated. The aqueous phase was extracted with EtOAc (× 3), then the combined organic extracts were washed with sat. brine solution (1 vol), dried over Na_2SO_4 and concentrated *in vacuo*. The crude product was dissolved in 3 N HCl in MeOH (0.25 M) and stirred for 2 h, after which the reaction mixture was concentrated then taken up in EtOAc (0.5 M). This was extracted with 2 M aqueous HCl (1 vol) then the aqueous phase was basified with 2 M aqueous NaOH (30 mL) and extracted with EtOAc (× 3). The combined organic extracts were dried over Na_2SO_4 and concentrated *in vacuo*. Purification afforded the desired product.

2-Methyl-*N*-(5-phenylpent-1-en-3-yl)propane-2-sulfinamide

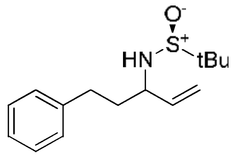

Following general procedure H, using sulfinimine **13** (2.50 g, 10.5 mmol) and vinylmagnesium bromide (15.8 mL of a 1.0 M solution in THF). Purification by flash chromatography on silica gel, eluting with 20% EtOAc in hexane afforded an inseparable mixture of the diastereoisomeric *title compound* (2.38 g, 8.97 mmol, 85%, 2:1) as a pale yellow oil.

^{1}H NMR (500 MHz, $CDCl_3$) δ 7.38-7.31 (2H, m, includes 1.34H, m, ArH, *major*, and 0.66H, m, ArH *minor*), 7.28-7.20 (3H, m, includes 2H, m, ArH, *major*, and 1H, m, ArH, *minor*), 5.95 (0.33H, ddd, *J* = 17.3, 10.3, 7.0, pentenyl H-C2, *minor*), 5.78 (0.67H, ddd, *J* = 17.5, 10.2, 7.6, pentenyl H-C2, *major*), 5.39-5.24 (2H, m, includes 1.34H, m, pentenyl H_2-C1, *major*, and 0.66H, m, pentenyl H_2-C1, *minor*), 3.95-3.82 (1H, m, includes 0.67H, m, pentenyl H-C3, *major*, and 0.33H, m, pentenyl H-C3, *minor*), 3.27-3.18 (1H, m, includes 0.67H, m, NH, *major*, and 0.33H, m, NH, *minor*), 2.83-2.66 (2H, m, includes 1.34H, m, pentenyl H_2-C5, *major*, and 0.66H, m, pentenyl H_2-C5, *minor*), 2.10-1.86 (2H, m, includes 1.34H, m, pentenyl H_2-C4, *major*, and 0.66H, m, pentenyl H_2-C4, *minor*), 1.29 (2.97H, s, $C(CH_3)_3$, *minor*), 1.25 (6.03H, s, $C(CH_3)_3$, *major*);

^{13}C NMR (125 MHz, $CDCl_3$) δ 141.5 (C_q), 141.2 (C_q), 139.5 (CH), 138.8 (CH), 128.4 (ArC), 128.4 (ArC), 128.4 (ArC), 128.3 (ArC), 126.0 (ArC), 125.9 (ArC), 117.4 (CH_2), 116.8 (CH_2), 58.2 (CH), 58.0 (CH), 55.8 (C_q), 55.4 (C_q), 37.9 (CH_2), 37.0 (CH_2), 31.8 (CH_2), 31.7 (CH_2), 22.6 (CH_3), 22.5 (CH_3);

IR ν_{max} (neat)/cm^{-1}: 3212 (N-H), 3026, 2979, 2949, 2864, 1642, 1603, 1496;

HRMS (ESI): $C_{15}H_{24}NOS$ [M+H$^+$]: calculated 266.1573, found 266.1574.

N-(1,3-Diphenylpropyl)-2-methylpropane-2-sulfinamide

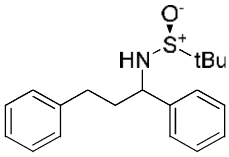

Following general procedure H, using sulfinimine **13** (1.00 g, 4.21 mmol) and phenylmagnesium bromide (6.32 mL of of 1.0 M solution in THF). Purification by flash chromatography on silica gel, eluting with 20% EtOAc in hexane afforded an inseparable mixture of the diastereoisomeric title compounds (729 mg, 2.31 mmol, 55%, 3:1) as a colourless gum.

^{1}H NMR (500 MHz, $CDCl_3$) δ 7.40-7.24 (7H, m, includes 5.25H, m, ArH, *major*, and 1.75H, m, ArH, *minor*), 7.19-7.09 (3H, m, includes 2.25H, m, ArH, *major*, and 0.75H, m, ArH, *minor*), 4.45-4.37 (1H, m, includes 0.75H, m, propyl H-C1, *major*, and 0.25H, m, propyl H-C1, *minor*), 3.45-3.36 (1H, m, includes 0.75H, m, NH, *major*, and 0.25H, m, NH, *minor*), 2.62-2.51 (1H, m, includes 0.75H, m, propyl H_a-C2, *major*, and 0.25H, m, propyl H_a-C2, *minor*), 2.51-2.42 (1H, m includes 0.75H, m, propyl H_a-C3, *major*, and 0.25H, m, propyl H_a-C3, *minor*), 2.37 (0.75H, ddt, J = 13.4, 10.9, 5.6, propyl H_b-C2, *major*), 2.21-2.12 (0.25H, m, H_b-C2, *minor*), 2.12-2.02 (1H, m includes 0.75H, m, propyl H_b-C3, *major*, and 0.25H, m, propyl H_b-C3, *minor*), 1.23 (6.75H, s, $C(CH_3)_3$, *major*), 1.14 (2.25H, s, $C(CH_3)_3$, *minor*);

^{13}C NMR (125 MHz, $CDCl_3$) δ 142.1 (C_q), 141.7 (C_q), 141.4 (C_q), 141.1 (C_q), 128.8 (ArC), 128.5 (ArC), 128.5 (ArC), 128.4 (ArC), 128.4 (ArC), 128.3 (ArC), 128.0 (ArC), 127.7 (ArC), 127.7 (ArC), 127.3 (ArC), 126.1 (ArC), 125.9 (ArC), 59.0 (CH), 58.7 (CH), 55.7 (C_q), 55.5 (C_q), 40.2 (CH_2), 38.2 (CH_2), 32.2 (CH_2), 31.9 (CH_2), 22.6 (CH_3), 22.5 (CH_3);

IR ν_{max} (neat)/cm^{-1}: 3240 (N-H), 3215, 3087, 3027, 2954, 1603, 1495, 1452;

HRMS (ESI): $C_{19}H_{26}NOS$ [$M+H^+$]: calculated 316.1730, found 316.1736.

N-Methyl-1,3-diphenylpropan-1-amine

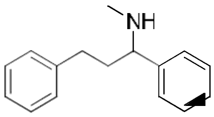

Following general procedure I, using *N*-(1,3-diphenylpropyl)-2-methylpropane-2-sulfinamide (700 mg, 2.22 mmol). Purification by SCX cartridge afforded the title compound (413 mg, 1.83 mmol, 82%) as a yellow oil.

^{1}H NMR (500 MHz, $CDCl_3$) δ 7.39-7.32 (2H, m, ArH), 7.32-7.24 (5H, m, ArH), 7.20-7.10 (3H, m, ArH), 3.49 (1H, dd, *J* = 7.8, 6.0, propyl H-C1), 2.60-2.48 (2H, m, propyl H_2-C3), 2.27 (3H, s, NCH_3), 2.14-2.04 (1H, m, propyl H_a-C2), 2.03-1.92 (1H, m, propyl H_b-C2);

^{13}C NMR (125 MHz, $CDCl_3$) δ 143.4 (C_q), 142.0 (ArC), 128.4 (2 × C, ArC), 128.3 (2 × C, ArC), 128.3 (2 × C, ArC), 127.4 (2 × C, ArC), 127.13 (ArC), 125.7 (C_q), 64.9 (propyl C1), 39.2 (propyl C2), 34.3 (NCH_3), 32.5 (propyl C3);

IR ν_{max} (neat)/cm^{-1}: 3061, 2935, 2850, 2789, 1602, 1493, 1475, 1452;

HRMS (ESI): $C_{16}H_{20}N$ [M+H$^+$]: calculated 226.1590, found 226.1592.

(*R*)-2-Methyl-*N*-(4-phenylbutan-2-yl)propane-2-sulfinamide

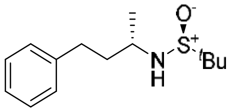

Following general procedure H, using sulfinimine **13** (1.00 g, 4.21 mmol) and methylmagnesium bromide (2.11 mL of 3.0 M solution in Et_2O). Purification by flash chromatography on silica gel, eluting with a gradient of 20-40% EtOAc in pentane afforded an inseparable mixture of the diastereoisomeric title compounds (726 mg, 2.87 mmol, 68%, *dr* 93:7) as a colourless solid. Ratio determined by comparison of *tert*-butyl signals in the ^{1}H NMR spectrum; only signals for the major isomer reported.

^{1}H NMR (500 MHz, $CDCl_3$) δ 7.30-7.25 (2H, m, ArH), 7.21-7.14 (3H, m, ArH), 3.39 (1H, dt, *J* = 13.2, 6.7, butyl H-C2), 2.92 (1H, d, *J* = 7.0, NH), 2.75-2.60 (2H, m, butyl H_2-C4), 1.90 -1.70 (2H, m, butyl H_2-C3), 1.31 (3H, d, *J* = 6.5, butyl H_3-C1), 1.22 (9H, s, $C(CH_3)_3$);

^{13}C NMR (125 MHz, $CDCl_3$) δ 141.8 (C_q), 128.4 (2 × C, ArC), 128.3 (2 × C, ArC), 125.9 (ArC), 55.7 (C_q), 52.1 (butyl C2), 39.9 (butyl C3), 32.1 (butyl C4), 23.2 (butyl C1), 22.6 (3 × C, $(CH_3)_3$);

IR ν_{max} (neat)/cm^{-1}: 3255 (N-H), 3062, 3025, 2965, 2923, 2862, 1493, 1454;

HRMS (ESI): $C_{14}H_{24}NOS$ [M+H$^+$]: calculated 254.1573, found 254.1574.

N-Methyl-4-phenylbutan-2-amine

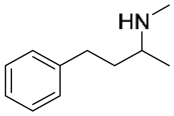

Following general procedure I, using (*R*)-2-methyl-*N*-(4-phenylbutan-2-yl)propane-2-sulfinamide (700 mg, 2.75 mmol) afforded the title compound (382 mg, 2.34 mmol, 85%) as a pale yellow oil.

^{1}H NMR (500 MHz, $CDCl_3$) δ 7.32-7.24 (2H, m, ArH), 7.22-7.14 (3H, m, ArH), 2.72-2.50 (3H, m, includes 2H, m, butyl H_2-C4, and 1H, m, butyl H-C2), 2.40 (3H, s, NCH_3), 1.84-1.72 (1H, m, butyl H_a-C3), 1.68-1.54 (1H, m, butyl H_b-C3), 1.09 (3H, d, *J* = 6.2, butyl H_3-C4);

^{13}C NMR (125 MHz, $CDCl_3$) δ 142.4 (C_q), 128.3 (2 × C, ArC), 128.3 (2 × C, ArC), 125.7 (ArC), 54.4 (butyl C2), 38.5 (butyl C3), 33.8 (NCH_3), 32.3 (butyl C4), 19.9 (butyl C1);

IR ν_{max} (neat)/cm^{-1}: 3304 (N-H), 3062, 2930, 2857, 2792, 1541, 1495, 1453;

HRMS (ESI): $C_{11}H_{18}N$ [M+H$^+$]: calculated 164.1434, found 164.1432.

Route E

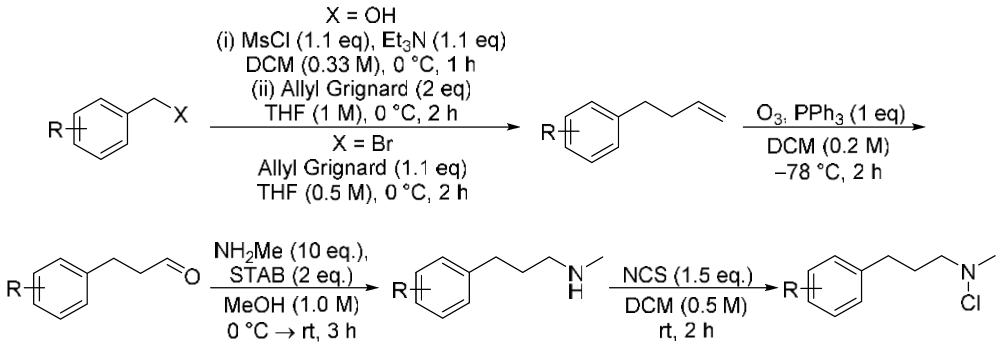

General Procedure J: Formation of benzyl mesylate and Grignard addition

To a stirred solution of benzyl alcohol (1.0 eq.) in DCM (0.33 M) at 0 °C was added Et_3N (1.1 eq.) and then MsCl (1.1 eq.) dropwise. After 2 h the reaction mixture was warmed to rt then the reaction was quenched with sat. aqueous $NaHCO_3$ and the phases were separated. The aqueous phase was extracted with DCM (× 2) and the combined organic extracts were washed with brine (1 vol), dried over Na_2SO_4 and then concentrated *in vacuo*. The crude residue was then flushed with N_2 and dissolved in THF (0.5 M). The reaction mixture was cooled to 0 °C and a solution of Grignard reagent (2.0 eq.) was added dropwise. After 2 h the reaction mixture was warmed to rt and the reaction was quenched with sat. aqueous NH_4Cl (2 vol), then the aqueous phase was extracted with EtOAc (× 3). The combined organic extracts were washed with brine (1 vol), dried over Na_2SO_4 and concentrated *in vacuo*. Purification afforded the desired product.

General Procedure K: Grignard addition to benzyl bromide

To a stirred solution of benzyl bromide (1.0 eq.) in THF (0.5 M) at 0 °C was added a solution of Grignard reagent (1.1 eq.) dropwise. The reaction mixture was stirred at 0 °C for 30 min then warmed to rt and stirred for 1.5 h after which the reaction was quenched with sat. aqueous NH_4Cl (2 vol). The aqueous phase was extracted with EtOAc (× 3) and the combined organic extracts were washed with brine (1 vol), dried over Na_2SO_4 and concentrated *in vacuo*. Purification afforded the desired product.

General Procedure L: Ozonolysis reaction

A stream of O_2 gas was bubbled through a solution of alkene (1.0 eq.) in DCM (0.2 M) at −78 °C for 5 min. After, O_3 gas was bubbled through the solution until the solution turned blue. O_2 was then bubbled through the reaction mixture until the solution turned colourless and then PPh_3 (1.05 eq.) was added and the reaction mixture was stirred until no peroxides remained (starch/I_2 test). The reaction mixture was warmed to rt and concentrated *in vacuo*. Purification afforded the desired products.

1-(But-3-en-1-yl)-2-phenylbenzene

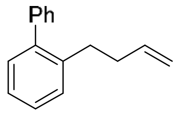

Following general procedure J, using (2-biphenyl)methanol (1.00 g, 5.43 mmol) and allymagnesium chloride (5.43 mL of a 2.0 M solution in THF). Purification by flash chromatography on silica gel, eluting with 20% DCM in hexane afforded the title compound (956 mg, 4.59 mmol, 85%) as a colourless oil. The data is in accordance with the literature.[9]

^{1}H NMR (500 MHz, $CDCl_3$) 7.48-7.22 (9H, m, ArH), 5.75 (1H, ddt, *J* = 16.9, 10.2, 6.6, butenyl H-C3), 4.98-4.88 (2H, m, butenyl H_2-C4), 2.77-2.68 (2H, m, butenyl H_2-C1), 2.29-2.21 (2H, m, butenyl H_2-C2);

^{13}C NMR (125 MHz, $CDCl_3$) δ 142.1 (C_q), 142.0 (C_q), 139.4 (C_q), 138.3 (butenyl C3), 130.2 (ArC), 129.4 (2 × C, ArC), 128.2 (2 × C, ArC), 127.5 (ArC), 126.9 (ArC), 125.9 (ArC), 114.8 (butenyl C4), 35.3 (butenyl C2), 32.7 (butenyl C1);

IR ν_{max} (neat)/cm^{-1}: 3060, 3021, 2926, 1640, 1598, 1500, 1478, 1450;

3-(2-Phenylphenyl)propanal

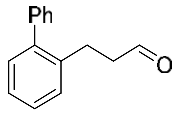

Following general procedure L, using 1-(but-3-en-1-yl)-2-phenylbenzene (750 mg, 3.60 mmol). Purification by flash chromatography on silica gel, eluting with 10% Et_2O in hexane afforded the *title compound* (596 mg, 2.83 mmol, 79%) as a colourless oil.

^{1}H NMR (400 MHz, $CDCl_3$) δ 9.63 (1H, t, *J* = 1.4, propyl H-C1), 7.49-7.17 (9H, m, ArH), 2.96 (2H, t, *J* = 7.8, propyl H_2-C3), 2.55 (2H, td, *J* = 7.8, 1.4, propyl H_2-C2);

^{13}C NMR (100 MHz, $CDCl_3$) δ 201.7 (propyl C1), 142.1 (C_q), 141.5 (C_q), 137.8 (C_q), 130.5 (ArC), 129.24 (ArC), 129.17 (2 × C, ArC), 128.4 (2 × C, ArC), 127.8 (ArC), 127.2 (ArC), 126.5 (ArC), 45.0 (propyl C2), 25.8 (propyl C3);

IR ν_{max} (neat)/cm^{-1}: 3059, 3022, 2891, 2822, 2722, 1719 (C=O), 1598, 1500;

N-Methyl[3-(2-phenylphenyl)propyl]amine

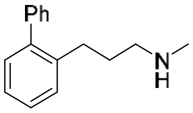

Following general procedure A, using 3-(2-phenylphenyl)propanal (450 mg, 2.14 mmol) and $MeNH_2$ (3.00 mL of an 8.0 M solution in EtOH) afforded the *title compound* (480 mg, 2.13 mmol, 99%) as a clear yellow oil.

^{1}H NMR (400 MHz, $CDCl_3$) δ 7.47-7.18 (9H, m, ArH), 2.68-2.58 (2H, m, propyl H_2-C1), 2.51-2.42 (2H, m, propyl H_2-C1), 2.33 (3H, s, NCH_3), 1.71-1.60 (2H, m, propyl H_2-C2), 1.35 (1H, br. s, NH);

^{13}C NMR (100 MHz, $CDCl_3$) δ 142.02 (C_q), 142.00 (C_q), 139.8 (C_q), 130.2 (ArC), 129.4 (2 × C, ArC), 128.1 (2 × C, ArC), 127.6 (ArC), 126.9 (ArC), 125.9 (ArC), 51.8 (propyl C1), 36.4 (NCH_3), 31.5 (propyl C3), 30.9 (propyl C2), one ^{13}C signal missing;

IR ν_{max} (neat)/cm^{-1}: 3057, 3021, 2930, 2861, 2789, 1478, 1437, 1374;

HRMS (ESI): $C_{16}H_{20}N$ [M+H$^+$]: calculated 226.1590, found 226.1587.

1-(But-3-en-1-yl)-4-phenylbenzene

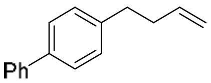

Following general procedure J, using (4-phenylphenyl)methanol (1.00 g, 5.43 mmol) and allymagnesium chloride (5.43 mL of a 2.0 M solution in THF). Purification by flash chromatography on silica gel, eluting with 5% DCM in hexane afforded the title compound (816 mg, 3.92 mmol, 72%) as a colourless oil.

^{1}H NMR (400 MHz, $CDCl_3$) δ 7.67-7.17 (9H, m, ArH), 5.88 (1H, ddt, *J* = 17.0, 10.2, 6.6, butenyl H-C3), 5.07 (1H, dd, *J* = 17.0, 1.6, butenyl H_{trans}-C4), 5.02-4.97 (1H, m, butenyl H_{cis}-C4), 2.81-2.69 (2H, m, butenyl H_2-C1), 2.46-2.36 (2H, m, butenyl H_2-C2);

^{13}C NMR (100 MHz, $CDCl_3$) δ 141.3 (C_q), 141.1 (C_q), 138.9 (C_q), 138.2 (butenyl C3), 129.0 (2 × C, ArC), 128.9 (2 × C, ArC), 127.2 (2 × C, ArC), 127.1 (2 × C, ArC), 115.2 (butenyl C4), 35.6 (butenyl C2), 35.2 (butenyl C1) one ^{13}C signal missing;

IR ν_{max} (neat)/cm^{-1}: 3077, 3027, 2924, 2853, 1639, 1601, 1519, 1486.

3-(4-Phenylphenyl)propanal

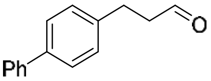

Following general procedure L, using 1-(but-3-en-1-yl)-4-phenylbenzene (750 mg, 3.60 mmol). Purification by flash chromatography on silica gel, eluting with 5% Et_2O in hexane afforded the *title compound* (640 mg, 3.04 mmol, 84%) as a colourless oil. The data is in accordance with the literature.

^{1}H NMR (400 MHz, $CDCl_3$) δ 9.86 (1H, s, propyl H-C1), 7.64-7.51 (4H, m, ArH), 7.48-7.22 (5H, m, ArH), 3.01 (2H, t, *J* = 7.5, propyl H_2-C3), 2.83 (2H, t, *J* = 7.4, propyl H_2-C2);

^{13}C NMR (100 MHz, $CDCl_3$) δ 201.6 (propyl C1), 141.0 (C_q), 139.6 (C_q), 139.5 (C_q), 128.89 (2 × C, ArC), 128.87 (2 × C, ArC), 127.5 (2 × C, ArC), 127.3 (ArC), 127.2 (2 × C, ArC), 45.4 (propyl C2), 27.9 (propyl C3);

IR ν_{max} (neat)/cm^{-1}: 3029, 2944, 2821, 2725, 1709 (C=O), 1597, 1582, 1563;

N-Methyl[3-(4-phenylphenyl)propyl]amine

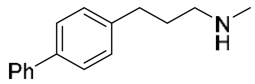

Following general procedure A, using 3-(4-phenylphenyl)propanal (550 mg, 2.62 mmol) and $MeNH_2$ (3.00 mL of an 8.0 M solution in EtOH) afforded the *title compound* (554 mg, 2.46 mmol, 94%) as a colourless gum.

^{1}H NMR (400 MHz, $CDCl_3$) δ 7.60-7.23 (9H, m, ArH), 2.74-2.60 (4H, m, includes 2H, m, propyl H_2-C1; and 2H, m, propyl H_2-C3), 2.44 (3H, s, NCH_3), 1.94-1.77 (3H, m, includes 1H, br. s, NH; and 2H, m, propyl H_2-C2);

^{13}C NMR (100 MHz, $CDCl_3$) δ 141.4 (C_q), 141.2 (C_q), 138.9 (C_q), 128.9 (2 × C, ArC), 128.8 (2 × C, ArC), 127.2 (2 × C, ArC), 127.14 (ArC), 127.11 (2 × C, ArC), 51.7 (propyl C1), 36.5 (NCH_3), 33.4 (propyl C3), 31.5 (propyl C2);

IR ν_{max} (neat)/cm^{-1}: 3314, 3055, 3027, 2930, 2852, 2789, 1601, 1563;

HRMS (ESI): $C_{16}H_{20}N$ [M+H$^+$]: calculated 226.1590, found 226.1586.

1-(But-3-en-1-yl)-4-chlorobenzene

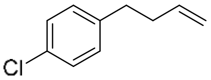

Following general procedure J, using 4-chlorobenzyl alcohol (1.30 g, 9.12 mmol) and allylmagensium bromide (9.10 mL of 2.0 M solution in THF). Purification by flash chromatography on silica gel, eluting with 10% EtOAc in hexane afforded the title compound (640 mg, 3.84 mmol, 42%) as a colourless oil. The NMR data is in accordance with the literature.[9]

^{1}H NMR (400 MHz, $CDCl_3$) δ 7.24 (2H, d, *J* = 8.6, ArH), 7.11 (2H, d, *J* = 8.6, ArH), 5.88-5.77 (1H, m, butenyl H-C3), 5.06-4.95 (2H, m, butenyl H_2-C4), 2.68 (2H, t, *J* = 7.7, butenyl H_2-C1), 2.39-2.30 (2H, m, butenyl H_2-C2);

^{13}C NMR (75 MHz, $CDCl_3$) δ 140.3 (C_q), 137.6 (butenyl C2), 131.5 (C_q), 129.8 (2 × C, ArC), 128.4 (2 × C, ArC), 115.3 (butenyl C1), 35.4 (butenyl C3), 34.7 (butenyl C4);

IR ν_{max} (neat)/cm^{-1}: 3078, 3027, 2978, 2928, 2856, 1641, 1491, 1439.

3-(4-Chlorophenyl)propanal

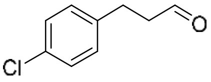

Following general procedure L, using 1-(but-3-en-1-yl)-4-chlorobenzene (550 mg, 3.30 mmol). Purification by flash chromatography on silica gel, eluting with 10% Et_2O in hexane afforded the

title compound (481 mg, 2.85 mmol, 87%) as a colourless oil. The NMR data is in accordance with the literature.[10]

^{1}H NMR (500 MHz, $CDCl_3$) δ 9.84 (1H, t, *J* = 1.2, propyl H-C1), 7.29 (2H, d, *J* = 8.2, ArH), 7.16 (2H, d, *J* = 8.2, ArH), 2.96 (2H, t, *J* = 7.5, propyl H_2-C3), 2.83-2.78 (2H, m, propyl H_2-C2);

^{13}C NMR (126 MHz, $CDCl_3$) δ ppm 201.0 (propyl C1), 138.8 (C_q), 132.1 (C_q), 129.7 (2 × ArC), 128.7 (2 × ArC), 45.1 (propyl C2), 27.4 (propyl C3);

IR ν_{max} (neat) / cm^{-1}: 3028, 2929, 2894, 2725, 1720, 1492, 1447, 1408.

[3-(4-Chlorophenyl)propyl](methyl)amine

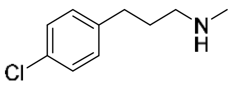

Following general procedure A, using 3-(4-chlorophenyl)propanal (300 mg, 1.78 mmol) and $MeNH_2$ (0.5 mL of an 8 M solution in EtOH). Purification by SCX cartridge afforded the title compound (288 mg, 1.57 mmol, 88%) as a yellow oil. The ^{1}H NMR data is in accordance with the literature.[11]

^{1}H NMR (400 MHz, $CDCl_3$) δ 7.30-7.22 (2H, m, ArH), 7.17-7.10 (2H, m, ArH), 2.69-2.60 (2H, m, propyl H_2-C1), 2.45 (3H, s, NCH_3), 2.16-1.96 (2H, m, propyl H_2-C3), 1.90-1.76 (2H, m, propyl H_2-C2);

^{13}C NMR (100 MHz, $CDCl_3$) δ 140.5 (C_q), 131.5 (C_q), 129.7 (2 × C, ArC), 128.5 (2 × C, ArC), 51.3 (propyl C1), 36.3 (NCH_3), 32.9 (propyl C3), 31.2 (propyl C2);

IR ν_{max} (neat) / cm^{-1}: 3025, 2933, 2857, 2793, 1632, 1538, 1490, 1383;

HRMS (ESI): $C_{10}H_{15}^{35}ClN$ [M+H$^+$]: calculated 184.0888, found 184.0892.

1-(But-3-en-1-yl)-3-chlorobenzene

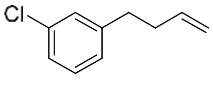

Following general procedure K, using 3-chlorobenzyl bromide (1.50 g, 7.30 mmol) and allymagnesium chloride (7.30 mL of 2.0 M solution in THF). Purification by flash chromatography on silica gel, eluting with 10% EtOAc in hexane afforded the *title compound* (1.20 g, 7.20 mmol, 99%) as a colourless oil. The data is in accordance with the literature.[9]

^{1}H NMR (400 MHz, $CDCl_3$) δ 7.26-7.17 (3H, m, ArH), 7.09 (1H, d, *J* = 7.2, ArH), 5.92-5.80 (1H, m, butenyl H-C3), 5.12-4.98 (2H, m, butenyl H_2-C4), 2.72 (2H, t, *J* = 7.7, butenyl H_2-C1), 2.39 (2H, m, butenyl H_2-C2);

^{13}C NMR (100 MHz, $CDCl_3$) δ 143.9 (C_q), 137.5 (butenyl C3), 134.1 (C_q), 129.5 (ArC), 128.6 (ArC), 126.7 (ArC), 126.0 (ArC), 115.3 (butenyl C4), 35.2 (butenyl C2), 35.0 (butenyl C1);

IR ν_{max} (neat) / cm^{-1}: 3077, 2978, 2928, 2857, 1640, 1598, 1573, 1476.

3-(3-Chlorophenyl)propanal

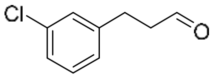

Following general procedure L, using 1-(but-3-en-1-yl)-3-chlorobenzene (1.00 g, 6.00mmol). Purification by flash chromatography on silica gel, eluting with 10% Et_2O in hexane afforded the title compound (766 mg, 4.54 mmol, 76%) as a colourless oil. The data is in accordance with the literature.[10]

^{1}H NMR (400 MHz, $CDCl_3$) δ 9.84 (1H, s, propyl H-C1), 7.30-7.18 (3H, m, ArH), 7.10 (1H, d, *J* = 7.1, ArH), 2.96 (2H, t, *J* = 7.4, propyl H_2-C2), 2.81 (2H, t, *J* = 7.4, propyl H_2-C3);

^{13}C NMR (100 MHz, $CDCl_3$) δ 200.9 (propyl C1), 142.4 (C_q), 134.3 (C_q), 129.9 (ArC), 128.5 (ArC), 126.6 (ArC), 126.5 (ArC), 45.0 (propyl C2), 27.7 (propyl C3);

IR ν_{max} (neat) / cm^{-1}: 3019, 2928, 2894, 2824, 2724, 1721 (C=O), 1598, 1573.

[3-(3-Chlorophenyl)propyl](methyl)amine

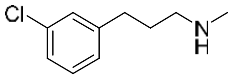

Following general procedure A, using 3-(3-chlorophenyl)propanal (700 mg, 4.15 mmol) and $MeNH_2$ (1.50 mL of a 8.0 M solution in EtOH). Purification by SCX cartridge afforded the *title compound* (672 mg, 3.66 mmol, 88%) as a colourless oil.

^{1}H NMR (400 MHz, $CDCl_3$) δ 7.26-7.15 (3H, m, ArH), 7.08 (1H, d, *J* = 7.2, ArH), 2.70-2.61 (4H, m, including 2H, m, propyl H_2-C1; and 2H, m, propyl H_2-C3), 2.47 (3H, s, NCH_3), 1.91-1.80 (2H, m, propyl H_2-C2);

^{13}C NMR (100 MHz, $CDCl_3$) δ 144.0 (C_q), 134.1 (C_q), 129.6 (ArC), 128.5 (ArC), 126.6 (ArC), 126.1 (ArC), 51.1 (propyl C1), 36.1 (NCH_3), 33.2 (propyl C3), 30.8 (propyl C2);

IR ν_{max} (neat) / cm^{-1}: 3059, 2935, 2858, 2796, 1596, 1571, 1536, 1473;

HRMS (ESI): $C_{10}H_{15}{}^{35}ClN$ [$M+H^+$]: calculated 184.0888, found 184.0886.

1-(But-3-en-1-yl)-4-trifluoromethylbenzene

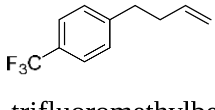

Following general procedure J, using 4-trifluoromethylbenzyl alcohol (700 mg, 3.96 mmol) and allylmagensium bromide (3.96 mL of 2.0 M solution in THF). Purification by flash chromatography on silica gel, eluting with 10% EtOAc in hexane afforded the title compound (354 mg, 1.77 mmol, 45%) as a colourless oil. The data is in accordance with the literature.[12]

^{1}H NMR (400 MHz, $CDCl_3$) δ 7.54 (2H, d, *J* = 8.1, ArH), 7.30 (2H, d, *J* = 8.1, ArH), 5.84 (1H, ddt, *J* = 16.9, 10.2, 6.6, butenyl H-C3), 5.09-4.98 (2H, m, butenyl H_2-C4), 2.78 (2H, t, *J* = 7.5, butenyl H_2-C1), 2.40 (2H, app. dd, *J* = 14.7, 7.5, butenyl H_2-C2);

^{13}C NMR (100 MHz, $CDCl_3$) δ 146.1 (C_q), 137.5 (butenyl C3), 128.9 (2 × C, Ar C2), 128.3 (q, *J* = 32.2, C_q), 125.4 (2 × C, q, *J* = 3.7, Ar C3), 124.5 (q, *J* = 271.7, CF_3), 115.6 (butenyl C4), 35.3 (butenyl C1), 35.2 (butenyl C2);

IR ν_{max} (neat)/cm^{-1}: 3082, 2982, 2932, 2861, 1642, 1619, 1418, 1322.

3-(4-Trifluoromethylphenyl)propanal

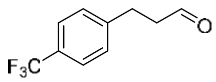

Following general procedure L, using 1-(but-3-en-1-yl)-4-trifluoromethylbenzene (300 mg, 1.50 mmol). Purification by flash chromatography on silica gel, eluting with 10% diethyl ether in hexane afforded the title compound (256 mg, 1.27 mmol, 85%) as a colourless oil. The data is in accordance with the literature.[13]

^{1}H NMR (500 MHz, $CDCl_3$) δ 9.82 (1H, s, propyl H-C1), 7.55 (2H, d, *J* = 8.1, ArH), 7.31 (2H, d, *J* = 8.1, ArH), 3.01 (2H, t, *J* = 7.4, propyl H_2-C3), 2.82 (2H, t, *J* = 7.4, propyl H_2-C2);

^{13}C NMR (100 MHz, $CDCl_3$) δ 200.8 (propyl C1), 144.7 (C_q), 128.9 (q, *J* = 32.3, C_q), 128.8 (2 × C, Ar C2), 125.7 (2 × C, q, *J* = 3.6, Ar C3), 124.9 (q, *J* = 271.8, CF_3), 45.0 (propyl C2), 27.9 (propyl C3);

IR ν_{max} (neat) / cm^{-1}: 2936, 2829, 2730, 1723 (C=O), 1619, 1585, 1419, 1322.

[3-(4-Trifluoromethylphenyl)propyl](methyl)amine

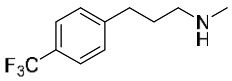

Following general procedure A, using 3-(4-trifluoromethylphenyl)propanal (200 mg, 0.99 mmol) and $MeNH_2$ (1.25 mL of an 8 M solution in EtOH). Purification by SCX cartridge afforded the title compound (180 mg, 0.83 mmol, 83%) as a yellow oil.

^{1}H NMR (400 MHz, $CDCl_3$) (400 MHz, $CDCl_3$) δ 7.52 (2H, d, *J* = 8.0, ArH), 7.28 (2H, d, *J* = 8.0, ArH), 2.71 (2H, t, *J* = 7.7, propyl H_2-C3), 2.60 (2H, t, *J* = 7.1, propyl H_2-C1), 2.42 (3H, s, NCH_3), 1.85-1.77 (2H, m, propyl H_2-C2);

^{13}C NMR (100 MHz, $CDCl_3$) δ 146.4 (C_q), 128.8 (2 × C, Ar C2), 128.3 (q, *J* = 32.3, Ar C3), 125.4 (2 × C, q, *J* = 3.9, Ar C3), 124.3 (q. *J* = 271.7, CF_3), 51.5 (propyl C1), 36.5 (NCH_3), 33.5 (propyl C3), 31.3 (propyl C2);

IR ν_{max} (neat) / cm^{-1}: 3294, 2938, 2862, 1619, 1537, 1476, 1418, 1322;

HRMS (ESI): $C_{11}H_{15}F_3N$ [M+H$^+$]: calculated 218.1151, found 218.1151.

1-(But-3-en-1-yl)-4-bromoebenzene

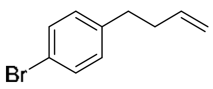

Following general procedure K, using 4-bromobenzyl bromide (10.0 g, 40.0 mmol) and allyl magnesium chloride (20 mL of 2.0 M solution in THF) afforded the title compound (8.52 g, 40.0 mmol, quant.) as a colourless oil. The data is in accordance with the literature.[12]

^{1}H NMR (500 MHz, $CDCl_3$) δ 7.40 (2H, d, *J* = 8.4, ArH), 7.06 (2H, d, *J* = 8.5, ArH), 5.83 (1H, ddt, *J* = 17.0, 10.2, 6.6, butenyl H-C3), 5.04 (1H, ddd, *J* = 17.0, 3.4, butenyl H_{trans}-C4), 4.99 (1H, dd, J = 10.2, 1.9, butenyl H_{cis}-C4), 2.71-2.61 (2H, m, butenyl H_2-C1), 2.35 (2H, app. dtt, J = 9.0, 7.8, 1.3, butenyl H_2-C2);

^{13}C NMR (125 MHz, $CDCl_3$) δ 140.9 (C_q), 137.7 (butenyl C3), 131.5 (2 × C, ArC), 130.4 (2 × C, ArC), 119.7 (C_q), 115.4 (butenyl H_2-C4), 35.4 (butenyl C2), 34.9 (butenyl C1);

IR ν_{max} (neat)/cm^{-1}: 3078, 3024, 2978, 2929, 2857, 1641, 1487, 1439.

3-(4-Bromophenyl)propanal

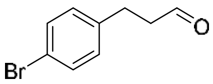

Following general procedure L, using 1-(but-3-en-1-yl)-4-bromoebenzene (8.10 g, 38.1 mmol). Purification by flash chromatography on silica gel, eluting with a gradient of 20-40% Et_2O in hexane afforded the title compound (7.13 g, 33.5 mmol, 88%) as a pale yellow oil. The data is in accordance with the literature.[14]

^{1}H NMR (400 MHz, $CDCl_3$) δ 9.80 (1H, s, propyl H-C1), 7.40 (2H, t, *J* = 8.3, ArH), 7.07 (2H, d, *J* = 8.3, ArH), 2.90 (2H, t, *J* = 7.4, propyl H_2-C3), 2.76 (2H, t, *J* = 7.4, propyl H_2-C2);

^{13}C NMR (100 MHz, $CDCl_3$) δ 201.1 (propyl C1), 139.5 (C_q), 131.8 (2 × C, ArC), 130.2 (2 × C, ArC), 120.2 (C_q), 45.1 (propyl C3), 27.6 (propyl C2);

IR ν_{max} (neat)/cm^{-1}: 2930, 2823, 2724, 1719 (C=O), 1591, 1487, 1438, 1404.

[3-(4-Bromophenyl)propyl](methyl)amine

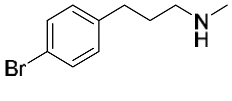

Following general procedure A, using 3-(4-bromophenyl)propanal (5.00 g, 23.5 mmol) and methylamine (30.0 mL of a 8.0 M solution in EtOH, 235 mmol) afforded the *title compound* (5.31 g, 23.3 mmol, 99%) as a yellow oil.

^{1}H NMR (400 MHz, $CDCl_3$) δ 7.38 (2H, d, *J* = 8.3, ArH), 7.05 (2H, d, *J* = 8.3, ArH), 2.64-2.55 (4H, m, includes 2H, m, propyl H_2-C1; and 2H, m, propyl H_2-C3), 2.41 (3H, s, NCH_3), 1.83-1.73 (2H, m, propyl H_2-C2);

^{13}C NMR (100 MHz, $CDCl_3$) δ 141.1 (C_q), 131.5 (2 × C, ArC), 130.2 (2 × C, ArC), 119.6 (C_q), 51.3 (propyl C1), 36.4 (NCH_3), 33.1 (propyl C3), 31.2 (propyl C2);

IR ν_{max} (neat)/cm^{-1}: 3310 (N-H), 3023, 2932, 2857, 2798, 1537, 1487, 1452;

HRMS (ESI): $C_{10}H_{15}{}^{79}BrN$ [$M+H^+$]: calculated 228.0382, found 228.0376.

tert-Butyl-*N*-[3-(4-bromophenyl)propyl]-*N*-methylcarbamate

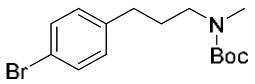

To a stirred solution of [3-(4-bromophenyl)propyl](methyl)amine (5.00 g, 21.9 mmol, 1.0 eq.) in DCM (60 mL) was added Et_3N (6.11 mL, 43.8 mmol, 2.0 eq.), Boc_2O (5.26 g, 24.1 mmol, 1.1 eq.) and DMAP (269 mg, 2.20 mmol, 0.1 eq.). The reaction mixture was stirred at rt for 1 h then was diluted with H_2O (100 mL) and the aqueous phase was extracted with EtOAc (3 × 75 mL). The combined organic extracts were washed with H_2O (100 mL), dried over Na_2SO_4 and concentrated *in vacuo*. Purification by flash chromatography on silica gel, eluting with a gradient of 0-40% EtOAc in hexane afforded the *title compound* (5.74 g, 17.5 mmol, 80%) as a colourless oil.

^{1}H NMR (400 MHz, $CDCl_3$) δ 7.38 (2H, d, *J* = 8.2, ArH), 7.05 (2H, d, *J* = 8.2, ArH), 3.27 (2H, br. s, propyl H_2-C1), 2.83 (3H, br. s, NCH_3), 2.59-2.47 (2H, m, propyl H_2-C3), 1.86-1.73 (2H, m, propyl H_2-C2), 1.43 (9H, s, $OC(CH_3)_3$);

^{13}C NMR (100 MHz, $CDCl_3$) δ 155.9 (C=O), 140.8 (C_q), 131.5 (2 × C, ArC), 130.2 (2 × C, ArC), 119.7 (C_q), 79.4 (C_q), 48.4 (propyl C1), 34.2 (NCH_3), 32.6 (propyl C3), 29.4 (propyl C2), 28.6 (3 × C, $OC(CH_3)_3$);

IR ν_{max} (neat)/cm^{-1}: 2974, 2930, 2863, 1688 (C=O), 1487, 1453, 1423, 1391;

HRMS (ESI): $C_{15}H_{22}{}^{17}BrNO_2Na$ [$M+Na^+$]: calculated 350.0726, found 350.0721.

tert-Butyl-*N*-methyl-*N*-{3-[4-(tetramethyl-1,3,2-dioxaborolan-2-yl)phenyl]propyl}carbamate

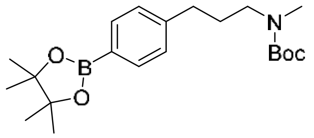

To a stirred solution of *tert*-butyl-*N*-[3-(4-bromophenyl)propyl]-*N*-methylcarbamate (750 mg, 2.28 mmol, 1.0 eq.) in DMSO (9.2 mL) was added $PdCl_2$(dppf)•DCM (73 mg, 0.09 mmol, 0.05 eq.), $B_2(pin)_2$ (580 mg, 2.28 mmol, 1.0 eq.) and KOAc (538 mg, 5.49 mmol, 3.0 eq.). The reaction mixture was heated at 90 °C for 18 h then cooled to rt and diluted with H_2O (75 mL). The aqueous phase was extracted with hexane (2 × 50 mL) then hexane:Et_2O (1:1 v/v, 2 × 50 mL). The combined organic extracts were washed with brine (50 mL), dried over Na_2SO_4 and

concentrated *in vacuo*. Purification by flash chromatography on silica gel, eluting with 10% EtOAc in hexane afforded the *title compound* (532 mg, 1.42 mmol, 62%) as a colourless solid.

^{1}H NMR (400 MHz, $CDCl_3$) δ 7.73 (2H, d, *J* = 7.8, ArH), 7.19 (2H, d, *J* = 7.8, ArH), 3.23 (2H, br. s, propyl H_2-C1), 2.83 (3H, br. s, NCH_3), 2.61 (2H, t, *J* = 7.7, propyl H_2-C3), 1.88-1.77 (2H, m. propyl H_2-C2), 1.44 (9H, s, $OC(CH_3)_3$), 1.33 (12H, s, 2 × $OC(CH_3)_2$);

^{13}C NMR (100 MHz, $CDCl_3$) δ 156.0 (C=O), 145.3 (C_q), 135.1 (2 × C, ArC), 127.9 (2 × C, ArC), 83.8 (2 × C, O*C*$(CH_3)_2$), 79.4 (O*C*$(CH_3)_3$), 48.7 (propyl C1), 34.3 (NCH_3), 33.4 (propyl C3), 29.5 (propyl C2), 28.6 (3 × C, OC(*C*$H_3)_3$), 25.0 (4 × C, OC(*C*$H_3)_2$) one ArC_q signal missing;

IR ν_{max} (neat)/cm^{-1}: 2980, 2970, 2934, 2862, 1681 (C=O), 1611, 1483, 1458;

HRMS (ESI): $C_{21}H_{34}BNO_4Na$ [M+Na$^+$]: calculated 398.2473, found 398.2475.

Methyl({3-[4-(tetramethyl-1,3,2-dioxaborolan-2-yl)phenyl]propyl})amine

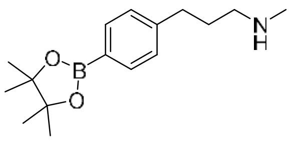

To a stirred solution of *tert*-butyl-*N*-methyl-*N*-{3-[4-(tetramethyl-1,3,2-dioxaborolan-2-yl)phenyl]propyl}carbamate (400 mg, 1.07 mmol, 1.0 eq.) in DCM (3 mL) at 0 °C was added TFA (3 mL) portionwise. The reaction mixture was warmed to rt and stirred for 1 h then concentrated *in vacuo*. The residue was then dissolved in DCM (5 mL) and K_2CO_3 (663 mg, 4.80 mmol, 5 eq.) was added portionwise. The reaction mixture was stirred at RT for 1 h, then diluted with H_2O (10 mL) and the aqueous phase was extracted with EtOAc (2 × 20 mL). The combined organic extracts were washed with brine (10 mL), dried over Na_2SO_4 and concentrated *in vacuo*. Purificatiom by SCX-cartridge afforded the *title compound* (253 mg, 0.92 mmol, 86%) as a yellow oil.

^{1}H NMR (400 MHz, $CDCl_3$) δ 7.73 (2H, d, *J* = 7.9, ArH), 7.20 (2H, d, *J* = 7.9, ArH), 2.72-2.63 (2H, m, propyl H_2-C1), 2.63-2.57 (2H, m, propyl H_2-C3), 2.42 (3H, s, NCH_3), 1.81 (2H, dt, *J* = 14.5, 7.4, propyl H_2-C2), 1.33 (12H, s, 2 × $OC(CH_3)_2$);

^{13}C NMR (100 MHz, $CDCl_3$) δ 145.8 (C_q), 135.1 (2 × C, ArC), 128.0 (2 × C, ArC), 83.8 (2 × C, O*C*$(CH_3)_2$), 51.8 (propyl C1), 36.6 (NCH_3), 34.0 (propyl C3), 31.5 (propyl C2), 25.0 (4 × C, OC(*C*$H_3)_2$) one ArC_q signal missing;

IR ν_{max} (neat)/cm^{-1}: 3305 (N-H), 2977, 2932, 2861, 1678, 1611, 1518, 1460;

HRMS (ESI): $C_{16}H_{27}BNO_2$ [M+H$^+$]: calculated 276.2129, found 276.2131.

Route F

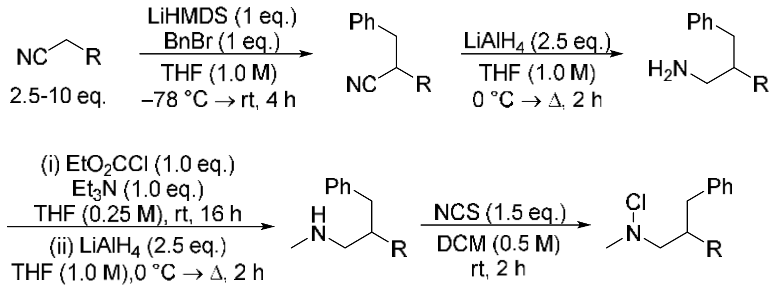

General Procedure M: Nitrile Alkylation

To a stirred solution of nitrile (2.5 – 10 eq.) in THF (1.0 M) at –78 °C was added a solution of LiHMDS (1.0 eq.) dropwise. The reaction mixture was stirred for 1 h then a solution of benzyl bromide (1.0 eq.) in THF (1.0 M) was added dropwise. The reaction mixture was stirred for 0.5 h then warmed to rt and stirred for 2.5 h, before being concentrated *in vacuo* and taken up in H_2O (1 vol). The aqueous phase was extracted with EtOAc (× 3) and the combined organic extracts were washed with brine (1 vol), dried over Na_2SO_4 and concentrated *in vacuo*. Purification afforded the desired products.

General Procedure N: Carbamate Formation and Reduction

To a stirred solution of amine (1.0 eq.) in THF (0.25 M) at 0 °C, was added Et_3N (1.0 eq.) and ethyl chloroformate (1.0 eq.). The reaction mixture was warmed to rt, stirred for 16 h then the reaction was quenched with H_2O (1 vol) and extracted with EtOAc (× 3). The combined organic extracts were washed with brine (1 vol), dried over Na_2SO_4 and concentrated *in vacuo*. To a stirred suspension of $LiAlH_4$ (2.0 eq.) in THF (1.0 M) at 0 °C was added a solution of the crude carbamate (1.0 eq.) in THF (1.0 M) dropwise. The reaction mixture was heated at reflux for 2 h, then cooled to 0 °C and the reaction was quenched with H_2O (1.0 eq.), 2 M aqueous NaOH (1.0 eq.) and H_2O (5.0 eq.) then stirred for 1 h at rt until the reaction mixture had turned colourless. The resultant slurry was dried over Na_2SO_4, filtered through a pad of Celite and the Celite was washed with EtOAc. The filtrate was concentrated *in vacuo*. Purification afforded the desired products.

2-Methyl-3-phenylpropanenitrile

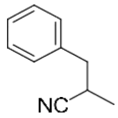

Following general procedure M, using propionitrile (7.10 mL, 100 mmol). Purification by flash chromatography on silica gel, eluting with a gradient of 2-10% EtOAc in hexane afforded the *title*

compound (1.18 g, 8.13 mmol, 81%) as a colourless oil. The data is in accordance with the literature.[15]

^{1}H NMR (300 MHz, $CDCl_3$) δ 7.30-7.12 (5H, m, ArH), 2.93-2.70 (3H, m, includes 1H, m, propyl H-C2; and 2H, m, propyl H_2-C3), 1.25 (3H, d, *J* = 6.7, CH_3);

^{13}C NMR (75 MHz, $CDCl_3$) δ 137.0 (C_q), 129.2 (2 × C, ArC), 128.8 (2 × C, ArC), 127.4 (ArC), 122.7 (propyl C1), 40.1 (propyl C3), 27.7 (propyl C2), 17.7 (CH_3);

IR ν_{max} (neat)/cm^{-1}: 3030, 2983, 2938, 2238 (C≡N), 1496, 1454, 1083, 699.

2-Methyl-3-phenylpropan-1-amine

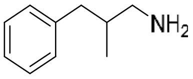

Following general procedure F, using 2-methyl-3-phenylpropanenitrile (1.25 g, 8.61 mmol). Purification by flash chromatography on silica gel, eluting with EtOAc (+ 1% Et_3N) then 10% MeOH in DCM (+ 1% Et_3N) afforded the title compound (331 mg, 2.22 mmol, 26%) as a yellow oil. The data is in accordance with the literature.[16]

^{1}H NMR (500 MHz, $CDCl_3$) δ 7.31-7.24 (2H, m, ArH), 7.21-7.13 (3H, m, ArH), 2.70 (1H, dd, *J* = 13.4, 6.2, propyl H_a-C3), 2.67-2.64 (1H, m, propyl H_a-C1), 2.53 (1H, dd, *J* = 12.5, 6.9, propyl H_b-C1), 2.38 (1H, dd, *J* = 13.4, 8.2, propyl H_b-C3), 2.00-1.72 (3H, m, includes 2H, br. s, NH_2, and 1H, m, propyl H-C2), 0.89 (3H, d, *J* = 6.7, CH_3);

^{13}C NMR (125 MHz, $CDCl_3$) δ 140.9 (C_q), 129.1 (2 × C, ArC), 128.2 (2 × C, ArC), 125.8 (ArC), 48.0 (propyl C1), 41.0 (propyl C3), 38.3 (propyl C2), 17.4 (CH_3);

HRMS (ESI): $C_{10}H_{16}N$ [M+H$^+$]: calculated 150.1277, found 150.1275.

Methyl(2-methyl-3-phenylpropyl)amine

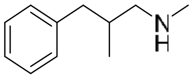

Following general procedure N, using 2-methyl-3-phenylpropan-1-amine (300 mg, 2.01 mmol) afforded the *title compound* (224 mg, 1.36 mmol, 99%) as a colourless oil.

^{1}H NMR (500 MHz, $CDCl_3$) δ 7.30-7.24 (2H, m, ArH), 7.21-7.13 (3H, m, ArH), 2.72 (1H, dd, *J* = 13.4, 6.0, propyl H_a-C3), 2.54 (1H, dd, *J* = 11.6, 5.9, propyl H_a-C1), 2.45-2.35 (5H, m, includes 3H, s, NCH_3, 1H, m, propyl H_b-C3; and 1H, m, propyl H_b-C1), 1.94 (1H, td, *J* = 13.1, 6.6, propyl H-C2), 0.89 (3H, d, *J* = 6.6, CH_3);

^{13}C NMR (125 MHz, $CDCl_3$) δ 141.1 (C_q), 129.3 (2 × C, ArC), 128.3 (2 × C, ArC), 125.9 (ArC), 58.5 (propyl C1), 41.7 (propyl C3), 36.8 (NCH_3), 35.4 (propyl C2), 18.1 (CH_3);

IR ν_{max} (neat)/cm^{-1}: 3062, 3026, 2954, 2922, 1603, 1542, 1493, 1453;

HRMS (ESI): $C_{11}H_{18}N$ [M+H$^+$]: calculated 164.1434, found 164.1439.

Miscellaneous amine synthesis

1-[Benzyl(methyl)amino]-3-phenylpropan-2-ol

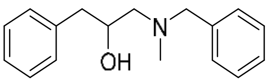

To a stirred solution of (2,3-epoxypropyl)benzene (1.00 g, 7.50 mmol, 1.0 eq.) in MeOH (15 mL) was added K_2CO_3 (5.18 g, 37.5 mmol, 5.0 eq.) and BnMeNH (4.84 mL, 37.5 mmol, 5.0 eq.). The reaction mixture was heated at reflux for 16 h and then the reaction mixture was cooled to rt and concentrated. The crude mixture was taken up in H_2O (30 mL) and extracted with EtOAc (3 × 25 mL). The combined organic extracts were washed with brine (30 mL), dried over Na_2SO_4 and concentrated *in vacuo*. Purification by flash chromatography on silica gel, eluting with EtOAc (+ 1% Et_3N) afforded the *title compound* (1.75 g, 6.85 mmol, 91%) as a pale yellow oil.

^{1}H NMR (400 MHz, $CDCl_3$) δ 7.36-7.19 (10H, m, ArH), 4.01-3.91 (1H, m, propyl H-C2), 3.66 (1H, d, *J* = 13.1, NCH_aH_b), 3.45 (1H, d, *J* = 13.1, NCH_aH_b), 2.83 (1H, dd, *J* = 13.7, 7.1, propyl H_a-C1), 2.67 (1H, dd, *J* = 13.7, 5.6, propyl H_b-C1), 2.47 (1H, dd, *J* = 12.2, 10.3, propyl H_a-C3), 2.37 (1H, dd, *J* = 12.2, 3.3, propyl H_b-C3), 2.21 (3H, s, NCH_3);

^{13}C NMR (100 MHz, $CDCl_3$) δ 138.54 (C_q), 138.52 (C_q), 129.4 (2 × C, ArC), 129.1 (2 × C, ArC), 128.5 (4 × C, ArC), 127.4 (ArC), 126.4 (ArC), 68.2 (propyl C2), 63.0 (propyl C3), 62.5 (NCH_2), 42.1 (NCH_3), 41.5 (propyl C1);

IR ν_{max} (neat)/cm^{-1}: 3425 (O-H), 3061, 3027, 2931, 2843, 2793, 1602, 1495;

HRMS (ESI): $C_{17}H_{22}NO$ [M+H$^+$]: calculated 256.1696, found 256.1696.

Benzyl(2-methoxy-3-phenylpropyl)methylamine

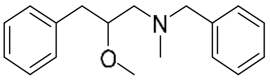

To a stirred suspension of NaH (156 mg of a 60% dispersion in mineral oil, 3.90 mmol, 1.0 eq.) in THF (5 mL) at 0 °C was added a solution of 1-[benzyl(methyl)amino]-3-phenylpropan-2-ol (1.00 g, 3.90 mmol, 1.0 eq.) in THF (5 mL) dropwise. The reaction mixture was stirred at 0 °C for 30 minutes then MeI (0.24 mL, 3.90 mmol, 1.0 eq.) was added portionwise. The reaction mixture was warmed to rt and stirred for 3 h, after which H_2O (20 mL) was added and the phases separated. The aqueous phase was extracted with EtOAc (3 × 20 mL) and the combined organic extracts were washed with brine (20 mL), dried over Na_2SO_4 and concentrated *in vacuo*. Purification by flash chromatography on silica gel, eluting with 25% EtOAc in hexane afforded *title compound* (494 mg, 1.83 mmol, 47%) as a colourless oil.

^{1}H NMR (400 MHz, $CDCl_3$) δ 7.38-7.16 (10H, m, ArH), 3.63-3.53 (3H, m, includes 1H, m, propyl H-C2; and 2H, m, NCH_2), 3.39 (3H, s, OCH_3), 2.93 (1H, dd, *J* = 13.9, 5.4, propyl H_a-C1),

2.82 (1H, dd, J = 13.9, 6.6, propyl H_b-C1), 2.54 (1H, dd, J = 13.0, 6.1, propyl H_a-C3), 2.47 (1H, dd, J = 13.0, 5.4, propyl H_b-C3), 2.26 (3H, s, NCH_3);

^{13}C NMR (100 MHz, $CDCl_3$) δ 139.3 (C_q), 139.2 (C_q), 129.6 (2 × C, ArC), 129.1 (2 × C, ArC), 128.3 (4 × C, ArC), 127.1 (ArC), 126.1 (ArC), 81.0 (NCH_2), 63.0 (propyl C2), 60.4 (propyl C3), 57.5 (OCH_3), 43.2 (NCH_3), 39.0 (propyl C1);

IR ν_{max} (neat)/cm^{-1}: 3085, 3062, 3027, 2976, 2928, 2823, 1703, 1602;

HRMS (ESI): $C_{18}H_{24}NO$ [M+H$^+$]: calculated 270.1852, found 270.1849.

(2-Methoxy-3-phenylpropyl)(methyl)amine

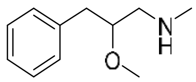

To a stirred solution of benzyl(2-methoxy-3-phenylpropyl)methylamine (400 mg, 1.49 mmol, 1.0 eq.) in degassed EtOH (6 mL) was added Pd/C (159 mg, 0.15 mol, 0.1 eq., 10% wt Pd). The solution was evacuated and flushed with N_2 (× 3) and then flushed with H_2 and left to stir under an atmosphere of H_2 for 48 h. The reaction mixture was filtered through a pad of Celite and the Ceilte was washed with EtOAc (200 mL). The filtrate was concentrated *in vacuo*. Purification by flash chromatography on silica gel, eluting with a gradient of 0-10% MeOH in DCM (+1% Et_3N) afforded the *title compound* (187 mg, 1.04 mmol, 70%) as a yellow oil.

^{1}H NMR (400 MHz, $CDCl_3$) δ 7.35-7.21 (5H, m, ArH), 3.69-3.58 (1H, m, propyl H-C2), 3.42 (3H, s, OCH_3), 2.95 (1H, dd, J = 13.8, 5.6, propyl H_a-C1), 2.76 (1H, dd, J = 13.8, 7.0, propyl H_b-C1), 2.67 (1H, dd, J = 12.3, 3.5, propyl H_a-C3), 2.60 (1H, dd, J = 12.3, 7.8, propyl H_b-C3), 2.46 (3H, s, NCH_3);

^{13}C NMR (100 MHz, $CDCl_3$) δ 138.2 (C_q), 129.5 (2 × C, ArC), 128.5 (2 × C, ArC), 126.4 (ArC), 81.1 (propyl C2), 57.5 (OCH_3), 54.5 (propyl C1), 38.3 (propyl C3), 36.2 (NCH_3);

IR ν_{max} (neat)/cm^{-1}: 3334 (N-H), 3027, 2930, 2826, 2794, 1603, 1495, 1454;

HRMS (ESI): $C_{11}H_{18}NO$ [M+H$^+$]: calculated 180.1383, found 180.1383.

Benzyl(4-phenylbutan-2-yl)amine

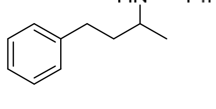

To a stirred solution of 4-phenylbutan-2-one (1.00 mL, 6.48 mmol, 1.0 eq.) in EtOH (20 mL) was added benzylamine (1.42 mL, 13.0 mmol, 2.0 eq.) and Ti(O^{i}Pr)$_4$ (3.85 mL, 13.0 mmol, 2.0 eq.). The reaction mixture was stirred for 5 h at rt then cooled to 0 °C and $NaBH_4$ (492 mg, 13.0 mmol, 2.0 eq.) was added portionwise. The reaction mixture was warmed to rt, then after 30 min was concentrated *in vacuo*, then taken up in EtOAc (25 mL) and aqueous NH_4OH (20 mL of a 2 M solution) was added to this. Na_2SO_4 was added and the crude mixture was filtered through a pad

of Celite, the Celite was washed with EtOAc (200 mL) and the filtrate was collected and concentrated *in vacuo*. Purification by automated flash chromatography on silica gel, eluting with a gradient of 20-100% EtOAc in hexane afforded the title compound (1.54 g, 6.43 mmol, 99%) as a colourless oil. The data is in accordance with the literature.[17]

^{1}H NMR (400 MHz, $CDCl_3$) δ 7.39-7.16 (10H, m, ArH), 3.85 (1H, d, *J* = 13.0, NCH_aH_b), 3.75 (1H, d, *J* = 13.0, NCH_aH_b), 2.81-2.70 (1H, m, butyl H-C2), 2.68 (2H, ddd, *J* = 9.4, 6.4, 4.9, butyl H_2-C4), 1.83 (1H, ddt, *J* = 13.0, 9.4, 6.4, butyl H_a-C3), 1.69 (1H, ddt, *J* = 13.0, 9.4, 6.4, butyl H_b-C3), 1.16 (3H, d, *J* = 6.3, butyl H_3-C1);

^{13}C NMR (100 MHz, $CDCl_3$) δ 142.6 (C_q), 141.0 (C_q), 128.5 (2 × C, ArC), 128.48 (2 × C, ArC), 128.47 (2 × C, ArC), 128.3 (2 × C, ArC), 127.0 (ArC), 125.8 (ArC), 52.2 (butyl C2), 51.5 (NCH_2), 38.9 (butyl C3), 32.2 (butyl C4), 20.5 (butyl C1);

IR ν_{max} (neat) / cm^{-1}: 3084, 3061, 3026, 2923, 2859, 1602, 1495, 1453;

HRMS (ESI): $C_{17}H_{22}N$ [M+H$^+$]: calculated 240.1747, found 240.1740.

Synthesis of methyl(1-phenyloctan-3-yl)amine

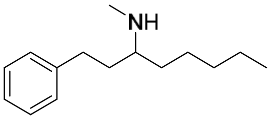

To a stirred solution of 3-phenyl propionitrile (1.00 g, 7.51 mmol, 1.0 eq.) in toluene (15 mL) at 0 °C was added pentylmagnesium bromide (3.75 mL of a 2.0 M solution in Et_2O, 1.0 eq.) dropwise. The reaction mixture was heated at reflux for 2 h, then cooled to 0 °C and EtO_2CCl (0.72 mL, 7.51 mmol, 1.0 eq.) was added dropwise. The reaction mixture was warmed to rt and stirred for 2 h, after which it was transferred by canular to a suspension of $LiAlH_4$ (1.14 g, 30.0 mmol, 4.0 eq.) in THF (30 mL) at 0 °C. The reaction mixture was then heated at reflux for 4 h, after which it was cooled to 0 °C and the reaction was quenched with H_2O (4 mL), 2.0 M aqueous NaOH (2 mL) and H_2O (2 mL), then stirred for 1 h at rt until the reaction mixture had turned white. The resultant slurry was dried over $MgSO_4$, filtered through a pad of Celite and the pad of Celite was washed with EtOAc (100 mL). The filtrate was concentrated *in vacuo* to afford the *title compound* **373** (1.22 g, 5.56 mmol, 74%) as a clear yellow oil. **^{1}H NMR** (400 MHz, $CDCl_3$) δ 7.31-7.14 (5H, m, ArH), 2.63 (2H, dd, *J* = 9.7, 6.7, octyl H_2-C1), 2.49-2.42 (1H, m, octyl H_1-C3), 2.39 (3H, s, NCH_3), 1.75-1.66 (2H, m, octyl H_2-C2), 1.48-1.39 (2H, m. octyl H_2-C4), 1.36-1.24 (6H, m, octyl H_2-C5-7), 0.94-0.83 (3H, m, octyl H_3-C8); **^{13}C NMR** (100 MHz, $CDCl_3$) δ 142.9 (C_q), 128.5 (4 × C, ArC), 125.8 (ArC4), 58.9 (octyl C3), 35.5 (octyl C1), 33.7 (octyl C4), 33.5 (NCH_3), 32.3 (octyl C2), 32.2 (CH_2), 25.5 (CH_2), 22.8 (CH_2), 14.2 (octyl C8); **IR** ν_{max} (neat)/cm^{-1}: 3062, 3026, 2926, 2856, 2788, 1603, 1495, 1454; **HRMS** (ESI): $C_{15}H_{26}N$ [M+H$^+$]: calculated 220.2060, found 220.2064.

2-(2-Phenylethyl)pyrrolidine

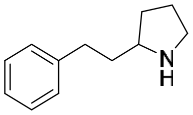

To a stirred solution of benzyltriphenylphosphonium bromide (2.17 g, 5.00 mmol, 4.0 eq.) in THF (10 mL) at 0 °C was added a solution of LiHMDS in THF (3.75 mL of a 1.0 M solution in THF, 3.0 eq.) dropwise. The reaction mixture turned bright orange upon addition. After 15 mins, a solution of *tert*-butyl 2-formylpyrrolidine-1-carboxylate (250 mg, 1.25 mmol, 1.0 eq.) in THF (2.5 mL) was added dropwise then the reaction mixture was warmed to rt and stirred for 16 h. The reaction was quenched with H_2O (10 mL) and the aqueous phase was extracted with Et_2O (3 × 15 mL). The combined organic extracts were washed with H_2O (3 × 20 mL), dried over Na_2SO_4 and concentrated *in vacuo*. The crude gum was dissolved in hexane/Et_2O (9:1, 10 mL) and filtered through a pad of silica, the silica washed with hexane/Et_2O (9:1, 150 mL), and the filtrate collected and concentrated *in vacuo* to give a pale yellow gum. The crude gum (306 mg) was taken up in EtOH (6 mL) and Pd/C (117 mg, 0.11 mol, 0.1 eq., 10% wt Pd) was added. The solution was evacuated and flushed with N_2 (× 3) and then flushed with H_2 and left to stir under an atmosphere of H_2 for 2 h. The reaction mixture was filtered through a pad of Celite and the Ceilte was washed with EtOAc (100 mL). The filtrate was concentrated *in vacuo*. The crude residue was taken up in DCM (6 mL) and the reaction mixture was cooled to 0 °C and TFA (5 mL) was added slowly. The reaction mixture was warmed to rt and stirred for 16 h, after which it was concentrated *in vacuo* and taken up in sat. aqueous K_2CO_3 (10 mL). The aqueous phase was extracted with EtOAc (3 × 10 mL) and the combined organic extracts were dried over Na_2SO_4 and concentrated *in vacuo*. Purification by SCX cartridge afforded the title compound (137 mg, 0.78 mmol, 62%) as a yellow oil.

^{1}H NMR (300 MHz, MeOD) δ 7.30-7.10 (5H, m, ArH), 3.02-2.87 (2H, m, includes 1H, m, pyrrolidinyl H-C2, and 1H, m, ethyl H_a-C2), 2.82-2.72 (1H, m, ethyl H_b-C2), 2.72-2.62 (2H, m, pyrrolidinyl H_2-C5), 2.00-1.87 (1H, m, ethyl H_a-C1), 1.87-1.64 (4H, m, includes 2H, m, pyrrolidinyl H_2-C3; and 2H, m, pyrrolidinyl H_2-C4), 1.40-1.23 (1H, m, ethyl H_b-C1);

^{13}C NMR (75 MHz, MeOD) δ 143.4 (C_q), 129.4 (2 × C, ArC), 129.4 (2 × C, ArC), 126.8 (ArC), 59.9 (pyrrolidinyl C2), 46.9 (ethyl C2), 38.7 (pyrrolidinyl C3), 34.8 (pyrrolidinyl C5), 32.6 (ethyl C1), 26.1 (pyrrolidinyl C4);

IR ν_{max} (neat)/cm^{-1}: 3061, 3025, 2935, 2858, 1603, 1495, 1454, 1365;

HRMS (ESI): $C_{12}H_{18}N$ [M+H$^+$]: calculated 176.1434, found 176.1437.

2-(2-Phenylethyl)-piperidine

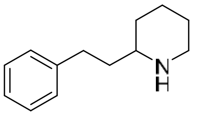

To a stirred solution of benzyltriphenylphosphonium bromide (3.05 g, 7.04 mmol, 1.5 eq.) in THF (14 mL) at 0 °C was added a solution of LiHMDS in THF (6.1 mL of a 1.0 M solution in THF, 1.3 eq.) dropwise. The reaction mixture turned bright orange upon addition. After 15 mins, a solution of *tert*-butyl 2-formylpiperidine-1-carboxylate (1 g, 4.69 mmol, 1.0 eq.) in THF (4 mL) was added dropwise then the reaction mixture was warmed to rt and stirred for 2 h. The reaction was quenched with H_2O (30 mL) and the aqueous phase was extracted with Et_2O (3 × 25 mL). The combined organic extracts were washed with H_2O (3 × 20 mL), dried over Na_2SO_4 and concentrated *in vacuo*. The crude gum was dissolved in hexane/Et_2O (9:1, 20 mL) and filtered through a pad of silica, the silica washed with hexane/Et_2O (9:1, 200 mL), and the filtrate collected and concentrated *in vacuo* to give a pale yellow gum. The crude gum (1.00 g) was taken up in MeOH (30 mL) and Pd/C (369 mg, 0.11 mol, 0.1 eq., 10% wt Pd) was added. The solution was evacuated and flushed with N_2 (× 3) and then flushed with H_2 and left to stir under an atmosphere of H_2 for 16 h. The reaction mixture was filtered through a pad of Celite and the Ceilte was washed with EtOAc (200 mL). The filtrate was concentrated *in vacuo*. The crude residue was taken up in DCM (6 mL) and the reaction mixture was cooled to 0 °C and TFA (5 mL) was added slowly. The reaction mixture was warmed to rt and stirred for 1 h, after which it was concentrated *in vacuo* and taken up in DCM (10 mL), then K_2CO_3 (2.22 g, 16.0 mmol, 5.0 eq.) was added and the reaction mixture stirred for 1 h. The reaction mixture was diluted with H_2O (30 mL) and the phases separated. The aqueous phase was extracted with DCM (2 × 25 mL) and the combined organic extracts were washed with brine (30 mL), dried over Na_2SO_4 and concentrated *in vacuo* to afford the title compound (536 mg, 2.83 mmol, 60%) as a yellow oil.

^{1}H NMR (400 MHz, $CDCl_3$) δ 7.35-7.13 (5H, m, ArH), 3.14-3.05 (1H, m, piperidinyl H_a-C6), 2.76-2.60 (3H, m, includes 2H, m, ethyl H_2-C2; and 1H, m, piperidinyl H_b-C6), 2.56-2.47 (1H, m, piperidinyl H_1-C2), 1.96 (1H, br. s, NH), 1.85-1.77 (1H, m, piperidinyl H_a-C5), 1.77-1.65 (3H, m, includes 2H, m, ethyl H_2-C1; and 1H, m, piperidinyl H_a-C3) 1.65-1.58 (1H, m, piperidinyl H_a-C4) 1.50-1.29 (2H, m, includes 1H, m, piperidinyl H_b-C4; and1H, m, piperidinyl H_b-C5), 1.22-1.08 (1H, m, piperidinyl H_b-C3);

^{13}C NMR (100 MHz, $CDCl_3$) δ 142.5 (C_q), 128.5 (ArC), 128.4 (ArC), 125.9 (ArC), 56.6 (piperidinyl C2), 47.2 (piperidinyl C6), 39.3 (ethyl C1), 33.0 (piperidinyl C3), 32.4 (ethyl C2), 26.7 (piperidinyl C4), 24.9 (piperidinyl C5);

IR ν_{max} (neat)/cm^{-1}: 3303 (N-H), 3061, 3025, 2925, 2852, 2798, 2738, 1602;

HRMS (ESI): $C_{13}H_{20}N$ [M+H$^+$]: calculated 190.1590, found 190.1587.

3-Phenylcyclohexan-1-one

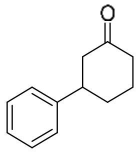

Following general procedure C, using 2-cyclohexene-1-one (0.73 mL, 7.5 mmol) and $PhB(OH)_2$ (1.37 g, 11.3 mmol). Purification by flash chromatography on silica gel, eluting with 20% EtOAc in hexane afforded the title compound (1.30 g, 7.46 mmol, 99%) as a colourless oil. The data is in accordance with the literature.[18]

^{1}H NMR (400 MHz, $CDCl_3$) δ 7.40-7.33 (2H, m, ArH), 7.30-7.22 (3H, m, ArH), 3.04 (1H, tt, *J* = 11.7, 3.9, cyclohexyl H-C3), 2.67-2.52 (2H, m, cyclohexyl H_2-C2), 2.52-2.36 (2H, m, cyclohexyl H_2-C6), 2.23-2.07 (2H, m, contains 1H, m, cyclohexyl H_a-C4; and 1H, m, cyclohexyl H_a-C5), 1.95-1.73 (2H, m, contains 1H, m, cyclohexyl H_b-C4; and 1H, m, cyclohexyl H_b-C5);

^{13}C NMR (100 MHz, $CDCl_3$) δ 211.1 (C_q), 144.5 (C_q), 128.8 (2 × C, ArC), 126.8 (ArC), 126.7 (2 × C, ArC), 49.0 (cyclohexyl C2), 44.8 (cyclohexyl C3), 41.3 (cyclohexyl C6), 32.9 (cyclohexyl C4), 25.6 (cyclohexyl C5);

IR ν_{max} (neat)/cm^{-1}: 3061, 3028, 2937, 2865, 1707 (C=O), 1603, 1496, 1450.

2-Benzylcyclohexan-1-one

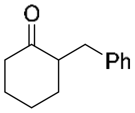

To a stirred solution of cyclohexanone (2.08 mL, 20.0 mmol, 1.0 eq.) in THF (20 mL) at -78 °C was added a solution of LiHMDS (20 mL of 1.0 M solution in THF, 1.0 eq.) dropwise. After 0.5 h a solution of benzyl bromide (2.62 mL, 20.0 mmol, 1.0 eq.) in THF (20 mL) was added via syringe pump at a rate of 1 drop per second, then the reaction mixture was warmed to rt and stirred for 4 h. After concentration *in vacuo* the residue was taken up in H_2O (40 mL) and extracted with EtOAc (3 × 30 mL) and the combined organic extracts were washed with brine (30 mL), dried over Na_2SO_4 and concentrated *in vacuo*. Purification by flash chromatography on silica gel, eluting with 50% DCM in hexane afforded the title compound (2.83 g, 15.0 mmol, 75%) as a colourless oil. The data is in accordance with the literature.[19]

^{1}H NMR (500 MHz, $CDCl_3$) δ 7.28 (2H, m, ArH), 7.22-7.11 (3H, m, ArH), 3.24 (1H, dd, *J* = 13.9, 4.8, benzyl H_a-C1*HH*), 2.60-2.50 (1H, m, cyclohexyl H-C2), 2.47-2.38 (2H, m, includes 1H, m, cyclohexyl H_a-C6, and 1H, dd, *J* = 13.8, 8.7, benzyl H_b-C1), 2.33 (1H, td, *J* = 12.9, 5.8, cyclohexyl H_b-C6), 2.12-1.97 (2H, m, includes 1H, m, cyclohexyl H_a-C3; and 1H, m, cyclohexyl H_a-C5), 1.88-1.78 (1H, m, cyclohexyl H_a-C4), 1.73-1.52 (2H, m, includes 1H, m, cyclohexyl H_b-C4; and 1H, m, cyclohexyl H_b-C5), 1.36 (1H, app. qd, *J* = 12.4, 3.6, cyclohexyl H_b-C3);

^{13}C NMR (75 MHz, $CDCl_3$) δ ppm 212.2 (C1), 140.1 (C_q), 128.9 (2 × C, ArC), 128.0 (2 × C, ArC), 125.7 (ArC), 52.2 (C2), 41.9 (C6), 35.2 (benzyl C1), 33.1 (C3), 27.8 (C5), 24.8 (C4);

IR ν_{max} (neat)/cm^{-1}: 3025, 2933, 2859, 1705 (C=O), 1495, 1448, 1312, 1127;

3-(2,6-Dimethylphenyl)-*N*-methylbutanamide

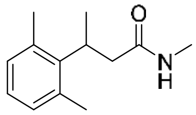

Following general procedure C, using *N*-methylcrotonamide (500 mg, 5.04 mmol) and 2,6-dimethylphenylboronic acid (1.13 g, 7.56 mmol). Purification by flash chromatography on silica gel, eluting with 50% EtOAc in hexane afforded the *title compound* (490 mg, 2.39 mmol, 47%) as a colourless gum.

^{1}H NMR (500 MHz, $CDCl_3$) δ 6.98 (3H, s, ArH), 5.33 (1H, br. s, NH), 3.87 (1H, app. hp, *J* = 7.3, butyl H-C3), 2.73 (3H, d, *J* = 4.8, NCH_3), 2.56 (2H, d, *J* = 7.3, butyl H_2-C2), 2.40 (6H, s, $ArCH_3$), 1.36 (3H, d, *J* = 7.3, butyl H_3-C4);

^{13}C NMR (125 MHz, $CDCl_3$) δ 172.9 (butyl C1), 141.8 (ArC), 135.8 (C_q), 130.3 (C_q), 128.5 (C_q), 126.0 (2 × C, ArC), 42.3 (butyl C2), 31.9 (butyl C3), 26.2 (NCH_3), 21.5 (2 × C, $ArCH_3$), 18.9 (butyl C4);

IR ν_{max} (neat)/cm^{-1}: 3267 (N-H), 3087, 2961, 2876, 1637 (C=O), 1570, 1462, 1411;

HRMS (ESI): $C_{13}H_{20}NO$ [M+H$^+$]: calculated 206.1539, found 206.1539.

[3-(2,6-Dimethylphenyl)butyl](methyl)amine

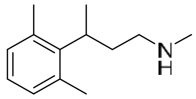

Following general procedure F, using 3-(2,6-dimethylphenyl)-N-methylbutanamide (375 mg, 1.83 mmol). Purification by SCX cartridge afforded the *title compound* (283 mg, 1.48 mmol, 81%) as a pale yellow oil.

^{1}H NMR (500 MHz, $CDCl_3$) δ 6.97 (3H, s, ArH), 3.37-3.29 (1H, m, butyl H-C3), 2.60-2.53 (1H, m, butyl H_a-C1), 2.52-2.30 (10H, m, includes 3H, s, NCH_3; 6H, br. m, $ArCH_3$ and 1H, m, butyl H_b-C1), 2.06-1.95 (1H, m, butyl H_a-C2), 1.93-1.83 (1H, m, butyl H_b-C2), 1.32 (3H, d, *J* = 7.3, butyl H_3-C4);

^{13}C NMR (125 MHz, $CDCl_3$) δ 142.7 (ArC), 136.2 (C_q), 130.4 (C_q), 128.3 (C_q), 125.6 (2 × C, ArC), 51.2 (butyl C1), 36.5 (butyl C2), 35.6 (N*C*H_3), 33.0 (butyl C3), 21.6 (2 × C, $ArCH_3$), 19.1 (butyl C4);

IR ν_{max} (neat)/cm^{-1}: 3017, 2957, 2872, 2788, 1544, 1467, 1380, 1308;

HRMS (ESI): $C_{13}H_{22}N$ [M+H$^+$]: calculated 192.1747, found 192.1745.

2-(But-3-en-1-yl)-1,3-dichlorobenzene

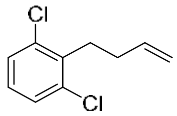

Following general procedure J, using 2,6-dichlorobenzyl bromide (1.00 g, 4.18 mmol) and allylmagnesium chloride (2.30 mL of a 2.0 M solution in THF) afforded the title compound (827 mg, 4.11 mmol, 98%) as a colourless oil.

^{1}H NMR (400 MHz, $CDCl_3$) δ 7.29-7.22 (2H, m, ArH), 7.10-6.99 (1H, m, ArH), 5.96 (1H, ddt, *J* = 16.9, 10.2, 6.7, butenyl H-C3), 5.07 (1H, dd, *J* = 16.9, 1.1, butenyl H_{trans}-C4) 5.04-4.97 (1H, m, butenyl H_{cis}-C4), 3.06-2.95 (2H, m, butenyl H_2-C1), 2.38-2.26 (2H, m, butenyl H_2-C2);

^{13}C NMR (100 MHz, $CDCl_3$) δ 137.9 (C_q), 137.6 (butenyl C3), 135.5 (2 × C, C_q), 128.3 (2 × C, ArC), 127.7 (ArC), 115.3 (butenyl C4), 32.3 (butenyl C3), 30.9 (butenyl C1);

IR ν_{max} (neat)/cm^{-1}: 3078, 2978, 2942, 2872, 1641, 1582, 1561, 1490.

3-(2,6-Dichlorophenyl)propanal

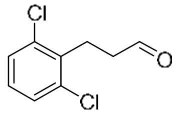

Following general procedure L, using 2-(but-3-en-1-yl)-1,3-dichlorobenzene (827 mg, 4.11 mmol). Purification by flash chromatography on silica gel, eluting with 10% Et_2O in hexane afforded the *title compound* (528 mg, 2.60 mmol, 63%) as a colourless oil.

^{1}H NMR (400 MHz, $CDCl_3$) δ 9.86 (1H, s, propyl H-C1), 7.32-7.26 (2H, m, ArH), 7.14-7.06 (1H, m, ArH), 3.26 (2H, t, *J* = 8.1, propyl H_2-C3), 2.73 (2H, t, *J* = 8.1, propyl H_2-C2);

^{13}C NMR (100 MHz, $CDCl_3$) δ 200.9 (propyl C1), 136.4 (C_q), 135.4 (2 × C, C_q), 128.4 (2 × C, ArC), 128.3 (ArC), 42.1 (propyl C2), 24.2 (propyl C3);

IR ν_{max} (neat)/cm^{-1}: 2953, 2857, 1699 (C=O), 1582, 1561, 1433, 1409, 1395.

[3-(2,6-Dichlorophenyl)propyl](methyl)amine

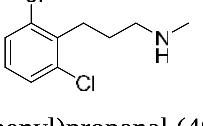

To a stirred solution of 3-(2,6-dichlorophenyl)propanal (400 mg, 1.97 mmol, 1.0 eq.) in DCM (10 mL) was added a solution of $MeNH_2$ (3.5 mL of an 8.0 M solution in EtOH, 10 eq.). This was stirred for 0.5 h then cooled to 0 °C and sodium trisacetoxyborohydride (835 mg, 3.94 mmol, 2.0 eq.) was added portionwise. The reaction mixture was stirred for 4 h then the reaction was quenched with H_2O (20 mL). The organic phase was separated and the aqueous phase was extracted with DCM (1 × 20 mL) and the combined organic extracts were then washed with brine,

dried over Na_2SO_4 and concentrated *in vacuo*. Due to the presence of residual imine the residue was re-dissolved in MeOH (10 mL) and $NaBH_4$ (149 mg, 3.94 mmol, 2.0 eq.) was added portionwise. After 20 minutes the reaction mixture was diluted with H_2O (30 mL) and then extracted with EtOAc (3 × 20 mL). The combined organic extracts were washed with brine, dried over Na_2SO_4 and concentrated *in vacuo*. Purification by SCX cartridge afforded the *title compound* (319 mg, 1.46 mmol, 74%) as a pale yellow oil.

^{1}H NMR (400 MHz, $CDCl_3$) δ 7.28-7.22 (2H, m, ArH), 7.08-7.00 (1H, m, ArH), 2.99-2.92 (2H, m, propyl H_2-C1), 2.70 (2H, t, J = 7.2, propyl H_2-C3), 2.46 (3H, s, NCH_3), 1.84-1.74 (2H, m, propyl H_2-C2);

^{13}C NMR (100 MHz, $CDCl_3$) δ 138.1 (C_q), 135.4 (2 × C, C_q), 128.3 (2 × C, ArC), 127.7 (ArC), 51.6 (propyl C1), 36.3 (NCH_3), 29.2 (propyl C3), 28.1 (propyl C2);

IR ν_{max} (neat)/cm^{-1}: 3283, 3057, 2933, 2870, 2790, 1582, 1561, 1434;

HRMS (ESI): $C_{10}H_{14}{}^{35}Cl_2N$ [M+H$^+$]: calculated 218.0498, found 218.0493.

1.3 *N*-Chloroamine data

N-Chloro-*N*-methyl-3-phenylpropan-1-amine 1a

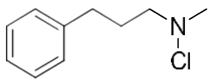

Following general procedure B, using *N*-methyl-3-phenylpropan-1-amine (500 mg, 3.35 mmol). Purification by flash chromatography on silica gel, eluting with 10% EtOAc in hexane afforded the *title compound* **1a** (565 mg, 3.08 mmol, 92%) as a colourless oil.

^{1}H NMR (300 MHz, $CDCl_3$) δ 7.32-7.25 (2H, m, ArH), 7.22-7.14 (3H, m, ArH), 2.93 (3H, s, NCH_3), 2.88 (2H, t, J = 6.9, propyl H_2-C1), 2.68 (2H, t, J = 7.7, propyl H_2-C3), 2.03-1.90 (2H, m, propyl H_2-C2);

^{13}C NMR (75 MHz, $CDCl_3$) δ 141.7 (C_q), 128.4 (2 × C, ArC), 128.4 (2 × C, ArC), 125.9 (ArC), 65.2 (propyl C1), 53.0 (NCH_3), 32.7 (propyl C3), 29.7 (propyl C2);

IR ν_{max} (neat)/cm^{-1}: 3027, 2949, 2866, 1603, 1496, 1454, 1439, 1172;

HRMS data could not be obtained.

N-Benzyl-*N*-chloro-3-phenylpropan-1-amine 1b

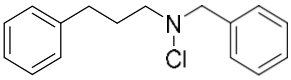

Following general procedure B, using *N*-benzyl-3-phenylpropan-1-amine (500 mg, 2.22 mmol). Purification by flash chromatography on silica gel, eluting with 10% EtOAc afforded the *title compound* **1b** (471 mg, 1.81 mmol, 82%) as a colourless oil.

^{1}H NMR (300 MHz, $CDCl_3$) δ 7.48-7.05 (10H, m, ArH), 4.12 (2H, s, NCH_2Ph), 2.99 (2H, t, *J* = 6.7, propyl H_2-C1), 2.71 (2H, t, *J* = 7.5, propyl H_2-C3), 2.12-2.00 (2H, m, propyl H_2-C2);

^{13}C NMR (75 MHz, $CDCl_3$) δ 141.8 (C_q), 137.1 (C_q), 129.2 (2 × C, ArC), 128.5 (2 × C, ArC), 128.4 (2 × C, ArC), 128.3 (2 × C, ArC), 127.8 (ArC), 125.8 (ArC), 68.4 (NCH_2Ph), 62.1 (propyl C1), 32.6 (propyl C3), 29.4 (propyl C2);

IR ν_{max} (neat)/cm^{-1}: 3027, 2946, 2838, 1602, 1495, 1453, 1101, 1029;

HRMS (ESI): $C_{16}H_{19}{}^{35}ClN$ [M+H$^+$]: calculated 260.1201, found 260.1201.

N-Chloro-*N*-(3-phenylpropyl)butan-1-amine 1c

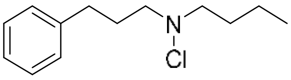

Following general procedure B, using *N*-(3-phenylpropyl)butan-1-amine (500 mg, 2.61 mmol). Purification by flash chromatography on silica gel, eluting with 10% EtOAc in hexane afforded the *title compound* **1c** (429 mg, 1.90 mmol, 73%) as a yellow oil.

^{1}H NMR (300 MHz, $CDCl_3$) δ 7.35-7.26 (2H, m, ArH), 7.26-7.16 (3H, m, ArH), 2.98-2.89 (4H, m, includes 2H, m, propyl H_2-C1; and 2H, m, butyl H_2-C1), 2.71 (2H, t, J = 7.6, propyl H_2-C3), 2.09-1.97 (2H, m, propyl H_2-C2), 1.73-1.60 (2H, m, butyl H_2-C2), 1.47-1.32 (2H, m, butyl H_2-C3), 0.95 (2H, t, *J* = 7.3, butyl H_3-C4);

^{13}C NMR (75 MHz, $CDCl_3$) δ 141.8 (C_q), 128.5 (2 × C, ArC), 128.3 (2 × C, ArC), 125.8 (ArC), 64.1 (butyl C1), 63.3 (propyl C1), 32.8 (propyl C3), 30.0 (butyl C2), 29.5 (propyl C2), 20.0 (butyl C3), 13.9 (butyl C4);

IR ν_{max} (neat)/cm^{-1}: 3027, 2955, 2864, 2835, 1496, 1453, 745, 697;

HRMS (ESI): $C_{13}H_{21}{}^{35}ClN$ [M+H$^+$]: calculated 226.1357, found 226.1358.

N-Chloro-*N*-(3-phenylpropyl)hexan-1-amine 1d

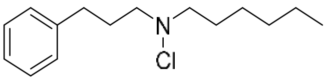

Following general procedure B, using *N*-(3-phenylpropyl)hexan-1-amine (225 mg, 1.03 mmol). Purification by flash chromatography on silica gel, eluting with 10% EtOAc in pentane afforded the *title compound* **1d** (209 mg, 0.83 mmol, 81%) as a colourless oil.

^{1}H NMR (300 MHz, $CDCl_3$) δ 7.30 (2H, m, ArH), 7.25-7.13 (3H, m, ArH), 2.92 (4H, m, includes 2H, m, propyl H_2-C1; 2H, m, hexyl H_2-C1), 2.75-2.61 (2H, m, propyl H_2-C3), 2.09-1.95 (2H, m, propyl H_2-C2), 1.75-1.60 (2H, m, hexyl H_2-C2), 1.43-1.22 (6H, m, hexyl H_2-C3-5), 0.91 (3H, t, *J* = 6.8, hexyl H_3-C6);

^{13}C NMR (75 MHz, $CDCl_3$) δ 142.0 (C_q), 128.6 (2 × C, ArC), 128.5 (2 × C, ArC), 126.0 (ArC), 64.6 (CH_2), 63.4 (CH_2), 32.9 (CH_2), 31.8 (CH_2), 29.6 (CH_2), 28.0 (CH_2), 26.7 (CH_2), 22.7 (CH_2), 14.2 (CH_3);

IR ν_{max} (neat)/cm^{-1}: 3085, 3027, 2928, 1063, 1496, 1454, 1347, 1302;

HRMS (ESI): $C_{15}H_{25}^{35}ClN$ [M+H$^+$]: calculated 254.1670, found 254.1675.

N-Chloro-*N*-(3-phenylpropyl)prop-2-en-1-amine 1e

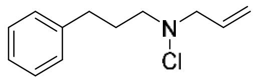

Following general procedure B, using *N*-(3-phenylpropyl)prop-2-en-1-amine (500 mg, 2.85 mmol). Purification by flash chromatography on silica gel, eluting with 10% EtOAC in hexane afforded the *title compound* **1e** (523 mg, 2.49 mmol, 87%) as a pale yellow oil.

^{1}H NMR (500 MHz, $CDCl_3$) δ 7.34-7.27 (2H, m, ArH), 7.24-7.17 (3H, m, ArH), 6.02-5.91 (1H, m, propenyl H-C2), 5.32-5.22 (2H, m, propenyl H_2-C3), 3.61 (2H, dd, *J* = 6.4, 0.9, propenyl H_2-C1), 2.94 (2H, t, *J* = 6.9, propyl H_2-C1), 2.70 (2H, t, *J* = 7.7, propyl H_2-C3), 2.07-1.97 (2H, m, propyl H_2-C2);

^{13}C NMR (125 MHz, $CDCl_3$) δ 141.7 (propenyl C2), 133.6 (C_q), 128.4 (2 × C, ArC), 128.3 (2 × C, ArC), 125.8 (ArC), 119.2 (propenyl C3), 66.9 (propenyl C1), 62.1 (propyl C1), 32.7 (propyl C3), 29.4 (propyl C2);

IR ν_{max} (neat)/cm^{-1}: 3084, 3063, 3026, 2948, 2840, 1645, 1603, 1496;

HRMS (ESI): $C_{12}H_{17}^{35}ClN$ [M+H$^+$]: calculated 210.1044, found 210.1039.

N-Chloro-*N*-methyl-4-phenylbutan-2-amine 1f

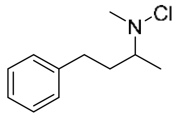

Following general procedure B, using *N*-methyl-4-phenylbutan-2-amine (500 mg, 3.06 mmol). Purification by flash chromatography on silica gel, eluting with 10% EtOAc in hexane afforded the *title compound* **1f** (350 mg, 1.77 mmol, 58%) as a pale yellow oil.

^{1}H NMR (500 MHz, $CDCl_3$) δ 7.31-7.26 (2H, m, ArH), 7.23-7.16 (3H, m, ArH), 2.93-2.84 (4H, m, includes 3H, s, NCH_3; and 1H, m, butyl H-C2), 2.73-2.66 (2H, m, butyl H_2-C4), 1.98 (1H, ddt, *J* = 13.6, 8.8, 6.8, butyl H_a-C3), 1.73-1.63 (1H, m, butyl H_b-C3), 1.16 (3H, d, *J* = 6.3, butyl H_3-C1);

^{13}C NMR (125 MHz, $CDCl_3$) δ 142.1 (C_q), 128.4 (2 × C, ArC), 128.3 (2 × C, ArC), 125.8 (ArC), 64.5 (butyl C2), 48.1 (NCH_3), 36.3 (butyl C3), 32.3 (butyl C4), 14.2 (butyl C1);

IR ν_{max} (neat)/cm^{-1}: 3026, 2973, 2947, 2863, 1603, 1495, 1453, 1433;

HRMS (ESI): $C_{11}H_{17}{}^{35}ClN$ [M+H$^+$]: calculated 198.1044, found 198.1938.

Benzyl(chloro)(4-phenylbutan-2-yl)amine 1g

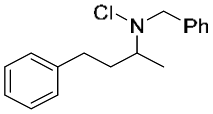

Following general procedure B, using benzyl(4-phenylbutan-2-yl)amine (500 mg, 2.09 mmol). Purification by flash chromatography on silica gel, eluting with 10% EtOAc in hexane afforded the *title compound* **1g** (554 mg, 2.02 mmol, 97%) as a colourless oil.

^{1}H NMR (400 MHz, $CDCl_3$) δ 7.42-7.14 (10H, m, ArH), 4.15 (1H, d, *J* = 13.7, NCH_aH$_b$), 4.03 (1H, d, *J* = 13.7, NCH$_a$$H_b$), 3.12-3.00 (1H, m, butyl H-C2), 2.86-2.74 (1H, m, butyl H$_a$-C4), 2.74-2.65 (1H, m, butyl H$_b$-C4), 2.15-2.02 (1H, m, butyl H$_a$-C3), 1.78-1.65 (1H, m, butyl H$_b$-C3), 1.24 (3H, d, *J* = 6.2, butyl H$_3$-C1);

^{13}C NMR (100 MHz, $CDCl_3$) δ 142.4 (C$_q$), 137.9 (C$_q$), 129.0 (2 × C, ArC), 128.7 (2 × C, ArC), 128.51 (2 × C, ArC), 128.48 (2 × C, ArC), 127.8 (ArC), 125.9 (ArC), 64.0 (NCH_2), 62.3 (butyl C2), 36.6 (butyl C3), 32.6 (butyl C4), 14.5 (butyl C1);

IR ν_{max} (neat) / cm^{-1}: 3086, 3062, 3027, 2970, 2931, 2860, 1603, 1495;

HRMS (ESI): $C_{17}H_{21}{}^{35}ClN$ [M+H$^+$]: calculated 274.1357, found 274.1353.

N-Chloro-*N*-methyl-1,3-diphenylpropan-1-amine 1h

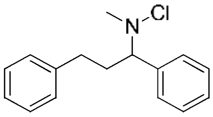

Following general procedure B, using *N*-methyl-1,3-diphenylpropan-1-amine (350 mg, 1.55 mmol). Purification by flash chromatography on silica gel, eluting with 10% EtOAc in hexane afforded the *title compound* **1h** (386 mg, 1.49 mmol, 96%) as a pale yellow gum.

^{1}H NMR (500 MHz, $CDCl_3$) δ 7.45-7.34 (5H, m, ArH), 7.34-7.27 (2H, m, ArH), 7.25-7.15 (3H, m, ArH), 3.76 (1H, dd, *J* = 8.7, 4.4, propyl H-C1), 2.79 (3H, s, NCH_3), 2.61-2.50 (3H, m, includes 2H, m, propyl H$_2$-C3; and 1H, m, propyl H$_a$-C2), 2.25-2.13 (1H, m, propyl H$_b$-C2);

^{13}C NMR (125 MHz, $CDCl_3$) δ 141.6 (C$_q$), 138.5 (C$_q$), 128.9 (2 × C, ArC), 128.4 (2 × C, ArC), 128.3 (2 × C, ArC), 128.3 (2 × C, ArC), 128.1 (ArC), 125.9 (ArC), 75.2 (propyl C1), 49.7 (NCH_3), 35.6 (propyl C2), 32.3 (propyl C3);

IR ν_{max} (neat)/cm^{-1}: 3061, 3027, 2953, 2858, 1602, 1583, 1494, 1452;

HRMS (ESI): $C_{16}H_{19}{}^{35}ClN$ [M+H$^+$]: calculated 260.1201, found 260.1197.

N-Chloro-*N*-methyl-5-phenylpent-1-en-3-amine 1i

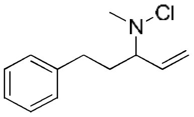

Following general procedure I, using 2-methyl-*N*-(5-phenylpent-1-en-3-yl)propane-2-sulfinamide (2.00 g, 7.54 mmol) afforded the desired amine as an inseparable mixture with an unidentified impurity. This was carried forward to the chlorination step as a crude mixture. The crude gum (824 mg) was taken up in DCM (10 mL) and NCS (628 mg, 4.70 mmol, 1 eq.) was added. The reaction mixture was stirred for 3 h, then concentrated *in vacuo* and purified by flash chromatography on silica gel, eluting with 30% DCM in hexane to afford the *title compound* **1i** (791 mg, 3.77 mmol, 50%) as a colourless oil.

^{1}H NMR (500 MHz, $CDCl_3$) δ 7.33-7.27 (2H, m, ArH), 7.24-7.18 (3H, m, ArH), 5.94-5.84 (1H, m, pentenyl H-C2), 5.37 (1H, d, *J* = 10.3, pentenyl H_a-C1), 5.23-5.16 (1H, m, pentenyl H_b-C1), 3.13 (1H, dd, *J* = 14.6, 7.4, pentenyl H-C3), 2.87 (3H, s, NCH_3), 2.75-2.64 (2H, m, pentenyl H_2-C5), 2.18-2.09 (1H, m, pentenyl H_a-C4), 1.92-1.81 (1H, m, pentenyl H_b-4);

^{13}C NMR (125 MHz, $CDCl_3$) δ 141.8 (C_q), 135.1 (pentenyl C2), 128.5 (2 × C, ArC), 128.3 (2 × C, ArC), 125.8 (ArC), 119.9 (pentenyl C1), 72.8 (pentenyl C3), 49.2 (NCH_3), 34.9 (pentenyl C4), 31.9 (pentenyl C5);

IR ν_{max} (neat)/cm^{-1}: 3063, 3027, 2949, 2923, 2882, 2859, 1639, 1496;

HRMS (ESI): $C_{12}H_{17}{}^{35}ClN$ [M+H$^+$]: calculated 210.1044, found 210.1039.

N-chloro(methyl)(1-phenyloctan-3-yl)amine 1j

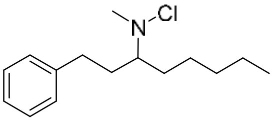

Following general procedure A, using amine (500 mg, 2.28 mmol). Purification by flash chromatography on silica gel, eluting with 10% EtOAc in hexane afforded the *title compound* **1j** (432 mg, 1.70 mmol, 75%) as a pale yellow oil.

^{1}H NMR (400 MHz, $CDCl_3$) δ 7.34-7.27 (2H, m, ArH), 7.25-7.17 (3H, m, ArH), 2.89 (3H, s, NCH_3), 2.79-2.62 (3H, m, includes 1H, m, octyl H_1-C3; and 2H, m, octyl H_2-C1), 2.01-1.89 (1H, m, octyl H_a-C2), 1.81-1.66 (2H, m, includes 1H, m, octyl H_b-C2; and 1H, m, octyl H_a-C4), 1.51-1.39 (1H, m, octyl H_b-C4), 1.39-1.24 (6H, m, octyl H_2-C5-7), 0.91 (3H, t, *J* = 6.9, octyl H_3-C8);

^{13}C NMR (100 MHz, $CDCl_3$) δ 142.4 (C_q), 128.6 (2 × C, ArC2), 128.5 (2 × C, ArC3), 125.9 (ArC4), 69.5 (octyl C3), 48.0 (NCH_3), 32.90 (octyl C2), 32.86 (octyl C1), 32.1 (CH_2), 30.0 (octyl C4), 26.5 (CH_2), 22.7 (CH_2), 14.2 (octyl C8);

IR ν_{max} (neat)/cm^{-1}: 3062, 3026, 2930, 2858, 1603, 1495, 1454, 1433;

HRMS (ESI): $C_{15}H_{25}^{35}ClN$ [M+H$^+$]: calculated 254.1670, found 254.1663.

N-Chloro(2-methoxy-3-phenylpropyl)methylamine 1k

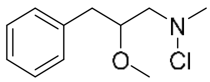

Following general procedure B, using (2-methoxy-3-phenylpropyl)(methyl)amine (150 mg, 0.84 mmol). Purification by flash chromatography on silica gel, eluting with 10% EtOAc in hexane afforded the *title compound* **1k** (179 mg, 0.84 mmol, quant.) as a colourless oil.

^{1}H NMR (400 MHz, $CDCl_3$) δ 7.37-7.20 (5H, m, ArH), 3.82-3.73 (1H, m, propyl H-C2), 3.43 (3H, s, OCH_3), 3.04-2.95 (4H, m, includes 3H, s, NCH_3; and 1H, m, propyl H_a-C1), 2.94-2.80 (3H, m, includes 2H, m, propyl H_2-C3; and 1H, m, propyl H_b-C1);

^{13}C NMR (100 MHz, $CDCl_3$) δ 138.3 (C_q), 129.7 (2 × C, ArC), 128.4 (2 × C, ArC), 126.4 (ArC), 80.0 (propyl C2), 69.1 (propyl C1), 58.0 (OCH_3), 53.9 (NCH_3), 38.4 (propyl C3);

IR ν_{max} (neat)/cm^{-1}: 3062, 3028, 2927, 2885, 2828, 1681, 1603, 1495;

HRMS (ESI): $C_{11}H_{16}^{35}ClNONa$ [M+Na$^+$]: calculated 236.0813, found 236.0806.

N-Chloro(methyl)(2-methyl-3-phenylpropyl)amine 1l

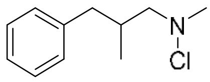

Following general procedure B, using methyl(2-methyl-3-phenylpropyl)amine (200 mg, 1.23 mmol). Purification by flash chromatography on silica gel, eluting with 10% EtOAc in hexane afforded the *title compound* **1l** (198 mg, 1.00 mmol, 81%) as a pale yellow oil.

^{1}H NMR (500 MHz, $CDCl_3$) δ 7.31-7.25 (2H, m, ArH), 7.22-7.15 (3H, m, ArH), 2.95 (3H, s, NCH_3), 2.83 (1H, dd, J = 13.4, 5.0, propyl H_a-C3), 2.76 (1H, dd, J = 12.7, 7.0, propyl H_a-C1), 2.67 (1H, dd, J = 12.7, 7.0, propyl H_b-C1), 2.38 (1H, dd, J = 13.4, 8.6, propyl H_b-C3), 2.24--2.15 (1H, m, propyl H-C2), 0.90 (3H, d, J = 6.7, CH_3);

^{13}C NMR (125 MHz, $CDCl_3$) δ 140.5 (C_q), 129.5 (2 × C, ArC), 128.3 (2 × C, ArC), 126.0 (ArC), 72.1 (propyl C1), 53.5 (NCH_3), 40.7 (propyl C3), 34.1 (propyl C2), 17.4 (CH_3);

IR ν_{max} (neat)/cm^{-1}: 3062, 3027, 2954, 2922, 2873, 1602, 1495, 1454;

HRMS data could not be obtained.

N-Chloro-*N*-methyl-3-phenylbutan-1-amine 1m

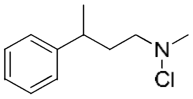

Following general procedure B, using *N*-methyl-3-phenylbutan-1-amine (250 mg, 1.53 mmol). Purification by flash chromatography on silica gel, eluting with 10% EtOAc in hexane afforded the *title compound* **1m** (230 mg, 1.16 mmol, 76%) as a pale yellow oil.

^{1}H NMR (300 MHz, $CDCl_3$) δ 7.35-7.27 (2H, m, ArH), 7.23-7.15 (3H, m, ArH), 2.87 (2H, s, NCH_3), 2.85-2.71 (3H, m, includes 1H, m, butyl H-C3; and 2H, m, butyl H_2-C1), 2.00-1.86 (2H, m, butyl H_2-C2), 1.28 (3H, d, *J* = 7.0, butyl H_3-C4);

^{13}C NMR (75 MHz, $CDCl_3$) δ 146.7 (C_q), 128.5 (2 × C, ArC), 127.0 (2 × C, ArC), 126.1 (ArC), 64.3 (butyl C1), 53.1 (NCH_3), 37.3 (butyl C3), 36.5 (butyl C2), 22.5 (butyl C4);

IR ν_{max} (neat)/cm^{-1}: 3027, 2958, 2871, 1602, 1493, 1452, 1374, 761;

HRMS (ESI): $C_{11}H_{17}{}^{35}ClN$ [M+H$^+$]: calculated 198.1044, found 198.1040.

N-Chloro-*N*-methyl-3,3-diphenylpropan-1-amine 1n

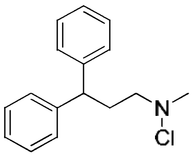

Following general procedure B, using *N*-methyl-3,3-diphenylpropan-1-amine (500 mg, 2.22 mmol). Purification by flash chromatography on silica gel, eluting with 10% EtOAc in pentane afforded the *title compound* **1n** (269 mg, 1.04 mmol, 47%) as a colourless gum.

^{1}H NMR (300 MHz, $CDCl_3$) δ 7.34-7.23 (8H, m, ArH), 7.21-7.14 (2H, m, ArH), 4.08 (1H, t, *J* = 7.9, propyl H-C3), 2.88 (3H, s, NCH_3) 2.81 (2H, t, *J* = 6.8, propyl H_2-C1), 2.46-2.36 (2H, m, propyl H_2-C2);

^{13}C NMR (75 MHz, $CDCl_3$) δ 144.4 (2 × C, C_q), 128.5 (4 × C, ArC), 127.9 (4 × C, ArC), 126.3 (2 × C, ArC), 64.2 (propyl C1), 53.2 (NCH_3), 48.1 (propyl C3), 34.0 (propyl C2);

IR ν_{max} (neat)/cm^{-1}: 3062, 3025, 2938, 1595, 1492, 1451, 777, 747;

HRMS (ESI): $C_{16}H_{19}{}^{35}ClN$ [M+H$^+$]: calculated 260.1201, found 260.1203.

N-Chloro(methyl)[3-(4-phenylphenyl)propyl]amine 1o

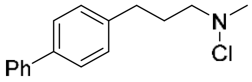

Following general procedure B, using *N*-methyl[3-(4-phenylphenyl)propyl]amine (350 mg, 1.55 mmol). Purification by flash chromatography on silica gel, eluting with 10% EtOAc in hexane afforded the *title compound* **1o** (302 mg, 1.16 mmol, 75%) as a pale yellow oil.

^{1}H NMR (400 MHz, $CDCl_3$) δ 7.63-7.22 (9H, m, ArH), 2.96 (3H, s, NCH_3), 2.95-2.90 (2H, m, propyl H_2-C1), 2.77-2.71 (2H, m, propyl H_2-C3), 2.07-1.97 (2H, m, propyl H_2-C2);

^{13}C NMR (100 MHz, $CDCl_3$) δ 141.2 (C_q), 140.9 (C_q), 139.0 (C_q), 129.0 (2 × C, ArC), 128.9 (2 × C, ArC), 127.3 (2 × C, ArC), 127.2 (ArC), 127.1 (2 × C, ArC), 65.4 (propyl C1), 53.2 (NCH_3), 32.5 (propyl C3), 29.8 (propyl C2);

IR ν_{max} (neat)/cm^{-1}: 3057, 3029, 2997, 2863, 1595, 1487, 1455, 1439;

HRMS (ESI): $C_{16}H_{19}^{35}ClN$ [M+H$^+$]: calculated 260.1201, found 260.1201.

N-Chloro-*N*-methyl-3-(*p*-tolyl)butan-1-amine 1p

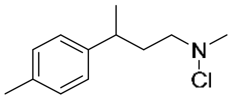

Following general procedure B, using *N*-methyl-3-(p-tolyl)butan-1-amine (250 mg, 1.41 mmol). Purification by flash chromatography on silica gel, eluting with 10% EtOAc in hexane afforded the *title compound* **1p** (227 mg, 1.07 mmol, 76%) as a colourless gum.

^{1}H NMR (500 MHz, $CDCl_3$) δ 7.15-7.08 (4H, m, ArH), 2.89 (3H, s, NCH_3), 2.85-2.71 (3H, m, includes 2H, m, butyl H_2-C1; and 1H, m, butyl H-C3), 2.34 (3H, s, $ArCH_3$), 2.00-1.85 (2H, m, butyl H_2-C2), 1.28 (3H, d, *J* = 7.0, butyl H_3-C4);

^{13}C NMR (125 MHz, $CDCl_3$) δ 143.6 (C_q), 135.5 (C_q), 129.1 (2 × C, ArC), 126.8 (2 × C, ArC), 64.4 (butyl C1), 53.1 (NCH_3), 36.8 (butyl C3), 36.5 (butyl C2), 22.6 (butyl C4), 21.0 ($ArCH_3$);

IR ν_{max} (neat)/cm^{-1}: 2956, 2924, 2870, 1514, 1455, 1438, 1374, 1349;

HRMS (ESI): $C_{12}H_{19}^{35}ClN$ [M+H$^+$]: calculated 212.1201, found 212.1194.

N-Chloro[3-(4-trifluoromethylphenyl)propyl]methylamine 1q

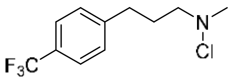

Following general procedure B, using [3-(4-trifluoromethylphenyl)propyl](methyl)amine (150 mg, 0.69 mmol). Purification by flash chromatography on silica gel, eluting with 10% EtOAc in hexane afforded the *title compound* **1q** (148 mg, 0.59 mmol, 86%) as a colourless oil.

^{1}H NMR (400 MHz, $CDCl_3$) δ 7.54 (2H, d, *J* = 8.1, ArH), 7.31 (2H, d, *J* = 8.1, ArH), 2.94 (3H, s, NCH_3), 2.87 (2H, t, *J* = 6.8, propyl H_2-C1), 2.75 (2H, t, *J* = 7.7, propyl H_2-C3), 2.02-1.94 (2H, m, propyl H_2-C2);

^{13}C NMR (100 MHz, $CDCl_3$) 146.0 (C_q), 128.9 (2 × C, Ar C2), 128.47 (d, *J* = 32.3, C_q), 125.5 (2 × C, q, *J* = 3.7, Ar C3), 124.5 (q, *J* = 271.8, CF_3), 65.0 (propyl C1), 53.2 (NCH_3), 32.6 (propyl C3), 29.6 (propyl C1);

IR ν_{max} (neat) / cm^{-1}: 2953, 2872, 2798, 1619, 1440, 1418, 1322, 1244;

HRMS could not be obtained.

N-Chloro-*N*-methyl-3-(*o*-tolyl)butan-1-amine 1r

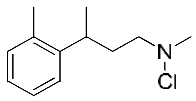

Following general procedure B, using *N*-methyl-3-(o-tolyl)butan-1-amine (200 mg, 1.13 mmol). Purification by flash chromatography on silica gel, eluting with 10% EtOAc in hexane afforded the *title compound* **1r** (187 mg, 0.88 mmol, 77%) as a colourless oil.

^{1}H NMR (300 MHz, $CDCl_3$) δ 7.24-7.04 (4H, m, ArH), 3.18-3.05 (1H, m, butyl H-C3), 2.88 (3H, s, NCH_3), 2.78 (2H, t, *J* = 7.1, butyl H_2-C1), 2.34 (3H, s, $ArCH_3$), 2.01-1.87 (2H, m, butyl H_2-C2), 1.24 (3H, d, *J* = 6.9, butyl H_3-C4);

^{13}C NMR (75 MHz, $CDCl_3$) δ 144.9 (C_q), 135.5 (C_q), 130.3 (ArC), 126.3 (ArC), 125.7 (ArC), 125.2 (ArC), 64.3 (butyl C1), 53.1 (NCH_3), 36.0 (butyl C2), 31.8 (butyl C3), 21.8 (butyl C4), 19.5 ($ArCH_3$);

IR ν_{max} (neat)/cm^{-1}: 3063, 3018, 2959, 2869, 1490, 1458, 1438, 1375;

HRMS (ESI): $C_{12}H_{19}{}^{35}ClN$ [M+H$^+$]: calculated 212.1201, found 212.1196.

N-Chloro[3-(4-chlorophenyl)propyl]methylamine 1s

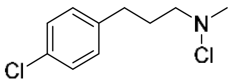

Following general procedure B, using [3-(4-chlorophenyl)propyl](methyl)amine (200 mg, 1.09 mmol). Purification by flash chromatography on silica gel, eluting with 10% EtOAc in hexane afforded the *title compound* **1s** (160 mg, 0.73 mmol, 67%) as a colourless oil.

^{1}H NMR (400 MHz, $CDCl_3$) δ 7.25 (2H, d, *J* = 8.3, ArH), 7.12 (2H, d, *J* = 8.3, ArH), 2.92 (3H, s, NCH_3), 2.85 (2H, t, *J* = 6.8, propyl H_2-C1), 2.64 (2H, t, *J* = 7.6, propyl H_2-C3), 1.98-1.90 (2H, m, propyl H_2-C2);

^{13}C NMR (100 MHz, $CDCl_3$) δ 140.1 (C_q), 131.6 (C_q), 129.8 (2 × C, ArC), 128.5 (2 × C, ArC), 65.0 (propyl C1), 53.1 (NCH_3), 32.0 (propyl C3), 29.6 (propyl C2);

IR ν_{max} (neat) / cm^{-1}: 2950, 2866, 1491, 1455, 1437, 1407, 1365, 1129;

HRMS could not be obtained.

3-(4-Bromophenyl)-*N*-chloro-*N*-methylbutan-1-amine 1t

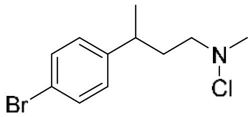

Following general procedure C, using 3-(4-bromophenyl)-*N*-methylbutan-1-amine (250 mg, 1.03 mmol). Purification by flash chromatography on silica gel, eluting with 10% EtOAc in hexane afforded the *title compound* **1t** (251 mg, 0.91 mmol, 88%) as a pale yellow gum.

^{1}H NMR (500 MHz, $CDCl_3$) δ 7.41 (2H, d, *J* = 8.4, ArH), 7.08 (2H, d, *J* = 8.4, ArH), 2.87 (3H, s, NCH_3), 2.81 (1H, dd, *J* = 15.3, 6.9, butyl H-C3), 2.75-2.67 (2H, m, butyl H_2-C1), 1.99-1.90 (1H, m, butyl H_a-C2), 1.90-1.80 (1H, m, butyl H_b-C2), 1.25 (3H, d, *J* = 6.9, butyl H_3-C4);

^{13}C NMR (125 MHz, $CDCl_3$) δ 145.7 (C_q), 131.5 (2 × C, ArC), 128.8 (2 × C, ArC), 119.7 (C_q), 64.0 (butyl C1), 53.1 (NCH_3), 36.7 (butyl C3), 36.3 (butyl C2), 22.3 (butyl C4);

IR ν_{max} (neat)/cm^{-1}: 2958, 2872, 2795, 1591, 1488, 1455, 1437, 1407;

HRMS (ESI): $C_{11}H_{16}^{35}Cl^{79}BrN$ [M+H$^+$]: calculated 276.1049, found 276.1045.

N-Chloro(methyl){3-[4-(tetramethyl-1,3,2-dioxaborolan-2-yl)phenyl]propyl}amine 1u

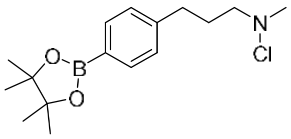

Following general procedure B, using methyl({3-[4-(tetramethyl-1,3,2-dioxaborolan-2-yl)phenyl]propyl})amine (250 mg, 0.91 mmol). Purification by flash chromatography on silica gel, eluting with 10% EtOAc in hexane afforded the *title compound* **1u** (232 mg, 0.75 mmol, 82%) as a clear yellow oil.

^{1}H NMR (400 MHz, $CDCl_3$) δ 7.74 (2H, d, *J* = 7.9, ArH), 7.21 (2H, d, *J* = 7.9, ArH), 2.92 (3H, s, NCH_3), 2.86 (2H, t, *J* = 7.4, propyl H_2-C1), 2.70 (2H, t, *J* = 7.4, propyl H_2-C3), 2.02-1.93 (2H, m, propyl H_2-C2), 1.34 (12H, s, $OC(CH_3)_2$);

^{13}C NMR (100 MHz, $CDCl_3$) δ 145.2 (C_q), 135.1 (2 × C, phenyl C3), 128.1 (2 × C, phenyl C-2), 83.8 (2 × C, O*C*$(CH_3)_2$), 65.3 (propyl C1), 53.2 (NCH_3), 33.1 (propyl C3), 29.7 (propyl C2), 25.0 (4 × C, OC(*C*$H_3)_2$), one ArC_q signal missing;

IR ν_{max} (neat) / cm^{-1}: 2972, 2877, 2802, 1611, 1559, 1520, 1460, 1439;

HRMS (ESI): $C_{16}H_{26}B^{35}ClNO$ [M+H$^+$]: calculated 310.1740, found 310.1739.

N-Chloro(methyl)[3-(2-phenylphenyl)propyl]amine 1v

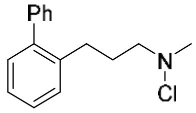

Following general procedure B, using *N*-methyl[3-(2-phenylphenyl)propyl]amine (250 mg, 1.11 mmol). Purification by flash chromatography on silica gel, eluting with 10% EtOAc in hexane afforded the *title compound* **1v** (215 mg, 0.83 mmol, 75%) as a pale yellow oil.

^{1}H NMR (400 MHz, $CDCl_3$) δ 7.37-7.09 (9H, m, ArH), 2.74 (3H, s, NCH_3), 2.69-2.64 (2H, m, propyl H_2-C1), 2.60-2.54 (2H, m, propyl H_2-C3), 1.75-1.65 (2H, m, propyl H_2-C2);

^{13}C NMR (100 MHz, $CDCl_3$) δ 142.1 (C_q), 141.9 (C_q), 139.3 (C_q), 130.3 (ArC), 129.5 (ArC), 129.3 (2 × C, ArC), 128.3 (2 × C, ArC), 127.6 (ArC), 127.0 (ArC), 126.0 (ArC), 65.7 (propyl C1), 53.0 (NCH_3), 30.3 (propyl C3), 29.8 (propyl C2);

IR ν_{max} (neat)/cm^{-1}: 3059, 3021, 2991, 2950, 2867, 2794, 1598, 1500;

HRMS (ESI): $C_{16}H_{19}^{35}ClN$ [M+H$^+$]: calculated 260.1201, found 260.1199.

N-Chloro-*N*-methyl-3-(*m*-tolyl)butan-1-amine 1w

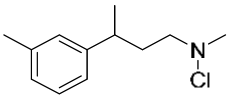

Following general procedure B, using *N*-methyl-3-(m-tolyl)butan-1-amine (250 mg, 1.41 mmol). Purification by flash chromatography on silica gel, eluting with 10% EtOAc in hexane afforded the *title compound* **1w** (249 mg, 1.18 mmol, 84%) as a colourless oil.

^{1}H NMR (500 MHz, $CDCl_3$) δ 7.24-7.15 (1H, m, ArH), 7.05-6.98 (3H, m, ArH), 2.89 (3H, s, NCH_3), 2.84-2.71 (3H, m, includes 2H, butyl H_2-C1; and 1H, m, butyl H-C3), 2.35 (3H, s, $ArCH_3$), 1.99-1.86 (2H, m, butyl H_2-C2), 1.28 (3H, d, *J* = 7.0, butyl H_3-C4);

^{13}C NMR (125 MHz, $CDCl_3$) δ 146.7 (C_q), 137.9 (C_q), 128.3 (ArC), 127.7 (ArC), 126.8 (ArC), 123.9 (ArC), 64.4 (butyl C1), 53.1 (NCH_3), 37.2 (butyl C3), 36.5 (butyl C2), 22.5 (butyl C4), 21.5 ($ArCH_3$);

IR ν_{max} (neat)/cm^{-1}: 3022, 2957, 2924, 2870, 1606, 1589, 1489, 1455;

HRMS (ESI): $C_{12}H_{19}^{35}ClN$ [M+H$^+$]: calculated 212.1201, found 212.1195.

N-Chloro[3-(3-chlorophenyl)propyl]methylamine 1x

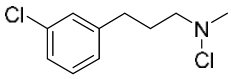

Following general procedure B, using [3-(3-chlorophenyl)propyl](methyl)amine (300 mg, 1.64 mmol). Purification by flash chromatography on silica gel, eluting with 10% EtOAc in hexane afforded the *title compound* **1x** (300 mg, 1.38 mmol, 85%) as a colourless oil.

^{1}H NMR (400 MHz, $CDCl_3$) δ 7.26-7.17 (3H, m, ArH), 7.10 (1H, d, *J* = 7.2, ArH), 2.96 (3H, s, NCH_3), 2.89 (2H, t, *J* = 6.8, propyl H_2-C1), 2.69 (2H, t, *J* = 7.7, propyl H_2-C3), 2.01-1.94 (2H, m, propyl H_2-C2);

^{13}C NMR (100 MHz, $CDCl_3$) δ 143.8 (C_q), 134.1 (C_q), 129.7 (ArC), 128.6 (ArC), 126.7 (ArC), 126.1 (ArC), 64.9 (propyl C1), 53.1 (NCH_3), 32.4 (propyl C3), 29.5 (propyl C2);

IR ν_{max} (neat) / cm^{-1}: 3061, 2992, 2950, 2919, 2867, 1597, 1572, 1475;

HRMS (ESI): $C_{10}H_{14}^{35}Cl_2N$ [M+H$^+$]: calculated 218.0498, found 218.0492.

N-Chloro-2-phenethylpyrrolidine 4a

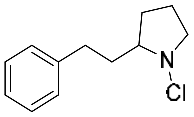

Following general procedure b, using 2-phenethylpyrrolidine (100 mg, 0.48 mmol). The reaction mixture was filtered through celite then the filtrate was collected then concentrated at room temperature *in vacuo* to afford the crude *N*-chloroamine **4a** as a colourless gum, which was used immediately without further purification.

N-Chloro-2-phenethylpiperidine 4b

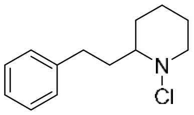

Following general procedure b, using 2-phenethylpiperidine (150 mg, 0.67 mmol). The reaction mixture was filtered through celite then the filtrate was collected then concentrated at room temperature *in vacuo* to afford the crude *N*-chloroamine **4b** as a colourless gum, which was used immediately without further purification.

(1*R**,2*S**)-2-Benzyl-*N*-chloro-*N*-methylcyclohexan-1-amine 6

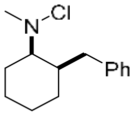

To a stirred solution of 2-benzylcyclohexan-1-one (2.80 g, 14.9 mmol, 1.0 eq.) in MeOH (30 mL) at rt was added NH_2Me (12 mL of an 8.0 M solution in EtOH, 10 eq.) and $Ti(O^iPr)_4$ (9.04 mL, 29.8 mmol, 2.0 eq.). The reaction mixture was stirred for 16 h at rt, then it was cooled to 0 °C and $NaBH_4$ (845 mg, 22.4 mmol, 1.5 eq.) was added portionwise then the reaction mixture was warmed to rt and stirred for 1 h. The reaction mixture was concentrated *in vacuo* then the crude gum was taken up in EtOAc (50 mL) and a 2 M aqueous NH_4OH (40 mL) and Na_2SO_4 were added. The resultant slurry was filtered through a pad of Celite, the Celite washed with EtOAc (400 mL) and the filtrate collected and concentrated *in vacuo* to afford the crude amine as an inseparable mixture of diastereoisomers (confirmation by LC-MS analysis).

Following general procedure B, using the crude amine mixture (350 mg, 1.72 mmol). Purification by flash chromatography on silica gel, eluting with a gradient of 20-40% DCM in hexane afforded the *title compound* **6** (280 mg, 1.18 mmol, 69%) as a yellow oil.

^{1}H NMR (500 MHz, $CDCl_3$) δ 7.28-7.13 (5H, m, ArH), 3.05 (3H, s, NCH_3), 2.97-2.88 (1H, m, benzyl H_a-C1), 2.69 (1H, dt, J = 11.6, 3.3, cyclohexyl H-C1), 2.55-2.45 (2H, m, includes 1H, m, benzyl H_b-C1; and 1H, m, cyclohexyl H-C2), 2.04-1.94 (1H, m, cyclohexyl H_a-C6), 1.91-1.82

(1H, m, cyclohexyl H_a-C5), 1.60 (1H, d, *J* = 14.0, cyclohexyl H_a-C3), 1.57-1.45 (2H, m, cyclohexyl H_2-C4), 1.45-1.36 (1H, m, cyclohexyl H_b-C6), 1.35-1.24 (1H, m, cyclohexyl H_b-C5), 1.23-1.16 (1H, m, cyclohexyl H_b-C3);

^{13}C NMR (75 MHz, $CDCl_3$) δ 141.6 (C_q), 128.9 (2 × C, ArC), 127.9 (2 × C, ArC), 125.3 (ArC), 73.8 (cyclohexyl C1), 49.7 (NCH_3), 39.0 (cyclohexyl C2), 29.7 ($PhCH_2$), 26.4 (cyclohexyl C3), 25.9 (cyclohexyl C5), 25.2 (cyclohexyl C6), 19.0 (cyclohexyl C4);

IR ν_{max} (neat)/cm^{-1}: 3025, 2928, 2855, 1601, 1494, 1448, 1367, 1338;

HRMS (ESI): $C_{14}H_{21}{}^{35}ClN$ [M+H$^+$]: calculated 238.1363, found 238.1357.

(1*R**,3*S**)-*N*-Chloro-*N*-methyl-3-phenylcyclohexan-1-amine 8

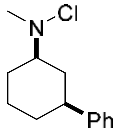

To a stirred solution of ketone (1.00 g, 5.74 mmol, 1.0 eq.) in MeOH (11.5 mL) at RT was added NH_2Me (7.0 mL of an 8.0 M solution in EtOH, 10 eq.) and Ti(O^{i}Pr)$_4$ (3.41 mL, 11.5 mmol, 2.0 eq.). The reaction mixture was stirred for 16 h at RT, then it was cooled to 0 °C and $NaBH_4$ (326 mg, 8.61 mmol, 1.5 eq.) was added portionwise then the reaction mixture was warmed to RT and stirred for 1 h. The reaction mixture was concentrated *in vacuo* then the crude gum was taken up in EtOAc (25 mL) and a 2 M aqueous NH_4OH (20 mL) and Na_2SO_4 were added. The resultant slurry was filtered through a pad of Celite, the Celite washed with EtOAc (250 mL) and the filtrate collected and concentrated *in vacuo* to afford the crude amine as an inseparable mixture of diastereoisomers (confirmation by LC-MS analysis).

Following general procedure B, using the crude amine mixture (1.00 g, 5.28 mmol). Purification by flash chromatography on silica gel, eluting with a gradient of 20-40% DCM in hexane afforded the *title compound* **8** (817 mg, 3.65 mmol, 69%) as a yellow oil.

^{1}H NMR (500 MHz, $CDCl_3$) δ 7.34-7.17 (5H, m, ArH), 2.95 (3H, s, NCH_3), 2.84 (1H, tt, *J* = 11.0, 3.5, cyclohexyl H-C1), 2.59 (1H, tt, *J* = 12.1, 3.3, cyclohexyl H-C3), 2.27-2.22 (1H, m, cyclohexyl H_a-C2), 2.17-2.10 (1H, m, cyclohexyl H_a-C6), 1.99 (1H, ddd, *J* = 9.1, 6.5, 3.1, cyclohexyl H_a-C5), 1.87 (1H, dd, *J* = 9.1, 3.9, cyclohexyl H_a-C4), 1.59-1.33 (4H, m, includes 1H, m, cyclohexyl H_b-C2; and1H, m, cyclohexyl H_b-C4; and 1H, m, cyclohexyl H_b-C5; and1H, m, cyclohexyl H_b-C6);

^{13}C NMR (125 MHz, $CDCl_3$) δ 146.5 (C_q), 128.6 (2 × C, ArC), 127.0 (2 × C, ArC), 126.4 (ArC), 70.2 (cyclohexyl C1), 48.6 (NCH_3), 43.5 (cyclohexyl C3), 37.0 (cyclohexyl C2), 33.7 (cyclohexyl C4), 29.3 (cyclohexyl C6), 25.3 (cyclohexyl C5);

IR ν_{max} (neat)/cm^{-1}: 3027, 2931, 2857, 1668, 1602, 1494, 1449, 1408;

HRMS (ESI): $C_{13}H_{19}{}^{35}ClN$ [M+H$^+$]: calculated 224.1201, found 224.1192.

N-Chloro-*N*-methyl-3-phenyl-3-(*p*-tolyl)propan-1-amine 10

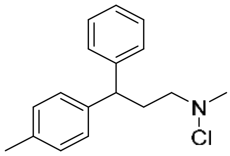

Following general procedure B, using *N*-methyl-3-phenyl-3-(p-tolyl)propan-1-amine (500 mg, 2.09 mmol). Purification by flash chromatography on silica gel, eluting with 10% EtOAc in hexane afforded the *title compound* **10** (465 mg, 1.70 mmol, 81%) as a colourless oil.

^{1}H NMR (500 MHz, $CDCl_3$) δ 7.32-7.22 (4H, m, ArH), 7.20-7.08 (5H, m, ArH), 4.04 (1H, t, *J* = 7.9, propyl H-C3), 2.89 (3H, s, NCH_3), 2.85-2.78 (2H, m, propyl H_2-C1), 2.42-2.36 (2H, m, propyl H_2-C2), 2.31 (3H, s, $ArCH_3$);

^{13}C NMR (125 MHz, $CDCl_3$) δ 144.7 (C_q), 141.4 (C_q), 135.8 (C_q), 129.2 (2 × C, ArC), 128.5 (2 × C, ArC), 127.8 (2 × C, ArC), 127.7 (2 × C, ArC), 126.2 (ArC), 64.3 (propyl C1), 53.2 (NCH_3), 47.7 (propyl C3), 34.0 (propyl C2), 21.0 ($ArCH_3$);

IR ν_{max} (neat)/cm^{-1}: 3023, 2981, 2938, 2920, 2890, 2852, 1597, 1581;

HRMS (ESI): $C_{17}H_{21}{}^{35}ClN$ [M+H$^+$]: calculated 274.1357, found 274.1354.

N-Chloro-*N*-methyl-3-phenyl-3-(3-(trifluoromethyl)phenyl)propan-1-amine 11

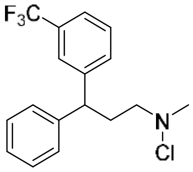

Following general procedure B, using *N*-methyl-3-phenyl-3-(3-(trifluoromethyl)phenyl)propan-1-amine (150 mg, 0.51 mmol). Purification by flash chromatography on silica gel, eluting with 10% EtOAc in hexane afforded the *title compound* **11** (151 mg, 0.46 mmol, 90%) as a colourless oil.

^{1}H NMR (500 MHz, $CDCl_3$) δ 7.52 (1H, s, ArH), 7.48-7.37 (3H, m, ArH), 7.34-7.29 (2H, m, ArH), 7.27-7.19 (3H, m, ArH), 4.18 (1H, t, *J* = 7.9, propyl H-C3), 2.89 (3H, s, NCH_3), 2.79 (2H, t, *J* = 6.8, propyl H_2-C1), 2.47-2.35 (2H, m, propyl H_2-C2);

^{13}C NMR (125 MHz, $CDCl_3$) δ 145.5 (C_q), 143.3 (C_q), 131.3 (ArC), 130.8 (q, *J* = 31.9, C_q), 129.0 (ArC), 128.7 (2 × C, ArC), 127.9 (2 × C, ArC), 126.7 (ArC), 124.6 (q, *J* = 3.8, ArC), 124.2 (q, *J* = 272.4, CF_3), 123.2 (q, *J* = 3.8, ArC), 63.7 (propyl C1), 53.2 (NCH_3), 47.7 (propyl C3), 33.8 (propyl C2);

IR ν_{max} (neat)/cm^{-1}: 3062, 3028, 2952, 2881, 1599, 1494, 1445, 1326;

HRMS (ESI): $C_{17}H_{18}{}^{35}ClF_3N$ [M+H$^+$]: calculated 328.1074, found 328.1069.

N-Chloro[3-(2,6-dichlorophenyl)propyl]methylamine 14

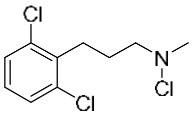

Following general procedure B, using [3-(2,6-dichlorophenyl)propyl](methyl)amine (250 mg, 1.15 mmol). Purification by flash chromatography on silica gel, eluting with 10% EtOAc in hexane afforded the *title compound* **15** (268 mg, 1.06 mmol, 92%) as a pale yellow oil.

^{1}H NMR (400 MHz, $CDCl_3$) δ 7.30-7.23 (2H, m, ArH), 7.10-7.04 (1H, m, ArH), 3.03-2.93 (7H, m, includes 3H, s, NCH_3; 2H, m, propyl H_2-C1; and 2H, m, propyl H_2-C3), 1.98-1.87 (2H, m, propyl H_2-C2);

^{13}C NMR (100 MHz, $CDCl_3$) δ 137.9 (C_q), 135.5 (2 × C, C_q), 128.3 (2 × C, ArC), 127.8 (ArC), 65.8 (propyl C1), 53.1 (NCH_3), 28.7 (propyl C3), 26.9 (propyl C2);

IR ν_{max} (neat)/cm^{-1}: 2950, 2873, 2845, 2794, 1582, 1561, 1455, 1434;

HRMS (ESI): $C_{10}H_{13}{}^{35}Cl_3N$ [M+H$^+$]: calculated 252.0108, found 252.0104.

N-Chloro[3-(2,6-dimethylphenyl)butyl]methylamine 16

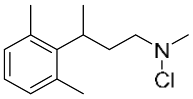

Following general procedure B, using [3-(2,6-dimethylphenyl)butyl](methyl)amine (250 mg, 1.31 mmol). Purification by flash chromatography on silica gel, eluting with 10% EtOAc in hexane afforded the *title compound* **17** (256 mg, 1.13 mmol, 86%) as a colourless oil.

^{1}H NMR (500 MHz, $CDCl_3$) δ 7.00 (3H, s, ArH), 3.44-3.34 (1H, m, butyl H-C3), 2.89 (3H, s, NCH_3), 2.83-2.71 (2H, m, butyl H_2-C1), 2.49-2.30 (6H, br. m, 2 × $ArCH_3$), 2.21-2.12 (1H, m, butyl H_a-C2), 2.08-2.00 (1H, m, butyl H_b-C2), 1.34 (3H, d, J = 7.3, butyl H_3-C4);

^{13}C NMR (125 MHz, $CDCl_3$) δ 142.0 (ArC), 136.4 (C_q), 130.4 (C_q), 128.3 (C_q), 125.7 (2 × C, ArC), 64.8 (butyl C1), 53.1 (NCH_3), 33.6 (butyl C2), 32.1 (butyl C3), 21.5 (2 × C, $ArCH_3$), 17.0 (butyl C4);

IR ν_{max} (neat)/cm^{-1}: 2956, 2873, 2794, 1580, 1462, 1438, 1262, 1176;

HRMS (ESI): $C_{13}H_{21}{}^{35}ClN$ [M+H$^+$]: calculated 226.1357, found 226.1353.

1.4 Tetrahydroquinoline data

Reaction optimisation: equivalents of methanesulfonic acid

To a solution of *N*-chloroamine **1a** (0.25 M in DCM) in a PYREX glass tube was added portionwise the requisite amount of methanesulfonic acid. The reactor was then irradiated with UV light according to General Procedure O. The reaction mixtures were analysed by ^{1}H NMR for the ratio of **2a** to the dechlorinated amine **3a** and, where appropriate, the product **2a** was isolated.

Entry	Conditions	Ratio (**2a**:**3a**)	Isolated Yield **2a**
1	$MeSO_3H$:DCM (1:1 vol)	100:0	80%
2	$MeSO_3H$ (10 equiv.)	100:0	91%
3	$MeSO_3H$ (5 equiv.)	75:25	61%
4	$MeSO_3H$ (2.5 equiv.)	0:100	n/a

General Procedure O: *N*-arylation reaction

A PYREX glass test tube (total vol. = 7 mL) was placed in a carousel holder that was placed in a water bath with the water at 18 °C, all above a stirrer hotplate. A solution of the *N*-chloroamine (1.0 eq.) in DCM (0.25 M) was added to the vial and stirred with a magnetic stirrer bar, then methanesulfonic acid (10 eq.) was added portionwise. The reactor was covered in aluminium foil and a red Perspex box was placed around it, then the reaction mixture was irradiated under UV light with a 125 W medium pressure mercury lamp at rt for 5 h. The reaction was either worked up by SCX cartridge (workup A) or by basic aqueous work up (workup B). Purification afforded the desired product.

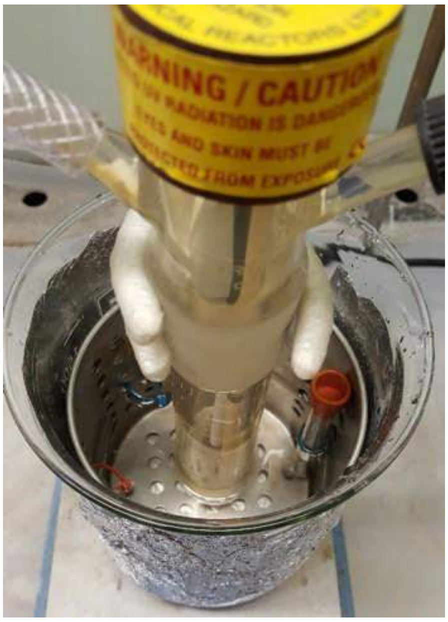

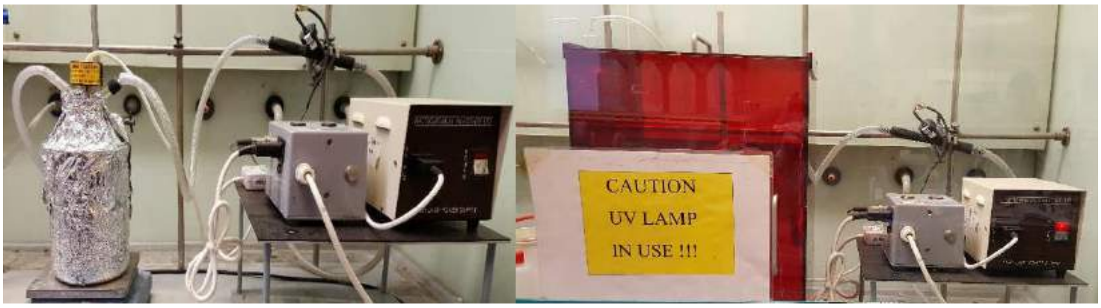

Workup A: SCX cartridge

TfOH (0.5 M in MeOH/5 g SPE-SCX) was washed through the SPE-SCX cartridge prior to use. The crude residue was loaded (3.5 mmol/5 g SPE-SCX silica) in the minimum amount of MeOH. The cartridge was washed with MeOH and the filtrate was collected. The cartridge was then washed with sat. methanolic NH_3 and the filtrate was collected and concentrated *in vacuo*.

Workup B: Basic aqueous workup

The crude reaction mixture was taken up in H_2O and washed with EtOAc. The aqueous phase was then basified with 2 M aqueous NaOH and extracted with EtOAc (× 3). The combined organic extracts were washed with brine, dried over Na_2SO_4 and concentrated *in vacuo*.

1-Methyl-1,2,3,4-tetrahydroquinoline 2a

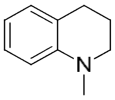

Following general procedure O, using chloroamine **1a** (100 mg, 0.54 mmol). Workup A afforded the title compound **2a** (72 mg, 0.49 mmol, 91%) as a yellow oil. The data is in accordance with the literature.[20]

^{1}H NMR (300 MHz, $CDCl_3$) δ 7.11-7.04 (1H, m, ArH-C7), 6.98-6.92 (1H, m, ArH-C5), 6.65-6.56 (2H, m, includes 1H, m, ArH-C6; and 1H, m, ArH-C8), 3.25-3.18 (2H, m, H_2-C2), 2.89 (3H, s, NCH_3), 2.81-2.73 (2H, m, H_2-C4), 2.03-1.92 (2H, m, H_2-C3);

^{13}C NMR (75 MHz, $CDCl_3$) δ 146.8 (C_q), 128.8 (ArC), 127.0 (ArC), 122.9 (C_q), 116.2 (ArC), 110.9 (ArC), 51.3 (C2), 39.1 (NCH_3), 27.8 (C4), 22.5 (C3);

IR ν_{max} (neat)/cm^{-1}: 2929, 2838, 1602, 1505, 1464, 1321, 1305, 1189;

HRMS (ESI): $C_{10}H_{14}N$ [M+H$^+$]: calculated 148.1121, found 148.1118.

1-Benzyl-1,2,3,4-tetrahydroquinoline 2b

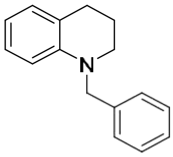

Following general procedure O, using chloroamine **1b** (100 mg, 0.38 mmol). Workup A afforded the title compound **2b** (69 mg, 0.31 mmol, 82%) as a yellow oil. The data is in accordance with the literature.[21]

^{1}H NMR (300 MHz, $CDCl_3$) δ 7.38-7.17 (5H, m, ArH), 7.05-6.89 (2H, m, includes 1H, m, ArH-C7; and 1H, m, ArH-C5), 6.61-6.48 (2H, m, includes 1H, m, ArH-C6; and 1H, m, ArH-C8), 4.48 (2H, s, NCH_2Ph), 3.37 (2H, t, J = 5.6, H_2-C2), 2.82 (2H, t, J = 6.3, H_2-C4), 2.10-1.92 (2H, m, H_2-C3);

^{13}C NMR (125 MHz, $CDCl_3$) δ 145.6 (C_q), 138.9 (C_q), 129.0 (ArC), 128.6 (2 × C, ArC), 127.1 (ArC), 126.7 (ArC), 126.6 (2 × C, ArC), 122.2 (C_q), 115.8 (ArC), 110.9 (ArC), 55.2 (NCH_2Ph), 49.9 (C2), 28.2 (C4), 22.4 (C3);

IR ν_{max} (neat)/cm^{-1}: 3061, 3024, 2924, 2839, 1600, 1494, 1449, 1343;

HRMS (ESI): $C_{16}H_{18}N$ [M+H$^+$]: calculated 224.1434, found 224.1435.

1-Butyl-1,2,3,4-tetrahydroquinoline 2c

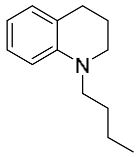

Following general procedure O, using chloroamine **1c** (100 mg, 0.44 mmol). Workup A afforded the *title compound* **2c** (49 mg, 0.26 mmol, 59%) as a brown oil.

^{1}H NMR (300 MHz, $CDCl_3$) δ 7.09-6.99 (1H, m, ArH-C7), 6.93 (1H, d, J = 7.2, ArH-C5), 6.60-6.49 (2H, m, includes 1H, m, ArH-C6; and 1H, m, ArH-C8), 3.32-3.18 (4H, m, includes 2H, m, H_2-C4; and 2H, m, butyl C1), 2.75 (2H, t, J = 6.4, H_2-C2), 2.00-1.89 (2H, m, H_2-C3), 1.64-1.51 (2H, m, butyl H_2-C2), 1.44-1.29 (2H, m, butyl H_2-C3), 0.96 (3H, t, J = 7.3, butyl H_3-C4);

^{13}C NMR (75 MHz, $CDCl_3$) δ 145.4 (C_q), 129.1 (ArC), 127.0 (ArC), 122.1 (C_q), 115.1 (ArC), 110.4 (ArC), 51.2 (butyl C1), 49.4 (C2), 28.4 (butyl C2), 28.2 (C4), 22.2 (C2), 20.5 (butyl C3), 14.0 (butyl C4);

IR ν_{max} (neat)/cm^{-1}: 3019, 2953, 2929, 2859, 1601, 1503, 1456, 1367;

HRMS (ESI): $C_{13}H_{20}N$ [M+H$^+$]: calculated 190.1590, found 190.1591.

1-Hexyl-1,2,3,4-tetrahydroquinoline 2d

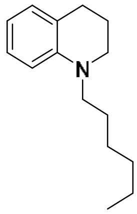

Following general procedure O, using chloroamine **1d** (100 mg, 0.39 mmol). Workup B followed by purification by flash chromatography on silica gel, eluting with 10% EtOAc in hexane afforded the *title compound* **2d** (23 mg, 0.11 mmol, 28%) as a colourless oil.

^{1}H NMR (300 MHz, $CDCl_3$) δ 7.09-6.99 (1H, m, ArH-C7), 6.97-6.89 (1H, m, ArH-C5), 6.63-6.47 (2H, m, includes 1H, m, ArH-C6; and 1H, m, ArH-C8), 3.34-3.15 (4H, m, includes: 2H, m, H_2-C2; and 2H, m, hexyl H_2-C1), 2.75 (2H, t, *J* = 6.4, H_2-C4), 2.02-1.88 (2H, m, H_2-C3), 1.66-1.51 (2H, m, hexyl H_2-C2), 1.40-1.24 (6H, m, hexyl H_2-C3-5), 0.98-0.81 (3H, m, hexyl H_3-C6);

^{13}C NMR (125 MHz, $CDCl_3$) δ 145.5 (C_q), 129.3 (ArC), 127.2 (ArC), 122.3 (C_q), 115.3 (ArC), 110.6 (ArC), 51.7 (CH_2), 49.6 (CH_2), 31.9 (CH_2), 28.4 (CH_2), 27.1 (CH_2), 26.3 (CH_2), 22.8 (CH_2), 22.4 (CH_2), 14.2 (*C*H_3);

IR ν_{max} (neat)/cm^{-1}: 3066, 2925, 2855, 1601, 1574, 1504, 1456, 1369;

HRMS (ESI): $C_{15}H_{24}N$ [M+H$^+$]: calculated 218.1903, found 218.1902.

A crude amine-containing fraction was also isolated (24 mg), which was suspected to be derived from Hofmann-Loeffler-Freytag reaction at the hexyl side-chain. This crude material was converted to its *N*-nosyl derivative by treatment with *p*-NsCl/Et_3N and then isolated by column chromatography. Overall, 12 mg of *N*-(4-chlorohexyl)-4-nitro-*N*-(3-phenylpropyl)benzene-1-sulfonamide was isolated (10% from **1d**), confirming H-L-F functionalisation of the side-chain.

1-Allyl-1,2,3,4-tetrahydroquinoline 2e

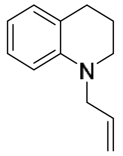

Following general procedure O, using chloroamine **1e** (100 mg, 0.48 mmol). Workup A followed by purification by flash chromatography on silica gel, eluting with 10% EtOAc in hexane afforded the *title compound* **2e** (33 mg, 0.19 mmol, 40%) as a colourless oil.

^{1}H NMR (500 MHz, $CDCl_3$) δ 7.06-7.00 (1H, m, ArH), 6.97-6.93 (1H, m, ArH), 6.60-6.53 (2H, m, includes 1H, m, ArH-C6; and 1H, m, ArH-C8), 5.86 (1H, ddt, *J* = 17.1, 10.1, 5.0, propenyl H-C2), 5.24 (1H, dd, *J* = 17.1, 1.7, propenyl H_A-C3), 5.18 (1H, dd, *J* = 10.1, 1.7, propenyl H_B-C3), 3.87 (2H, dt, *J* = 4.9, 1.6, propenyl H_2-C1), 3.31-3.24 (2H, m, H_2-C2), 2.77 (2H, t, *J* = 6.3, H_2-C4), 2.01-1.93 (2H, m, H_2-C3);

^{13}C NMR (125 MHz, $CDCl_3$) δ 145.3 (C_q), 133.6 (propenyl C2), 129.0 (ArC), 127.0 (ArC), 122.4 (C_q), 115.9 (ArC), 115.7 (propenyl C3), 111.0 (ArC), 53.8 (propenyl C1), 49.1 (C2), 28.1 (C4), 22.3 (C3);

IR ν_{max} (neat)/cm^{-1}: 3036, 3018, 2927, 2840, 1641, 1601, 1574, 1502;

HRMS (ESI): $C_{12}H_{16}N$ [M+H$^+$]: calculated 174.1277, found 174.1272.

1,2-Dimethyl-1,2,3,4-tetrahydroquinoline 2f

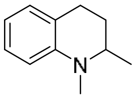

Following general procedure O, using chloroamine **1f** (300 mg, 1.52 mmol). Workup B followed by purification by flash chromatography on silica gel, eluting with 10% EtOAc in hexane afforded the *title compound* **2f** (194 mg, 1.20 mmol, 79%) as a yellow oil.

^{1}H NMR (500 MHz, $CDCl_3$) δ 7.13 (1H, t, *J* = 7.7, ArH-C7), 7.02 (1H, d, *J* = 7.3, ArH-C5), 6.64 (1H, t, *J* = 7.3, ArH-C6), 6.60 (1H, d, *J* = 8.2, ArH-C8), 3.52-3.44 (1H, m, H-C2), 2.94 (3H, s, NCH_3), 2.93-2.84 (1H, m, H_a-C4), 2.73 (1H, dt, *J* = 16.1, 4.6, H_b-C4), 2.07-1.99 (1H, m, H_a-C3), 1.84-1.76 (1H, m, H_b-C3), 1.18 (3H, d, *J* = 6.5, CH_3);

^{13}C NMR (125 MHz, $CDCl_3$) δ 145.4 (C_q), 128.5 (ArC), 127.1 (ArC), 122.1 (C_q), 115.4 (ArC), 110.6 (ArC), 53.8 (C2), 37.0 (NCH_3), 28.1 (C3), 23.8 (C4), 17.6 (CH_3);

IR ν_{max} (neat)/cm^{-1}: 3066, 3019, 2964, 2928, 2846, 2792, 1601, 1575;

HRMS (ESI): $C_{11}H_{16}N$ [M+H^+]: calculated 162.1277, found 162.1270.

1-Benzyl-2-methyl-1,2,3,4-tetrahydroquinoline 2g

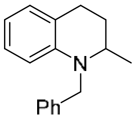

Following general procedure B, using chloroamine **1g** (100 mg, 0.37 mmol). Workup B, followed by purification by flash chromatography on silica gel, eluting with 10% EtOAc in hexane afforded the *title compound* **2g** (47 mg, 0.20 mmol, 54%) as a clear yellow oil.

^{1}H NMR (400 MHz, $CDCl_3$) δ 7.39-7.23 (5H, m, ArH), 7.05 (1H, d, *J* = 7.3, ArH-C5), 6.98 (1H, app. t, *J* = 7.7, ArH-C7), 6.61 (1H, app. t, *J* = 7.3, ArH-C6), 6.44 (1H, d, *J* = 8.2, ArH-C8), 4.60 (1H, d, *J* = 17.3, NCH_aH_b), 4.50 (1H, d, *J* = 17.3, NCH_aH_b), 3.67-3.57 (1H, m, H-C2), 3.02-2.91 (1H, m, H_a-C4), 2.80 (1H, dt, *J* = 16.0, 4.5, H_b-C4), 2.14-2.03 (1H, m, H_a-C3), 1.88 (1H, ddd, *J* = 13.0, 8.6, 4.3, H_b-C3), 1.23 (3H, d, *J* = 6.4, CH_3);

^{13}C NMR (100 MHz, $CDCl_3$) δ 144.9 (C_q), 139.6 (C_q), 128.9 (ArH), 128.7 (2 × C, ArC), 127.2 (C5), 126.8 (C7), 126.5 (2 × C, ArC), 121.9 (C_q), 115.6 (C6), 111.6 (C8), 53.5 (NCH_2), 53.2 (C2), 28.3 (C3), 24.2 (C4), 19.1 (methyl C1);

IR ν_{max} (neat) / cm^{-1}: 3062, 3024, 2964, 2926, 2848, 1600, 1574, 1494;

HRMS (ESI): $C_{17}H_{20}N$ [M+H^+]: calculated 238.1590, found 238.1586.

1-Methyl-2-phenyl-1,2,3,4-tetrahydroquinoline 2h

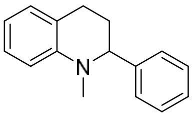

Following general procedure O, using chloroamine **1h** (250 mg, 0.96 mmol). Workup B followed by purification by flash chromatography on silica gel, eluting with 10% EtOAc in hexane afforded the *title compound* **2h** (96 mg, 0.43 mmol, 50%) as a yellow oil.

^{1}H NMR (500 MHz, $CDCl_3$) δ 7.34-7.28 (2H, m, ArH), 7.27-7.21 (1H, m, ArH), 7.21-7.14 (3H, m, ArH), 6.99 (1H, d, *J* = 7.2, ArH), 6.68 (1H, d, *J* = 8.2, ArH), 6.67-6.62 (1H, m, ArH), 4.49 (1H, t, *J* = 4.8, H-C2), 2.88 (3H, s, NCH_3), 2.66-2.54 (2H, m, H_2-C4), 2.25-2.16 (1H, m, H_a-C3), 2.06-1.99 (1H, m, H_b-C3);

^{13}C NMR (125 MHz, $CDCl_3$) δ 146.1 (C_q), 144.3 (C_q), 128.4 (2 × C, ArC), 127.3 (ArC), 126.8 (ArC), 126.5 (2 × C, ArC), 122.6 (C_q), 115.6 (ArC), 109.9 (ArC), 63.3 (C2), 37.7 (NCH_3), 30.2 (C3), 24.2 (C4);

IR ν_{max} (neat)/cm^{-1}: 3024, 2930, 2894, 2838, 1600, 1502, 1448, 1379;

HRMS (ESI): $C_{16}H_{18}N$ [M+H$^+$]: calculated 224.1434, found 224.1429.

1-Methyl-2-vinyl-1,2,3,4-tetrahydroquinoline 2i

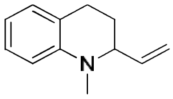

Following general procedure O, using chloroamine **1i** (500 mg, 2.38 mmol). Workup B followed by purification by flash chromatography on silica gel, eluting with 10% EtOAc in hexane afforded the *title compound* **2i** (209 mg, 1.21 mmol, 51%) as a colourless oil.

^{1}H NMR (500 MHz, $CDCl_3$) δ 7.12 (1H, t, *J* = 7.8, ArH-C7), 6.98 (1H, d, *J* = 7.1, ArH-C5), 6.65-6.59 (2H, m, ArH), 5.80 (1H, ddd, *J* = 16.9, 10.2, 6.5, vinyl H-C1), 5.13 (2H, ddt, *J* = 29.4, 17.1, 1.4, vinyl H_2-C2), 3.82 (1H, ddd, *J* = 10.4, 5.2, 0.9, H-C2), 2.91 (3H, s, NCH_3), 2.83-2.74 (1H, m, H_a-C4), 2.68 (1H, dt, *J* = 15.8, 4.7, H_b-C4), 2.02 (1H, ddt, *J* = 12.9, 11.4, 4.9, H_a-C3), 1.95-1.86 (1H, m, H_b-C3);

^{13}C NMR (125 MHz, $CDCl_3$) δ 145.6 (C_q), 138.9 (vinyl C1), 128.4 (ArC), 127.2 (ArC), 122.4 (C_q), 115.6 (ArC), 115.5 (vinyl C2), 110.2 (ArC), 61.7 (C2), 37.3 (NCH_3), 27.1 (C3), 24.3 (C4);

IR ν_{max} (neat)/cm^{-1}: 3019, 2976, 2929, 2894, 1639, 1601, 1575, 1498;

HRMS data could not be obtained.

Synthesis of 1-methyl-2-pentyl-1,2,3,4-tetrahydroquinoline 2j

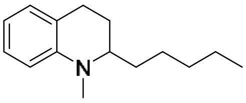

Following general procedure O, using chloroamine **1j** (100 mg, 0.39 mmol). Work up B then purification by flash chromatography on silica gel, eluting with 10% EtOAc in hexane afforded the title compound **2j** (59 mg, 0.27 mmol, 69%) as a clear yellow oil. The data is in accordance with the literature.[22]

^{1}H NMR (400 MHz, $CDCl_3$) δ 7.09 (1H, app. t, *J* = 7.7, ArH-C7), 6.98 (1H, d, *J* = 7.2, ArH-C5), 6.59 (1H, app. t, *J* = 7.3, ArH-C6), 6.54 (1H, d, *J* = 8.2, ArH-C8), 3.24 (1H, td, *J* = 8.4, 4.1, H_1-C2), 2.94 (3H, s, NCH_3), 2.87-2.76 (1H, m, H_a-C4), 2.67 (1H, dt, *J* = 16.2, 4.1, H_b-C4), 1.95-1.86 (2H, m, H_2-C3), 1.67-1.52 (1H, m, pentyl H_a-C1), 1.48-1.20 (7H, m, includes 1H, m, pentyl H_b-C1; and 6H, m, H_2-C5-7), 0.91 (3H, t, *J* = 6.8, pentyl H_3-C5);

^{13}C NMR (100 MHz, $CDCl_3$) δ 145.5 (C_q), 128.8 (C5), 127.2 (C7), 122.0 (C_q), 115.3 (C6), 110.5 (C8), 59.1 (C2), 38.1 (NCH_3), 32.2 (CH_2), 31.3 (pentyl C1), 25.9 (CH_2), 24.6 (C3), 23.7 (C4), 22.8 (CH_2), 14.2 (pentyl C5);

IR ν_{max} (neat)/cm^{-1}: 3020, 2926, 2856, 1602, 1575, 1498, 1479, 1455;

HRMS (ESI): $C_{15}H_{24}N$ [M+H$^+$]: calculated 218.1903, found 218.1903.

3-Methoxy-1-methyl-1,2,3,4-tetrahydroquinoline 2k

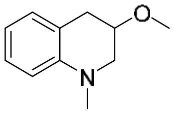

Following general procedure O, using chloroamine **1k** (100 mg, 0.47 mmol). Workup B, followed by purification by flash chromatography on silica gel, eluting with 10% EtOAc in hexane afforded the *title compound* **2k** (34 mg, 0.19 mmol, 40%) as a colourless oil.

^{1}H NMR (400 MHz, $CDCl_3$) δ 7.10 (1H, app. t, *J* = 7.5, ArH-C7), 7.00 (1H, d, *J* = 7.3, ArH-C5), 6.69-6.60 (2H, m. ArH), 3.84-3.75 (1H, m, H-C3), 3.46 (3H, s, OCH_3), 3.37 (1H, app. ddd, *J* = 11.1, 3.8, 1.8, H_a-C2), 3.15 (1H, app. ddd, *J* = 11.1, 7.1, 0.8, H_b-C2), 3.06 (1H, dd, *J* = 15.5, 4.3, H_a-C4), 2.92 (3H, s, NCH_3), 2.82 (1H, dd, *J* = 7.6, 5.7, H_b-C4);

^{13}C NMR (100 MHz, $CDCl_3$) δ 146.2 (C_q), 129.6 (ArC), 127.5 (ArC), 120.5 (C_q), 117.1 (ArC), 111.1 (ArC), 73.3 (C3), 56.4 (OCH_3), 54.8 (C4), 39.2 (NCH_3), 33.5 (C2);

IR ν_{max} (neat)/cm^{-1}: 2929, 2894, 2823, 1675, 1628, 1602, 1579, 1499;

HRMS (ESI): $C_{11}H_{16}NO$ [M+H$^+$]: calculated 178.1226, found 178.1233.

1,3-Dimethyl-1,2,3,4-tetrahydroquinoline 2l

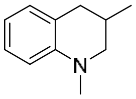

Following general procedure O, using chloroamine **1l** (100 mg, 0.51 mmol). Workup B followed by purification by flash chromatography on silica gel, eluting with 10% EtOAc in hexane

afforded the title compound **2l** (62 mg, 0.39 mmol, 77%) as a clear yellow oil. The ^{1}H NMR data was in accordance with the literature.[23]

^{1}H NMR (500 MHz, $CDCl_3$) δ 7.11-7.05 (1H, m, ArH), 6.98-6.93 (1H, m, ArH), 6.65-6.58 (2H, m, ArH), 3.17 (1H, app. ddd, *J* = 11.0, 4.0, 2.1, H_a-C2), 2.92-2.85 (4H, m, includes 3H, s, NCH_3; and 1H, m, H_b-C2), 2.79 (1H, app. ddd, *J* = 15.7, 4.8, 1.7, H_a-C4), 2.45 (1H, dd, *J* = 15.7, 10.6, H_b-C4), 2.18-2.08 (1H, m, H-C3), 1.05 (3H, d, *J* = 6.6, CH_3);

^{13}C NMR (125 MHz, $CDCl_3$) δ 146.4 (C_q), 129.0 (ArC), 127.2 (ArC), 122.6 (C_q), 116.3 (ArC), 110.9 (ArC), 58.5 (C2), 39.2 (NCH_3), 36.4 (C4), 27.6 (C3), 19.3 (CH_3);

IR ν_{max} (neat)/cm^{-1}: 3021, 2952, 2905, 2870, 2830, 1603, 1500, 1432;

HRMS (ESI): $C_{11}H_{16}N$ [M+H$^+$]: calculated 162.1277, found 162.1273.

1,4-Dimethyl-1,2,3,4-tetrahydroquinoline 2m

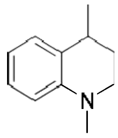

Following general procedure O, using chloroamine **1m** (100 mg, 0.51 mmol). Workup A afforded the *title compound* **2m** (59 mg, 0.37 mmol, 73%) as a yellow oil.

^{1}H NMR (300 MHz, $CDCl_3$) δ 7.13-7.02 (2H, m, ArH), 6.69-6.58 (2H, m, ArH), 3.31-3.14 (2H, m, H_2-C2), 2.95-2.84 (4H, m, includes 3H, s, NCH_3; and 1H, m, butyl H-C4), 2.10-1.97 (1H, m, H_a-C3), 1.75-1.64 (1H, m, H_b-C3), 1.29 (3H, d, *J* = 7.0, CH_3);

^{13}C NMR (75 MHz, $CDCl_3$) δ 145.9 (C_q), 127.8 (ArC_q), 127.0 (2 × C, ArC), 116.2 (ArC), 111.0 (ArC), 48.2 (C2), 39.2 (C4), 30.8 (NCH_3), 29.9 (C3), 22.7 (CH_3);

IR ν_{max} (neat)/cm^{-1}: 3028, 2956, 2926, 2864, 2818, 1602, 1501, 1447;

HRMS (ESI): $C_{11}H_{16}N$ [M+H$^+$]: calculated 162.1277, found 162.1274.

1-Methyl-4-phenyl-1,2,3,4-tetrahydroquinoline 2n

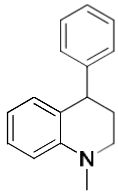

Following general procedure O, using chloroamine **1n** (100 mg, 0.39 mmol). Workup A followed by purification by flash chromatography on silica gel, eluting with 5% EtOAc in pentane afforded the *title compound* **2n** (57 mg, 0.26 mmol, 67%) as a colourless gum.

^{1}H NMR (300 MHz, $CDCl_3$) δ 7.34-7.09 (6H, m, ArH), 6.79-6.67 (2H, m, ArH), 6.62-6.53 (1H, m, ArH), 4.14 (1H, t, *J* = 6.2, H-C4), 3.28-3.12 (2H, m, H_2-C2), 2.95 (3H, s, NCH_3), 2.33-2.21 (1H, m, H_a-C3), 2.17-2.05 (1H, m, H_b-C3);

^{13}C NMR (75 MHz, $CDCl_3$) δ 146.5 (2 × C, ArC), 129.9 (ArC), 128.7 (2 × C, ArC), 128.3 (2 × C, ArC), 127.6 (ArC), 126.09 (ArC), 124.9 (ArC), 116.3 (ArC), 111.1 (ArC), 48.5 (C2), 43.4 (C4), 39.3 (NCH_3), 31.0 (C3);

IR ν_{max} (neat)/cm^{-1}: 3023, 2922, 2862, 2820, 1600, 1502, 1450, 1207;

HRMS (ESI): $C_{16}H_{18}N$ [M+H$^+$]: calculated 224.1434, found 224.1438.

1-Methyl-7-phenyl-1,2,3,4-tetrahydroquinoline 2o

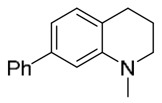

Following general procedure O, using chloroamine **1o** (100 mg, 0.39 mmol). Workup B followed by purification by flash chromatography on silica gel, eluting with 20% DCM in hexane afforded the *title compound* **2o** (63 mg, 0.28 mmol, 72%) as a colourless oil.

^{1}H NMR (400 MHz, $CDCl_3$) δ 7.56-7.47 (2H, m, ArH), 7.39-7.30 (2H, m, ArH), 7.28-7.21 (1H, m, ArH), 7.00-6.92 (1H, m, ArH), 6.80-6.70 (2H, m, ArH), 3.24-3.17 (2H, m, H_2-C2), 2.89 (3H, s, NCH_3), 2.74 (2H, t, *J* = 6.5, H_2-C4), 2.01-1.89 (2H, m, H_2-C3);

^{13}C NMR (100 MHz, $CDCl_3$) δ 147.1 (C_q), 142.5 (C_q), 140.5 (C_q), 129.3 (ArC), 128.7 (2 × C, ArC), 127.3 (2 × C, ArC), 127.0 (ArC), 122.2 (C_q), 115.4 (ArC), 110.0 (ArC), 51.5 (C2), 39.3 (NCH_3), 27.7 (C4), 22.6 (C3);

IR ν_{max} (neat)/cm^{-1}: 3054, 3028, 2924, 2837, 1678, 1605, 1561, 1515;

HRMS (ESI): $C_{16}H_{18}N$ [M+H$^+$]: calculated 224.1434, found 224.1434.

1,4,7-Trimethyl-1,2,3,4-tetrahydroquinoline 2p

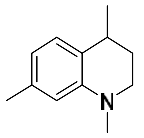

Following general procedure O, using chloroamine **1p** (100 mg, 0.47 mmol). Workup A afforded the *title compound* **2p** (54 mg, 0.31 mmol, 66%) as a colourless oil.

^{1}H NMR (500 MHz, $CDCl_3$) δ 6.97 (1H, d, *J* = 7.6, ArH-C5), 6.49 (1H, d, *J* = 7.6, ArH-C6), 6.45 (1H, s, ArH-C8), 3.29-3.16 (2H, m, H_2-C2), 2.93-2.85 (4H, m, includes 3H, s, NCH_3; and 1H, m, H-C4), 2.31 (3H, s, $ArCH_3$), 2.09-2.00 (1H, m, H_a-C3), 1.70 (1H, dtd, *J* = 10.3, 6.4, 4.0, H_b-C3), 1.29 (3H, d, *J* = 7.0, CH_3);

^{13}C NMR (125 MHz, $CDCl_3$) δ 146.0 (C_q), 136.5 (C_q), 127.7 (ArC), 125.2 (C_q), 117.0 (ArC), 111.7 (ArC), 48.3 (C2), 39.2 (NCH_3), 30.4 (C3), 30.2 (C4), 22.7 (CH_3), 21.5($ArCH_3$);

IR ν_{max} (neat)/cm^{-1}: 2954, 2921, 2855, 2812, 1611, 1572, 1507, 1484;

HRMS (ESI): $C_{12}H_{18}N$ [M+H$^+$]: calculated 176.1434, found 176.1428.

1-Methyl-7-trifluoromethyl-1,2,3,4-tetrahydroquinoline 2q

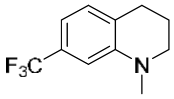

Following general procedure O, using chloroamine **1q** (100 mg, 0.40 mmol). Workup B, followed by purification by flash chromatography on silica gel, eluting with a gradient of 10% EtOAc in hexane afforded the title compound **2q** (26 mg, 0.12 mmol, 30%) as a colourless oil.

^{1}H NMR (400 MHz, $CDCl_3$) δ 7.01 (1H, d, *J* = 7.6, ArH-C5), 6.82 (1H, d, *J* = 7.6, ArH-C6), 6.74 (1H, s, ArH-C8), 3.30-3.25 (2H, m, H_2-C2), 2.92 (3H, s, NCH_3), 2.78 (2H, t, *J* = 6.3, H_2-C4), 2.02-1.95 (2H, m, H_2-C3);

^{13}C NMR (100 MHz, $CDCl_3$) δ 146.8 (C_q), 129.5 (q, *J* = 31.5, C_q), 128.9 (C5), 126.4 (C_q), 124.8 (d, *J* = 272.0, CF_3), 112.5 (q, *J* = 3.9, C6), 106.9 (q, *J* = 3.8, C8), 51.1 (C2), 39.0 (NCH_3), 27.9 (C4), 22.1 (C3);

IR ν_{max} (neat) / cm^{-1}:2933, 2843, 1615, 1582, 1510, 1488, 1467, 1445;

HRMS (ESI): $C_{22}H_{25}F_6N$ [$2M+H^+$]: calculated 431.1916, found 431.1903.

1,4,5-Trimethyl-1,2,3,4-tetrahydroquinoline 2r

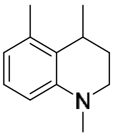

Following general procedure O, using chloroamine **1r** (100 mg, 0.47 mmol). Workup A afforded the *title compound* **2r** (58 mg, 0.33 mmol, 70%) as a yellow oil.

^{1}H NMR (300 MHz, $CDCl_3$) δ 6.99 (1H, dd, *J* = 8.1, 7.7, ArH-C7), 6.50 (2H, d, *J* = 7.7, ArH), 3.42 (1H, ddd, *J* = 13.0, 11.4, 3.7, H_a-C2), 3.20-3.05 (2H, m, includes 1H, m, H_b-C2; and 1H, m, H-C4), 2.93 (3H, s, NCH_3), 2.29 (3H, s, $ArCH_3$), 1.98 (1H, tt, *J* = 13.0, 5.0, H_a-C3), 1.72 (1H, ddt, *J* = 13.0, 3.7, 2.4, H_b-C3), 1.17 (3H, d, *J* = 7.0, CH_3);

^{13}C NMR (125 MHz, $CDCl_3$) δ 145.4 (C_q), 135.5 (AC_q), 126.5 (ArC), 125.6 (C_q), 118.3 (ArC), 108.8 (ArC), 45.9 (C2), 39.3 (NCH_3), 28.3 (C3), 27.3 (C4), 20.7 (CH_3), 19.0 ($ArCH_3$);

IR ν_{max} (neat)/cm^{-1}: 2926, 2861, 2813, 1617, 1509, 1463, 1412, 1373;

HRMS (ESI): $C_{12}H_{18}N$ [$M+H^+$]: calculated 176.1434, found 176.1432.

1-Methyl-7-chloro-1,2,3,4-tetrahydroquinoline 2s

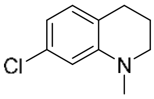

Following general procedure O, using chloroamine **1s** (100 mg, 0.46 mmol). Workup B, followed by purification by flash chromatography on silica gel, eluting with a gradient of 10% EtOAc in hexane afforded the title compound **2s** (33 mg, 0.18 mmol, 39%) as a colourless oil.

^{1}H NMR (400 MHz, $CDCl_3$) δ 6.83 (1H, d, *J* = 7.8, ArH-C5), 6.57-6.50 (2H, m, includes 1H, m, ArH-C6; and 1H, m, ArH-C8), 3.26-3.17 (2H, m, H_2-C2), 2.87 (3H, s, NCH_3), 2.70 (2H, t, *J* = 6.4, H_2-C4), 1.99-1.91 (2H, m, H_2-C3);

^{13}C NMR (100 MHz, $CDCl_3$) δ 147.6 (C_q), 132.6 (C_q), 129.6 (ArC), 121.2 (C_q), 115.7 (ArC), 110.6 (ArC), 51.0 (C2), 39.1 (NCH_3), 27.4 (C4), 22.3 (C3);

IR ν_{max} (neat) / cm^{-1}: 3022, 2929, 2890, 2840, 1599, 1564, 1502, 1466;

HRMS (ESI): $C_{10}H_{13}^{35}ClN$ [$M+H^+$]: calculated 182.0731, found 182.0723.

7-Bromo-1,4-dimethyl-1,2,3,4-tetrahydroquinoline 2t

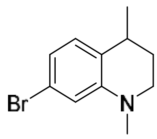

Following general procedure O, using chloroamine **1t** (100 mg, 0.36 mmol). Workup A, followed by purification by flash chromatography on silica gel, eluting with 10% EtOAc in hexane afforded the *title compound* **2t** (46 mg, 0.19 mmol, 53%) as a yellow oil.

^{1}H NMR (500 MHz, $CDCl_3$) δ 6.88 (1H, d, *J* = 8.0, ArH-C5), 6.73 (1H, dd, *J* = 8.0, 1.9, ArH-C6), 6.67 (1H, d, *J* = 1.9, ArH-C8), 3.30-3.17 (2H, m, H_2-C2), 2.88 (3H, s, NCH_3), 2.82 (1H, dt, *J* = 13.1, 6.5, H-C4), 1.99 (1H, ddt, *J* = 13.2, 8.6, 4.8, H_a-C3), 1.67 (1H, dtd, *J* = 10.4, 6.4, 4.1, H_b-C3), 1.25 (3H, d, *J* = 7.0, CH_3);

^{13}C NMR (125 MHz, $CDCl_3$) δ 147.1 (C_q), 128.8 (ArC), 126.6 (C_q), 120.7 (C_q), 118.5 (ArC), 113.2 (ArC), 47.9 (C2), 38.9 (NCH_3), 30.5 (C4), 29.5 (C3), 22.2 (CH_3);

IR ν_{max} (neat)/cm^{-1}: 2956, 2923, 2852, 1593, 1557, 1498, 1408, 1303;

HRMS (ESI): $C_{11}H_{15}^{81}BrN$ [$M+H^+$]: calculated 242.0362, found 242.0355.

1-Methyl-7-(tetramethyl-1,3,2-dioxaborolan-2-yl)-1,2,3,4-tetrahydroquinoline 2u

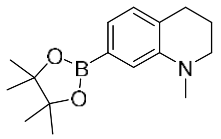

To a stirred solution of chloroamine **1u** (100 mg, 0.32 mmol) in DCM (1.07 mL) was added $MeSO_3H$ (0.21 mL, 3.23 mmol, 10.0 eq.). The reaction mixture was irradiated with a high pressure 125 W Hg lamp for 3 h, after which it was diluted with DCM (5 mL) and extracted with H_2O (5 mL). The layers were separated and the aqueous layer was basified with sat. $NaHCO_3$ solution (10 mL), then extracted with EtOAc (3 × 10 mL). The combined organic extracts were

washed with brine (10 mL), dried over Na_2SO_4 and concentrated *in vacuo*. Purification by flash chromatography on silica gel, eluting with a gradient of 10% EtOAc in hexane afforded the *title compound* **2u** (41 mg, 0.15 mmol, 47%) as a yellow oil.

^{1}H NMR (400 MHz, $CDCl_3$) δ 7.08 (1H, d, *J* = 7.3, ArH), 7.03 (1H, s, ArH-C8), 6.97 (1H, d, *J* = 7.3, ArH), 3.24-3.18 (2H, m, H_2-C2), 2.93 (3H, s, NCH_3), 2.77 (2H, t, *J* = 6.5, H_2-C4), 1.97 (2H, app. dt, *J* = 12.0, 6.3, H_2-C3), 1.33 (12H, s, 2 × $OC(CH_3)_2$);

^{13}C NMR (100 MHz, $CDCl_3$) δ 146.4 (C_q), 128.5 (ArC), 126.7 (C_q), 123.2 (ArC), 117.0 (ArC), 83.6 (2 × C, O*C*$(CH_3)_2$), 51.5 (C2), 39.4 (NCH_3), 28.2 (C4), 25.0 (4 × C, OC(*C*$H_3)_2$), 22.5 (C3), one C_q signal missing;

IR ν_{max} (neat)/cm^{-1}: 2976, 2930, 2838, 1695, 1605, 1561, 1510, 1480;

HRMS (ESI): $C_{16}H_{25}BNO_2$ [M+H$^+$]: calculated 274.1973, found 274.1980.

1-Methyl-5-phenyl-1,2,3,4-tetrahydroquinoline 2v

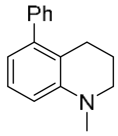

Following general procedure O, using chloroamine **1v** (100 mg, 0.39 mmol). Workup B followed by purification by flash chromatography on silica gel, eluting with 10% EtOAc in hexane afforded the title compound **2v** (65 mg, 0.29 mmol, 74%) as a clear yellow gum.

^{1}H NMR (500 MHz, $CDCl_3$) δ 7.41-7.35 (2H, m, ArH), 7.34-7.29 (3H, m, ArH), 7.16-7.10 (1H, m, ArH), 6.64 (1H, d, *J* = 8.2, ArH), 6.58 (1H, d, *J* = 7.5, ArH), 3.28-3.22 (2H, m, H_2-C2), 2.96 (3H, s, NCH_3), 2.62 (2H, t, *J* = 6.4, H_2-C4), 1.91-1.83 (2H, m, H_2-C3);

^{13}C NMR (125 MHz, $CDCl_3$) δ 147.0 (C_q), 142.6 (C_q), 141.9 (C_q), 129.3 (2 × C, ArC), 128.0 (2 × C, ArC), 126.7 (ArC), 126.6 (ArC), 120.8 (C_q), 118.3 (ArC), 110.2 (ArC), 51.3 (C2), 39.7 (NCH_3), 26.4 (C4), 22.7 (C3);

IR ν_{max} (neat)/cm^{-1}: 3055, 2921, 2850, 2818, 2786, 1579, 1483, 1461;

HRMS (ESI): $C_{16}H_{18}N$ [M+H$^+$]: calculated 224.1434, found 224.1434.

1,4,6-Trimethyl-1,2,3,4-tetrahydroquinoline and 1,4,8-trimethyl-1,2,3,4-tetrahydroquinoline 2w

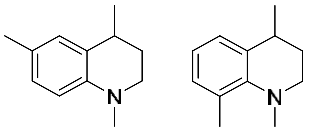

2.5:1

Following general procedure O, using chloroamine **1w** (100 mg, 0.47 mmol). Workup A afforded an inseparable mixture of the regioisomeric *title compounds* **2w** (57 mg, 0.33 mmol, 70%, 2.5:1) as a brown oil.

^{1}H NMR (500 MHz, $CDCl_3$) δ 7.06 (0.30H, d, *J* = 7.6, ArH, *minor*), 7.02 (0.30H, d, *J* = 7.3, ArH, *minor*), 6.94-6.86 (1.7H, m, includes 1.4H, m, ArH, *major*, and 0.3H, m, ArH, *minor*), 6.56 (0.70H, d, *J* = 7.9, ArH, *major*), 3.24-3.06 (2H, m, includes 1.4H, m, H_2-C2, *major*, and 0.6 H, m, H_2-C2, *minor*), 2.99-2.85 (3.1H, m, includes 2.1H, s, NCH_3, *major*, 0.7H, m, H-C4, *major*, and 0.3H, m, H-C4, *minor*), 2.74 (0.9H, s, NCH_3, *minor*), 2.33 (0.9H, s, $ArCH_3$, *minor*), 2.26 (2.1H, s, $ArCH_3$, *major*), 2.10-1.96 (1H, m, includes 0.7H, m, H_a-C3, *major*, and 0.3H, m, H_a-C3, *minor*), 1.76-1.67 (0.7H, m, H_b-C3, *major*), 1.63-1.55 (0.3H, m, H_b-C3, *minor*), 1.32 (0.9H, d, *J* = 7.1, CH_3, *minor*), 1.30 (2.1H, d, *J* = 7.0, CH_3, *major*);

^{13}C NMR (125 MHz, $CDCl_3$) δ 147.5 (C_q), 144.1 (C_q), 134.3 (C_q), 131.1 (C_q), 128.9 (ArC), 128.6 (ArC), 128.2 (C_q), 127.4 (ArC), 126.5 (ArC), 125.3 (C_q), 121.5 (ArC), 111.32 (ArC), 49.3 (CH_2), 48.4 (CH_2), 42.9 (NCH_3), 39.5 (NCH_3), 31.1 (CH), 30.7 (CH), 30.3 (CH_2), 25.1 (CH_2), 23.4 (CH_3), 22.9 (CH_3), 20.3 ($ArCH_3$), 18.8 ($ArCH_3$);

IR ν_{max} (neat)/cm^{-1}: 2959, 2923, 2862, 2820, 1589, 1492, 1467, 1449;

HRMS (ESI): $C_{12}H_{18}N$ [M+H$^+$]: calculated 176.1434, found 176.1428.

1-Methyl-6-chloro-1,2,3,4-tetrahydroquinoline and 1-methyl-8-chloro-1,2,3,4-tetrahydroquinoline 2x

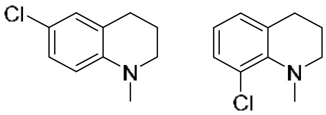

1.1:1

Following general procedure O, using chloroamine **1x** (100 mg, 0.46 mmol). Workup B, followed by purification by flash chromatography on silica gel, eluting with a gradient of 10% EtOAc in hexane afforded an inseparable mixture of regioisomeric title compounds **2x** (47 mg, 0.26 mmol, 57%, 1.1:1).

^{1}H NMR (400 MHz, $CDCl_3$) δ 7.20-7.15 (0.52H, m, ArH, *major*), 7.00 (0.48H, dd, *J* = 8.7, 2.6, ArH, *minor*), 6.97-6.94 (0.52H, m, ArH, *major*), 6.92-6.90 (0.48H, m, ArH, *minor*), 6.82 (0.52H, t, *J* = 7.7, ArH, *major*), 6.48 (0.48H, d, *J* = 8.7, ArH, *minor*), 3.22-3.18 (0.96H, m, H_2-C2, *minor*), 3.16-3.12 (1.04H, m, H_2-C2, *major*), 2.89 (1.56H, s, NCH_3, *major*), 2.86 (1.44H, s, NCH_3, *minor*), 2.80 (1.04H, t, *J* = 6.7, H_2-C4, *major*), 2.73 (0.96H, t, *J* = 6.5, H_2-C4, *minor*), 1.96 (0.96H, ddd, *J* = 12.8, 9.1, 4.6, H_2-C3, *minor*), 1.86 (1.04H, dtd, *J* = 10.8, 6.7, 2.8, H_2-C3, *major*);

^{13}C NMR (100 MHz, $CDCl_3$) δ 146.1 (C_q), 145.4 (C_q), 131.4 (C_q), 128.5 (ArC), 128.4 (ArC), 128.3 (ArC), 127.6 (C_q), 126.8 (ArC), 124.6 (C_q), 122.2 (ArC), 120.8 (C_q), 112.0 (ArC), 52.1 (CH_2), 51.2 (CH_2), 43.0 (CH_3), 39.3 (CH_3), 28.0 (CH_2), 27.8 (CH_2), 22.3 (CH_2), 17.3 (CH_2);

IR ν_{max} (neat) / cm^{-1}: 2935, 2861, 1596, 1561, 1501, 1464, 1443, 1416;

HRMS (ESI): $H_{10}C_{13}{}^{35}ClN$ [M+H$^+$]: calculated 182.0731, found 182.0728.

1H,2H,3H,3aH,4H,5H-Hexahydropyrrolo[1,2-a]quinoline 5a

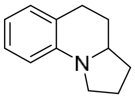

Following general procedure O, using chloroamine **4a** (100 mg, 0.48 mmol). Workup B followed by purification by flash chromatography on silica gel, eluting with 10% EtOAc in hexane afforded the *title compound* **5a** (62 mg, 0.36 mmol, 75%) as a yellow oil. The data is in accordance with the literarture.[24]

^{1}H NMR (500 MHz, $CDCl_3$) δ 7.09 (1H, t, *J* = 7.7, ArH-C8), 7.00 (1H, d, *J* = 7.3, ArH-C6), 6.57 (1H, t, *J* = 7.3, ArH-C7), 6.42 (1H, d, *J* = 8.0, ArH-C9), 3.44 (1H, tdd, *J* = 10.7, 5.1, 3.1, H-C3a), 3.34 (1H, td, *J* = 9.0, 2.1, H_a-C5), 3.24 (1H, dd, *J* = 16.6, 9.1, H_b-C5), 2.94-2.84 (1H, m, H_a-C1), 2.78 (1H, ddd, *J* = 16.0, 4.5, 2.3, H_b-C1), 2.19-2.03 (3H, m, includes 2H, m, H_2-C2; and 1H, m, H_a-C4), 2.01-1.88 (1H, m, H_b-C4), 1.56-1.40 (2H, m, H_2-C3);

^{13}C NMR (125 MHz, $CDCl_3$) δ 144.8 (C_q), 128.4 (ArC), 127.1 (ArC), 121.2 (C_q), 114.7 (ArC), 109.9 (ArC), 58.0 (C3a), 46.9 (C5), 33.2 (C1), 28.2 (C4), 27.4 (C2), 23.9 (C3);

IR ν_{max} (neat)/cm^{-1}: 3019, 2933, 2837, 1602, 1573, 1502, 1458, 1386;

HRMS (ESI): $C_{12}H_{16}N$ [M+H$^+$]: calculated 174.1277, found 174.1270.

1H,2H,3H,4H,4aH,5H,6H-Hexahydropyrido[1,2-a]quinoline 5b

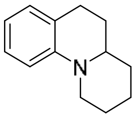

Following general procedure O, using chloroamine **4b** (100 mg, 0.45 mmol). Workup B followed by purification by flash chromatography on silica gel, eluting with 10% EtOAc in hexane afforded the title compound **5b** (70 mg, 0.37 mmol, 82%) as a pale yellow oil. The data is in accordance with the literature.[25]

^{1}H NMR (500 MHz, $CDCl_3$) δ 7.08 (1H, td, *J* = 8.2, 1.5, ArH-C9), 6.97 (1H, dd, *J* = 7.3, 0.5, ArH-C7), 6.83 (1H, d, *J* = 8.3, ArH-C10), 6.66 (1H, td, *J* = 7.3, 0.9, ArH-C8), 3.99-3.90 (1H, m, H_a-C6), 2.93-2.79 (2H, m, includes 1H, m, H-C4a, and 1H, m, H_a-C1), 2.73-2.63 (2H, m, includes 1H, m, H_b-C6, and 1H, m, H_b-C1), 1.95 (1H, dtd, *J* = 13.2, 5.2, 4.0, H_a-C4), 1.88-1.55 (5H, m, includes 1H, H_b-C4; 2H, m, H_2-C2; 1H, m, H_a-C3, and 1H, m, H_a-C5), 1.50-1.38 (2H, m, includes 1H, H_b-C3, and 1H, m, H_b-C5);

^{13}C NMR (125 MHz, $CDCl_3$) δ 147.1 (C_q), 129.2 (ArC), 127.1 (ArC), 125.1 (C_q), 117.4 (ArC), 112.9 (ArC), 57.1 (C4a), 48.3 (C6), 33.5 (C5), 30.5 (C4), 27.2 (C1), 26.0 (C2), 24.7 (C3);

IR ν_{max} (neat)/cm^{-1}: 3067, 3016, 2927, 2846, 2795, 1602, 1576, 1492;

HRMS (ESI): $C_{13}H_{18}N$ [M+H$^+$]: calculated 188.1434, found 188.1434.

(4a*R**,9a*R**)-10-Methyl-1,2,3,4,4a,9,9a,10-octahydroacridine 7

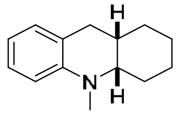

Following general procedure O, using chloroamine **6** (150 mg, 0.63 mmol). Work-up A followed by purification by flash chromatography on silica gel, eluting with 10% DCM in hexane afforded the title compound **7** (73.0 mg, 0.36 mmol, 57%) as a yellow gum. The data is in accordance with the literature.[20]

^{1}H NMR (500 MHz, $CDCl_3$) δ 7.06 (1H, t, *J* = 7.7, ArH-C6), 6.95 (1H, d, *J* = 7.3, ArH-C8), 6.57 (1H, t, *J* = 7.2, ArH-C7), 6.50 (1H, d, *J* = 8.2, ArH-C5), 3.16 (1H, app. dt, *J* = 10.7, 3.7, H-C4a), 2.97 (1H, dd, *J* = 16.1, 12.3, H_a-C9), 2.90 (3H, s, NCH_3), 2.51 (1H, dd, *J* = 16.2, 5.2, H_b-C9), 2.32-2.23 (1H, m, H-C9a), 1.81-1.61 (4H, m, includes 1H, m, H_a-C4; and 1H, m, H_a-C2, and 2H, m, H_2-C1), 1.55-1.22 (4H, m, incluces 1H, m, H_b-C4; and 1H, m, H_b-C2, and 2H, m, H_2-C3);

^{13}C NMR δ (125 MHz, $CDCl_3$) 144.8 (C_q), 128.9 (ArC), 127.0 (ArC), 121.2 (C_q), 115.2 (ArC), 109.8 (ArC), 61.0 (C9a), 36.9 (NCH_3), 31.7 (C4a), 29.9 (C9), 28.5 (C4), 25.7 (C1), 24.9 (C2), 20.6 (C3);

IR ν_{max} (neat)/cm^{-1}: 2917, 2851, 2829, 1602, 1572, 1491, 1287, 1198;

HRMS (ESI): $C_{14}H_{20}N$ [M+H$^+$]: calculated 202.1596, found 202.1597.

(1*S**,9*S**)-8-methyl-8-azatricyclo[7.3.1.0^{2,7}]trideca-2(7),3,5-triene 9

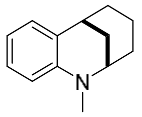

Following general procedure O, using chloroamine **8** (100 mg, 0.45 mmol) at 0.1 M final concentration. Workup B followed by purification by flash chromatography on silica gel, eluting with 10% DCM in hexane afforded the *title compound* **9** (34 mg, 0.18 mmol, 40%) as a colourless oil.

^{1}H NMR (400 MHz, $CDCl_3$) δ 7.13-7.06 (1H, m, ArH-C5), 6.94 (1H, d, *J* = 7.2, ArH-C3), 6.59-6.52 (2H, m, includes 1H, m, ArH-C4; and 1H, m, ArH-C6), 3.44 (1H, s, H-C9), 3.01-2.92 (4H, m, includes 3H, s, NCH_3; and 1H, s, H-C1), 1.99 (1H, d, *J* = 12.8, H_a-C10), 1.87 (2H, s, H_2-C13), 1.76-1.67 (2H, m, H_2-C12), 1.50-1.37 (2H, m, includes 1H, m, H_b-C10; and 1H, m, H_a-C11), 1.30-1.19 (1H, m, H_b-C11);

^{13}C NMR (100 MHz, $CDCl_3$) δ 147.5 (C_q), 128.1 (ArC), 127.3 (ArC), 126.3 (C_q), 114.8 (ArC), 108.6 (ArC), 54.9 (C9), 37.1 (C1), 34.9 (C12), 34.1 (NCH_3), 31.1 (C10), 30.0 (C13), 17.7 (C11);

IR ν_{max} (neat)/cm^{-1}: 3065, 3016, 2925, 2899, 2844, 1600, 1571, 1498;

HRMS (ESI): $C_{13}H_{18}N$ [M+H$^+$]: calculated 188.1434, found 188.1428.

1,7-dimethyl-4-phenyl-1,2,3,4-tetrahydroquinoline 12a and 1-methyl-4-(*p*-tolyl)-1,2,3,4-tetrahydroquinoline 12b

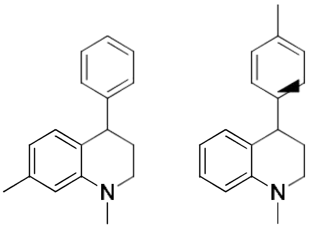

10.4:1

Following general procedure O, using chloroamine **10** (100 mg, 0.37 mmol). Workup A followed by purification by flash chromatography on silica gel, eluting with a gradient of 2-10% EtOAc in pentane afforded an inseparable 91:9 mixture of the regioisomeric *title compounds* (57 mg, 0.24 mmol, 65%) as a colourless gum. Ratio determined by comparison of aromatic signals in the ^{1}H NMR spectrum; only signals for the major isomer reported.

^{1}H NMR (500 MHz, $CDCl_3$) δ 7.34-7.27 (2H, m, ArH), 7.24-7.18 (1H, m, ArH), 7.15-7.19 (2H, m, ArH), 6.64 (1H, d, *J* = 7.5, ArH), 6.51 (1H, s, ArH), 6.41 (1H, d, *J* = 7.5, ArH), 4.11 (1H, t, *J* = 6.2, H-C4), 3.25-3.12 (2H, m, H_2-C2), 2.95 (3H, s, NCH_3), 2.31 (3H, s, $ArCH_3$), 2.29-2.21 (1H, m, H_a-C3), 2.14-2.04 (1H, m, H_b-C3);

^{13}C NMR (125 MHz, $CDCl_3$) δ 146.7 (2 × C, C_q), 137.1 (C_q), 129.8 (ArC), 128.6 (2 × C, ArC), 128.2 (2 × C, ArC), 126.0 (ArC), 122.1 (C_q), 117.1 (ArC), 111.8 (ArC), 48.6 (C2), 43.1 (C4), 39.3 (NCH_3), 31.3 (C3), 21.6 ($ArCH_3$);

IR ν_{max} (neat)/cm^{-1}: 3024, 2918, 2853, 2813, 1610, 1567, 1507, 1490;

HRMS (ESI): $C_{17}H_{20}N$ [M+H$^+$]: calculated 238.1590, found 238.1585.

1-methyl-4-(3-(trifluoromethyl)phenyl)-1,2,3,4-tetrahydroquinoline 13a

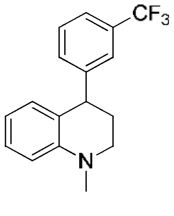

Following general procedure O, using chloroamine **11** (100 mg, 0.31 mmol). Workup A afforded the *title compound* **13a** (67 mg, 0.23 mmol, 74%) as a brown gum.

^{1}H NMR (500 MHz, $CDCl_3$) δ 7.50-7.35 (3H, m, ArH), 7.31-7.24 (1H, m, ArH), 7.18-7.11 (1H, m, ArH), 6.76-6.67 (2H, m, ArH), 6.62-6.54 (1H, m, ArH), 4.20 (1H, dd, *J* = 12.8, 6.6, H-C4),

3.28-3.21 (1H, m, H_a-C2), 3.19-3.12 (1H, m, H_b-C2), 2.96 (1H, s, NCH_3), 2.33-2.24 (1H, m, H_a-C3), 2.15-2.07 (1H, m, H_b-C3);

^{13}C NMR (100 MHz, $CDCl_3$) δ 147.7 (C_q), 147.0 (C_q), 132.3 (ArC), 130.8 (q, *J* = 32.0, C_q), 129.9 (ArC), 128.9 (ArC), 128.1 (ArC), 125.4 (q, *J* = 3.7, ArC), 124.4 (q, *J* = 272.4, CF_3), 123.9 (C_q), 123.2 (q, *J* = 3.7, ArC), 116.5 (ArC), 111.4 (ArC), 48.5 (C2), 43.5 (C4), 39.3 (NCH_3), 31.2 (C3);

IR ν_{max} (neat)/cm^{-1}: 3027, 2946, 2824, 1602, 1504, 1445, 1324, 1207;

HRMS (ESI): $C_{17}H_{17}F_3N$ [M+H$^+$]: calculated 292.1308, found 292.1310.

5-Chloro-1-methyl-1,2,3,4-tetrahydroquinoline 15

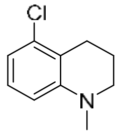

Following general procedure B, using chloroamine **14** (100 mg, 0.40 mmol). Workup B followed by purification by flash chromatography on silica gel, eluting with 10% EtOAc in hexane afforded the *title compound* **15** (37 mg, 0.20 mmol, 50%) as a clear yellow oil.

^{1}H NMR (400 MHz, $CDCl_3$) δ 7.02-6.94 (1H, m, ArH), 6.71-6.65 (1H, m, ArH), 6.51-6.45 (1H, m, ArH), 3.23-3.16 (2H, m, H_2-C2), 2.89 (3H, s, NCH_3), 2.81 (2H, t, *J* = 6.6, H_2-C4), 2.03-2.95 (2H, m, H_2-C3);

^{13}C NMR (100 MHz, $CDCl_3$) δ 148.4 (C_q), 134.5 (C_q), 127.3 (ArC), 120.6 (C_q), 117.1 (ArC), 109.5 (ArC), 51.0 (C2), 39.7 (NCH_3), 25.5 (C4), 22.1 (C3);

IR ν_{max} (neat)/cm^{-1}: 2942, 2863, 2820, 1589, 1563, 1490, 1461, 1445;

HRMS (ESI): $C_{10}H_{13}{}^{35}ClN$ [M+H$^+$]: calculated 182.0731, found 182.0731.

1,4,5,8-Tetramethyl-1,2,3,4-tetrahydroquinoline 17

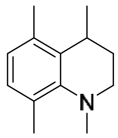

Following general procedure O, using chloroamine **16** (100 mg, 0.44 mmol). Workup B followed by purification by flash chromatography on silica gel, eluting with 50% DCM in hexane afforded the *title compound* **17** (34 mg, 0.18 mmol, 41%) as a colourless oil.

^{1}H NMR (500 MHz, $CDCl_3$) δ 6.93 (1H, d, *J* = 7.6, ArH), 6.72 (1H, d, *J* = 7.6, ArH), 3.23 (1H, td, *J* = 12.9, 2.8, H_a-C2), 3.14-3.08 (1H, m, H_b-C2), 3.08-3.03 (1H, m, H-C4), 2.73 (3H, s, NCH_3), 2.30 (3H, s, $ArCH_3$), 2.29 (3H, s, $ArCH_3$), 2.06 (1H, tdd, *J* = 13.0, 5.3, 4.0, H_a-C3), 1.52 (1H, ddd, *J* = 13.3, 5.3, 2.9, H_b-C3), 1.19 (3H, d, *J* = 7.0, CH_3);

^{13}C NMR (125 MHz, $CDCl_3$) δ 147.3 (C_q), 133.6 (C_q), 132.5 (C_q), 128.8 (ArC), 128.1 (C_q), 123.4 (ArC), 47.3 (C2), 43.9 (NCH_3), 28.1 (C4), 25.1 (C3), 21.3 (CH_3), 19.2 ($ArCH_3$), 18.9 ($ArCH_3$);

IR ν_{max} (neat)/cm^{-1}: 2929, 2864, 2787, 1737, 1578, 1460, 1397, 1370;

HRMS (ESI): $C_{13}H_{20}N$ [M+H^{+}]: calculated 190.1590, found 190.1586.

1.5 One-pot procedures

Synthesis of 1-methyl-1,2,3,4-tetrahydroquinoline 2a

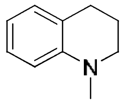

To a stirred solution of amine **3** (100 mg, 0.67 mmol, 1.0 eq.) in DCM (2.24 mL) was added NCS (89 mg, 0.67 mmol, 1.0 eq.) and the reaction mixture was stirred for 0.5 h. After this $MeSO_3H$ (0.44 mL, 6.70 mmol, 10 eq.) was added and the reaction mixture was irradiated for 3 h. The reaction mixture was extracted with H_2O (10 mL) and the aqueous phase was basified with 2M aqueous NaOH (10 mL) then extracted with EtOAc (3 × 10 mL). The combined organic extracts were washed with brine (10 mL), dried over Na_2SO_4 then concentrated *in vacuo*. Purification by flash chromatography on silica gel, eluting with a gradient of 10% EtOAc in hexane afforded the title compound **2a** (59 mg, 0.40 mmol, 60%) as a yellow oil.

Synthesis of 1H,2H,3H,3aH,4H,5H-pyrrolo[1,2-a]quinolone 5a

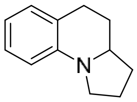

To a stirred solution of amine **4a** (100 mg, 0.57 mmol, 1.0 eq.) in DCM (1.9 mL) was added NCS (76 mg, 0.57 mmol, 1.0 eq.) and the reaction mixture was stirred for 0.5 h. After this $MeSO_3H$ (0.37 mL, 5.70 mmol, 10 eq.) was added and the reaction mixture was irradiated for 3 h. The reaction mixture was extracted with H_2O (10 mL) and the aqueous phase was basified with 2M aqueous NaOH (10 mL) then extracted with EtOAc (3 × 10 mL). The combined organic extracts were washed with brine (10 mL), dried over Na_2SO_4 then concentrated *in vacuo*. Purification by flash chromatography on silica gel, eluting with a gradient of 10% EtOAc in hexane afforded the title compound **6a** (73 mg, 0.42 mmol, 73%) as a yellow oil.

1.6 Angustureine 11 stereoselective total synthesis

(*S*)-2-Methyl-*N*-[(3*R*)-1-phenyloctan-3-yl]propane-2-sulfinamide

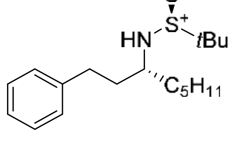

Following general procedure H, using (*S*,*E*)-2-Methyl-*N*-(3-phenylpropylidene)propane-2-sulfinamide (1.00 g, 4.21 mmol) and pentylmagensium bromide (6.3 mL of a 1.0 M solution in

THF). Purification by flash chromatography on silica gel, eluting with 20% EtOAc in hexane afforded an inseparable mixture of the diastereoisomeric *title compound* (1.01 g, 3.28 mmol, 78%, 85:15) as a yellow oil. Ratio of diastereoisomers determined by comparison of *tert*-butyl signals in the ^{1}H NMR spectrum.

^{1}H NMR (400 MHz, $CDCl_3$; signals reported for major isomer only) δ 7.46-7.05 (5H, m, ArH), 3.29 (1H, app. dq, *J* = 13.0, 6.4, octyl H-C3), 3.04 (1H, d, *J* = 6.6, NH), 2.82-2.71 (1H, m, octyl H_a-C1), 2.66-2.57 (1H, m, octyl H_b-C1), 1.96-1.82 (1H, m, octyl H_a-C2), 1.83-1.73 (1H, m, octyl H_b-C2), 1.67-1.56 (2H, m, octyl H_2-C4), 1.69-1.10 (15H, m, includes 6H, m, octyl H_2-C5-7; and 9H, s, $C(CH_3)_3$), 0.95-0.85 (3H, m, octyl H_3-C8)

^{13}C NMR (100 MHz, $CDCl_3$) δ 142.2 (C_q), 128.6 (2 × C, ArC), 128.5 (2 × C, ArC), 126.0 (C_q), 56.5 (octyl C3), 55.9 (C_q), 37.6 (octyl C2), 36.5 (octyl C4), 32.0 (octyl C1), 31.9 (CH_2), 25.5 (CH_2), 22.9 (3 × C, C($C$$H_3$)$_3$), 22.7 ($CH_2$), 14.2 (octyl C8);

IR ν_{max} (neat)/cm^{-1}: 3215 (N-H), 3085, 3062, 3027, 2953, 1603, 1495, 1455;

HRMS: (ESI): $C_{18}H_{32}NOS$ [M+H^{+}]: calculated 310.2199, found 310.2196.

(*R*)-Methyl(1-phenyloctan-3-yl)amine

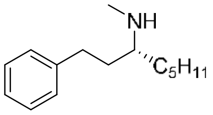

Following general procedure I, using (*S*)-2-Methyl-*N*-[(3*R*)-1-phenyloctan-3-yl]propane-2-sulfinamide (850 mg, 2.75 mmol). Purification by SCX cartridge afforded the title compound (544 mg, 2.48 mmol, 90%) as a clear yellow oil.

^{1}H NMR (400 MHz, $CDCl_3$) δ 7.31-7.14 (5H, m, ArH), 2.63 (2H, dd, *J* = 9.7, 6.7, octyl H_2-C1), 2.49-2.42 (1H, m, octyl H-C3), 2.39 (3H, s, NCH_3), 1.75-1.66 (2H, m, octyl H_2-C2), 1.48-1.39 (2H, m. octyl H_2-C4), 1.36-1.24 (6H, m, octyl H_2-C5-7), 0.94-0.83 (3H, m, octyl H_3-C8);

^{13}C NMR (100 MHz, $CDCl_3$) δ 142.9 (C_q), 128.5 (4 × C, ArC), 125.8 (ArC4), 58.9 (octyl C3), 35.5 (octyl C1), 33.7 (octyl C4), 33.5 (NCH_3), 32.3 (octyl C2), 32.2 (CH_2), 25.5 (CH_2), 22.8 (CH_2), 14.2 (octyl C8);

IR ν_{max} (neat)/cm^{-1}: 3062, 3026, 2926, 2856, 2788, 1603, 1495, 1454;

HRMS (ESI): $C_{15}H_{26}N$ [M+H^{+}]: calculated 220.2060, found 220.2064.

1-Methyl-2-pentyl-1,2,3,4-tetrahydroquinoline (*R*)-2j

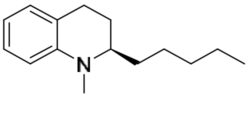

To a stirred solution of amine (100 mg, 0.46 mmol, 1.0 eq.) in DCM (1.84 mL) was added NCS (61 mg, 0.46 mmol, 1.0 eq.). The reaction mixture was stirred in the dark for 30 mins, after which

$MeSO_3H$ was added (0.29 mL, 4.60 mmol, 10 eq.) and the reactor was covered in aluminium foil and a red Perspex box was placed around it, then the reaction mixture was irradiated with UV light using a 125 W Hg lamp at rt for 3 h. The reaction was then diluted with H_2O (5 mL) and washed with EtOAc (10 mL). The aqueous phase was then basified with 2 M aqueous NaOH (10 mL) and extracted with EtOAc (3 × 10 mL). The combined organic extracts were washed with brine (10 mL), dried over Na_2SO_4 and concentrated *in vacuo*. Purification by flash chromatography on silica gel, eluting with 10% EtOAc in hexane afforded the title compound (*R*)-**2j** (52 mg, 0.24 mmol, 52%) as a clear yellow oil.

[□]$^{23}_{D}$ -5.2 (0.1, DCM)

1.7 ^{1}H and ^{13}C NMR spectra for *N*-chloroamines

^{1}H NMR, 300 MHz, $CDCl_3$ **1a**

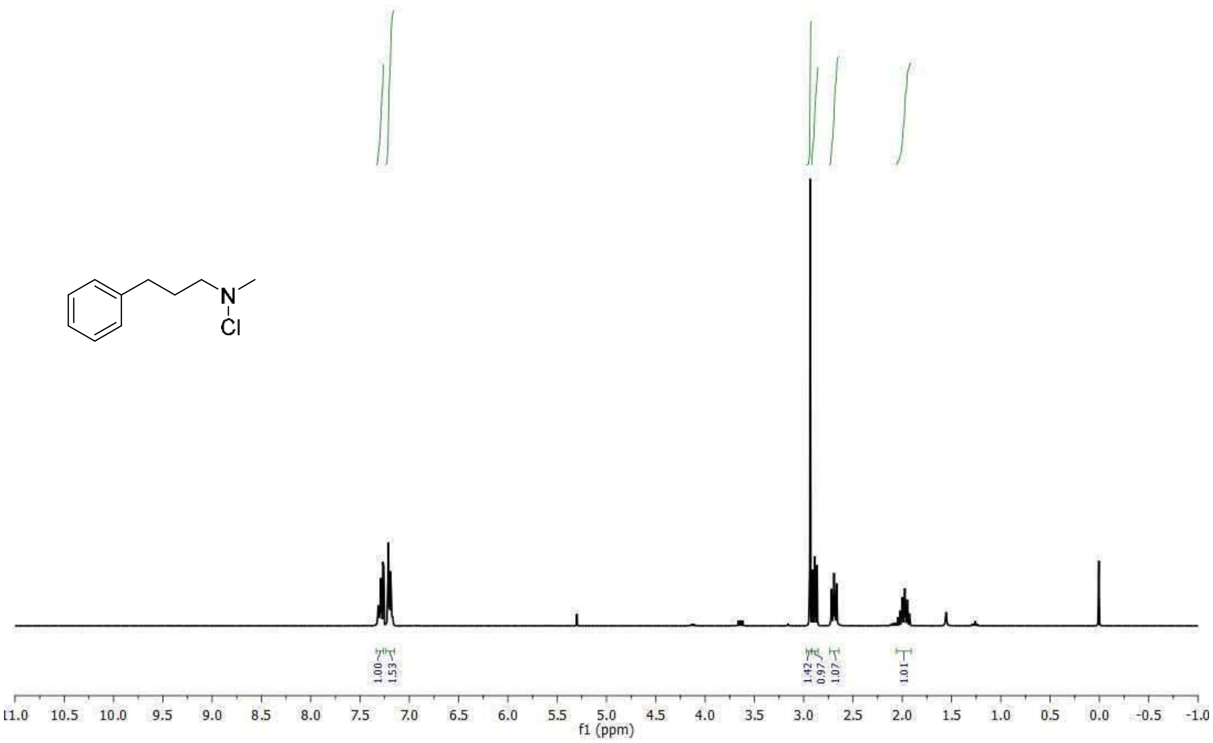

^{13}C NMR, 75 MHz, $CDCl_3$ **1a**

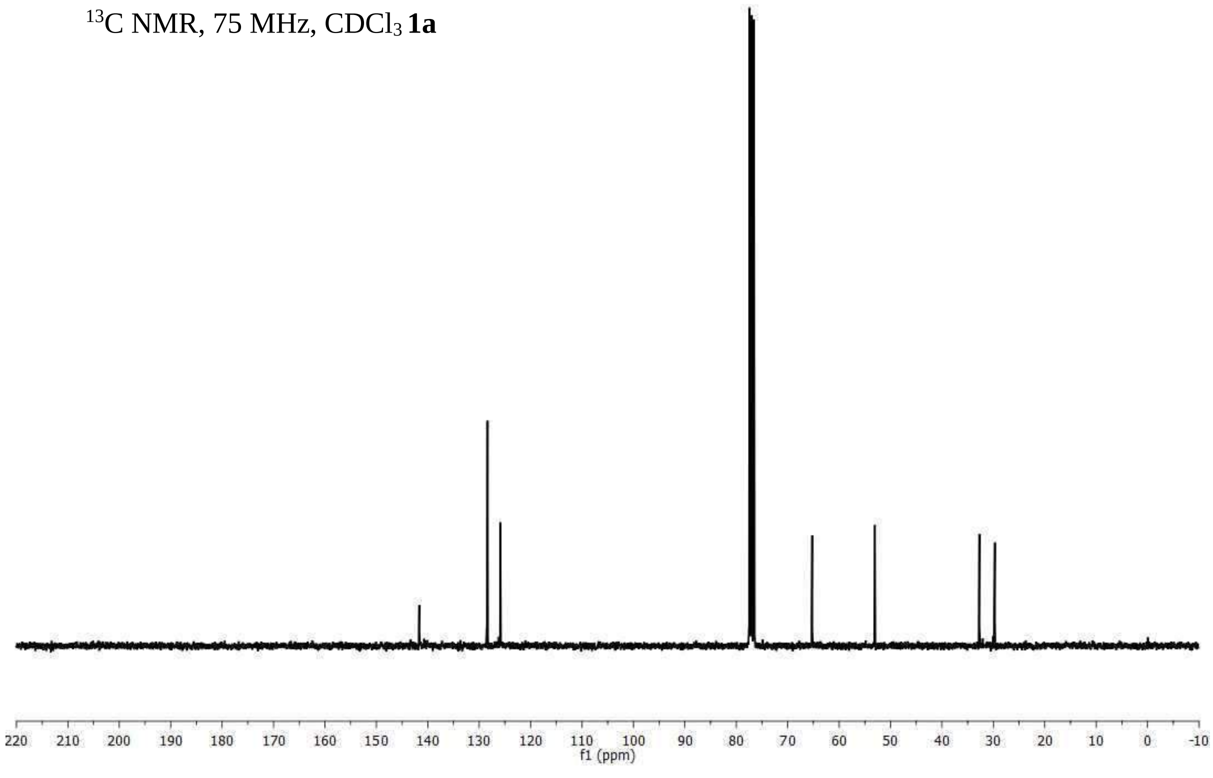

^{1}H NMR, 300 MHz, $CDCl_3$ **1b**

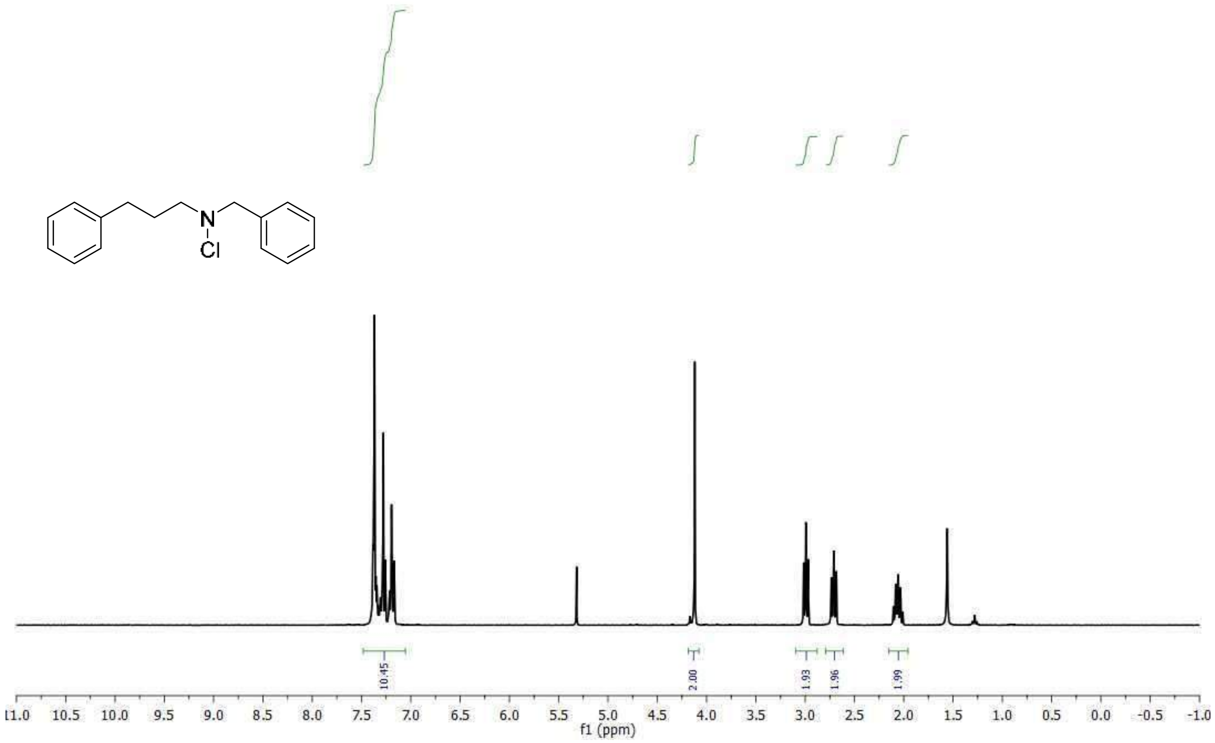

^{13}C NMR, 75 MHz, $CDCl_3$ **1b**

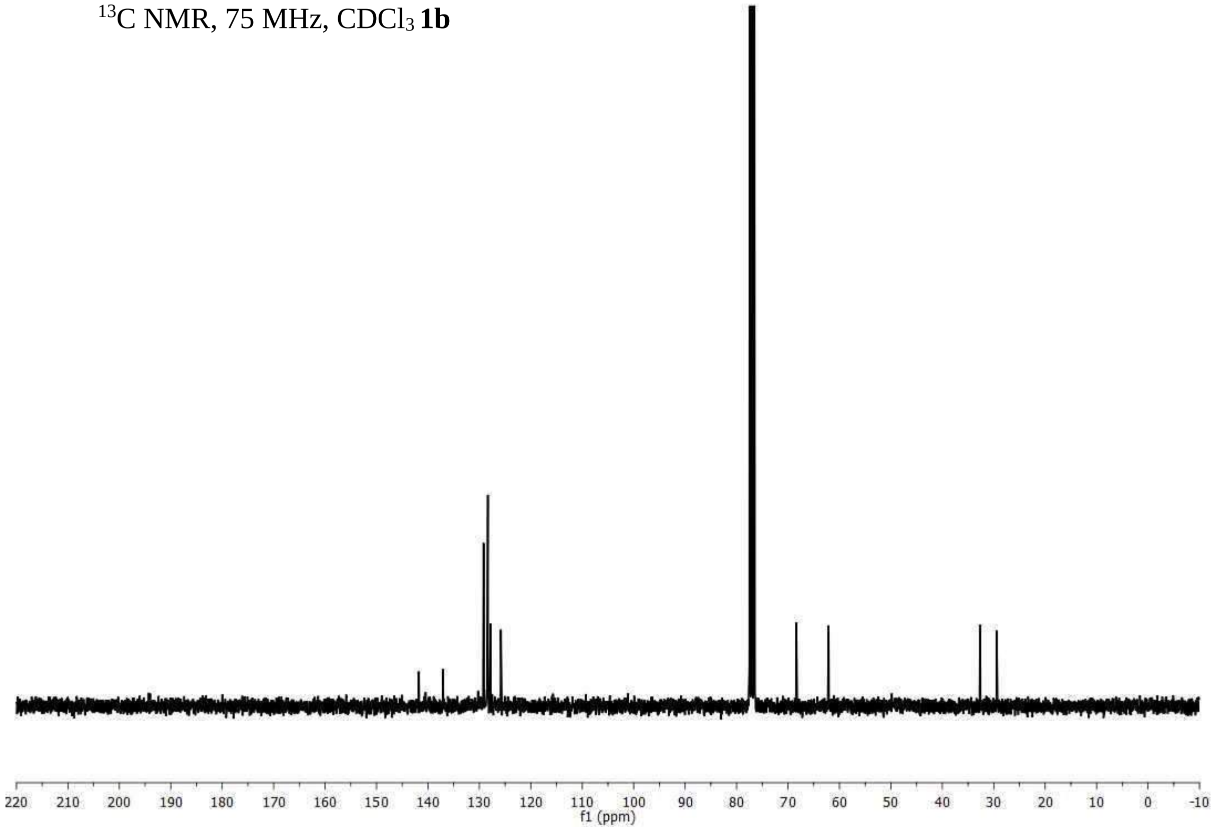

^{1}H NMR, 300 MHz, $CDCl_3$ **1c**

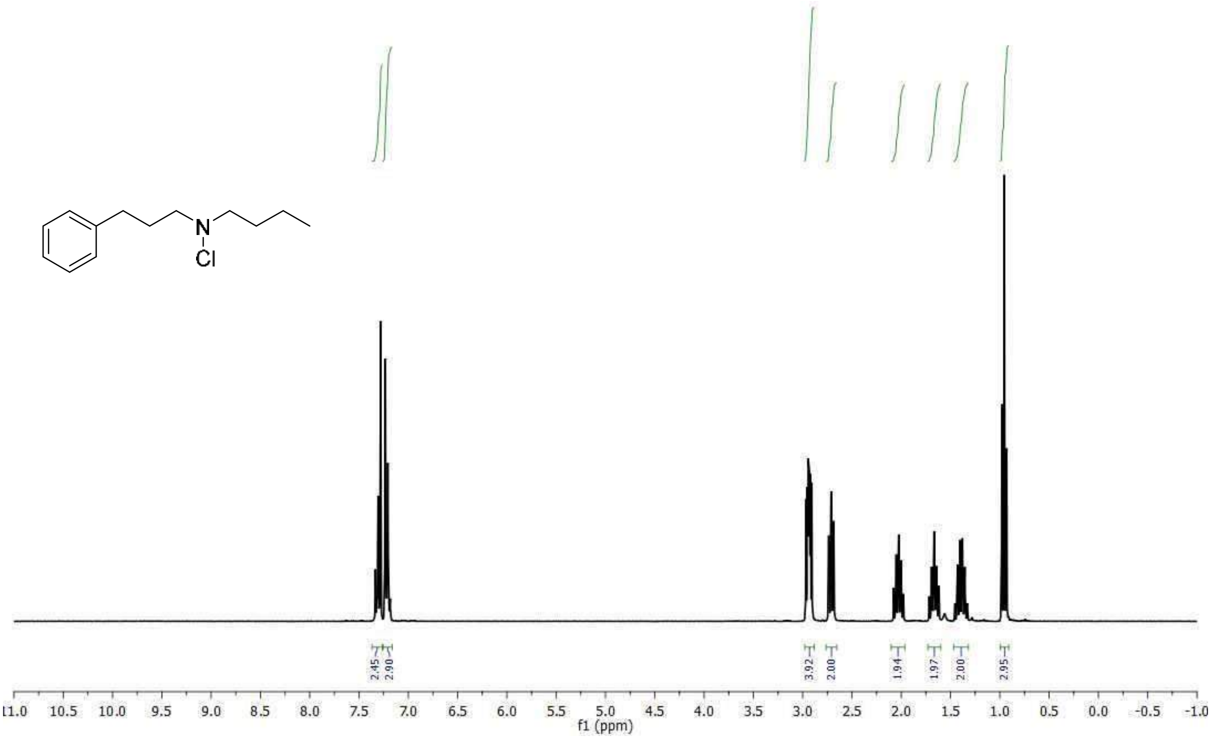

^{13}C NMR, 75 MHz, $CDCl_3$ **1c**

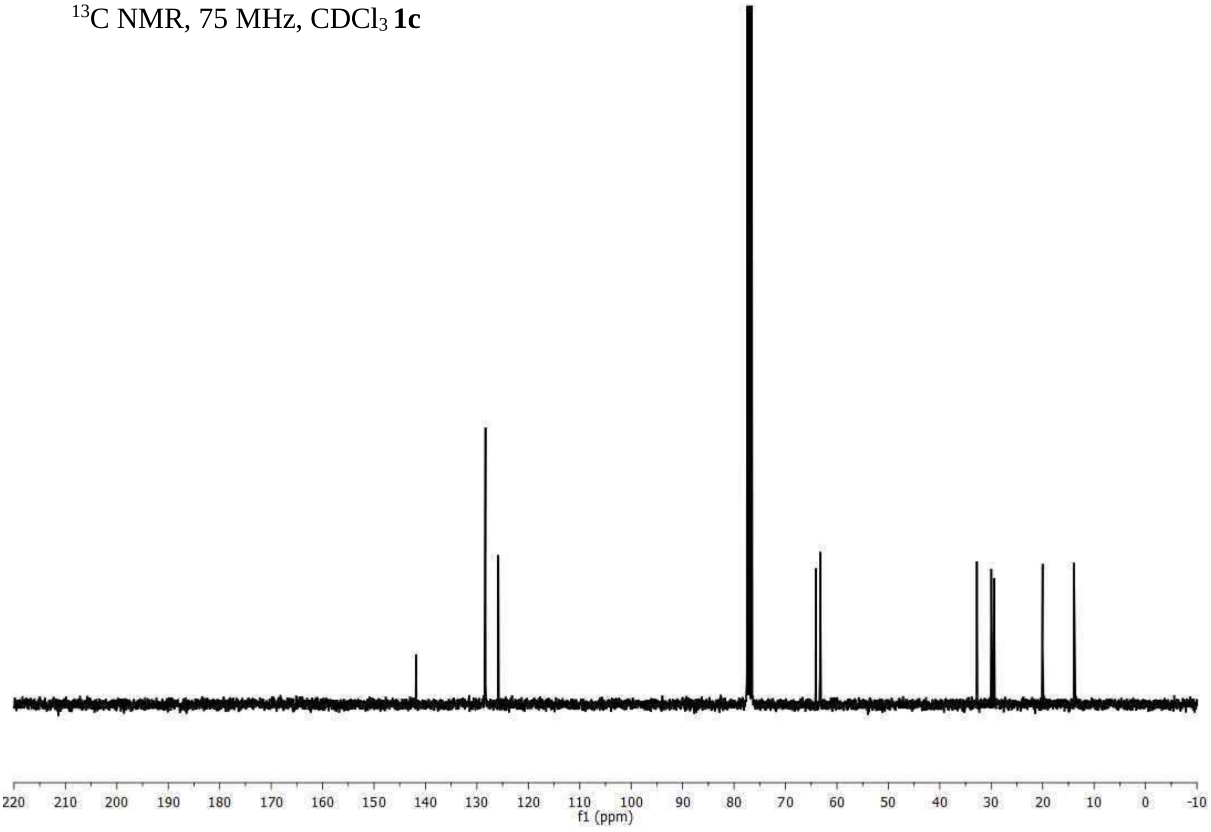

^{1}H NMR, 300 MHz, $CDCl_3$ **1d**

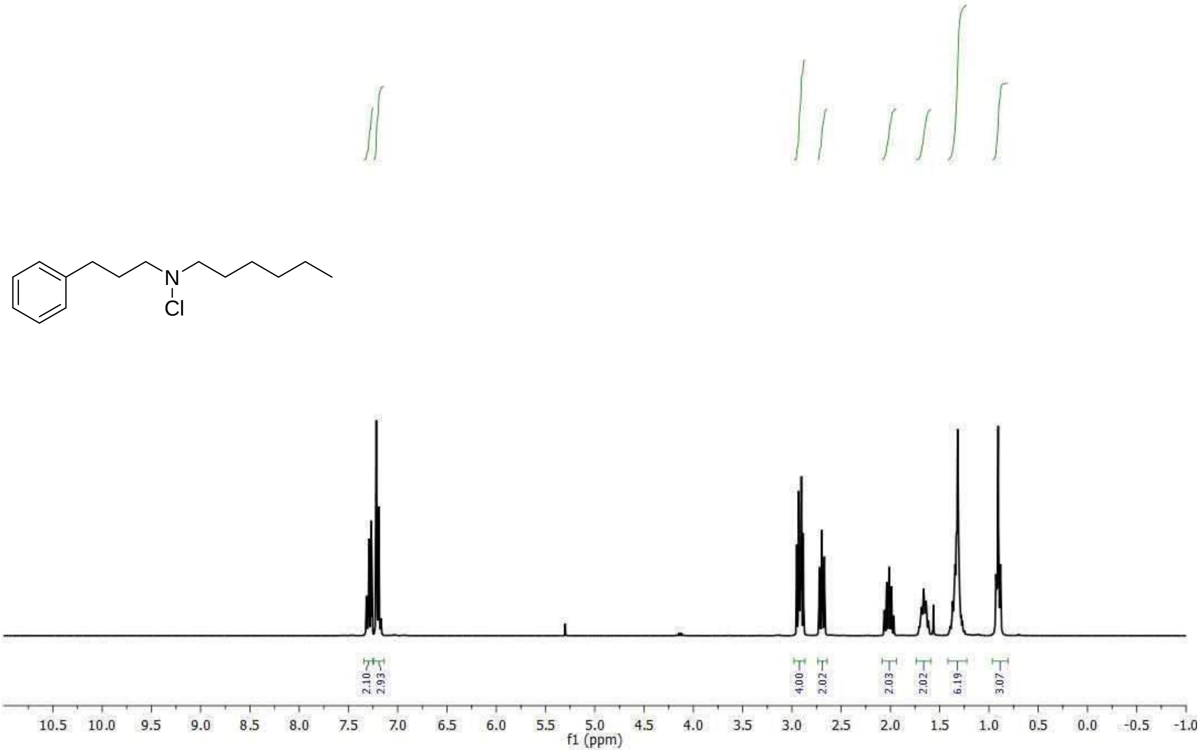

^{13}C NMR, 75 MHz, $CDCl_3$ **1d**

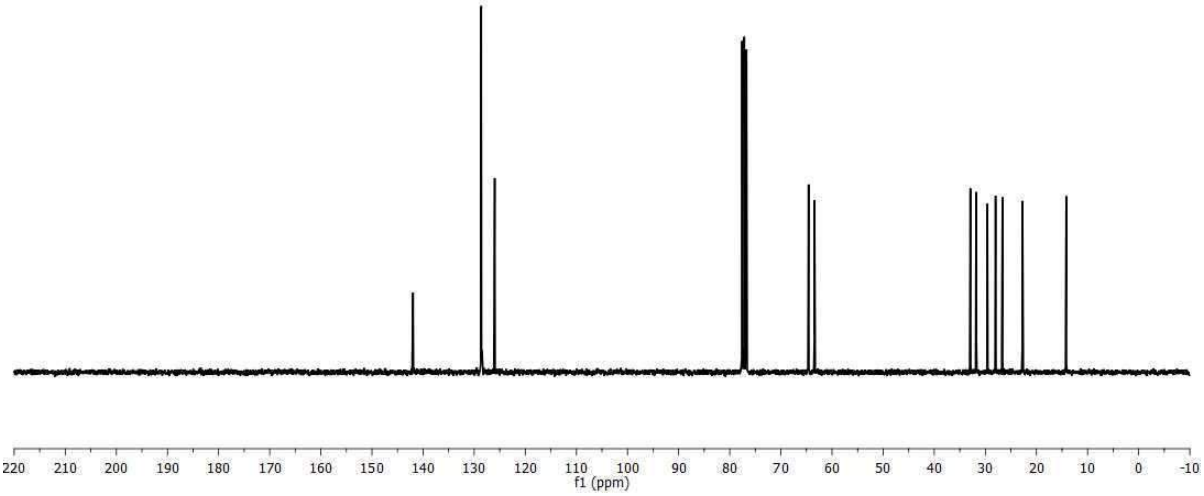

^{1}H NMR, 500 MHz, $CDCl_3$ **1e**

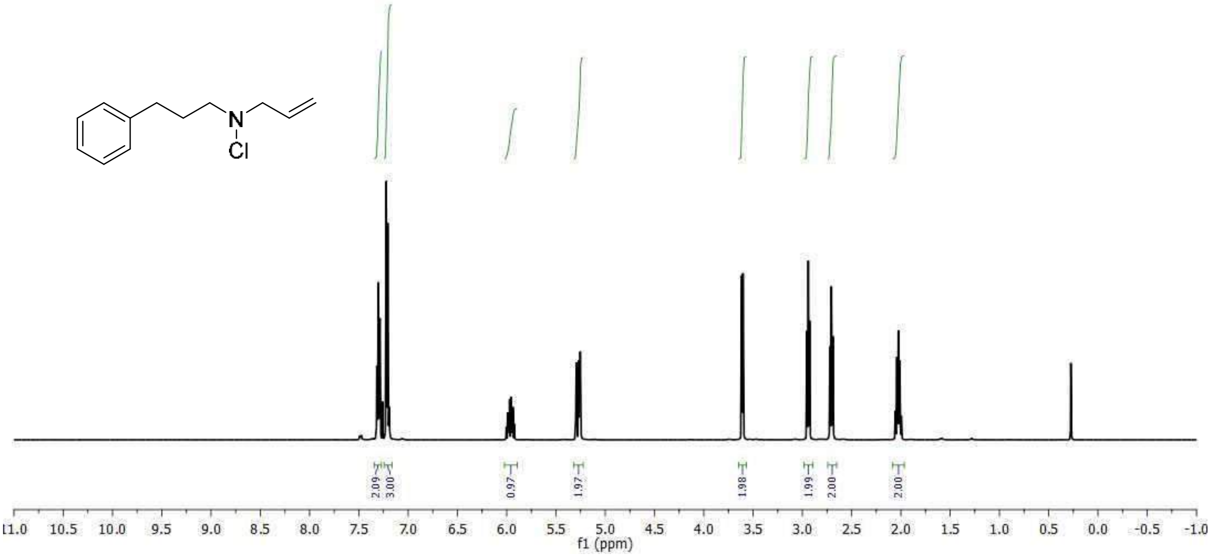

^{13}C NMR, 125 MHz, $CDCl_3$ **1e**

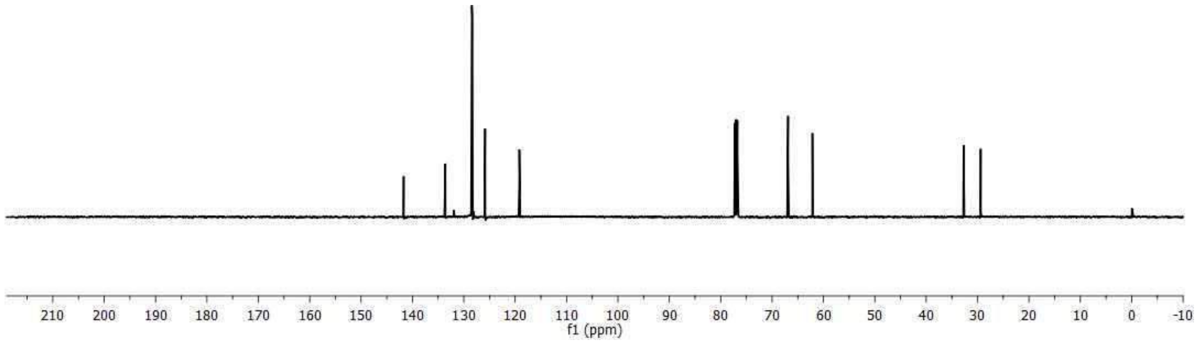

^{1}H NMR, 500 MHz, $CDCl_3$ **1f**

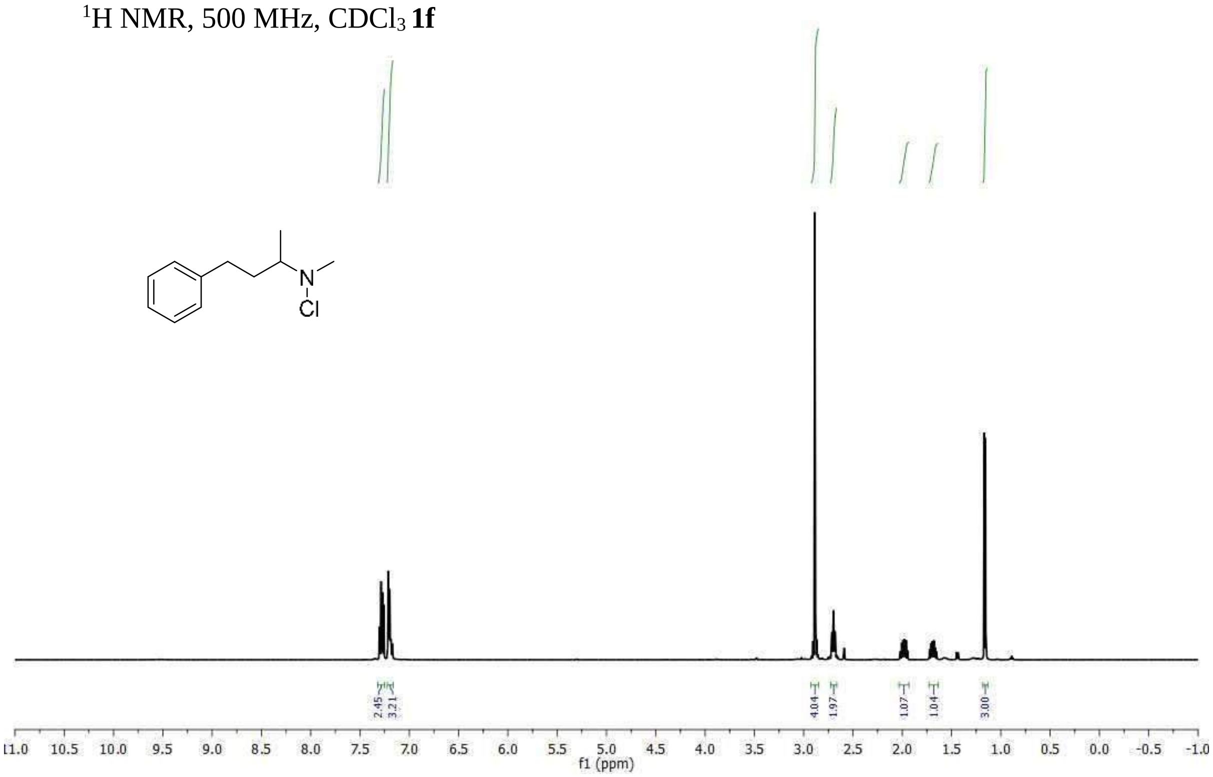

^{13}C NMR, 125 MHz, $CDCl_3$ **1f**

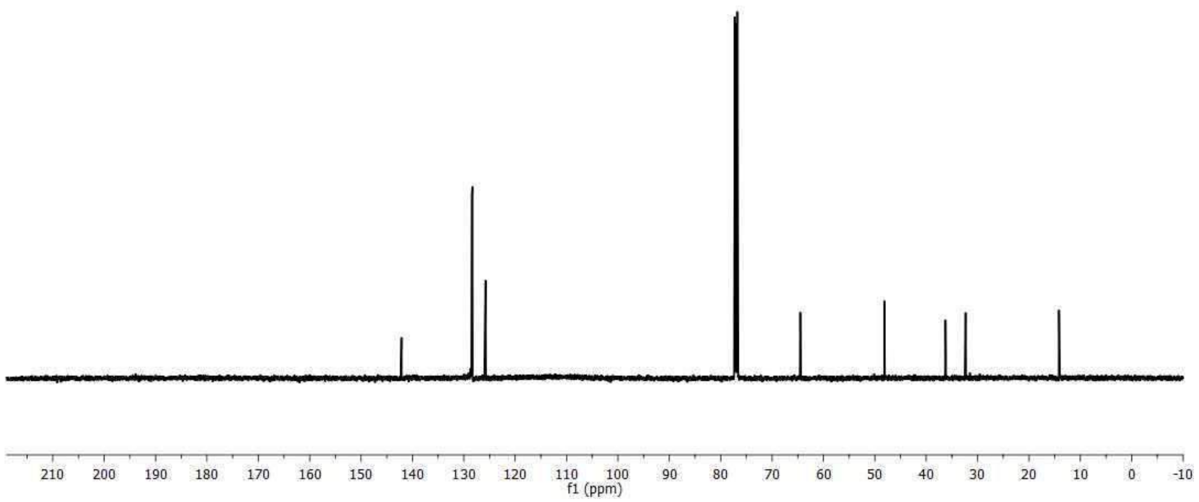

^{1}H NMR, 400 MHz, $CDCl_3$ **1g**

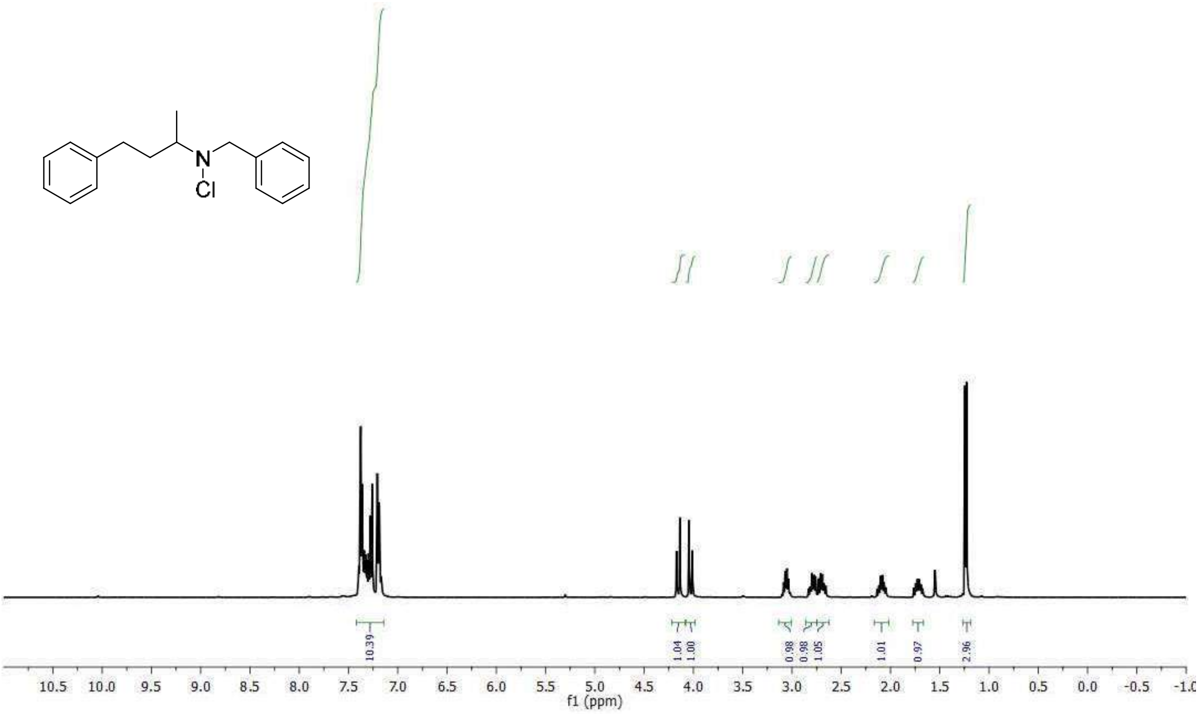

^{13}C NMR, 100 MHz, $CDCl_3$ **1g**

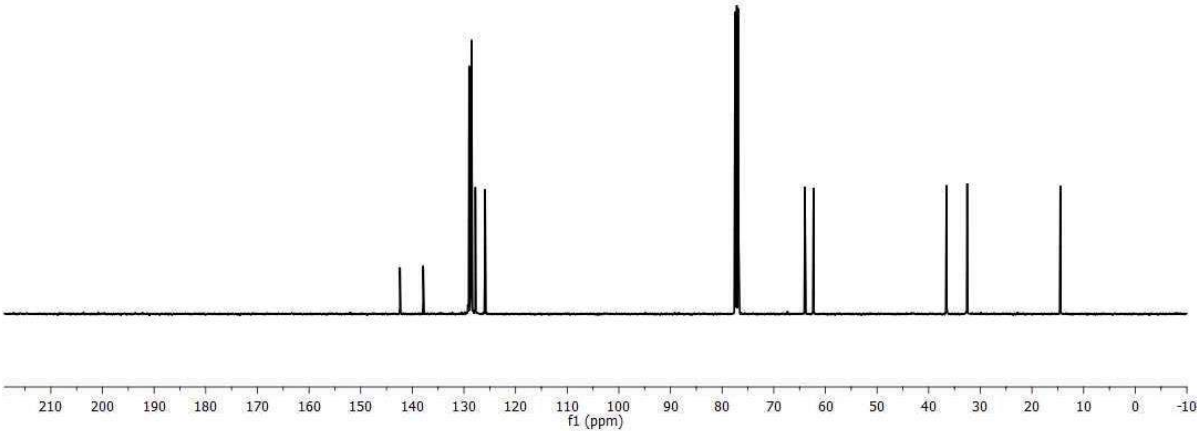

^{1}H NMR, 500 MHz, $CDCl_3$ **1h**

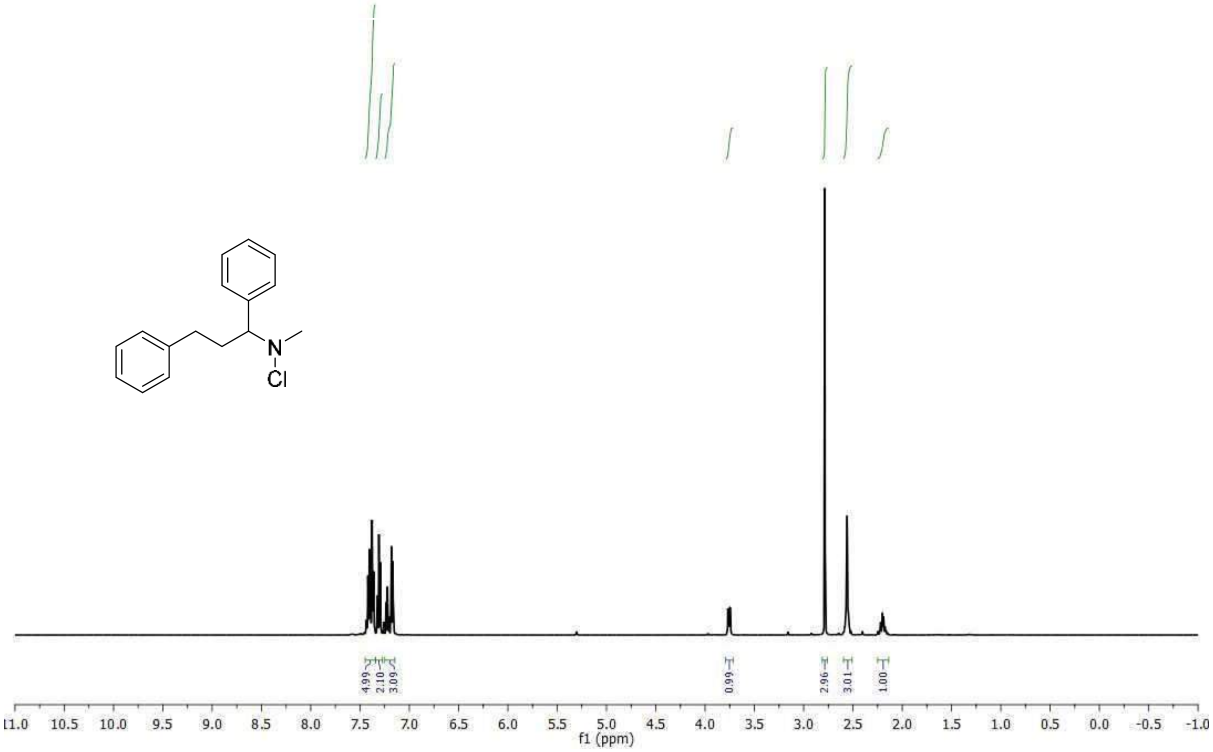

^{13}C NMR, 125 MHz, $CDCl_3$ **1h**

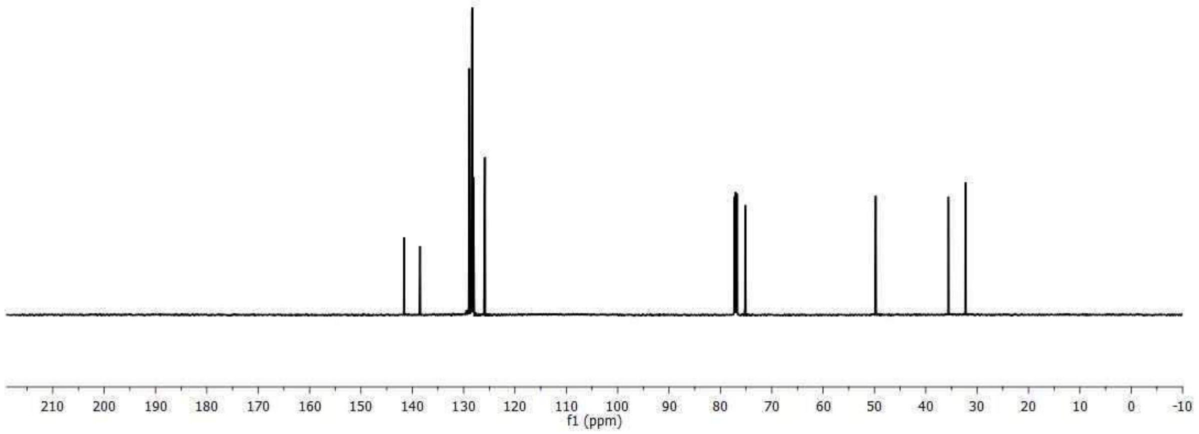

^{1}H NMR, 500 MHz, $CDCl_3$ **1i**

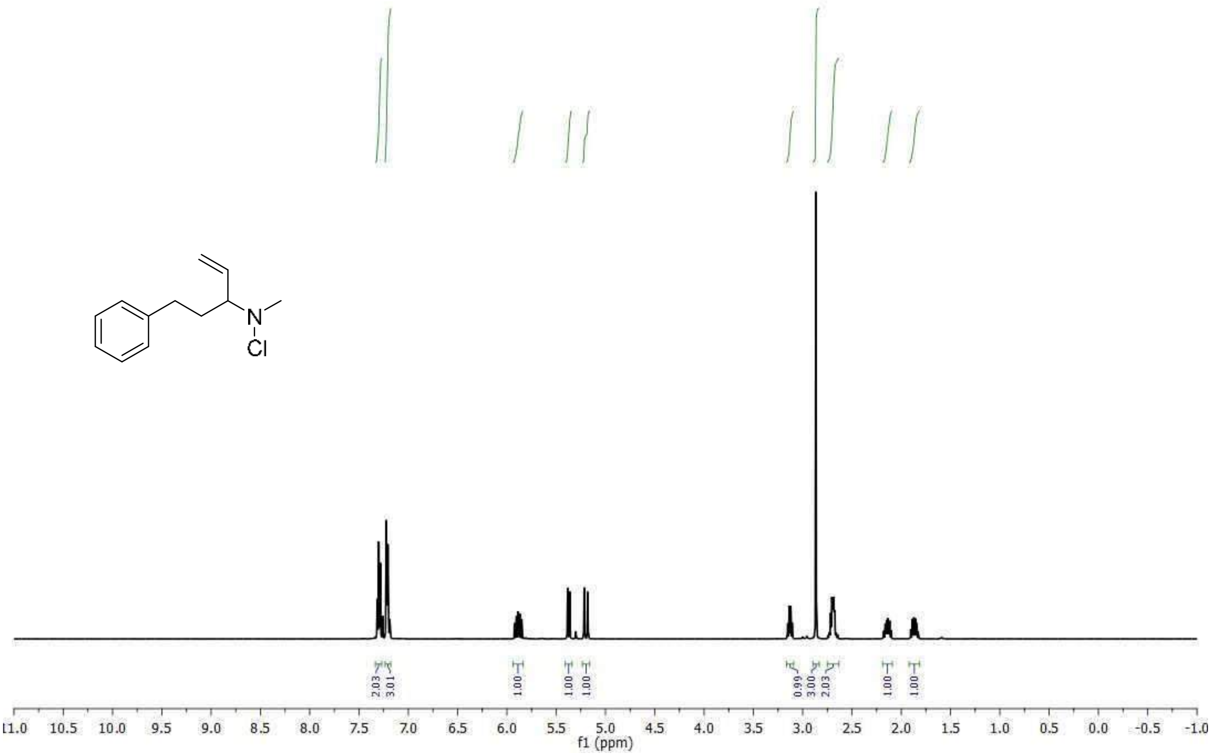

^{13}C NMR, 125 MHz, $CDCl_3$ **1i**

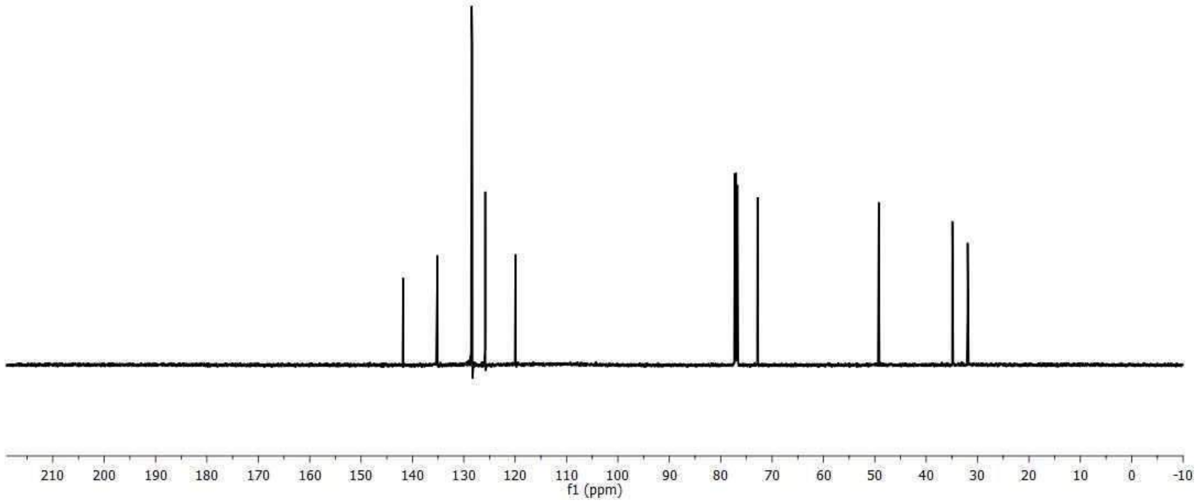

^{1}H NMR, 400 MHz, $CDCl_3$ **1k**

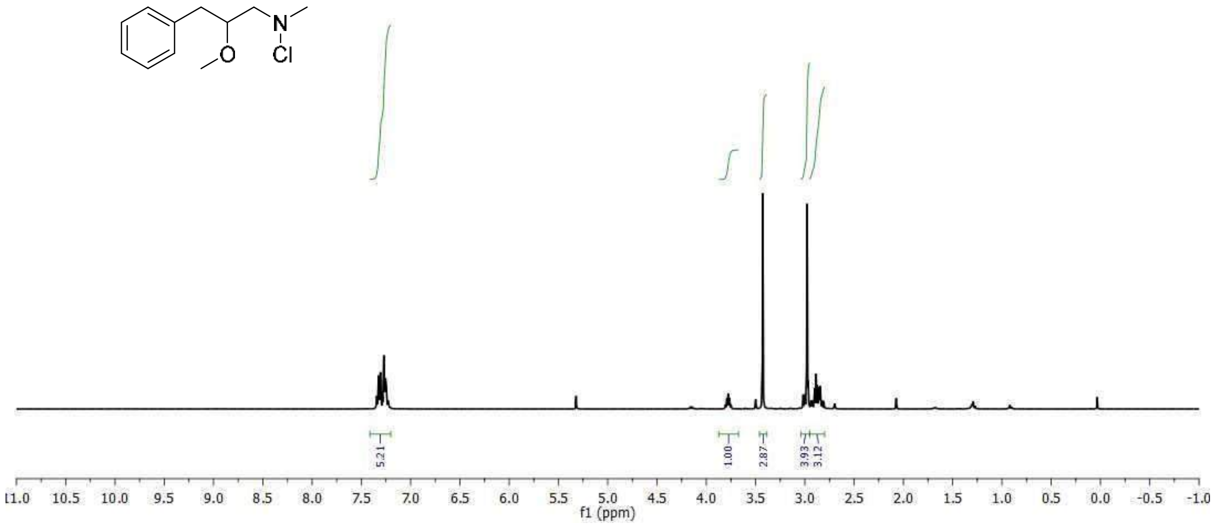

^{13}C NMR, 100 MHz, $CDCl_3$ **1k**

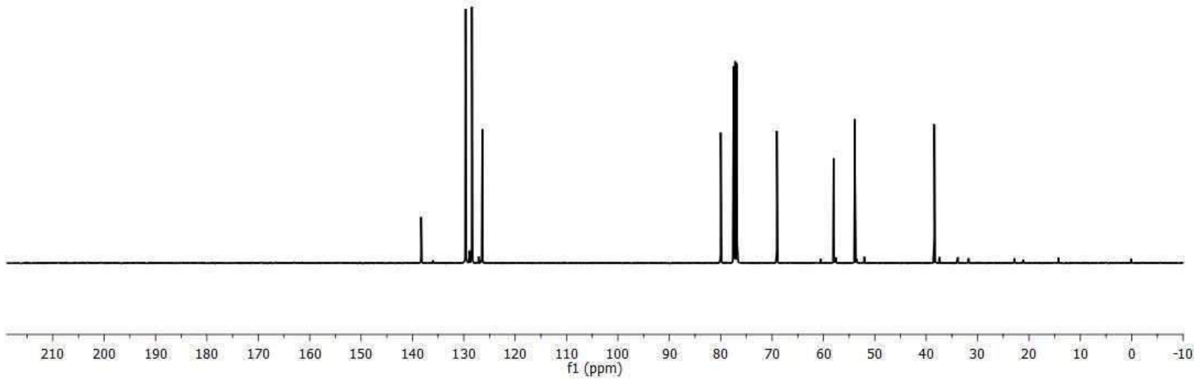

^{1}H NMR, 500 MHz, $CDCl_3$ **1l**

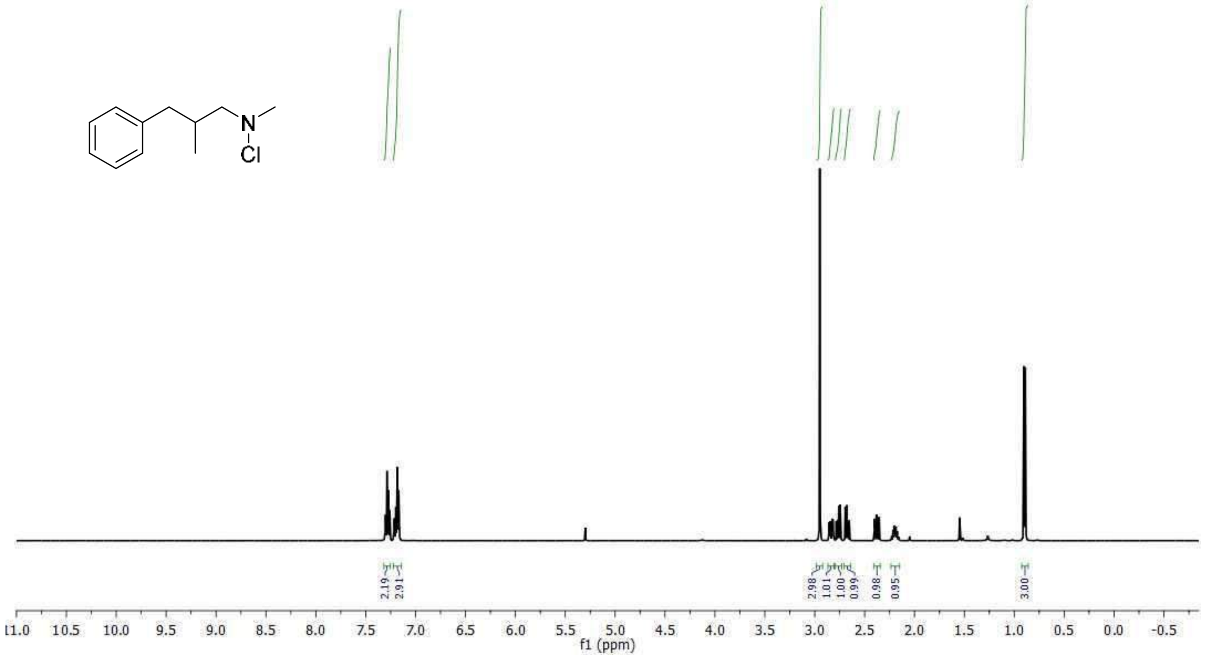

^{13}C NMR, 125 MHz, $CDCl_3$ **1l**

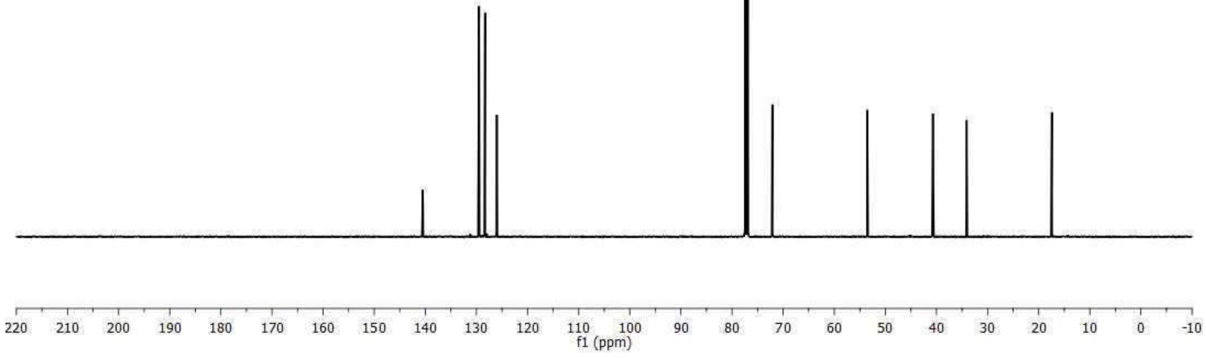

^{1}H NMR, 300 MHz, $CDCl_3$ **1m**

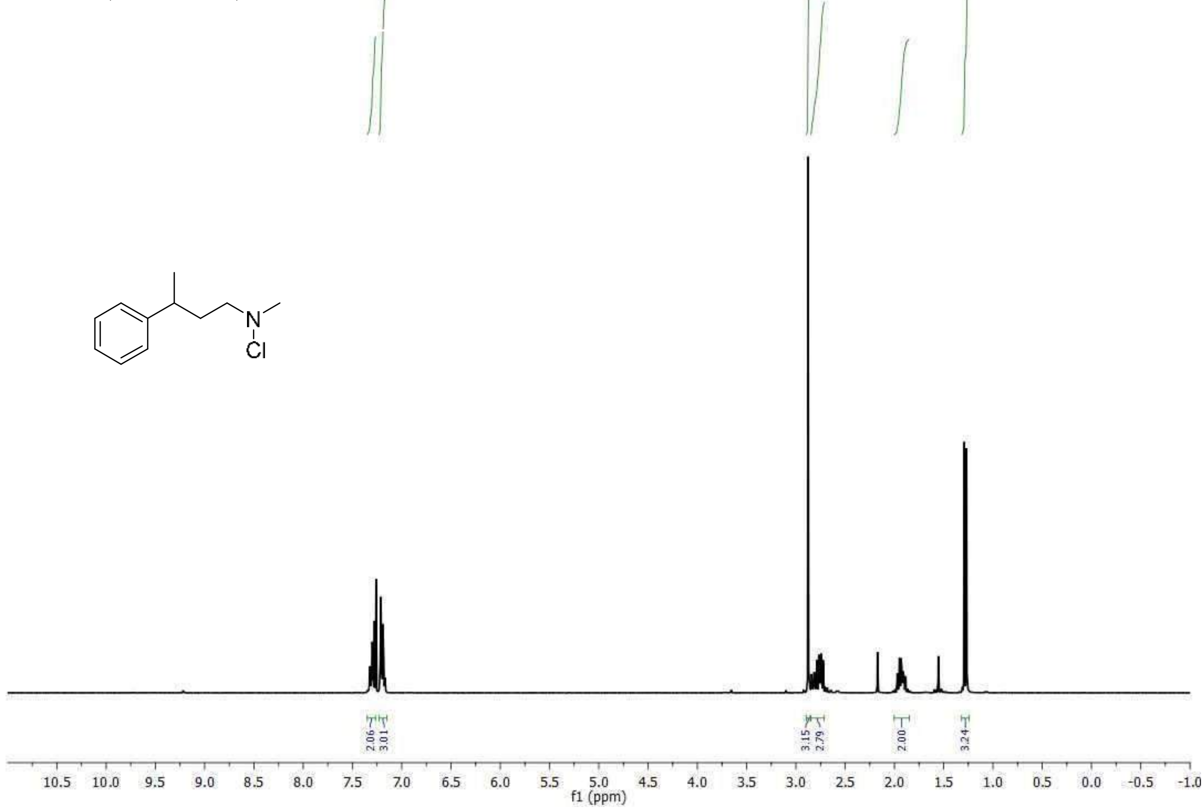

^{13}C NMR, 75 MHz, $CDCl_3$ **1m**

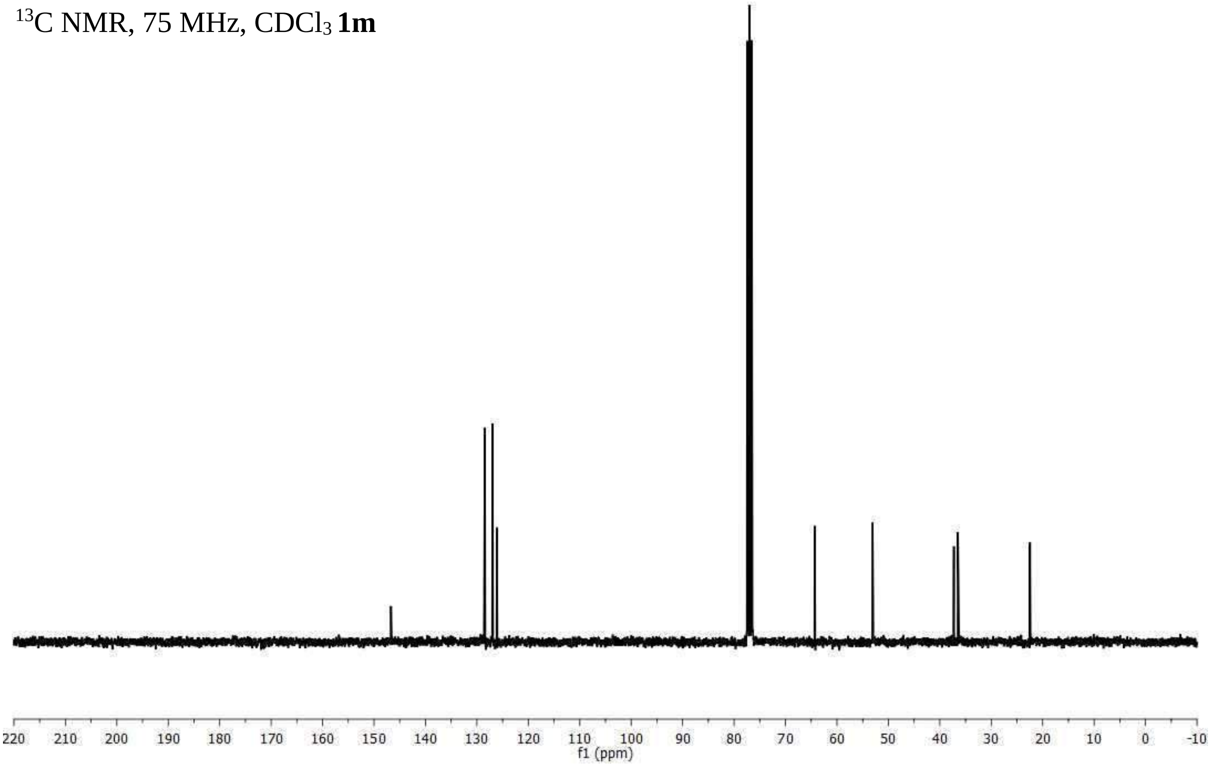

^{1}H NMR, 300 MHz, $CDCl_3$ **1n**

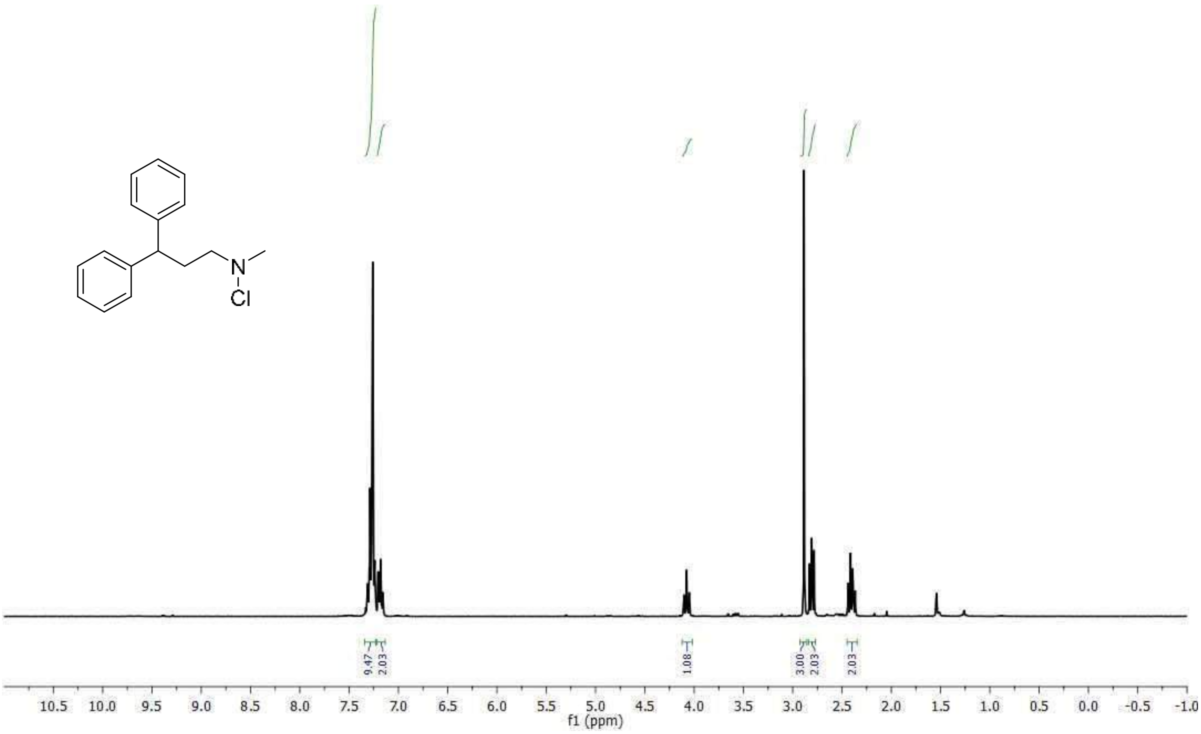

^{13}C NMR, 75 MHz, $CDCl_3$ **1n**

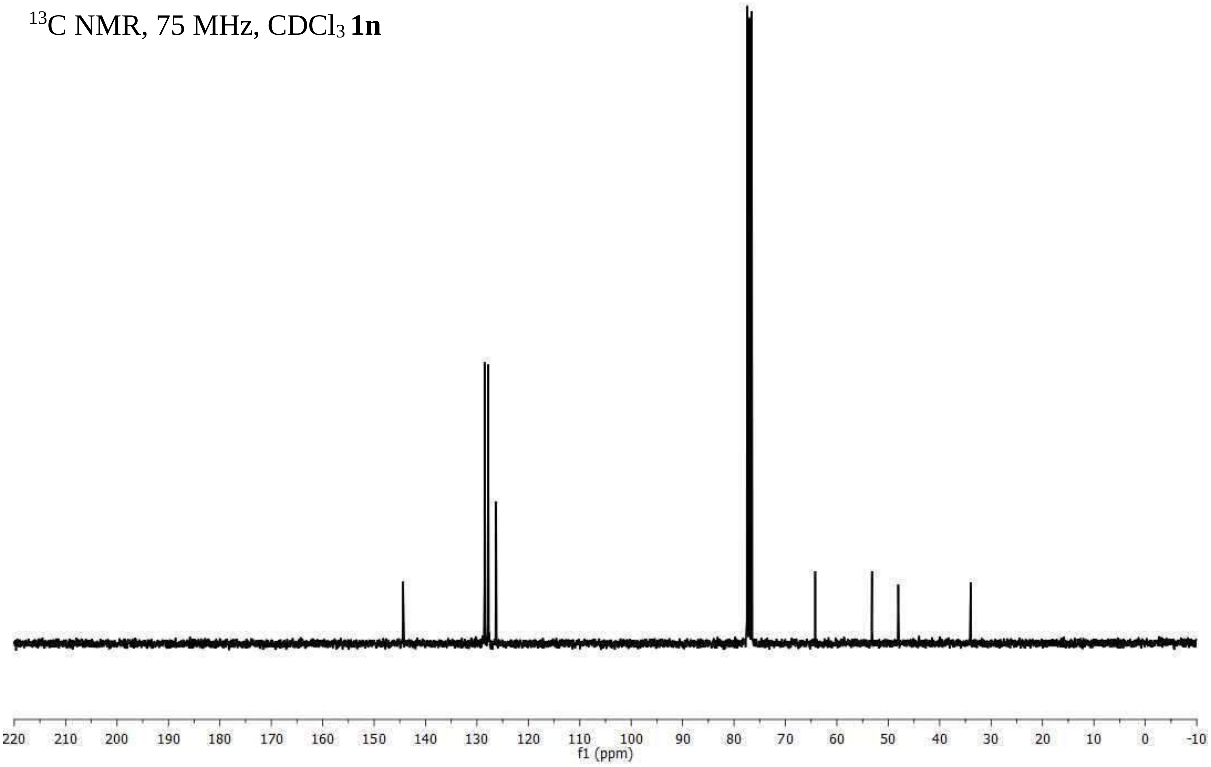

^{1}H NMR, 400 MHz, $CDCl_3$ **1o**

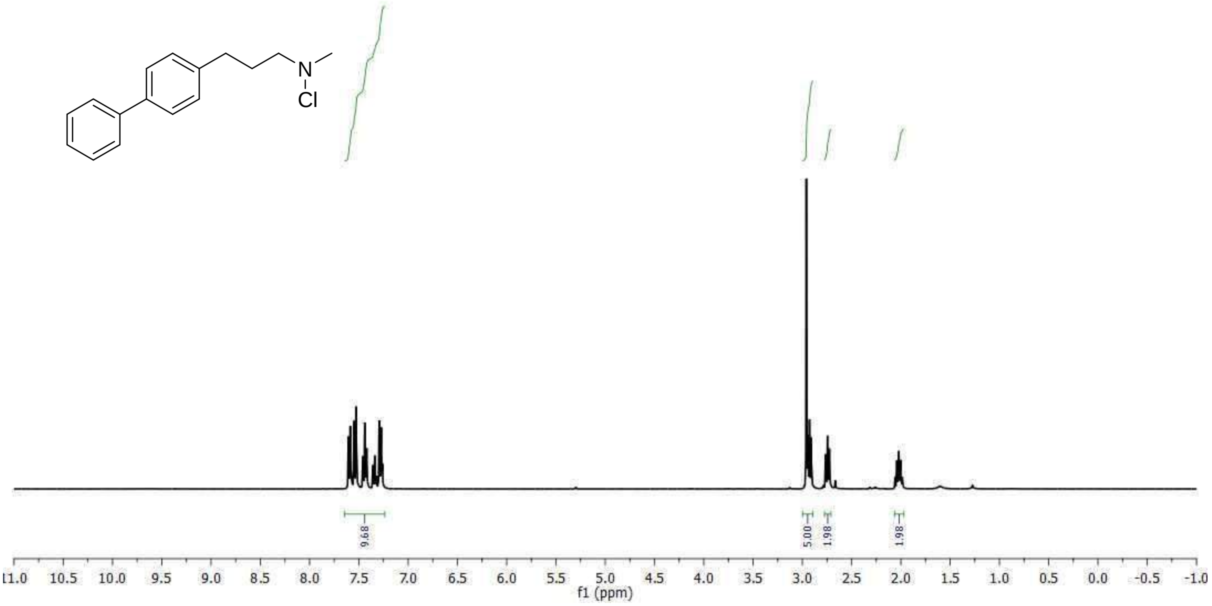

^{13}C NMR, 100 MHz, $CDCl_3$ **1o**

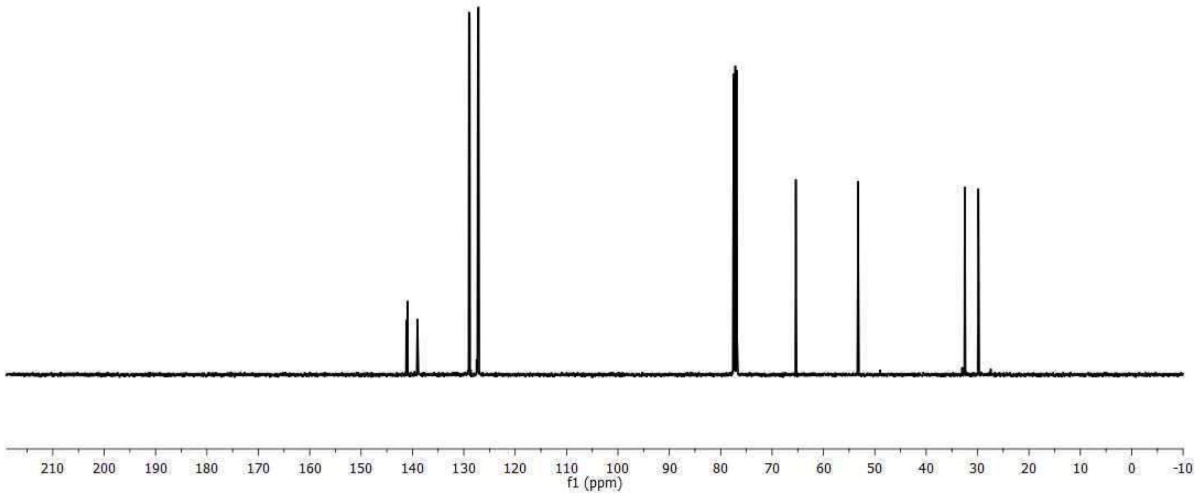

^{1}H NMR, 500 MHz, $CDCl_3$ **1p**

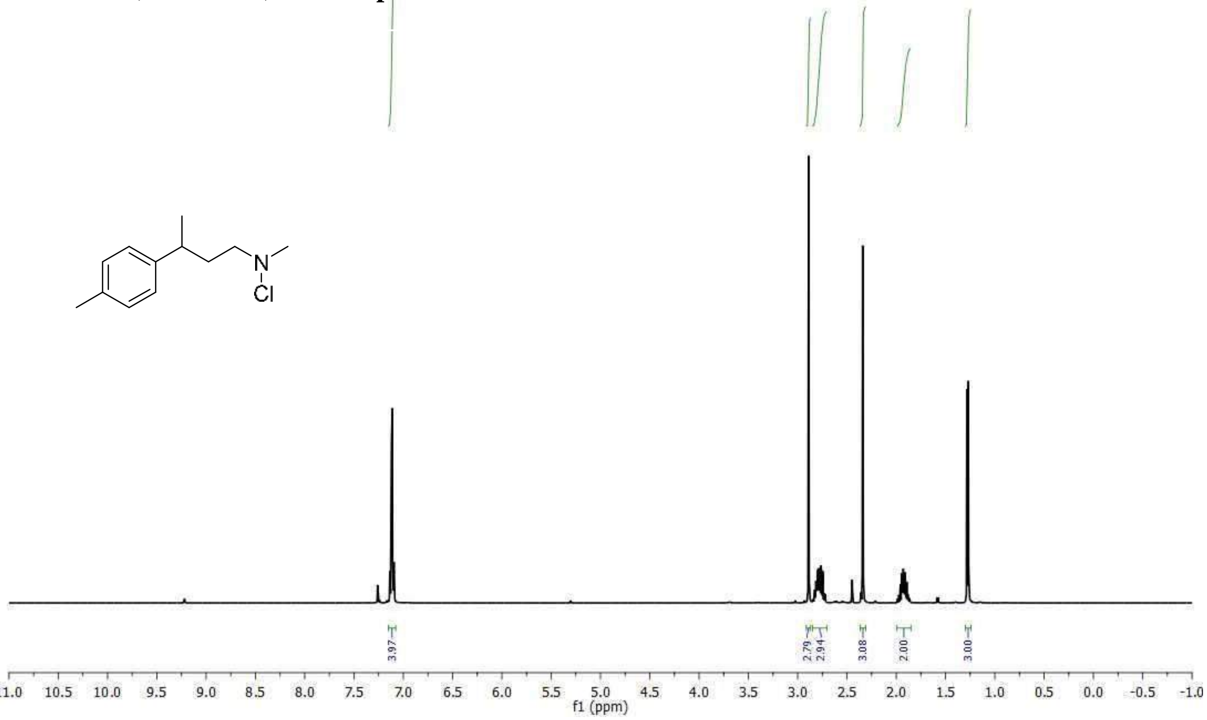

^{13}C NMR, 125 MHz, $CDCl_3$ **1p**

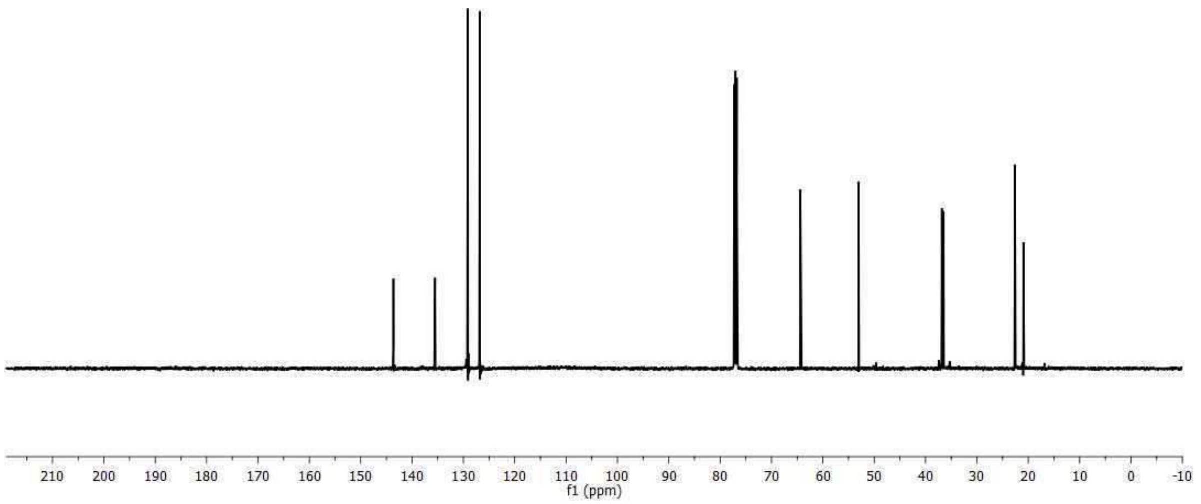

^{1}H NMR, 400 MHz, $CDCl_3$ **1q**

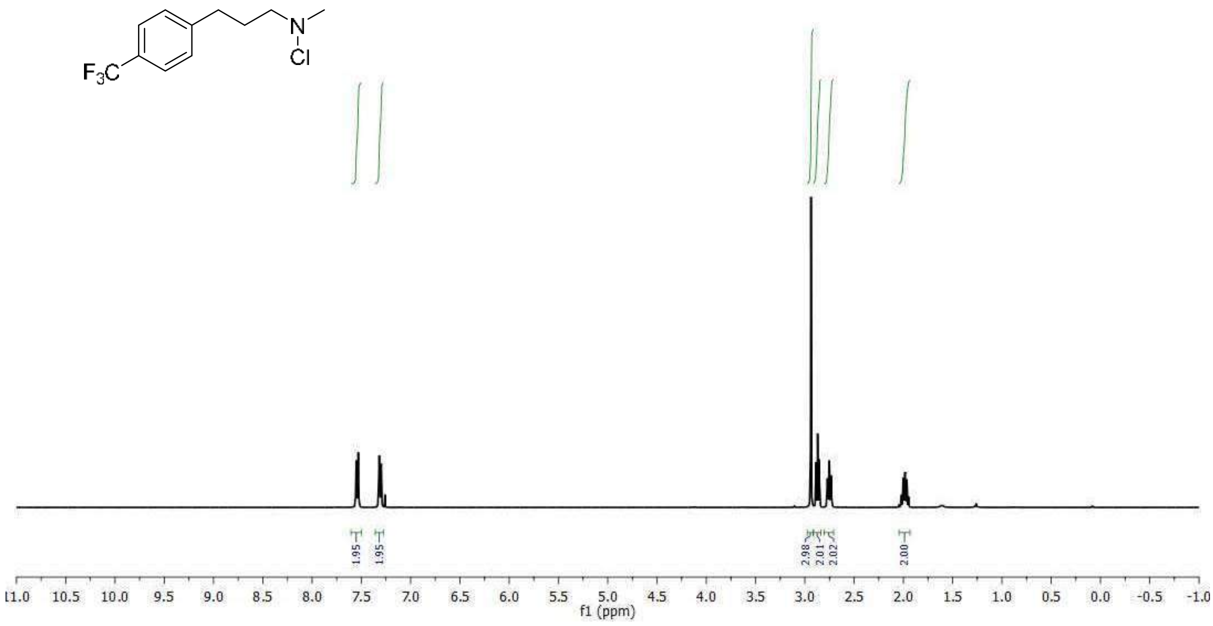

^{13}C NMR, 100 MHz, $CDCl_3$ **1q**

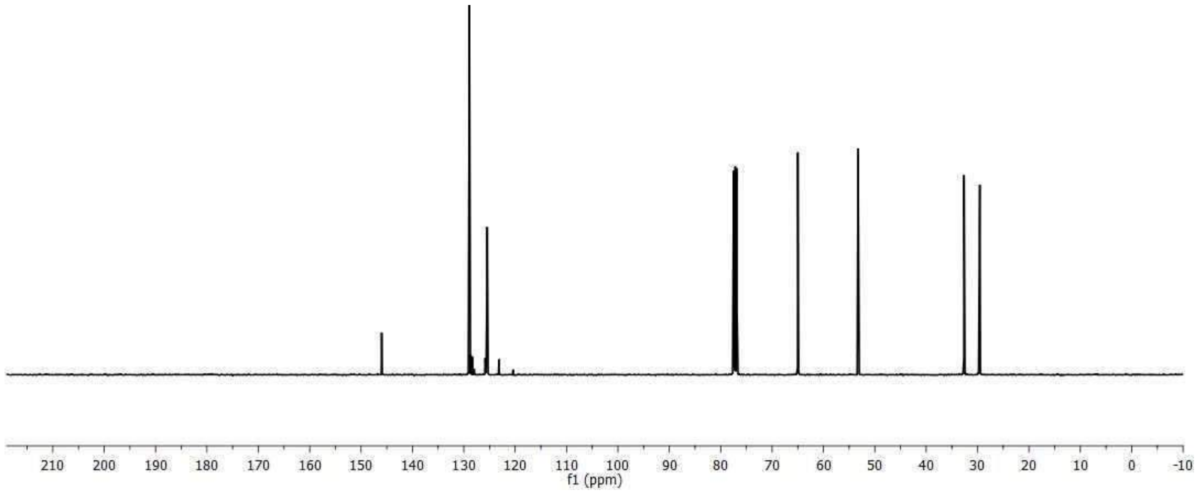

^{1}H NMR, 300 MHz, $CDCl_3$ **1r**

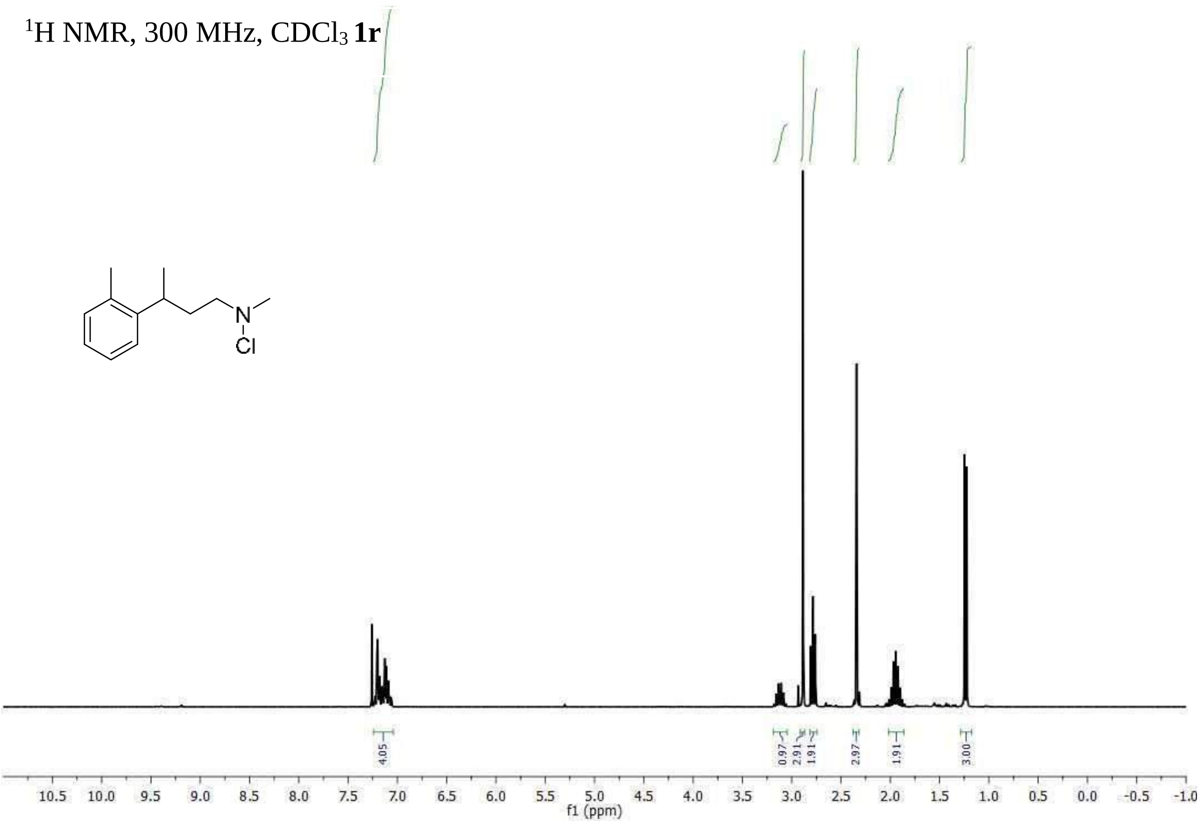

^{13}C NMR, 75 MHz, $CDCl_3$ **1r**

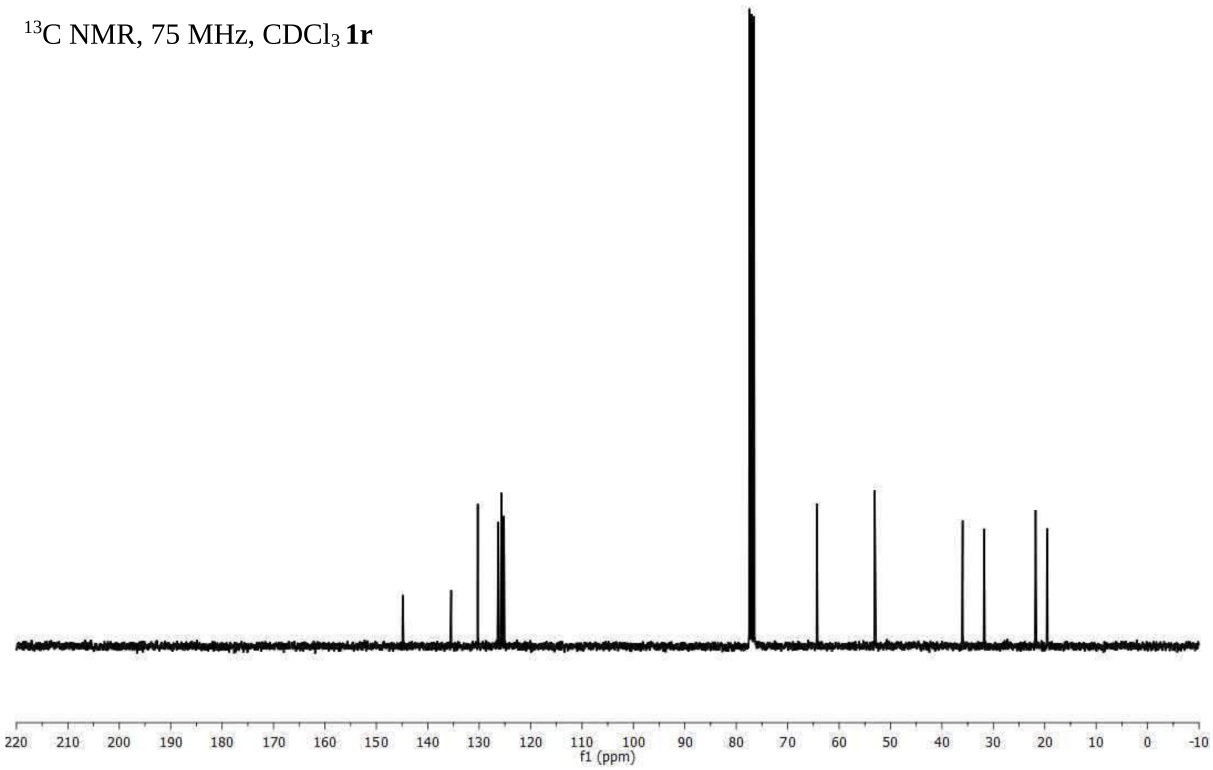

^{1}H NMR, 400 MHz, $CDCl_3$ **1s**

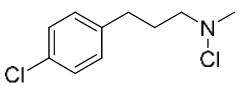

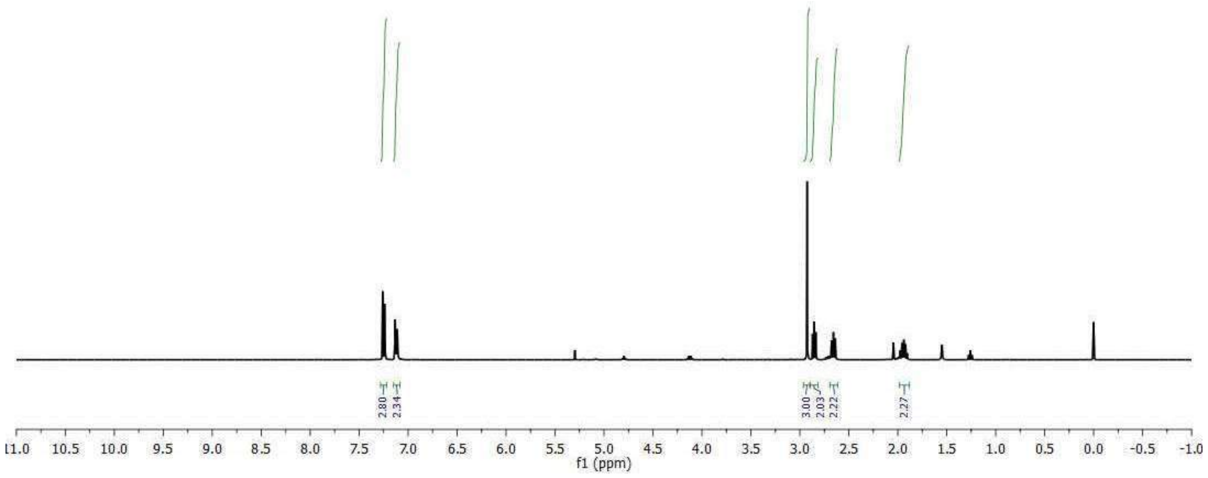

^{13}C NMR, 100 MHz, $CDCl_3$ **1s**

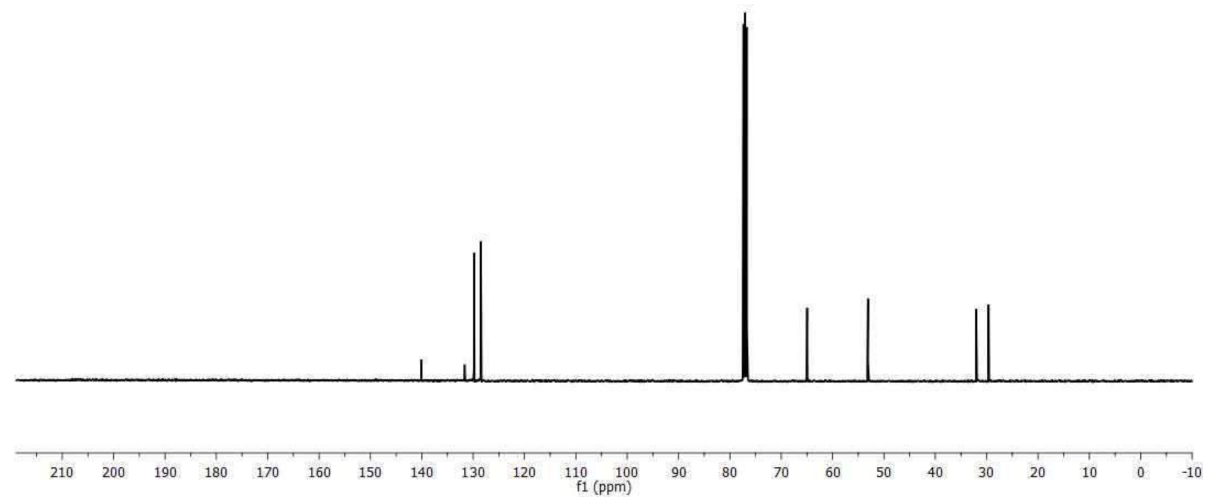

^{1}H NMR, 500 MHz, $CDCl_3$ **1t**

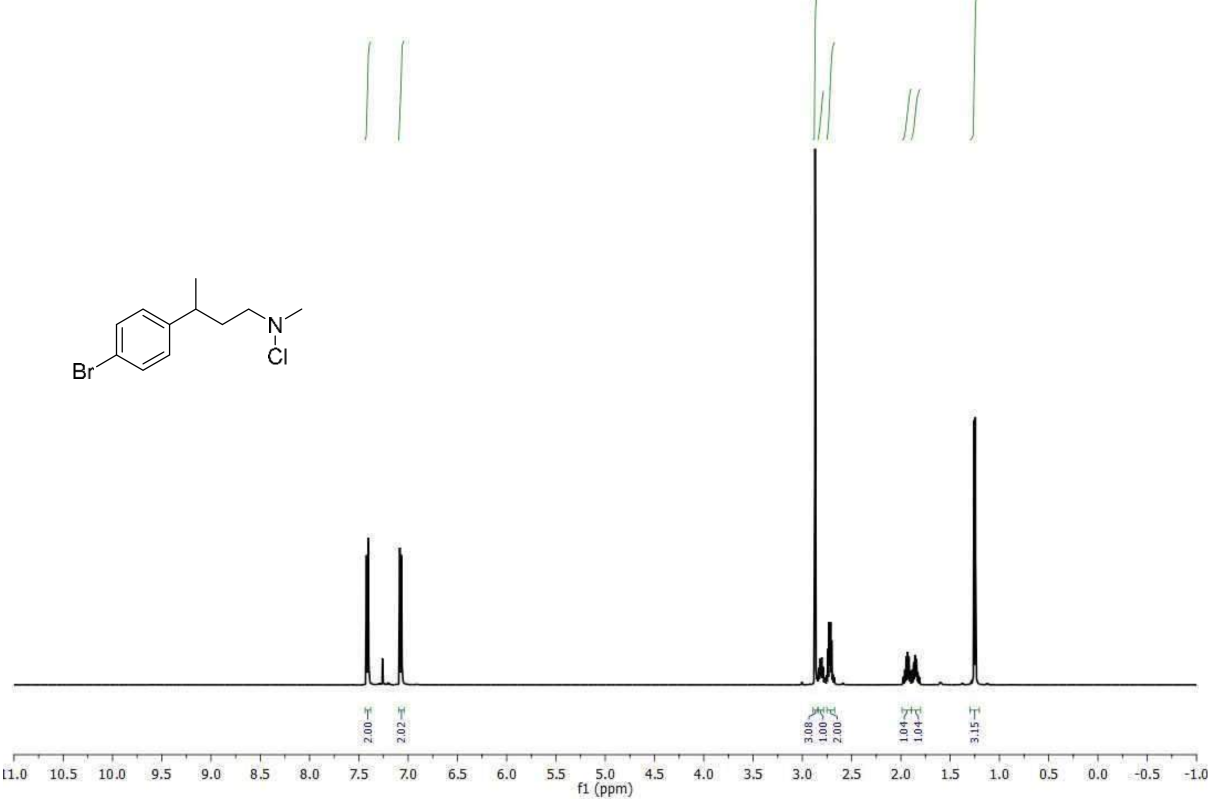

^{13}C NMR, 125 MHz, $CDCl_3$ **1t**

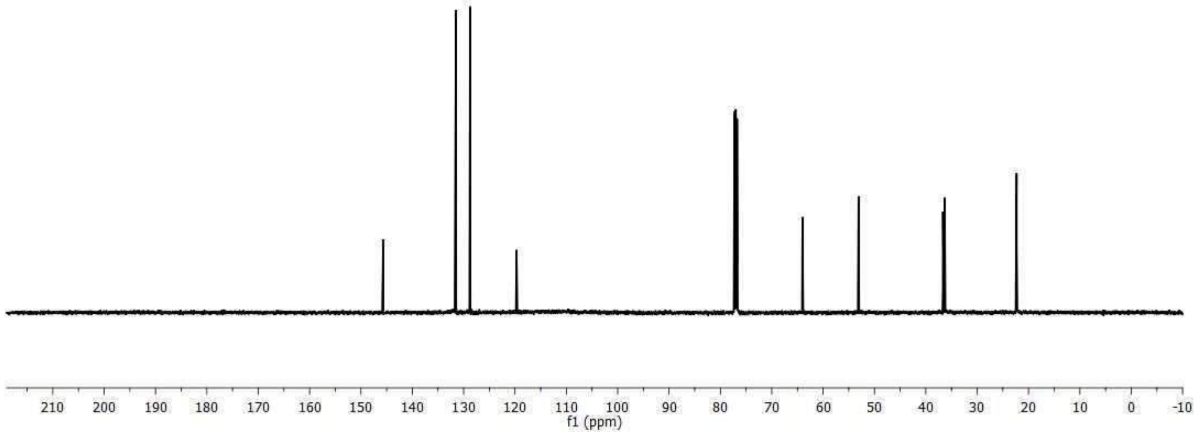

^{1}H NMR, 400 MHz, $CDCl_3$ **1u**

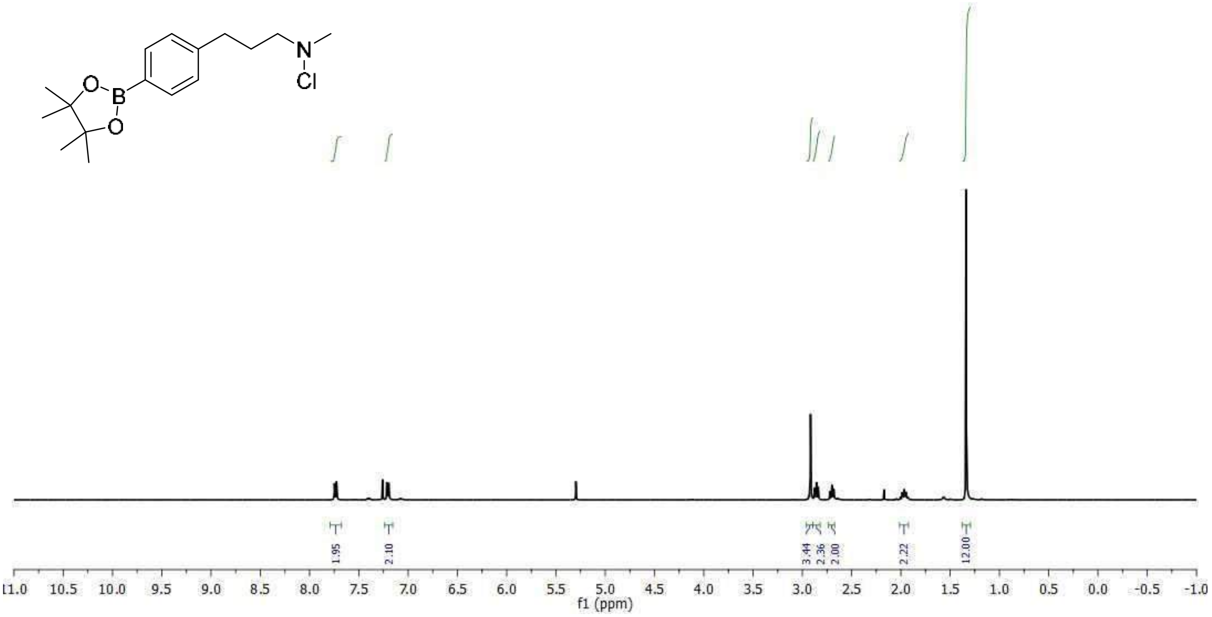

^{13}C NMR, 100 MHz, $CDCl_3$ **1u**

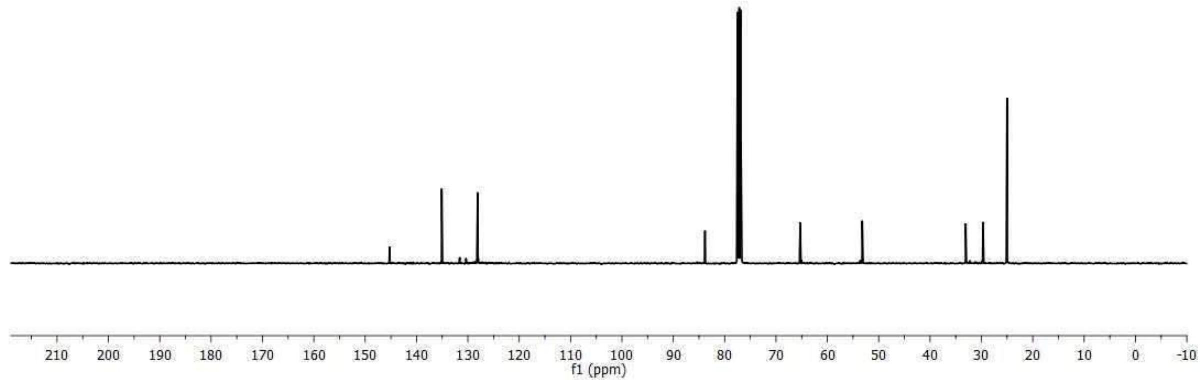

^{1}H NMR, 400 MHz, $CDCl_3$ **1v**

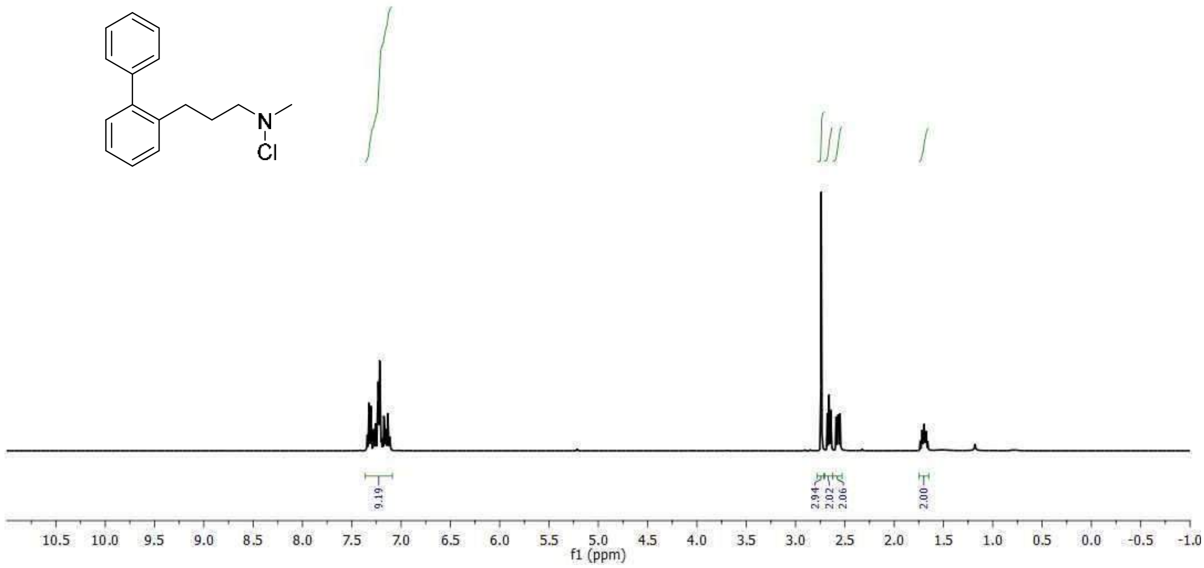

^{13}C NMR, 100 MHz, $CDCl_3$ **1v**

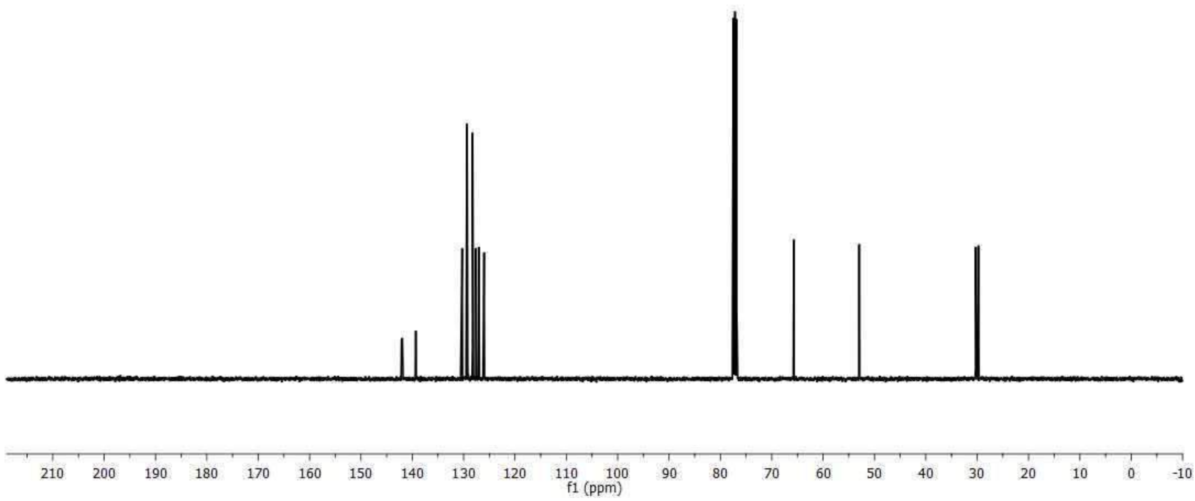

^{1}H NMR, 500 MHz, $CDCl_3$ **1w**

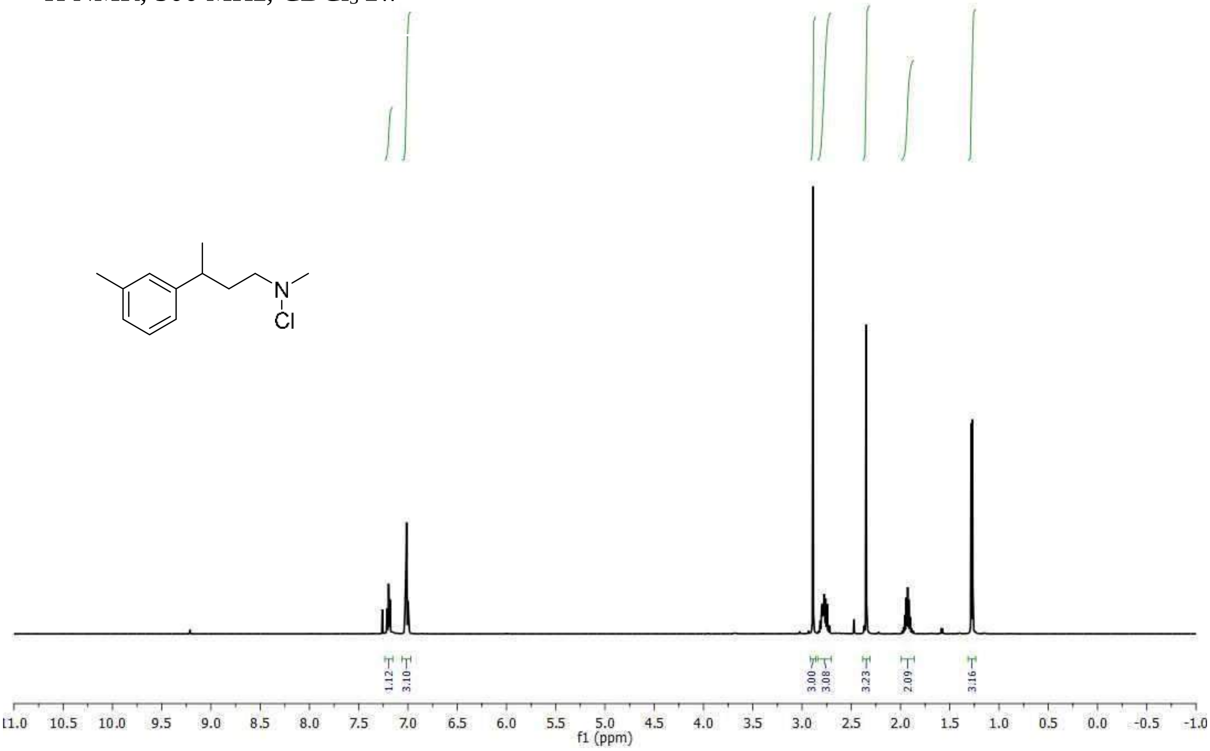

^{13}C NMR, 125 MHz, $CDCl_3$ **1w**

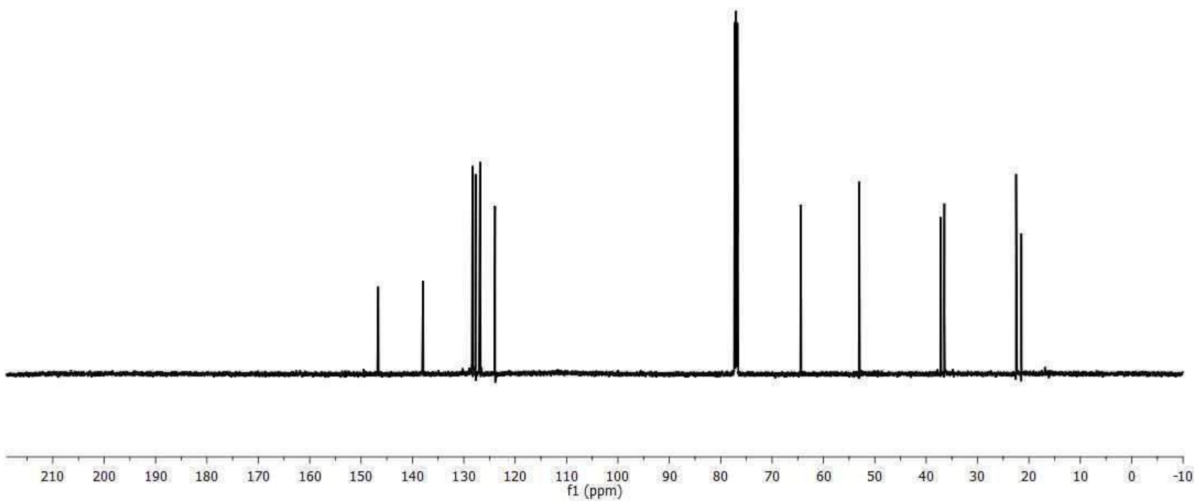

^{1}H NMR, 400 MHz, $CDCl_3$ **1x**

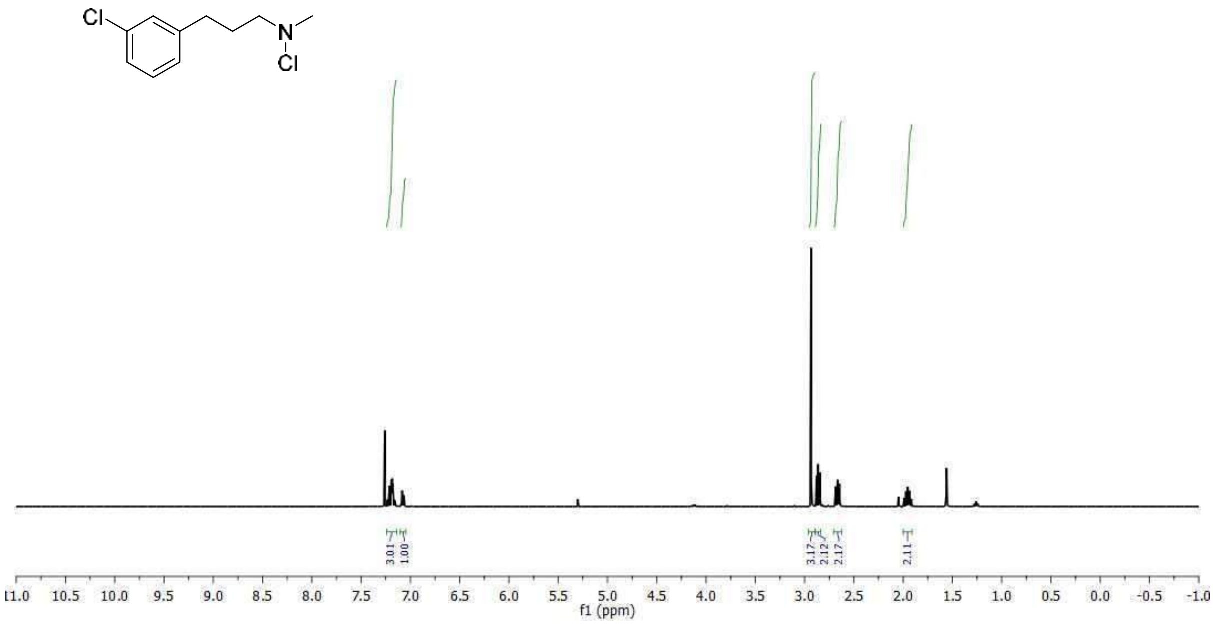

^{13}C NMR, 100 MHz, $CDCl_3$ **1x**

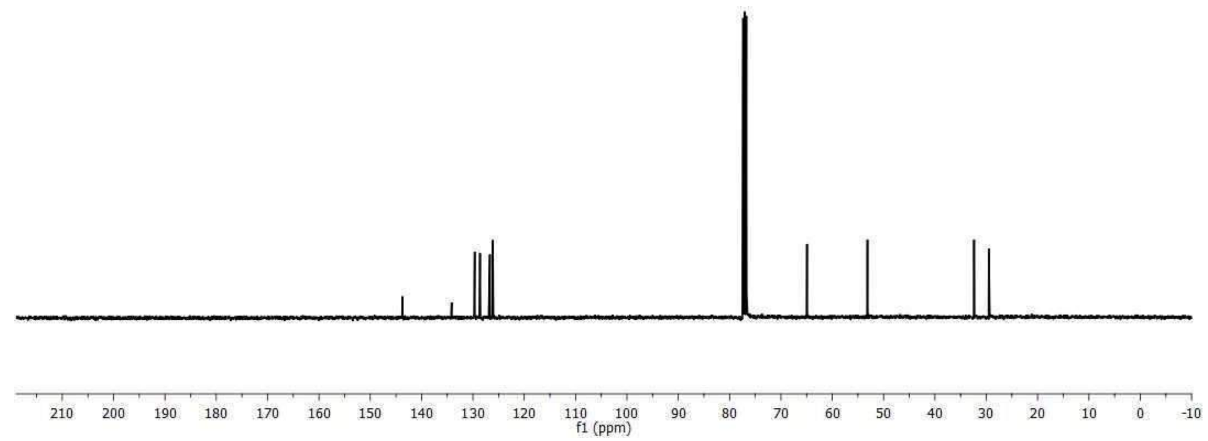

^{1}H NMR, 400 MHz, $CDCl_3$ **6**

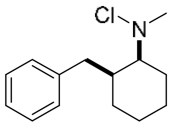

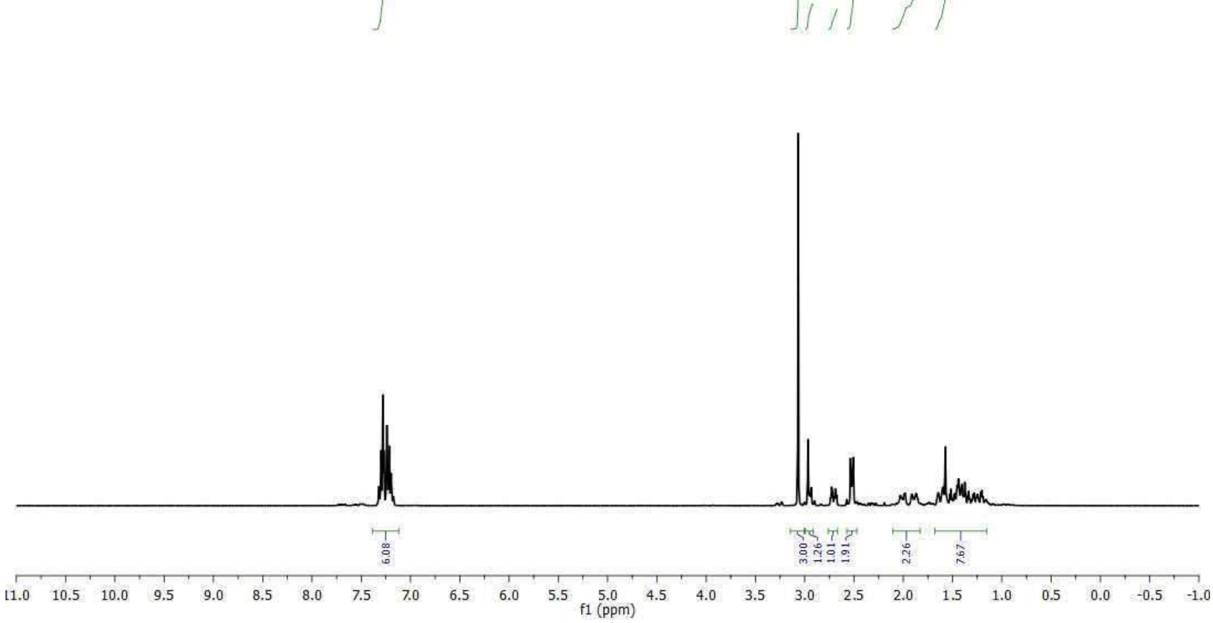

^{13}C NMR, 100 MHz, $CDCl_3$ **6**

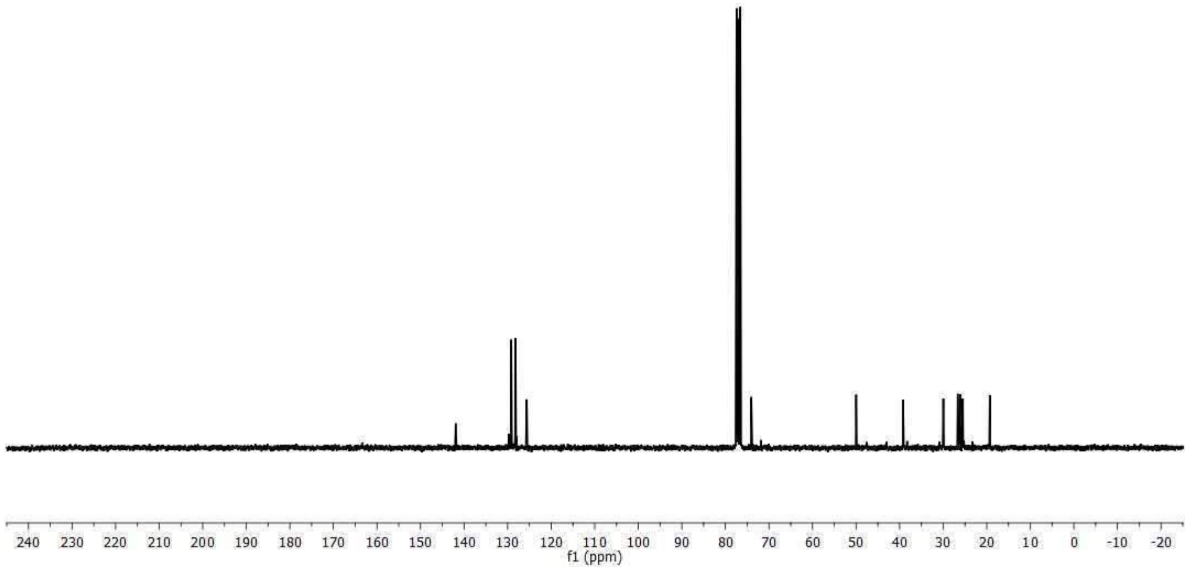

^{1}H NMR, 500 MHz, $CDCl_3$ **8**

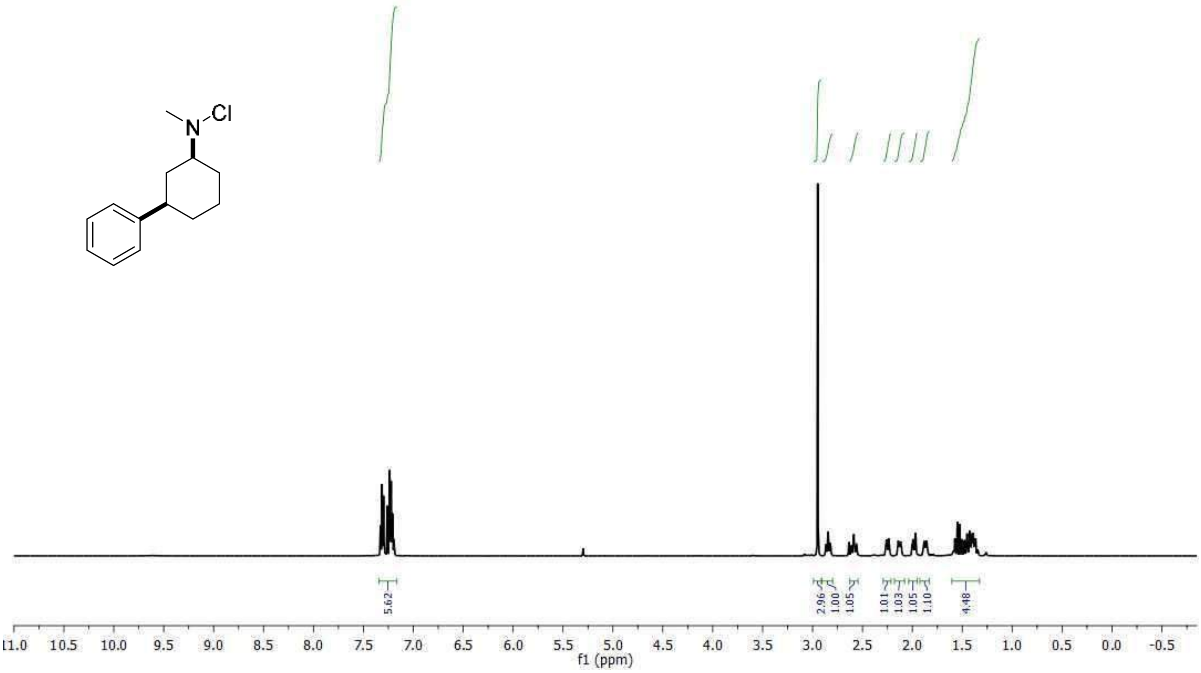

^{13}C NMR, 125 MHz, $CDCl_3$ **8**

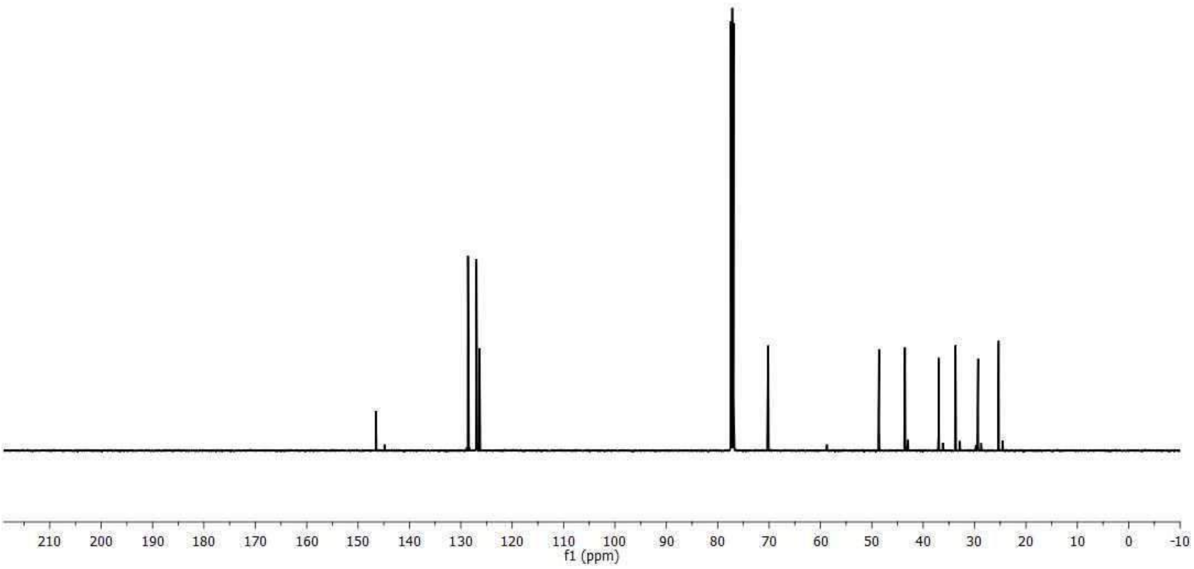

^{1}H NMR, 500 MHz, $CDCl_3$ **11**

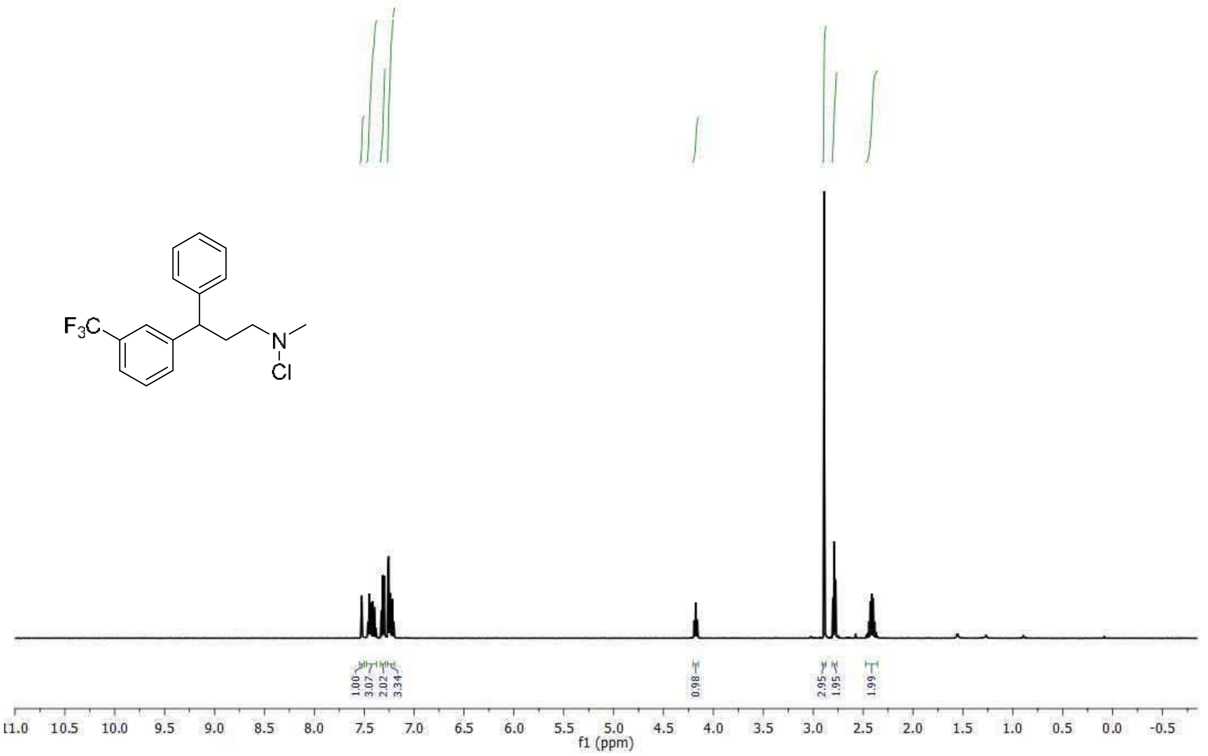

^{13}C NMR, 125 MHz, $CDCl_3$ **11**

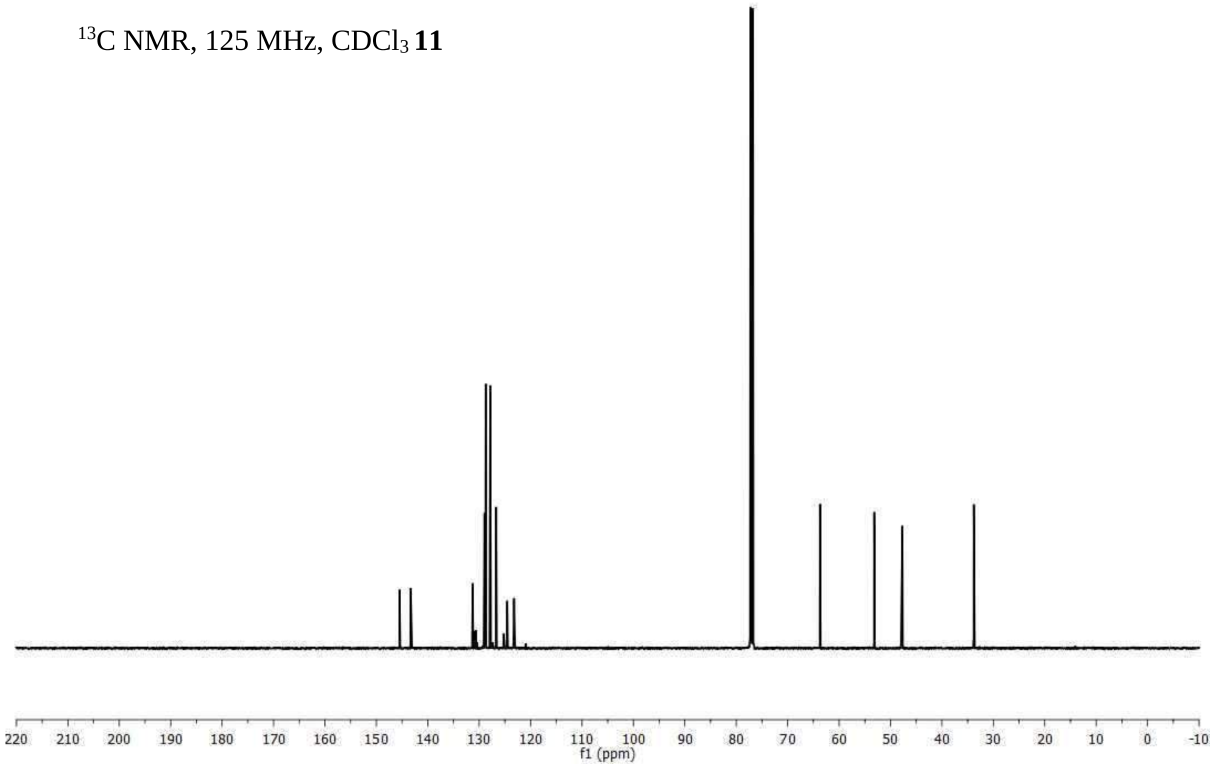

^{1}H NMR, 500 MHz, $CDCl_3$ **10**

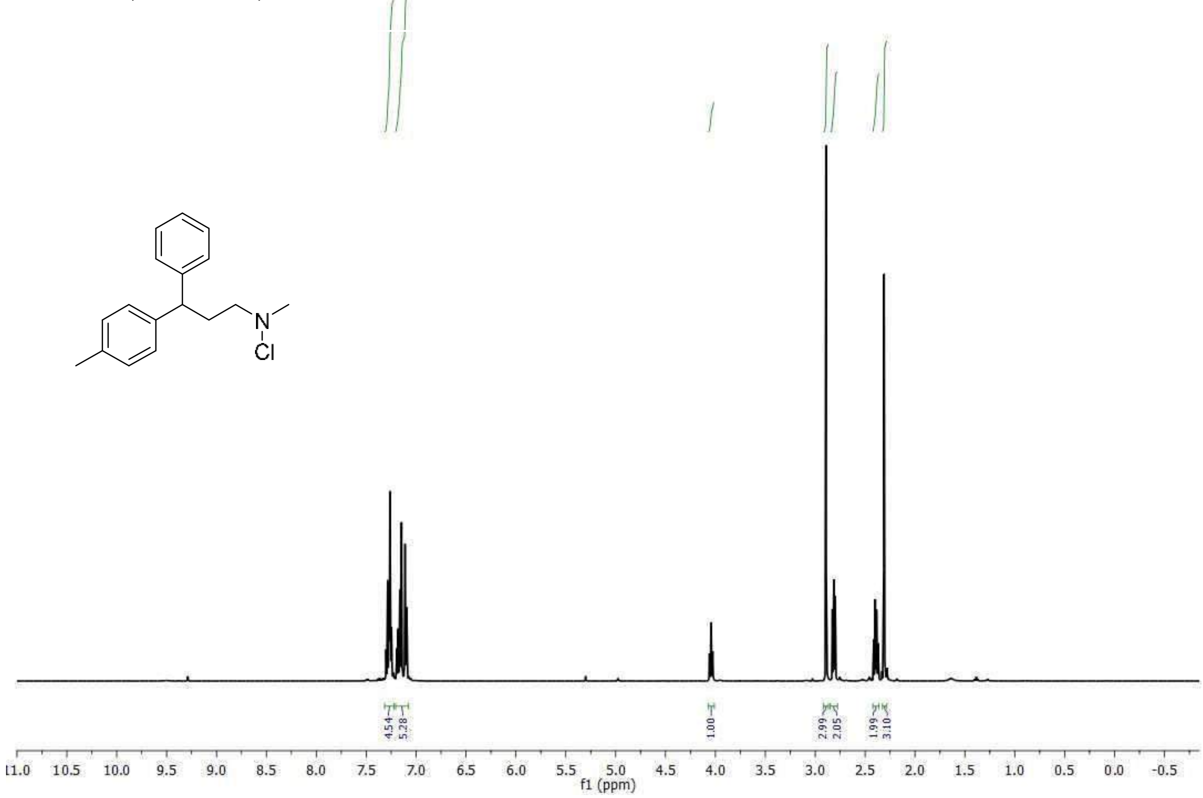

^{13}C NMR, 125 MHz, $CDCl_3$ **10**

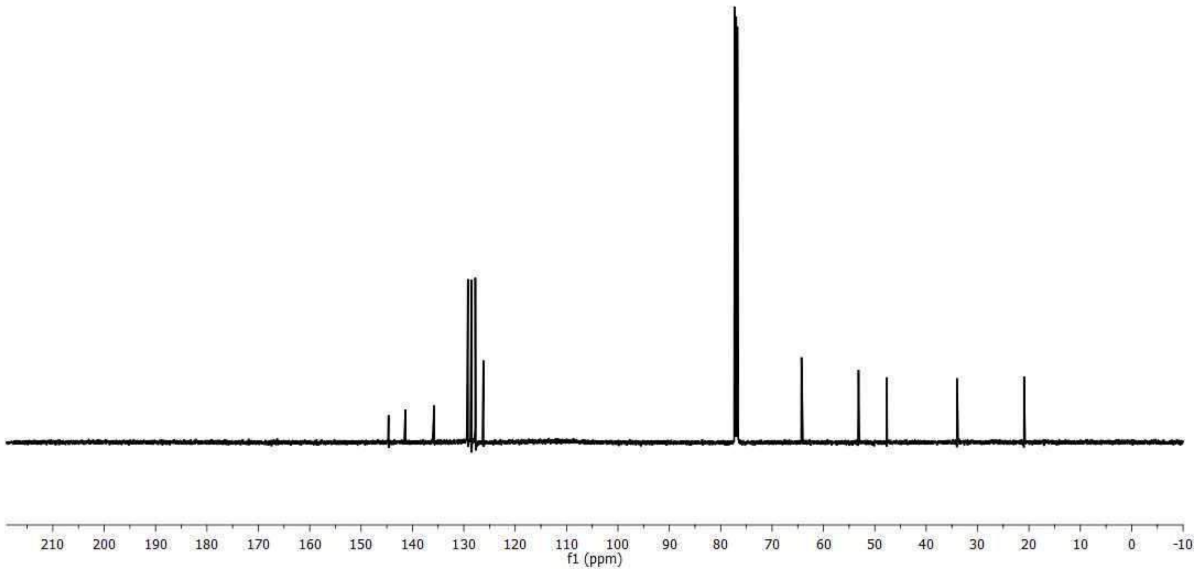

^{1}H NMR, 400 MHz, $CDCl_3$ **14**

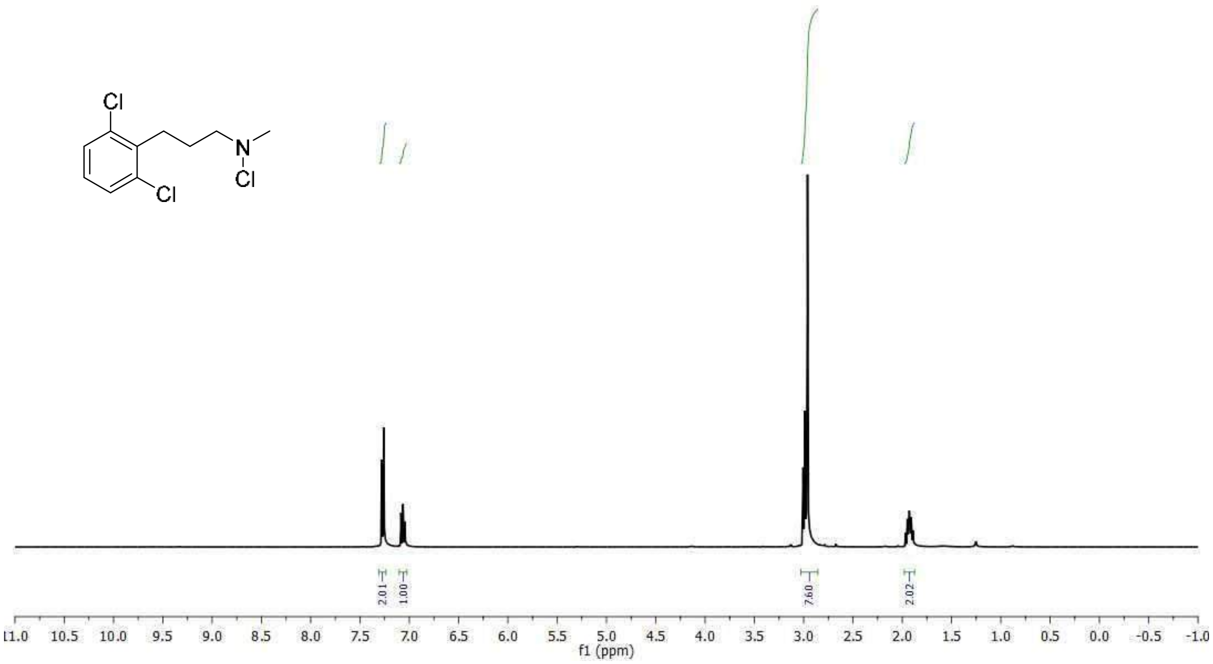

^{13}C NMR, 100 MHz, $CDCl_3$ **14**

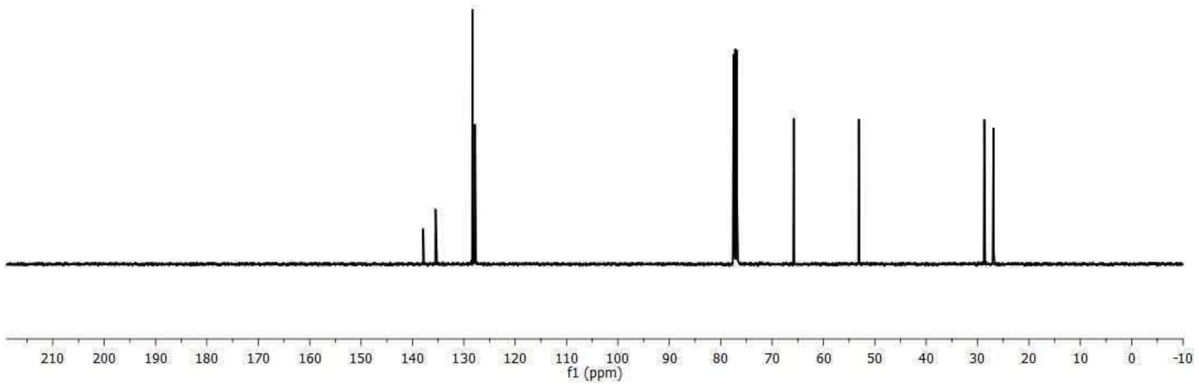

^{1}H NMR, 400 MHz, $CDCl_3$ **16**

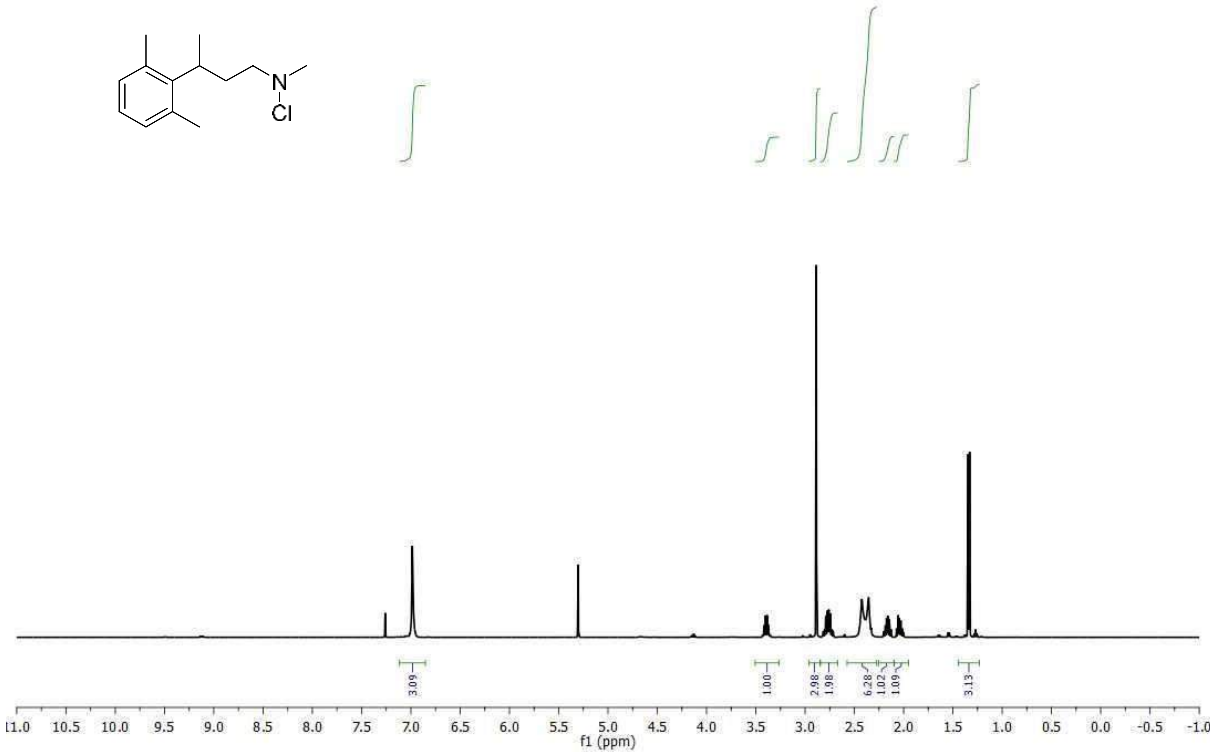

^{13}C NMR, 100 MHz, $CDCl_3$ **16**

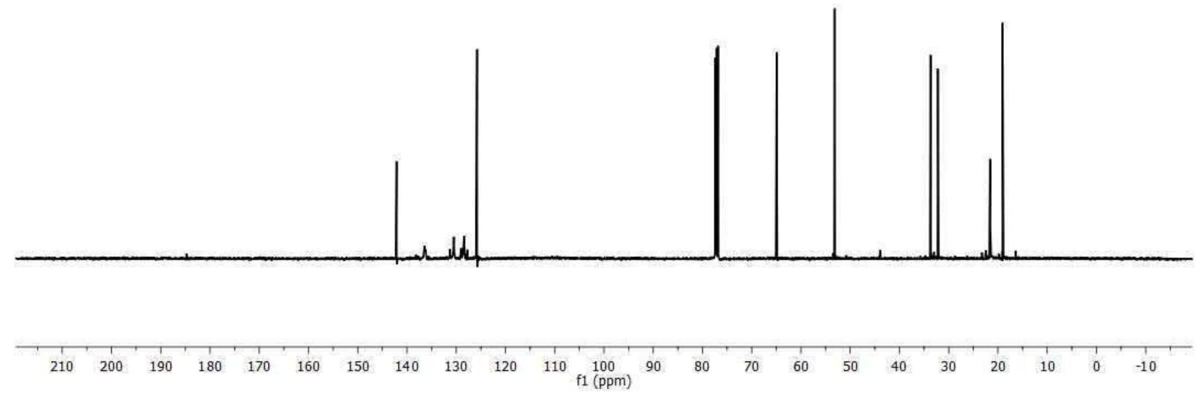

1.8 ^{1}H and ^{13}C NMR spectra for tetrahydroquinolines

^{1}H NMR, 300 MHz, $CDCl_3$ **2a**

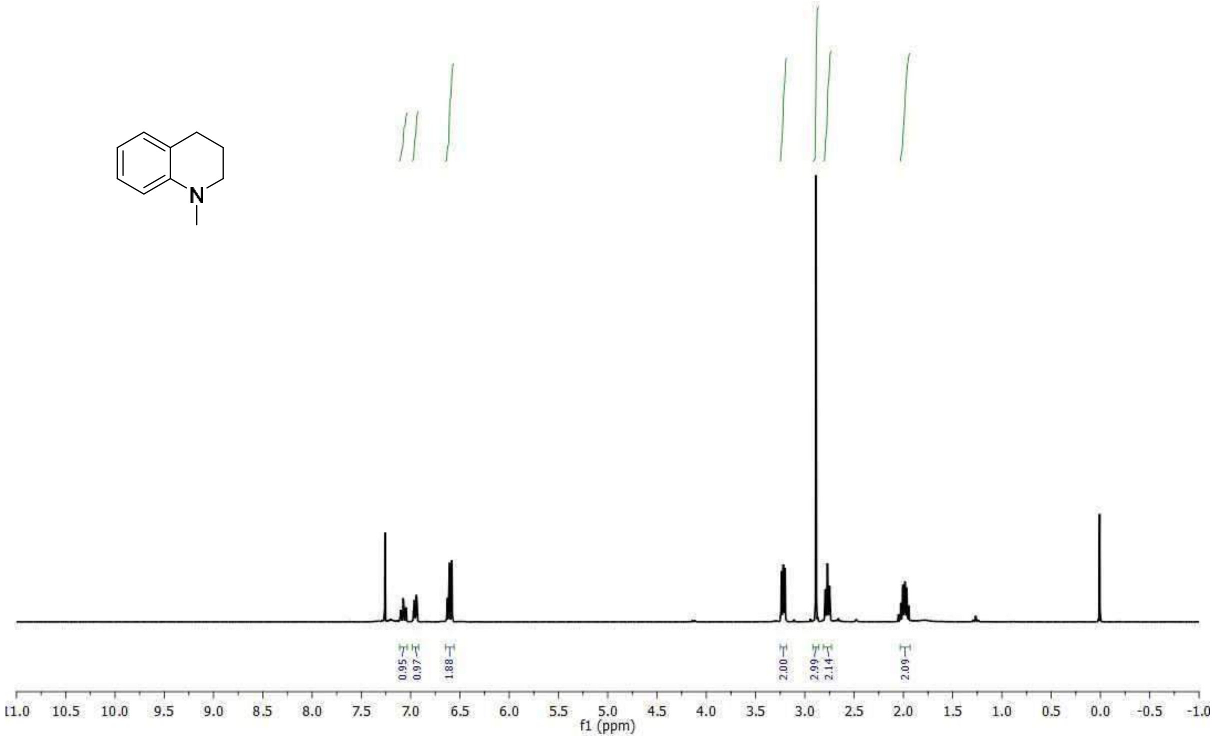

^{13}C NMR, 75 MHz, $CDCl_3$ **2a**

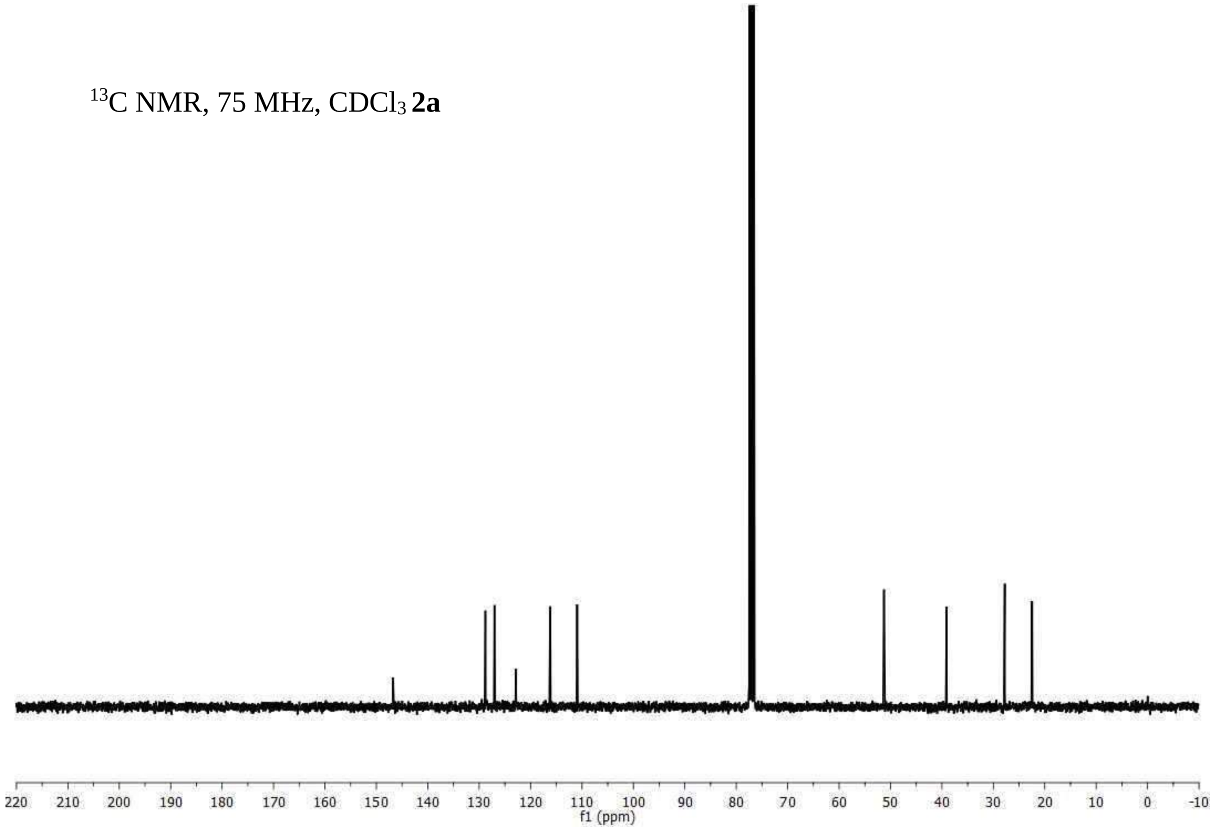

^{1}H NMR, 300 MHz, $CDCl_3$ **2b**

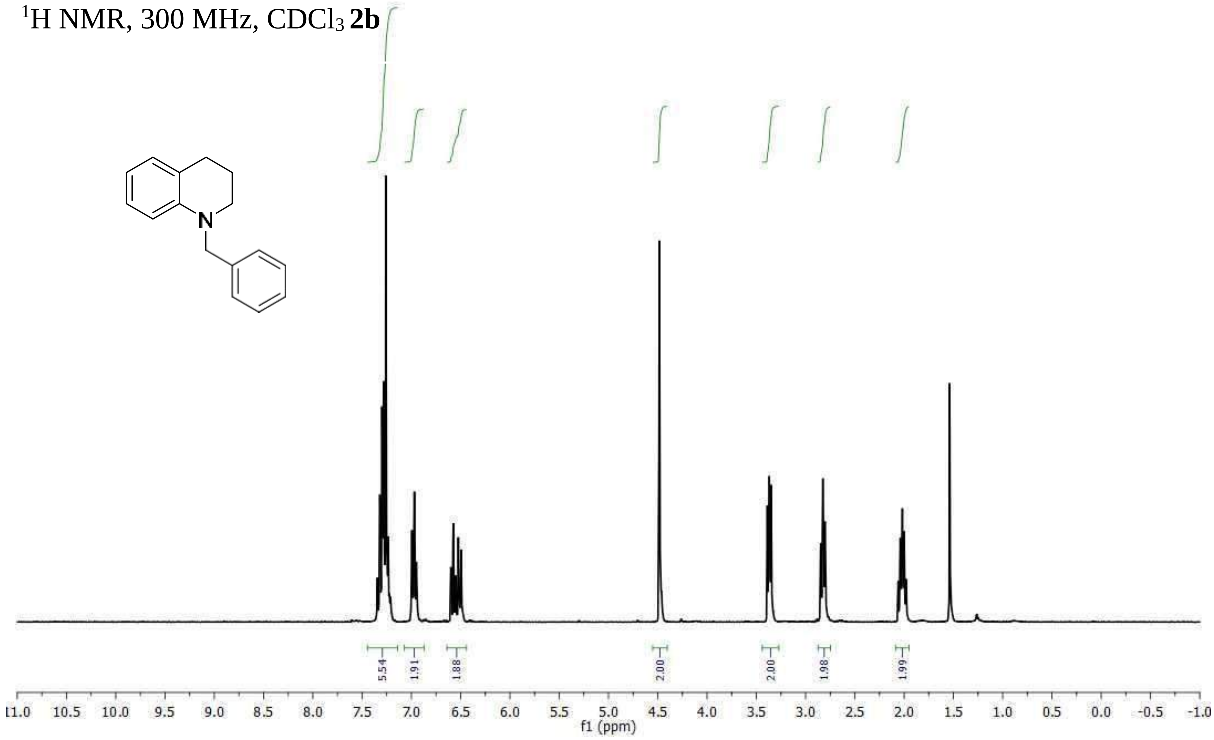

^{13}C NMR, 125 MHz, $CDCl_3$ **2b**

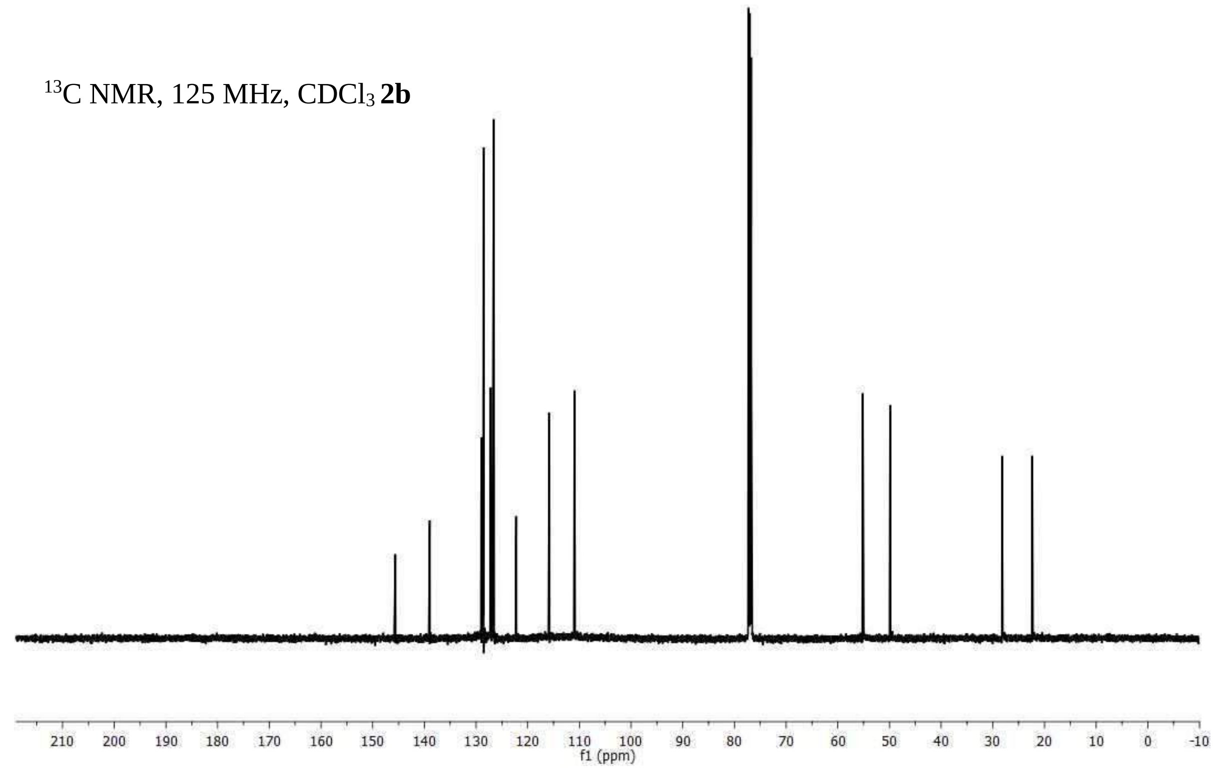

^{1}H NMR, 300 MHz, $CDCl_3$ **2c**

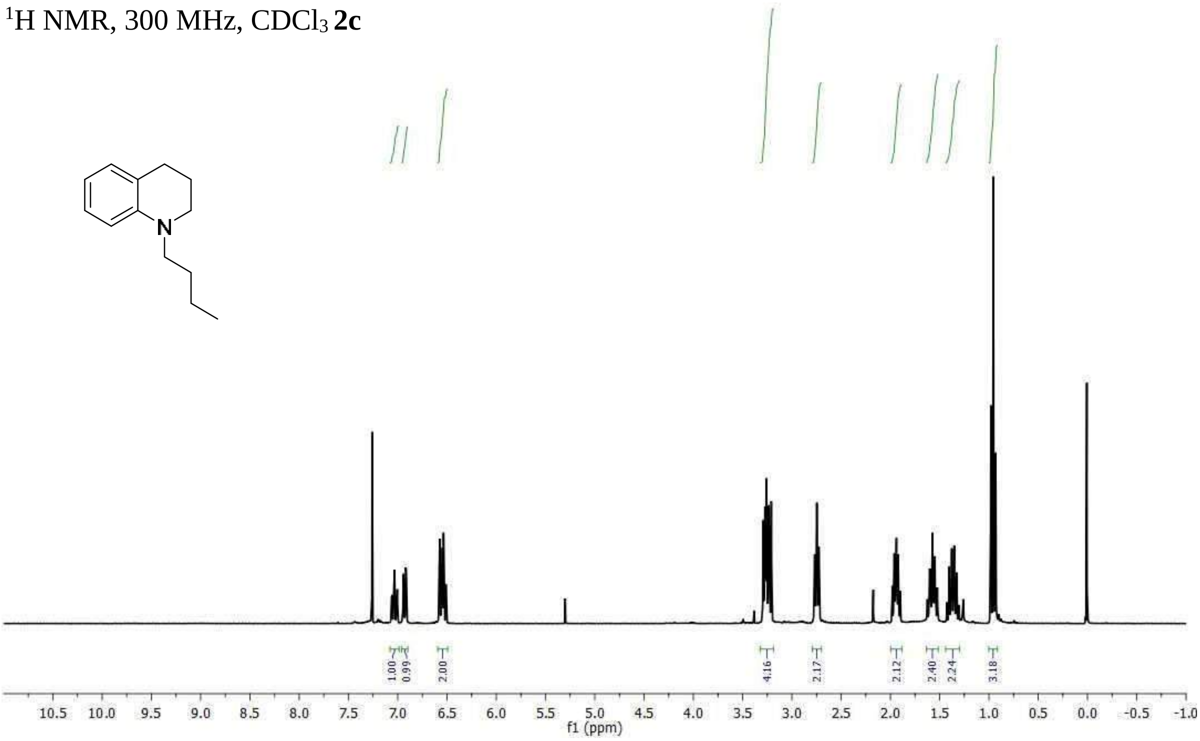

^{13}C NMR, 75 MHz, $CDCl_3$ **2c**

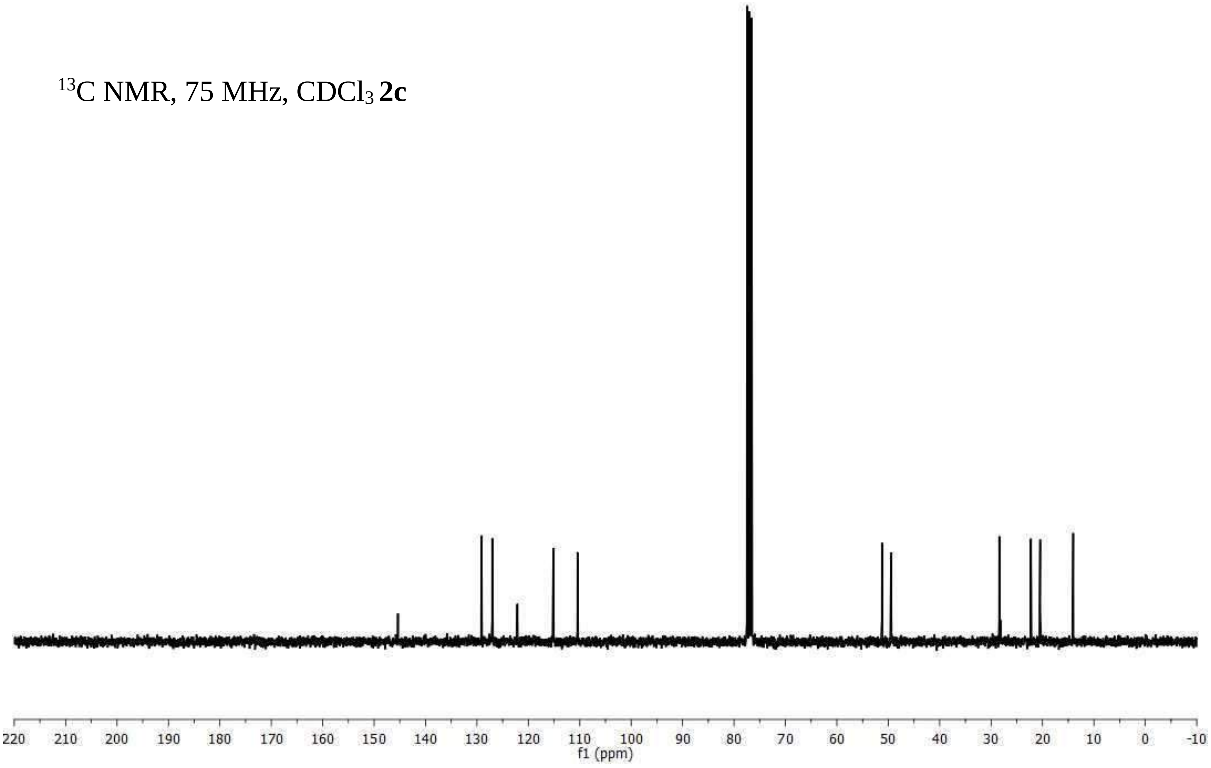

^{1}H NMR, 300 MHz, $CDCl_3$ **2d**

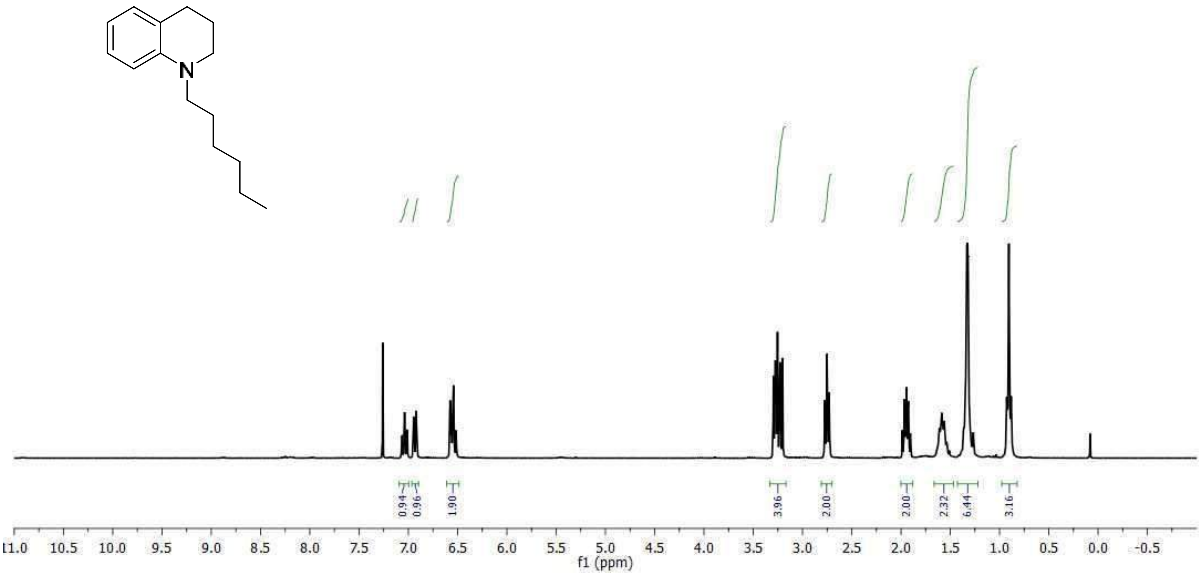

^{13}C NMR, 75 MHz, $CDCl_3$ **2d**

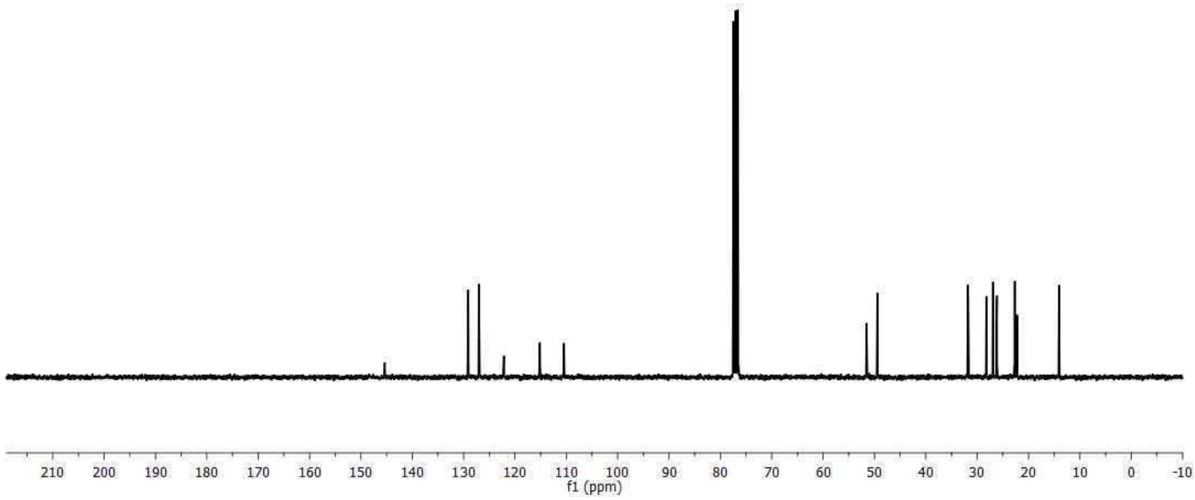

^{1}H NMR, 500 MHz, $CDCl_3$ **2e**

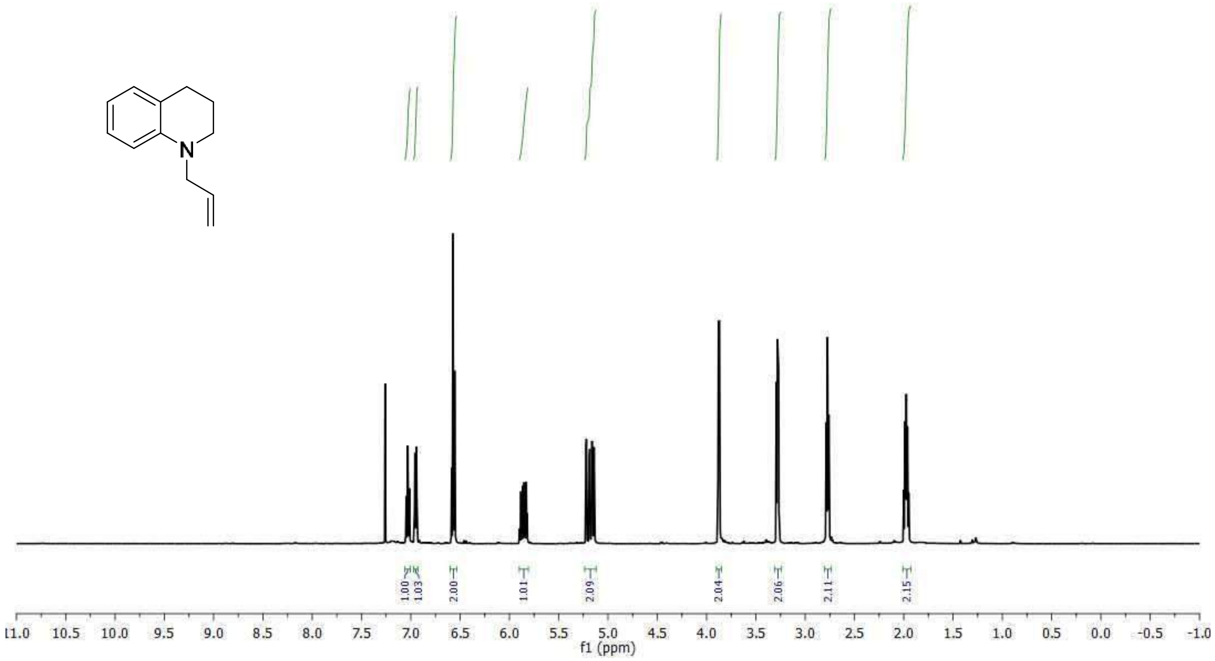

^{13}C NMR, 125 MHz, $CDCl_3$ **2e**

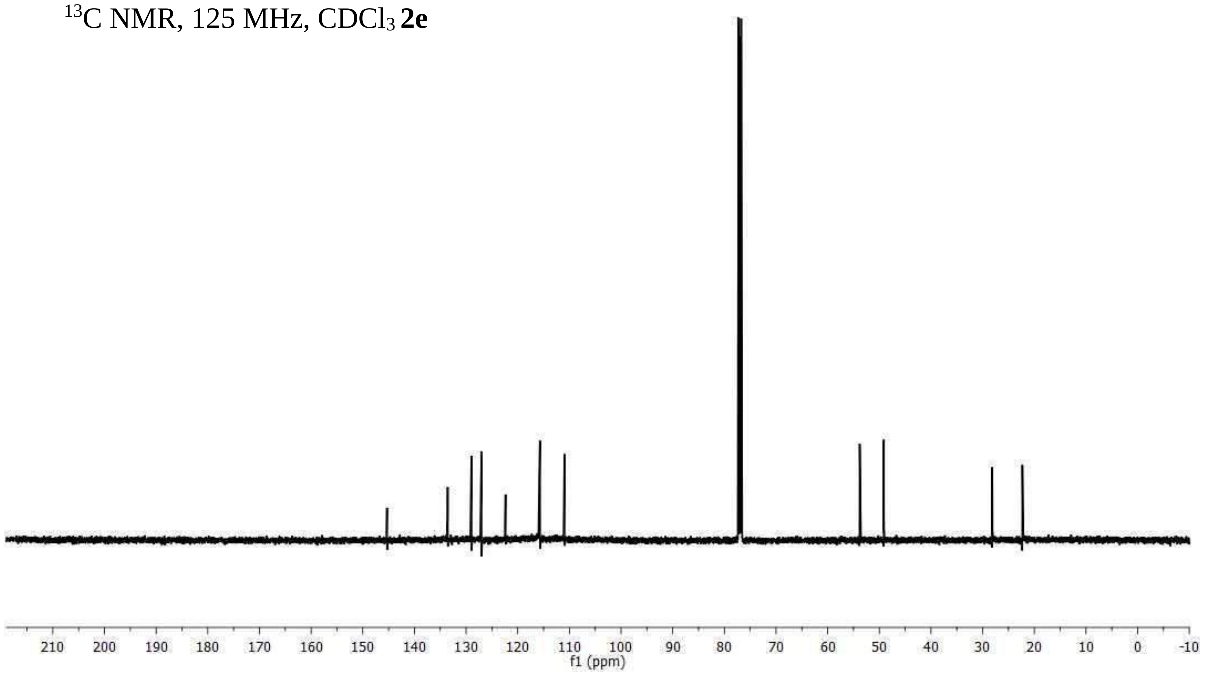

^{1}H NMR, 500 MHz, $CDCl_3$ **2f**

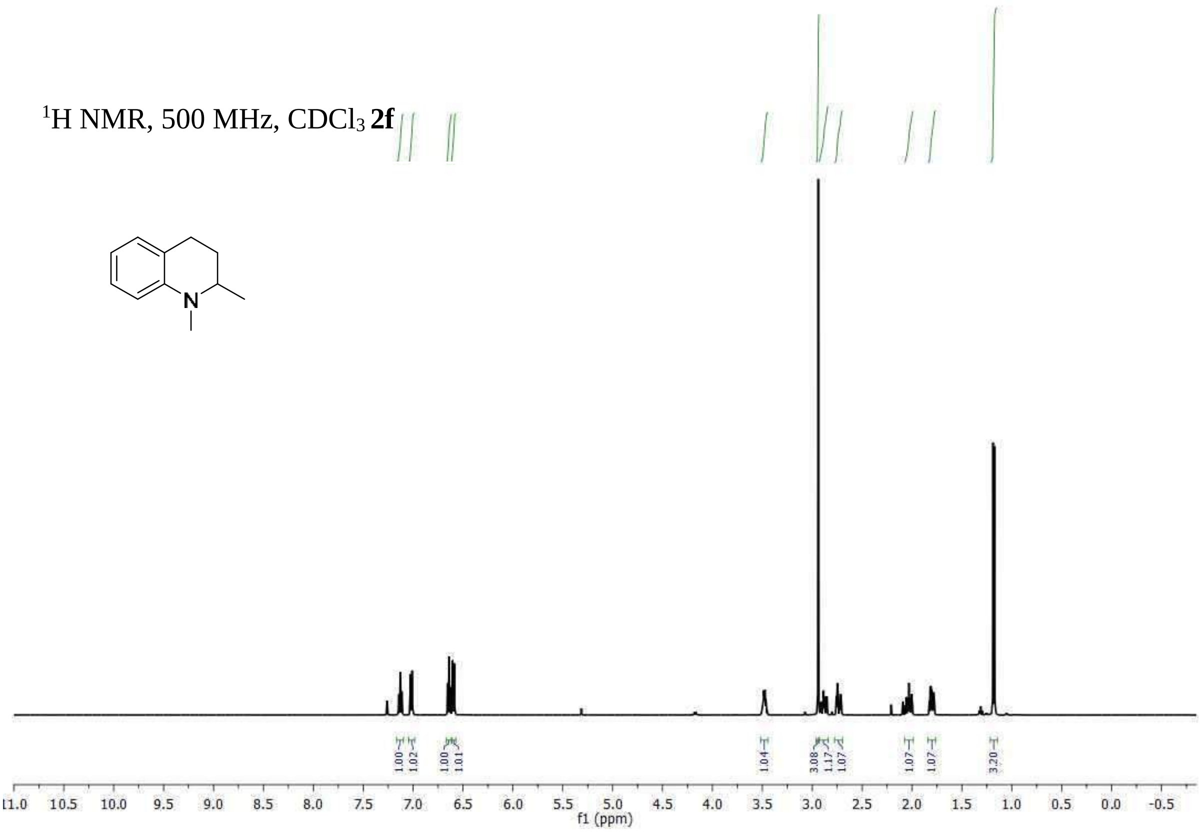

^{13}C NMR, 125 MHz, $CDCl_3$ **2f**

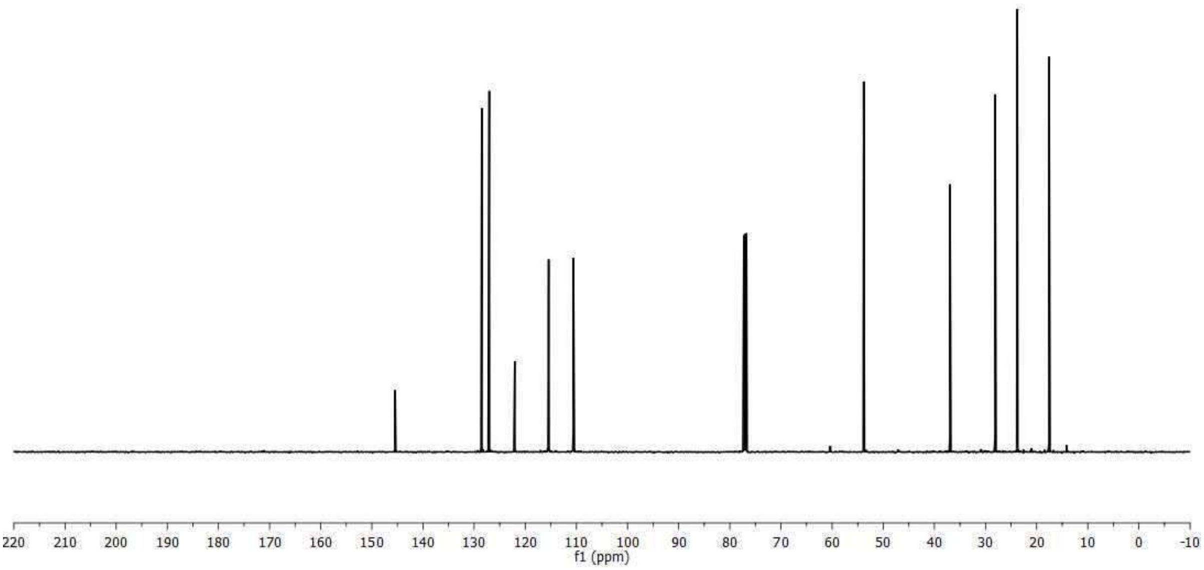

^{1}H NMR, 400 MHz, $CDCl_3$ **2g**

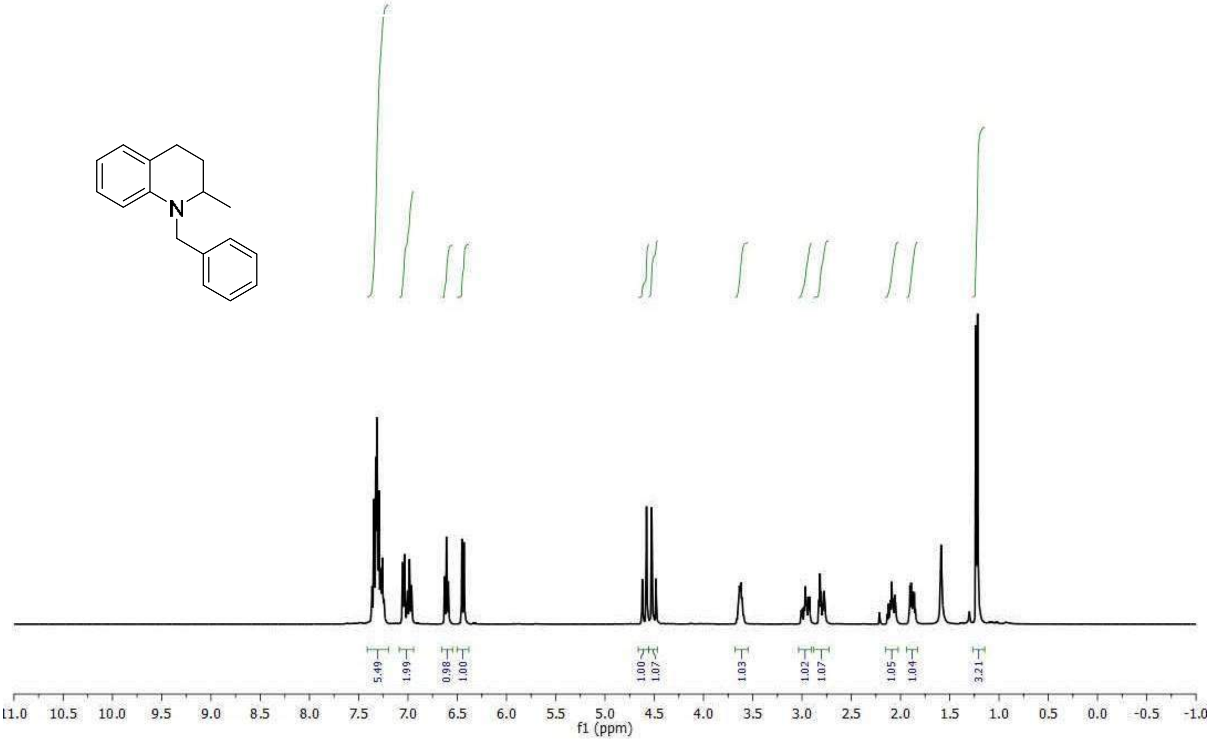

^{13}C NMR, 100 MHz, $CDCl_3$ **2g**

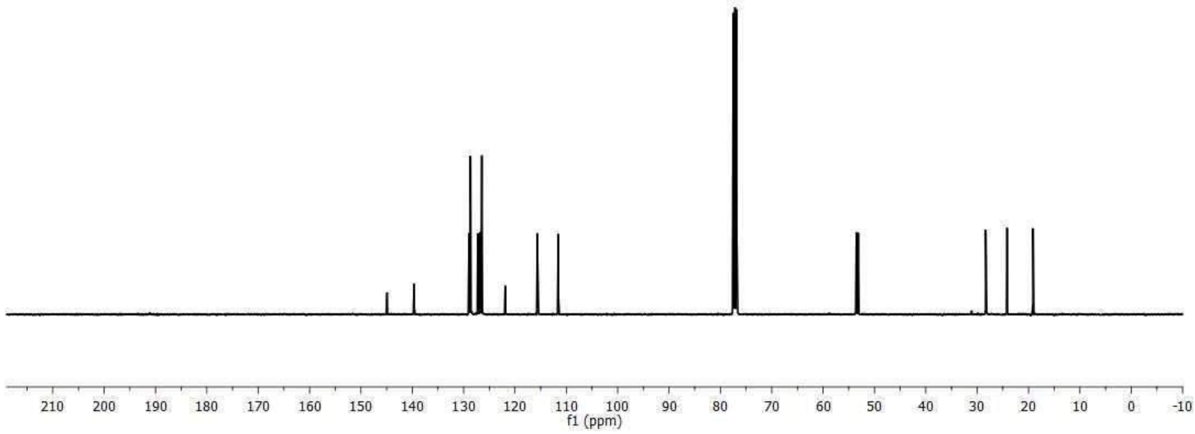

^{1}H NMR, 500 MHz, $CDCl_3$ **2h**

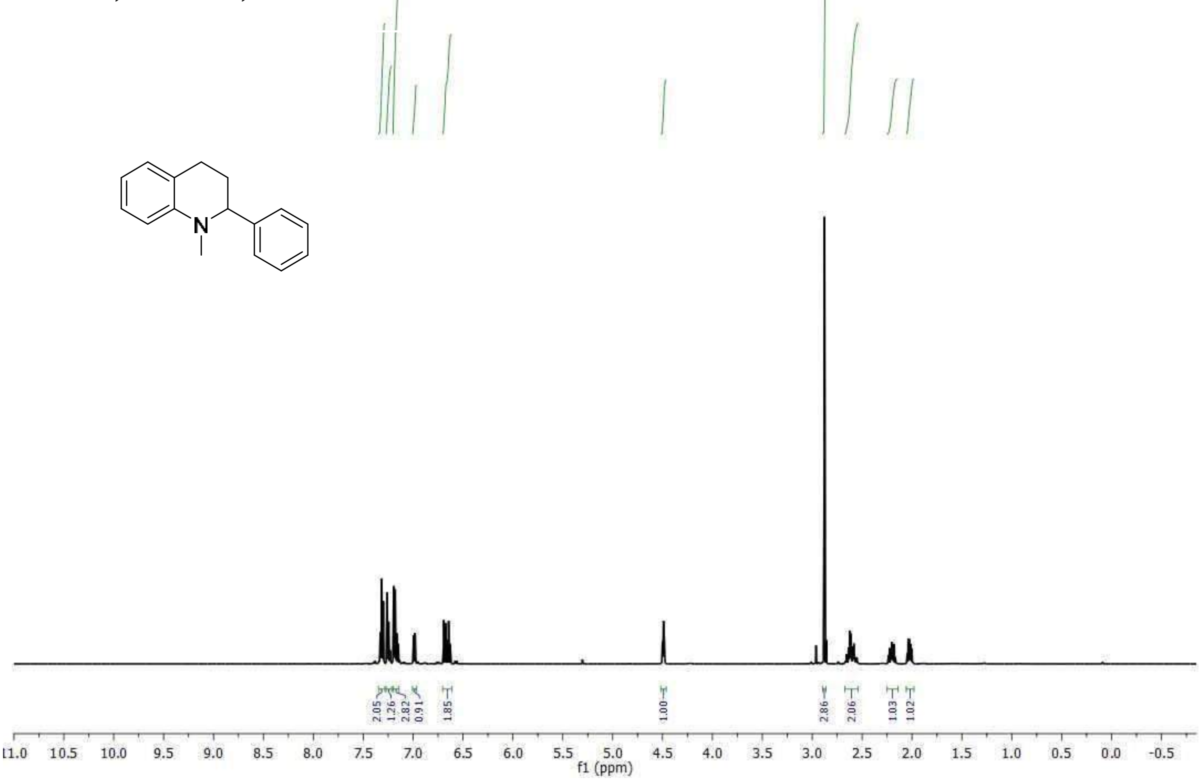

^{13}C NMR, 125 MHz, $CDCl_3$ **2h**

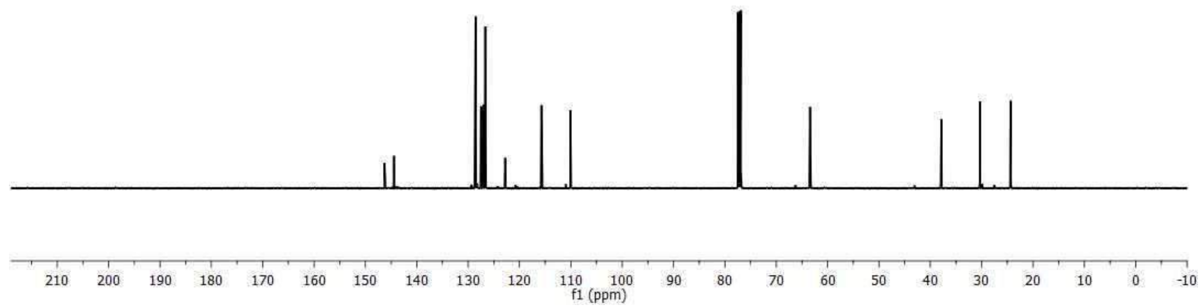

^{1}H NMR, 500 MHz, $CDCl_3$ **2i**

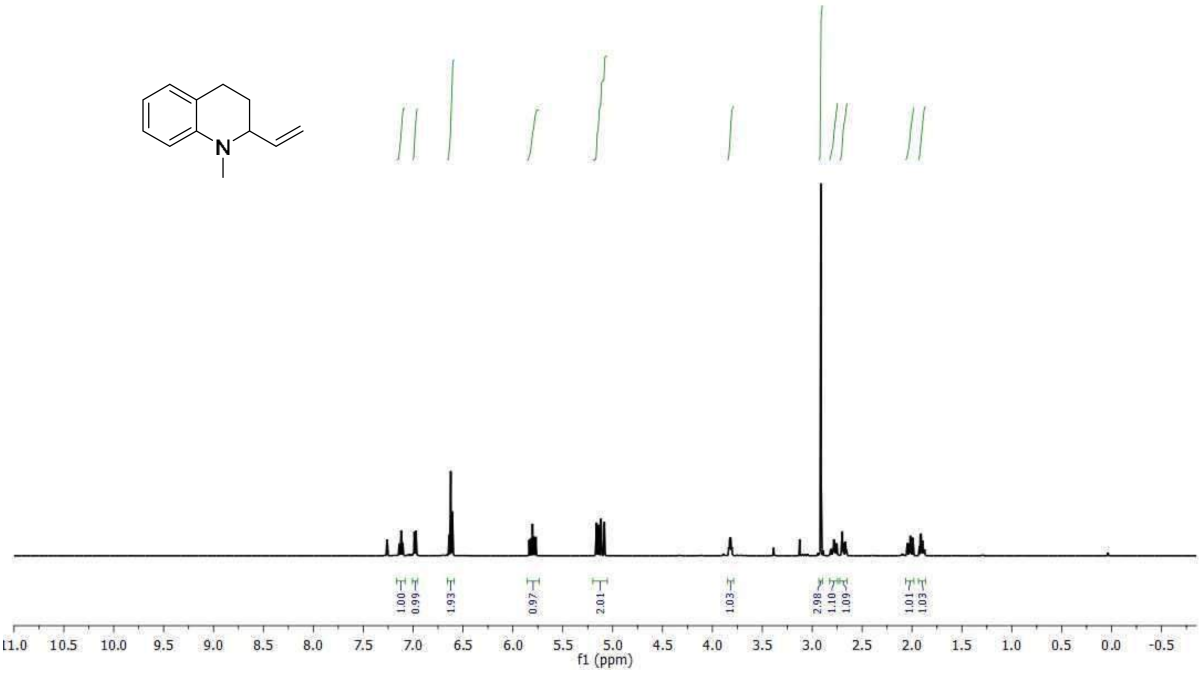

^{13}C NMR, 125 MHz, $CDCl_3$ **2i**

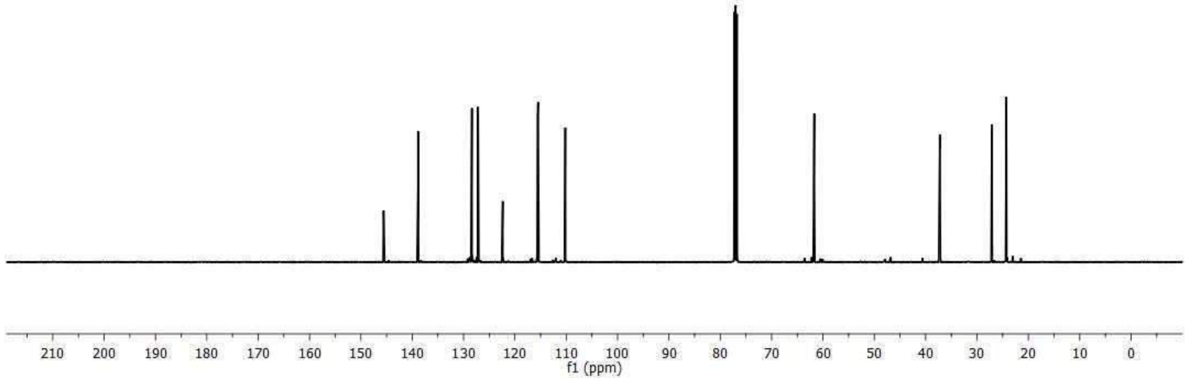

^{1}H NMR, 400 MHz, $CDCl_3$ **2j**

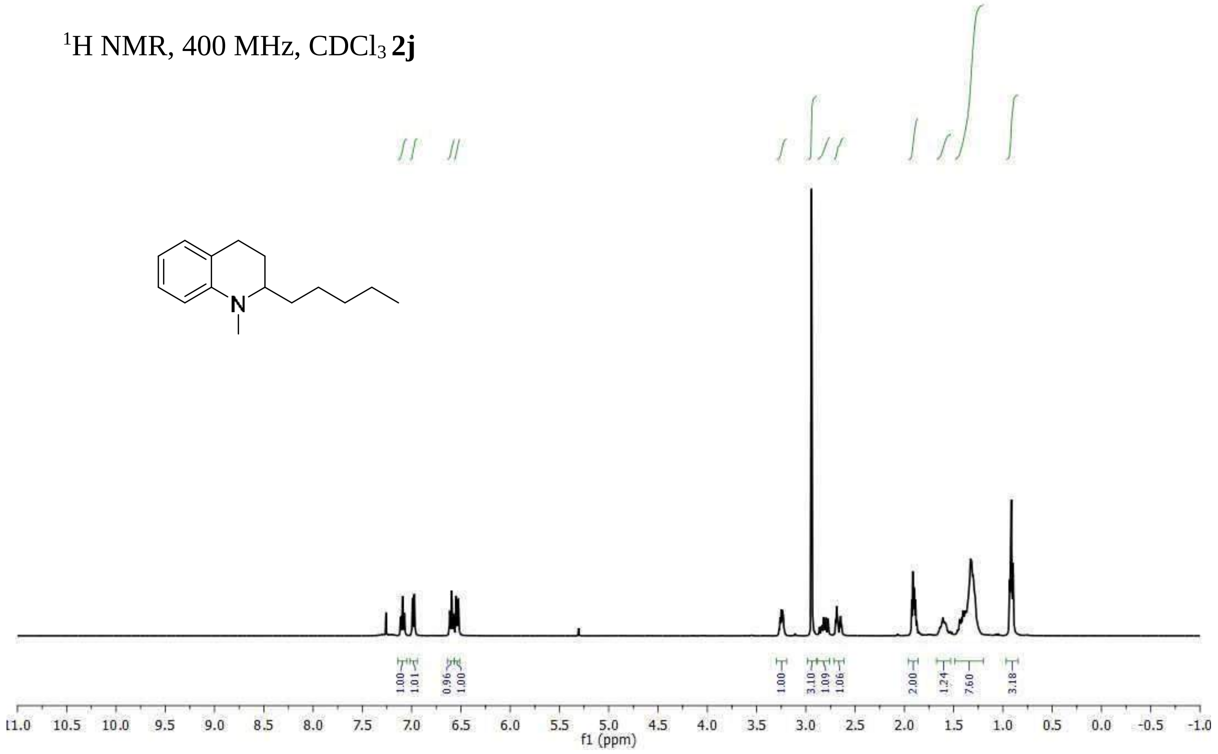

^{13}C NMR, 100 MHz, $CDCl_3$ **2j**

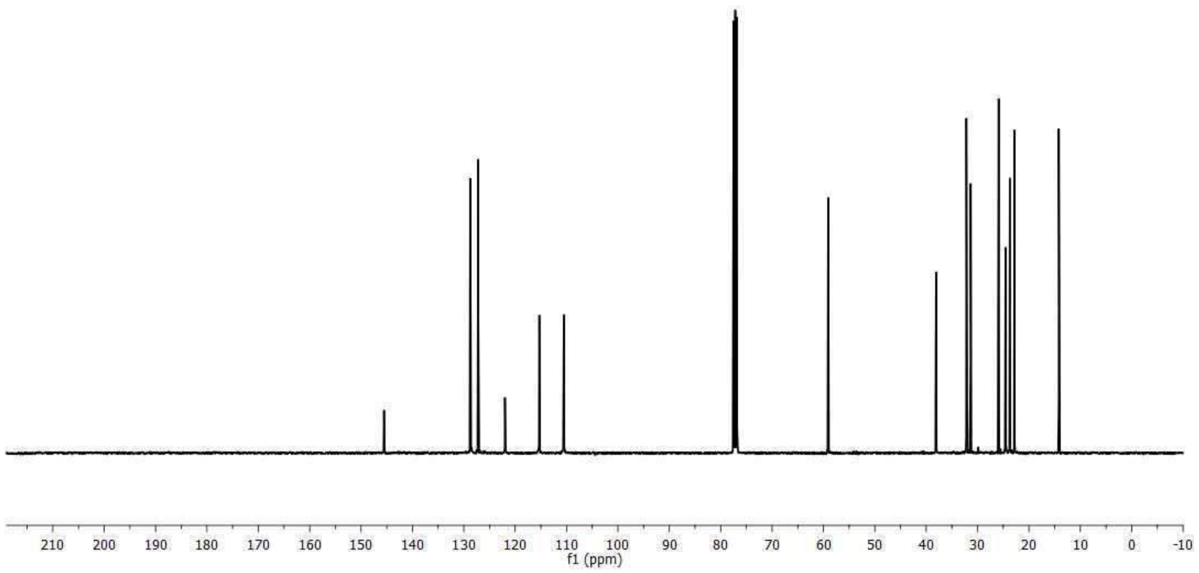

^{1}H NMR, 400 MHz, $CDCl_3$ **2k**

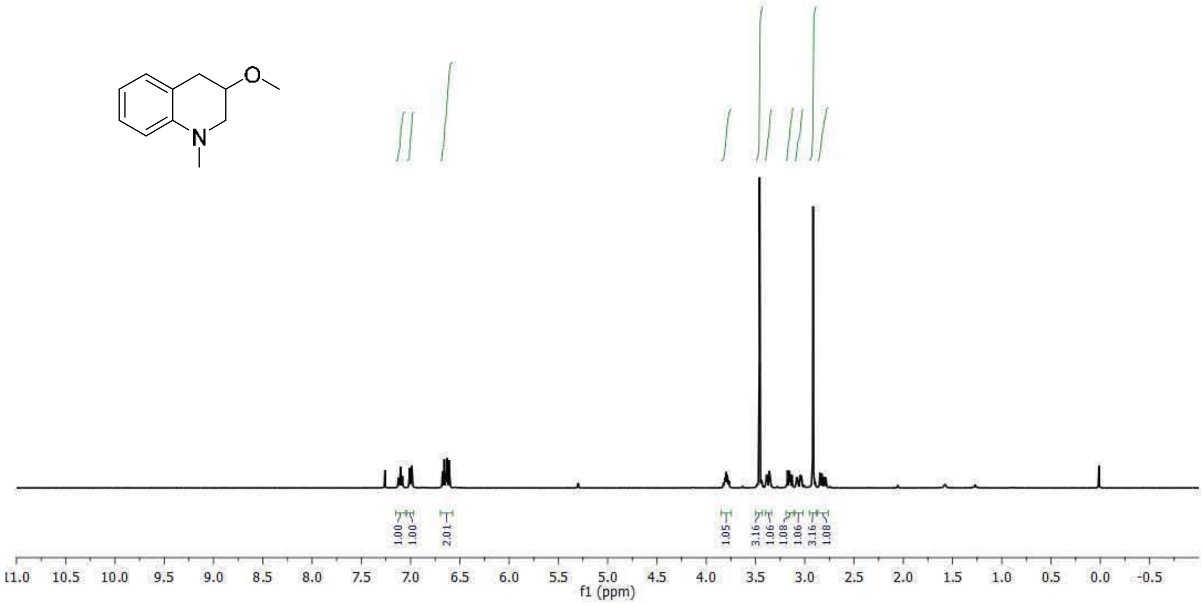

^{13}C NMR, 100 MHz, $CDCl_3$ **2k**

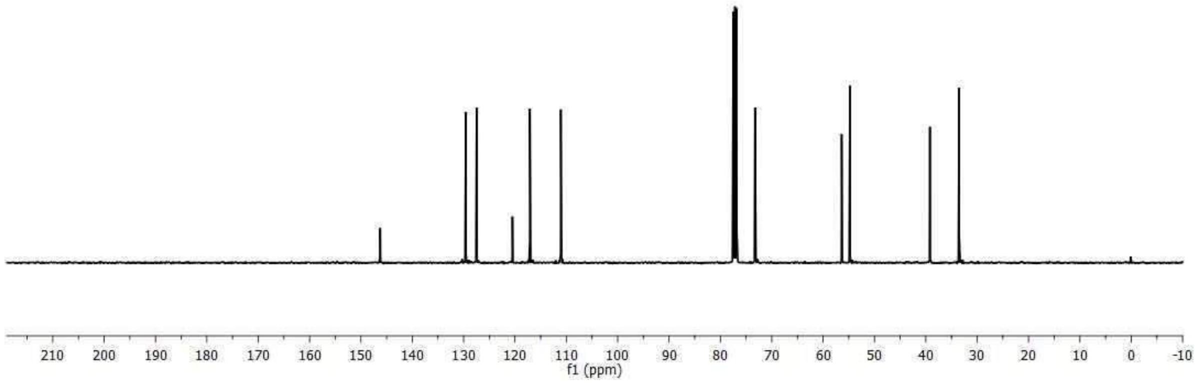

^{1}H NMR, 500 MHz, $CDCl_3$ **2l**

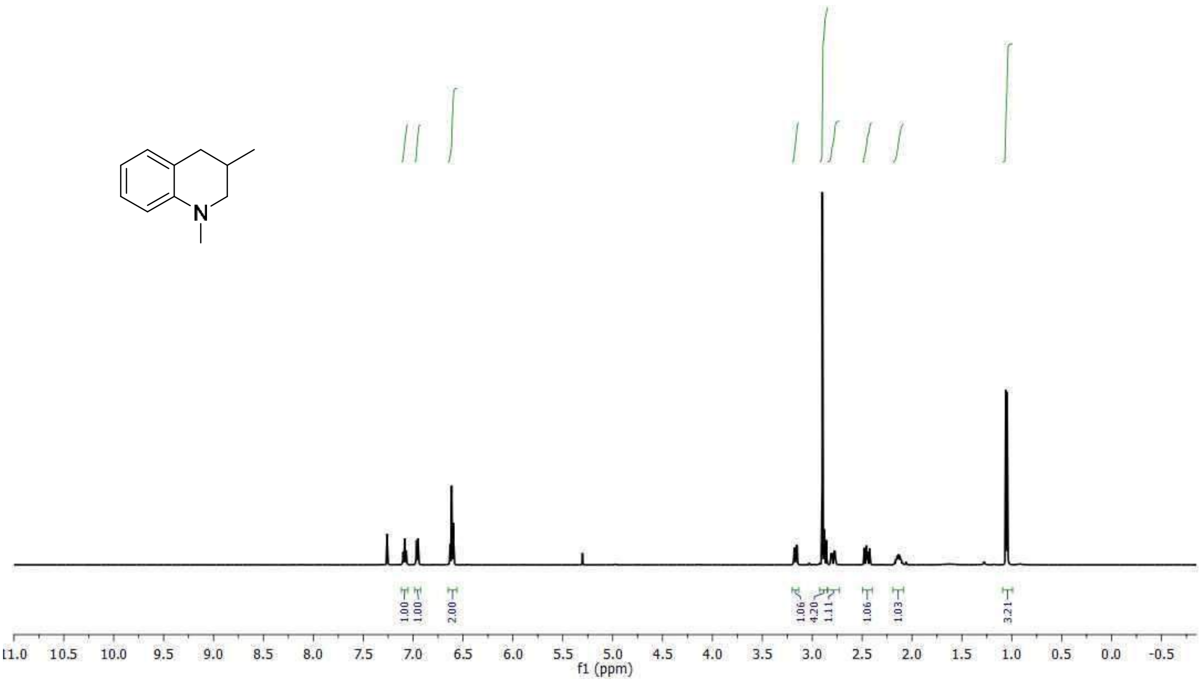

^{13}C NMR, 125 MHz, $CDCl_3$ **2l**

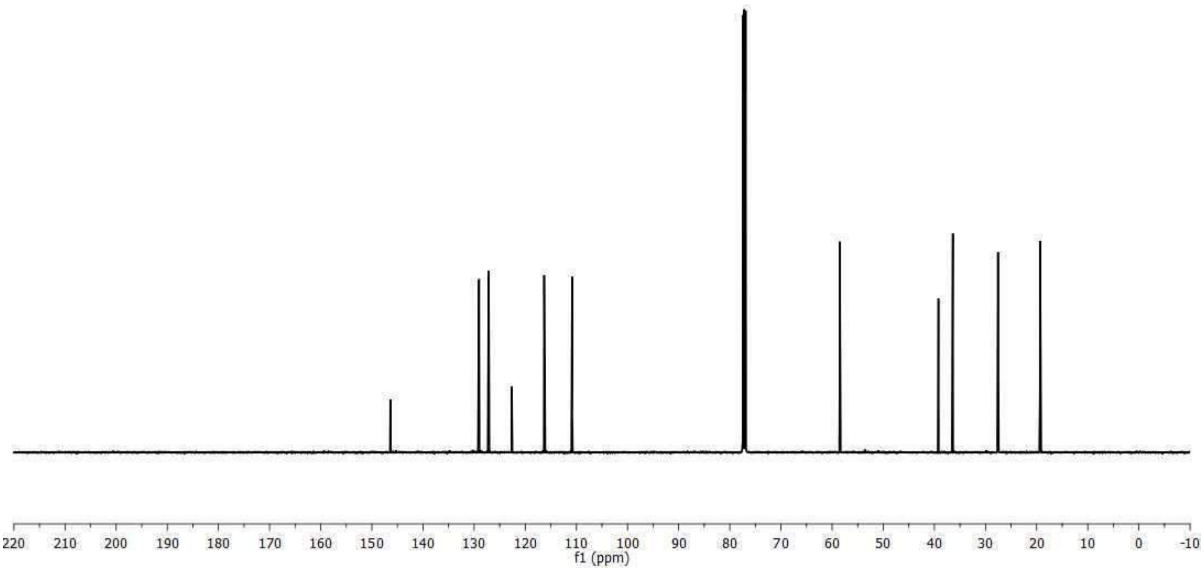

^{1}H NMR, 300 MHz, $CDCl_3$ **2m**

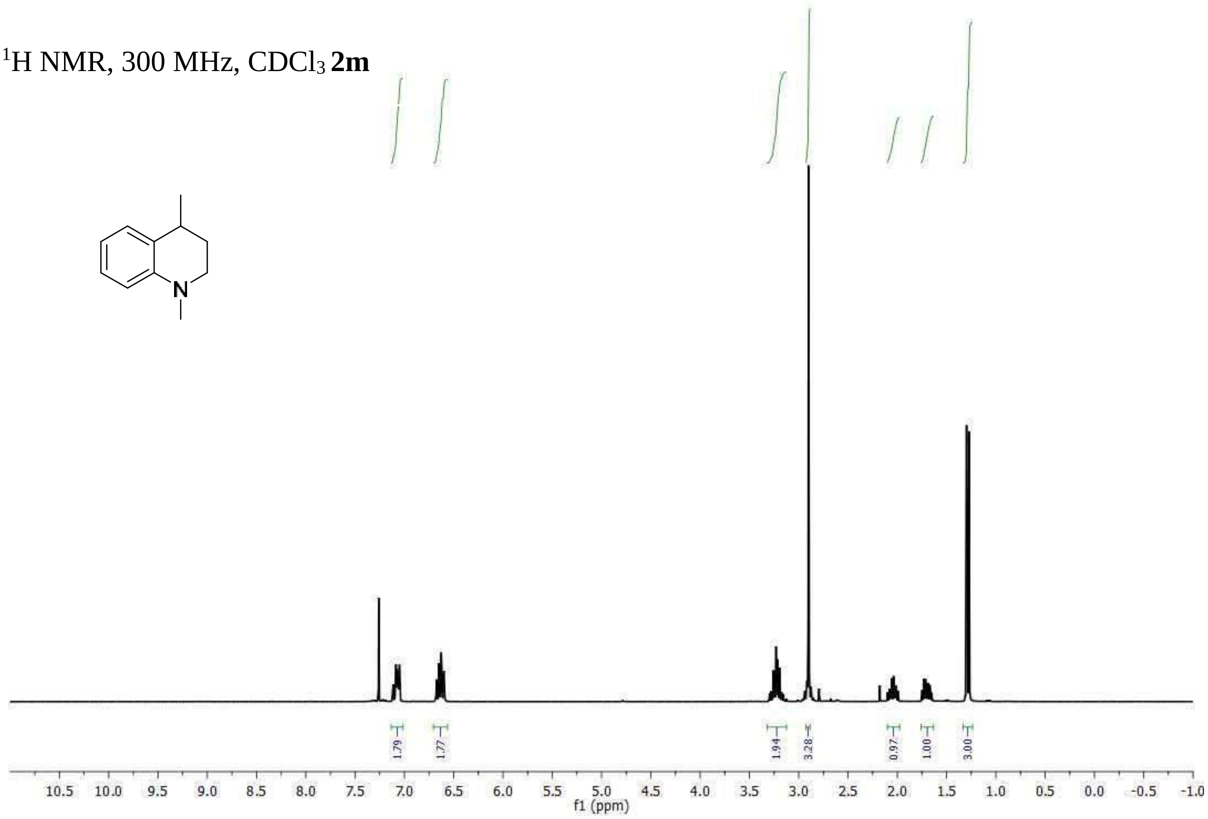

^{13}C NMR, 125 MHz, $CDCl_3$ **2m**

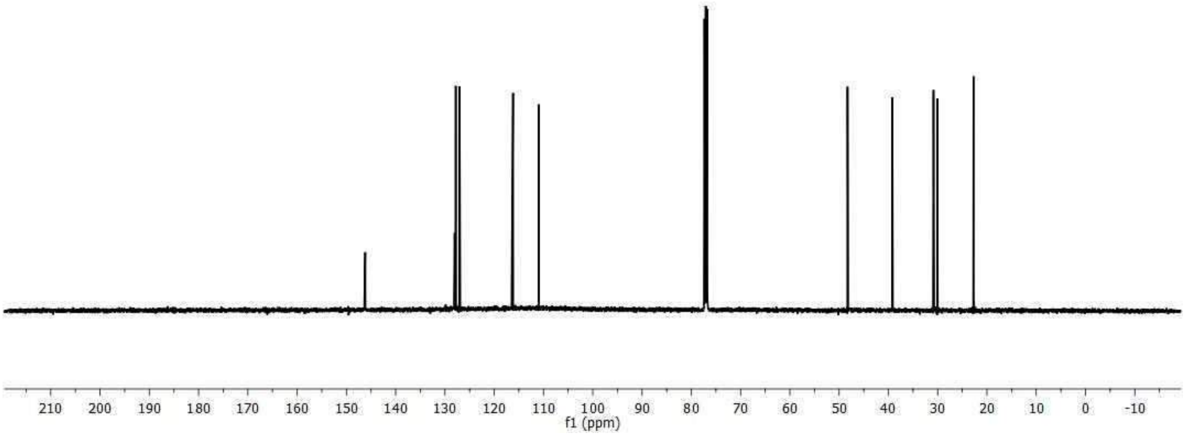

^{1}H NMR, 300 MHz, $CDCl_3$ **2n**

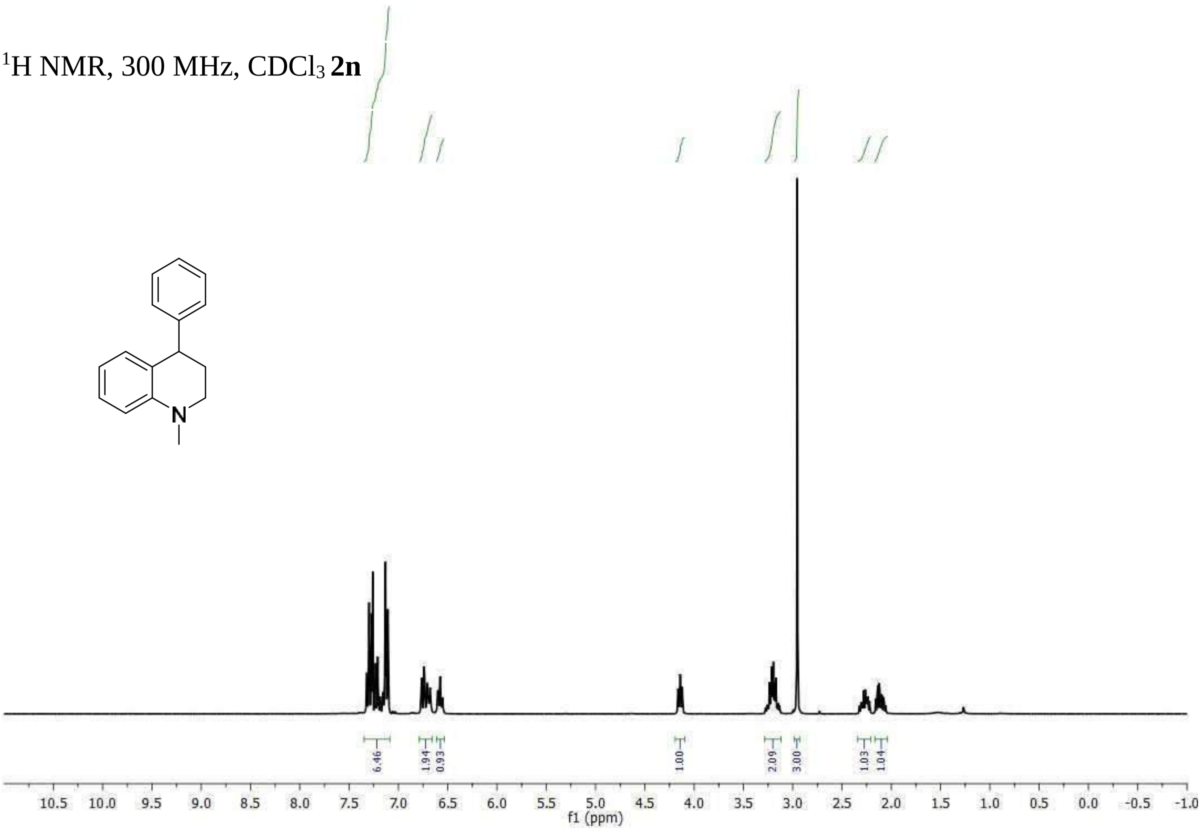

^{13}C NMR, 75 MHz, $CDCl_3$ **2n**

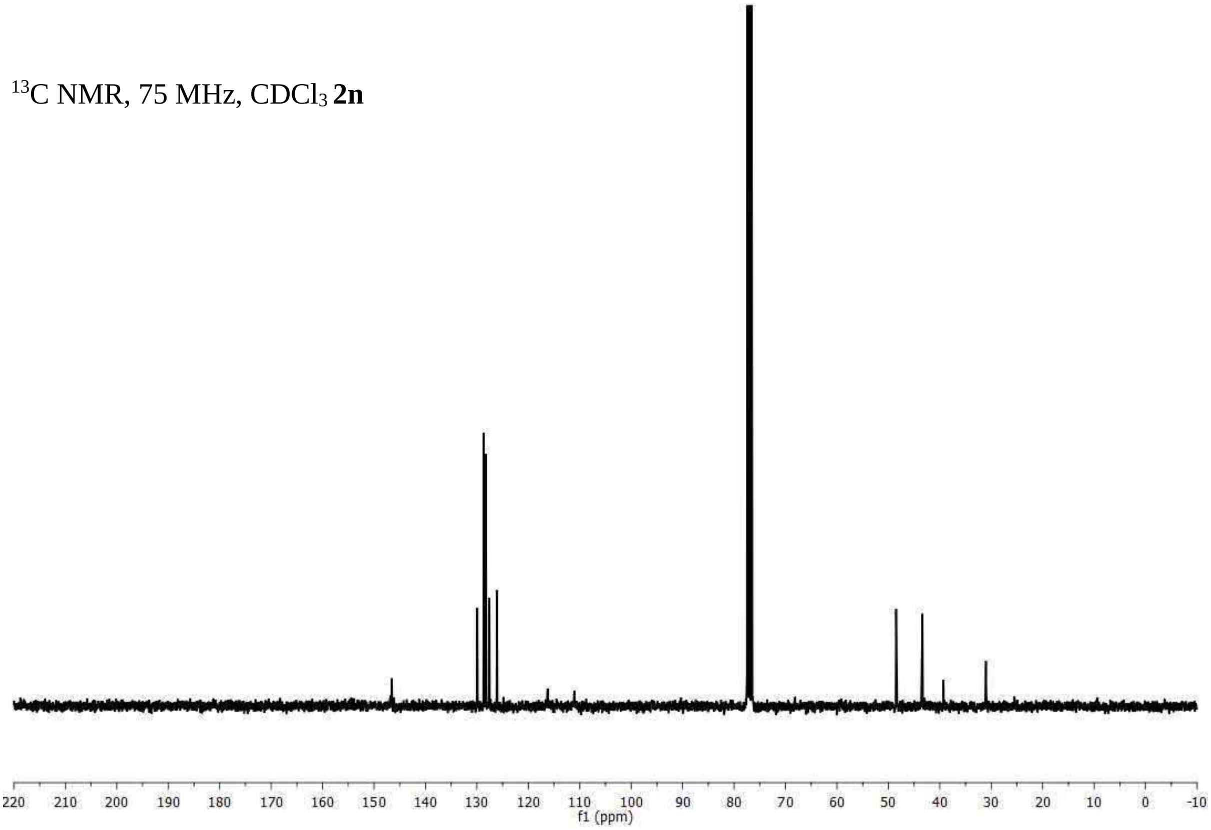

^{1}H NMR, 400 MHz, $CDCl_3$ **2o**

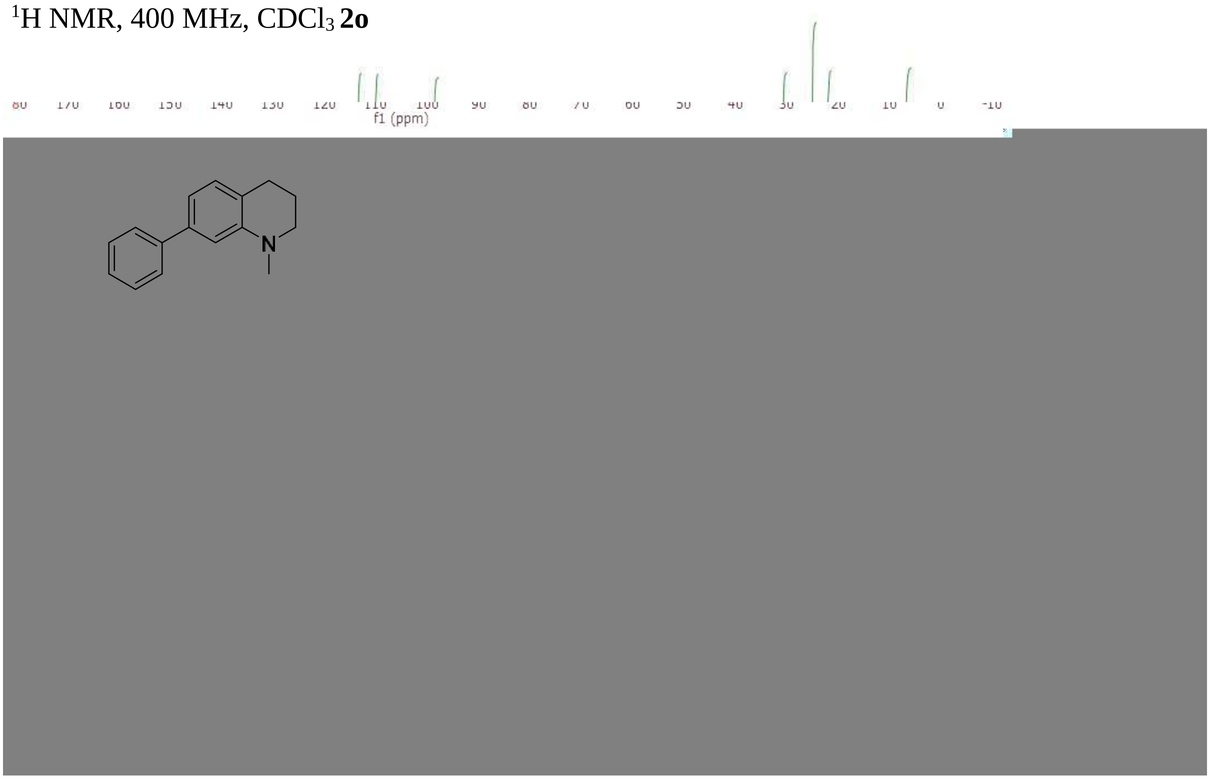

^{13}C NMR, 100 MHz, $CDCl_3$ **2o**

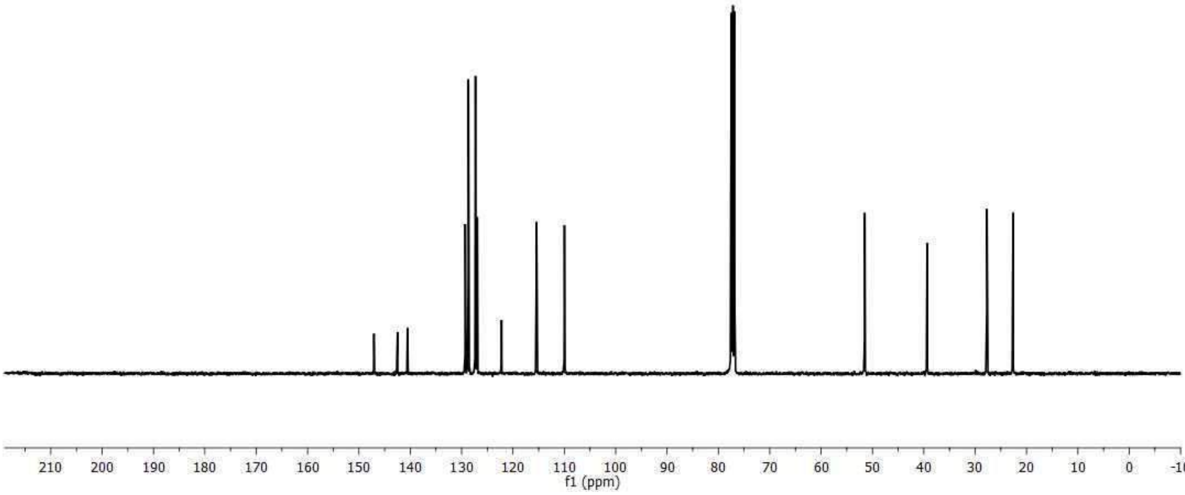

^{1}H NMR, 500 MHz, $CDCl_3$ **2p**

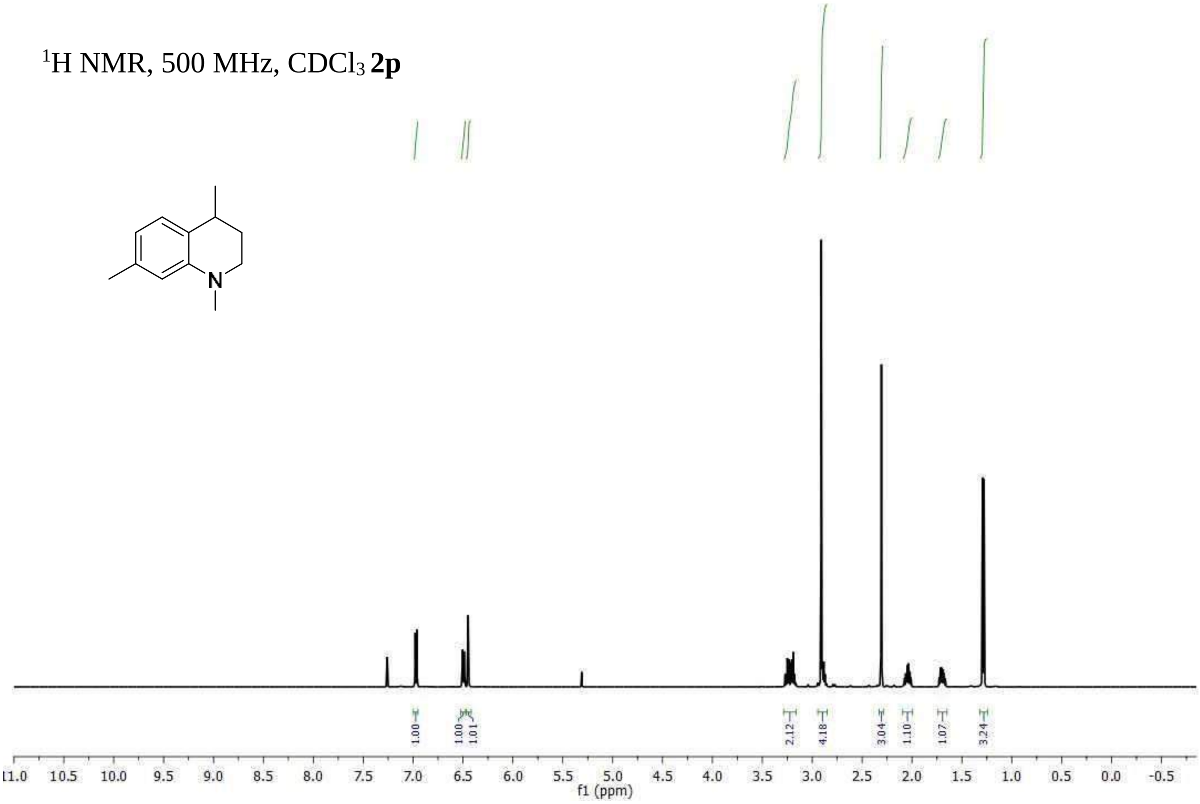

^{13}C NMR, 125 MHz, $CDCl_3$ **2p**

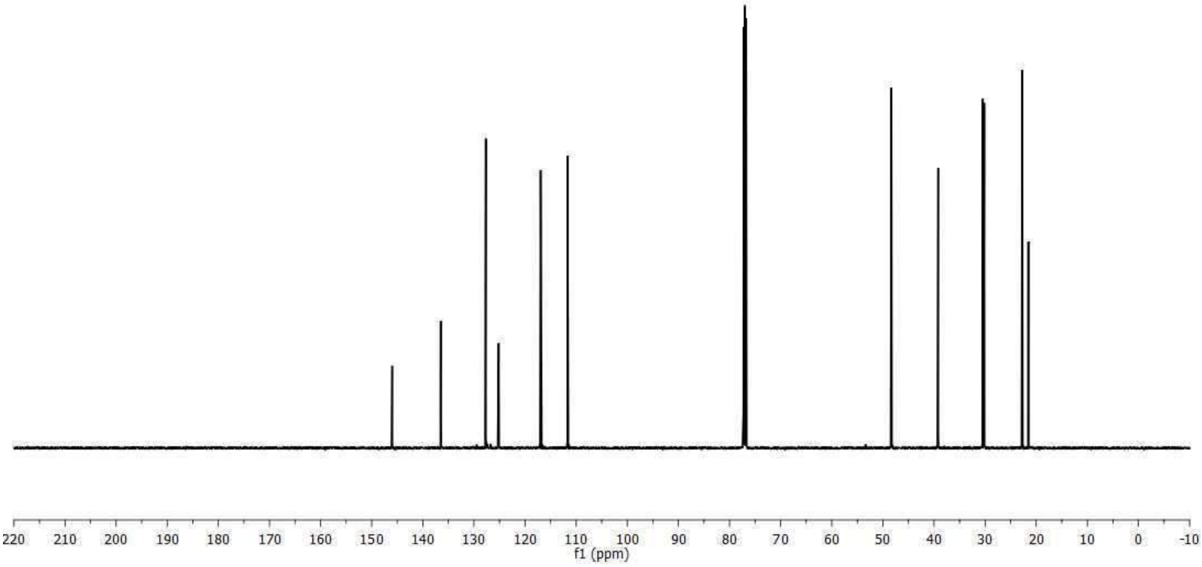

^{1}H NMR, 400 MHz, $CDCl_3$ **2q**

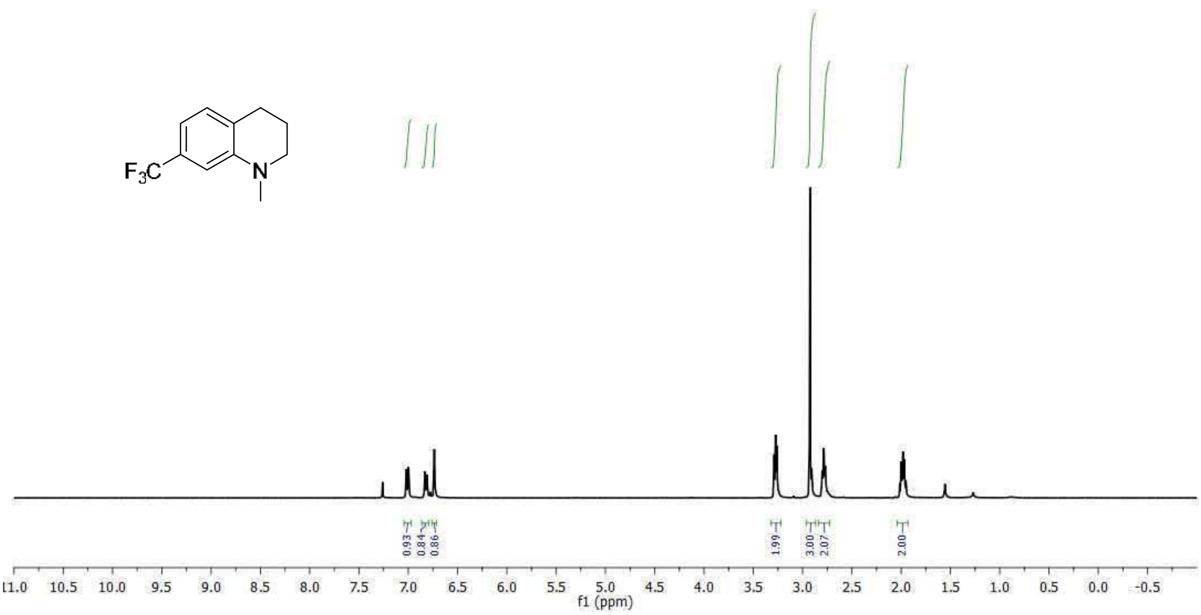

^{13}C NMR, 100 MHz, $CDCl_3$ **2q**

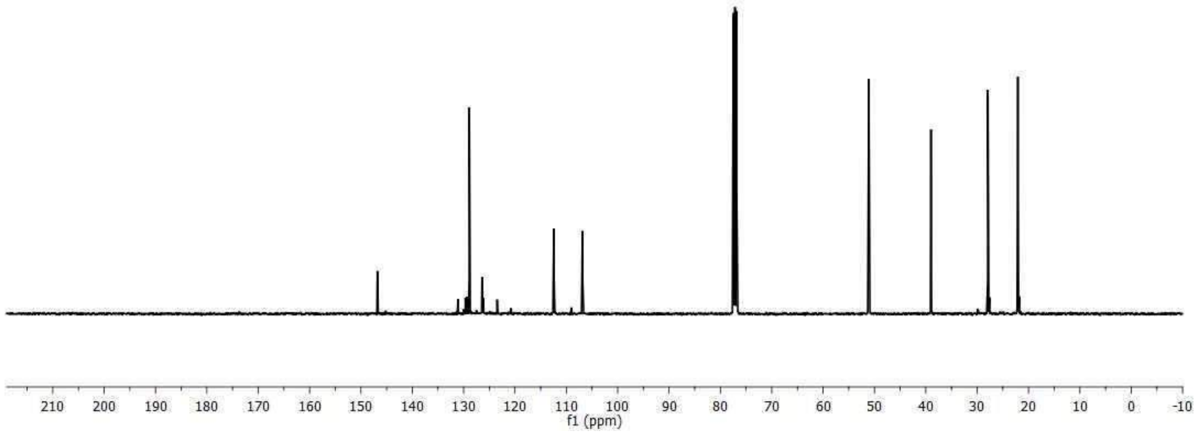

^{1}H NMR, 300 MHz, $CDCl_3$ **2r**

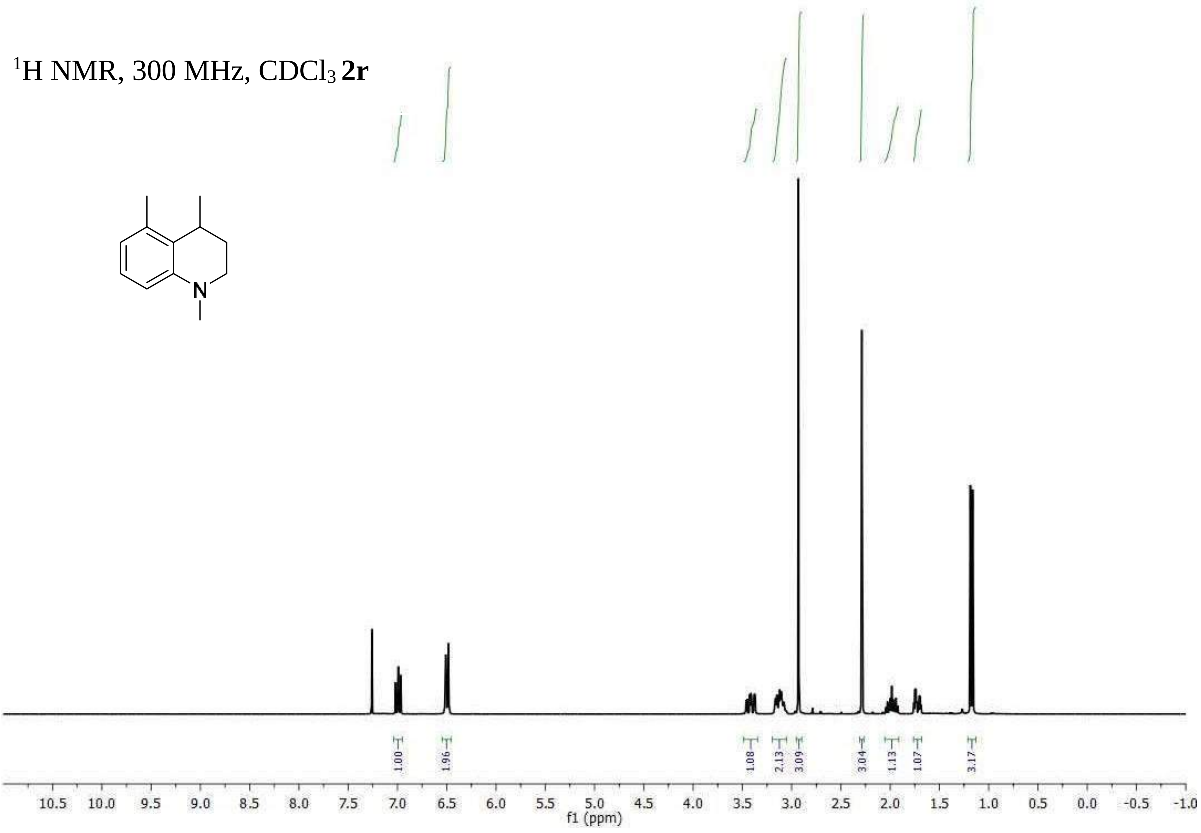

^{13}C NMR, 125 MHz, $CDCl_3$ **2r**

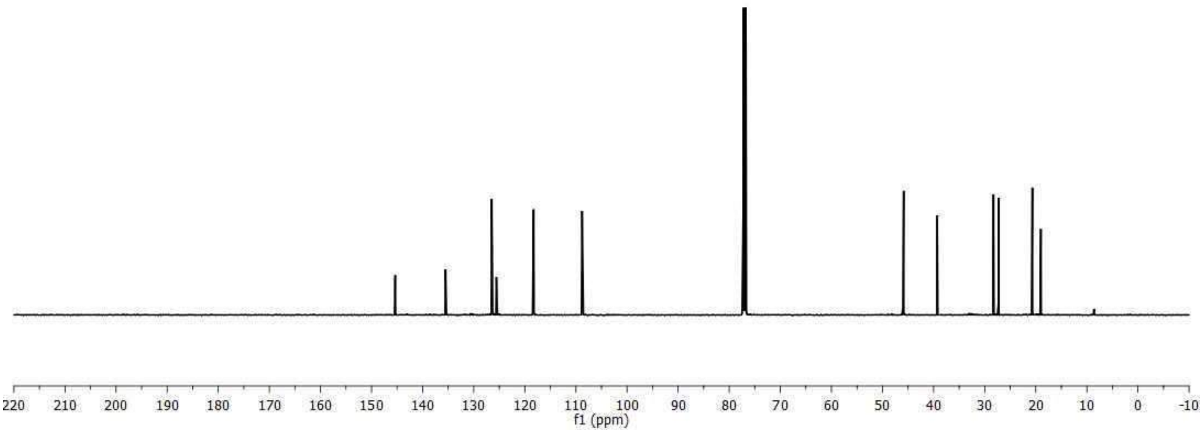

^{1}H NMR, 400 MHz, $CDCl_3$ **2s**

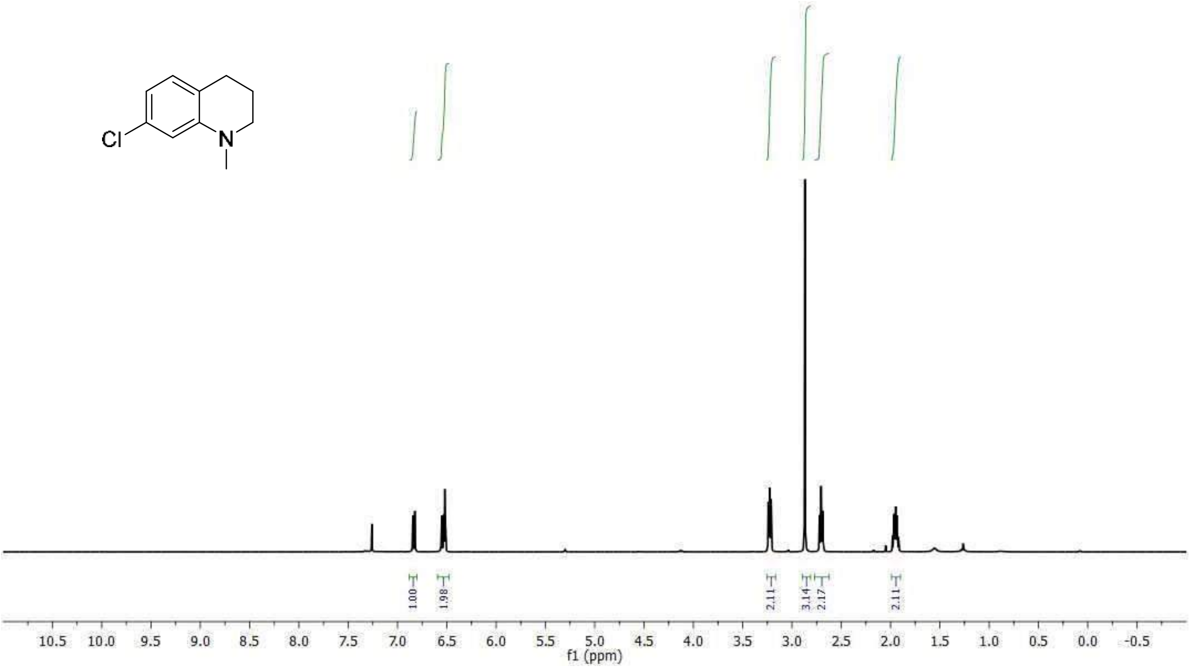

^{13}C NMR, 100 MHz, $CDCl_3$ **2s**

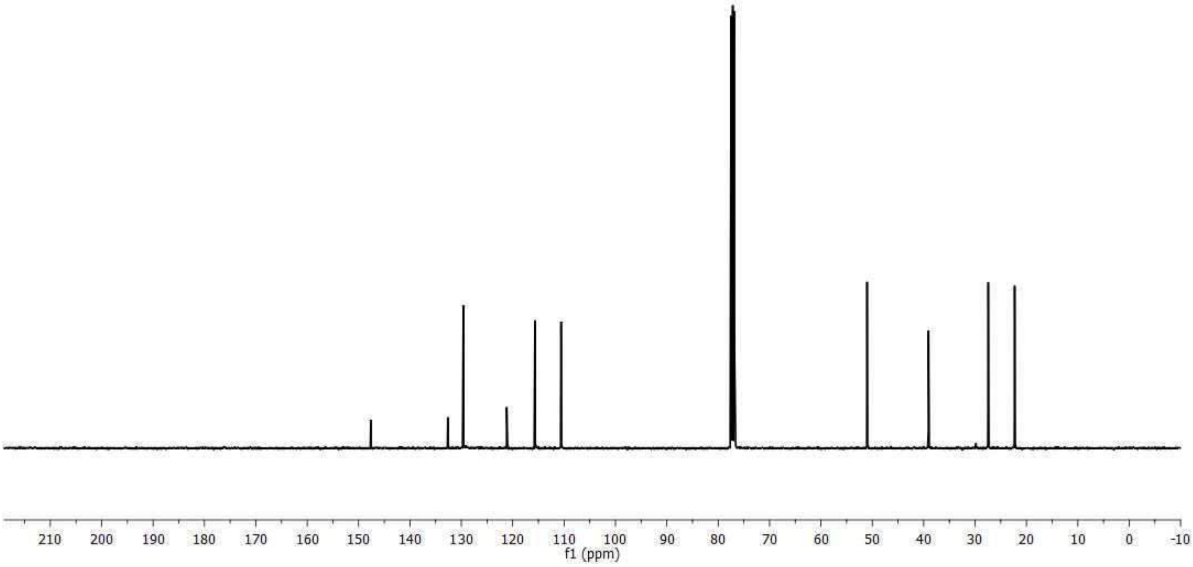

^{1}H NMR, 500 MHz, $CDCl_3$ **2t**

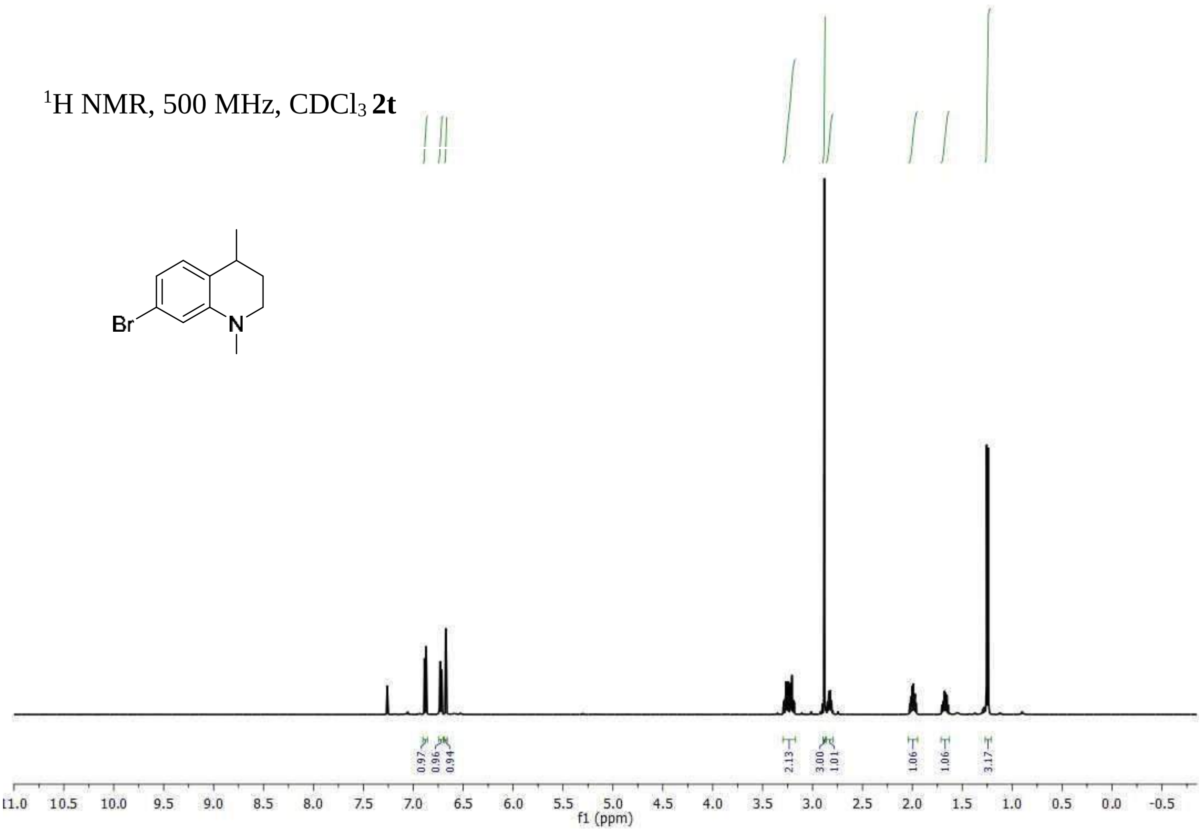

^{13}C NMR, 125 MHz, $CDCl_3$ **2t**

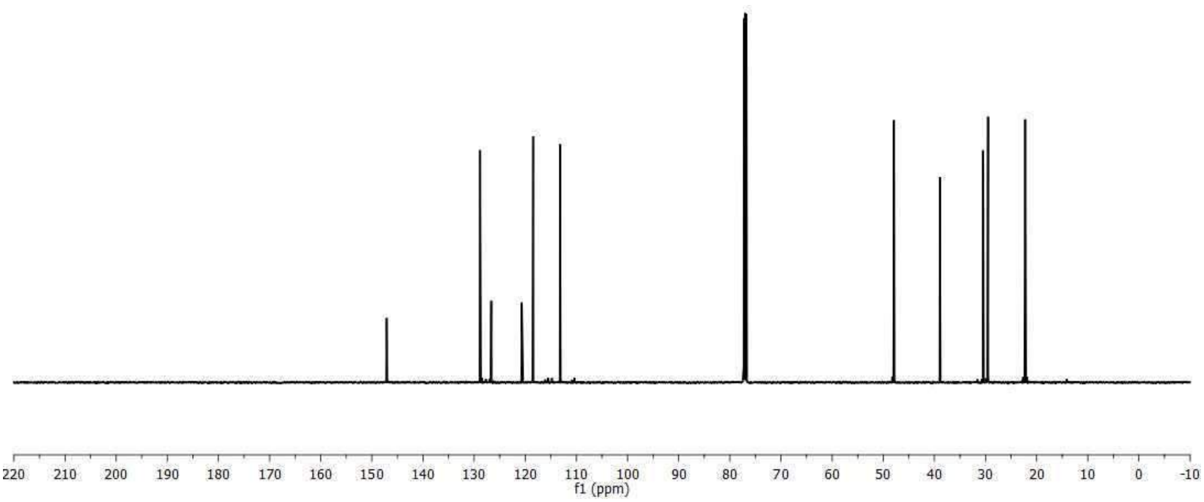

^{1}H NMR, 400 MHz, $CDCl_3$ **2u**

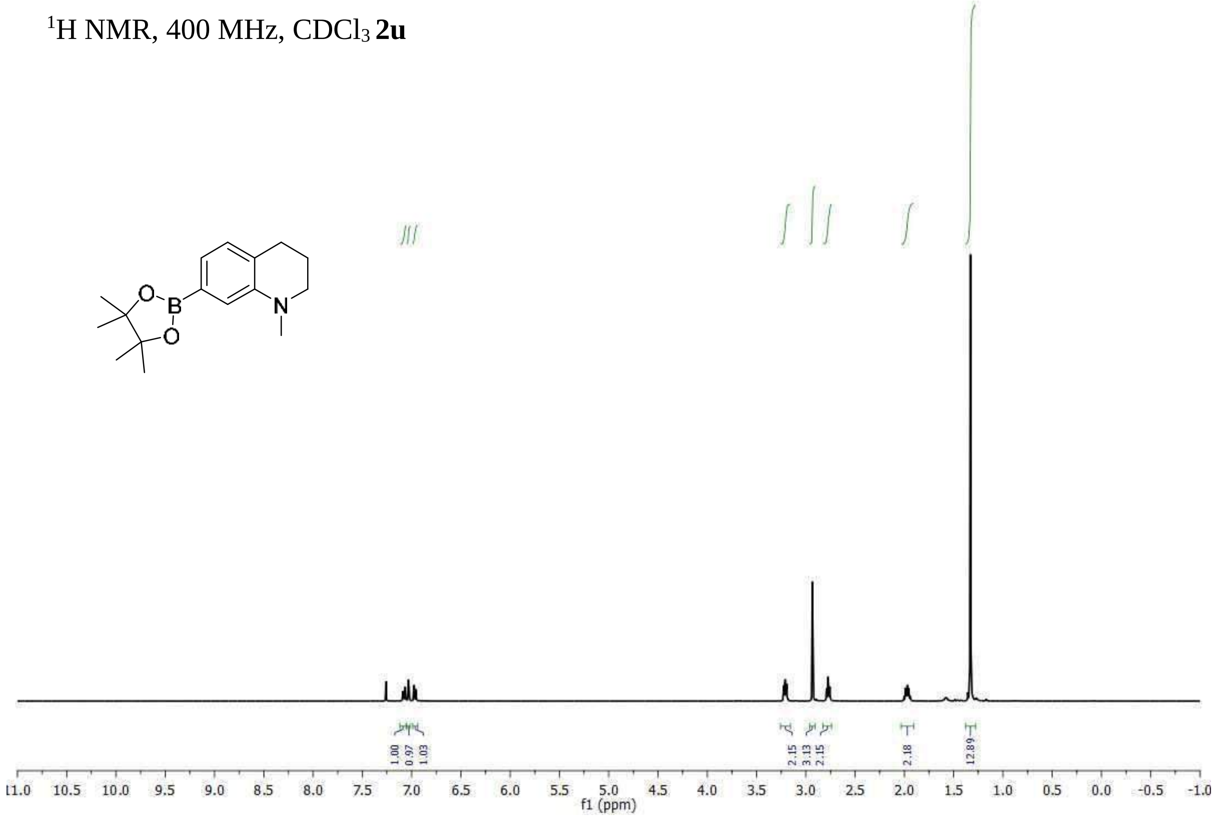

^{13}C NMR, 100 MHz, $CDCl_3$ **2u**

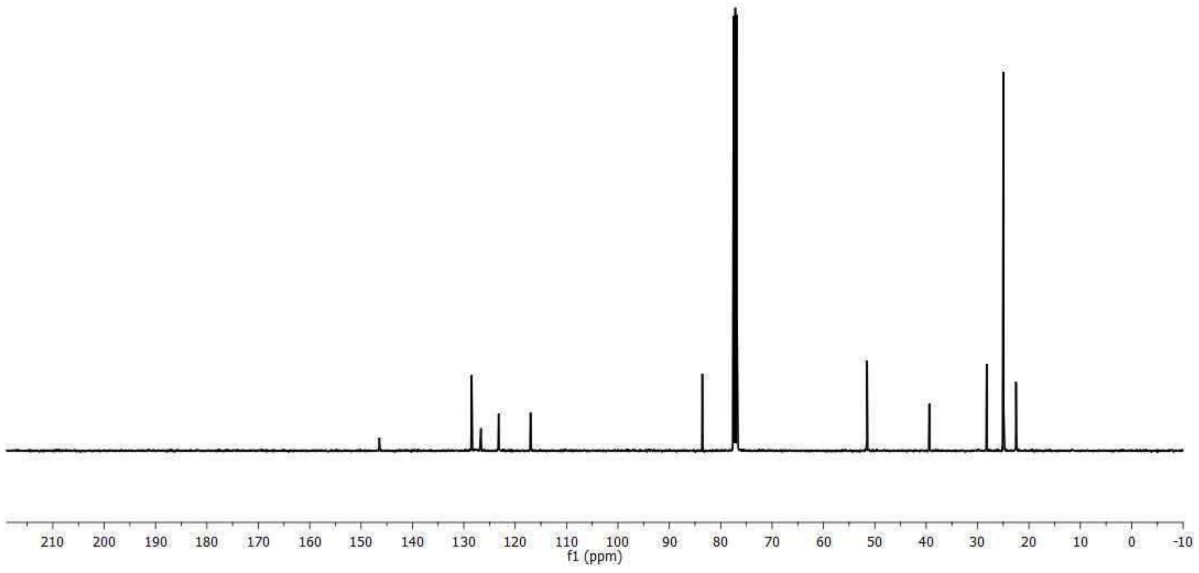

^{1}H NMR, 500 MHz, $CDCl_3$ **2v**

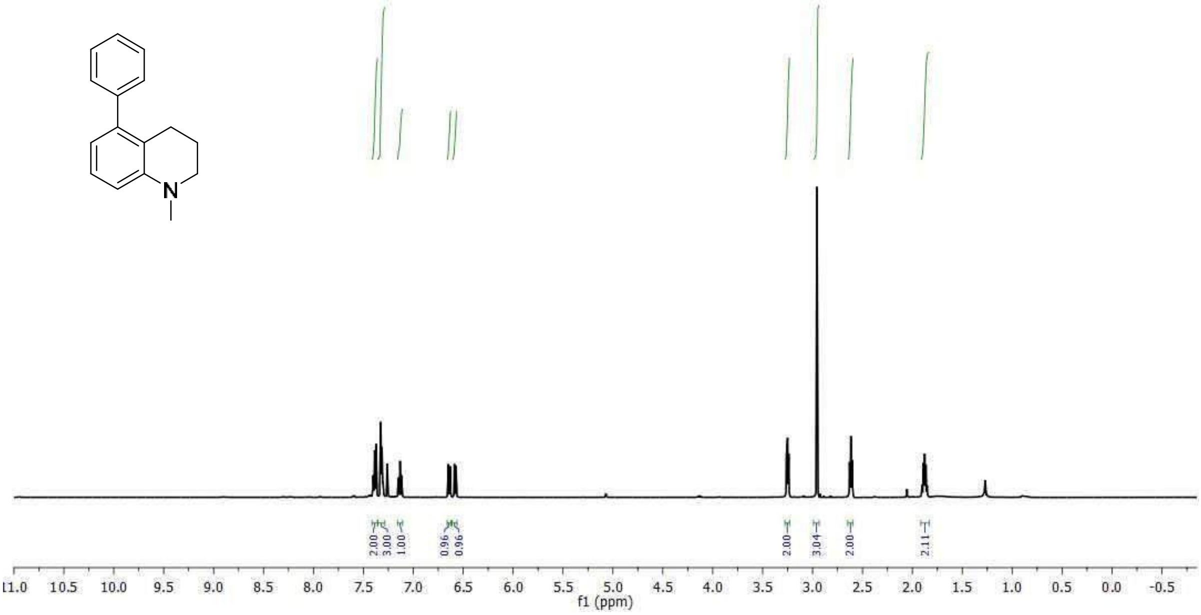

^{13}C NMR, 125 MHz, $CDCl_3$ **2v**

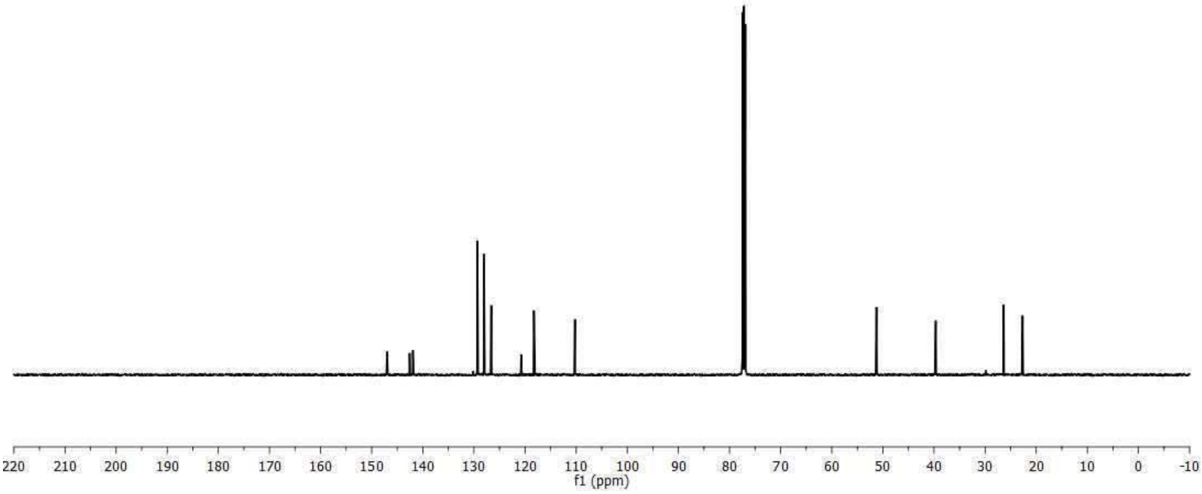

^{1}H NMR, 500 MHz, $CDCl_3$ **2w**

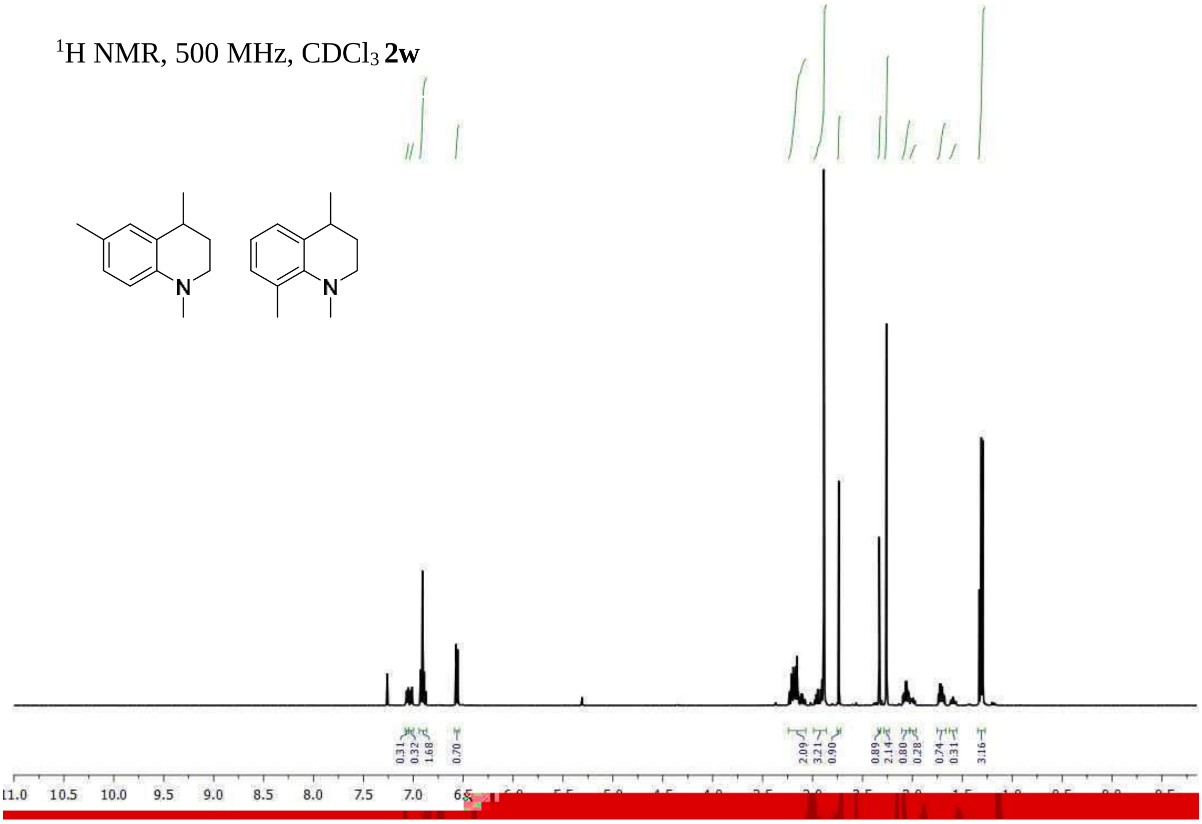

^{13}C NMR, 125 MHz, $CDCl_3$ **2w**

^{1}H NMR, 400 MHz, $CDCl_3$ **2x**

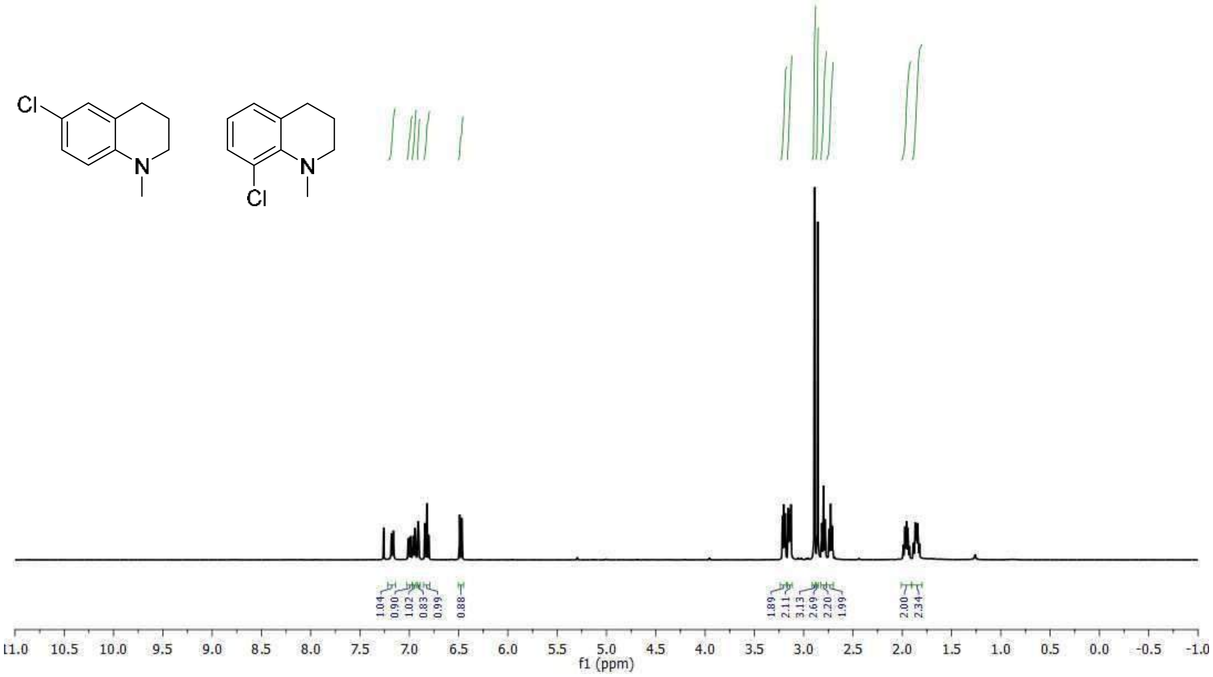

^{13}C NMR, 100 MHz, $CDCl_3$ **2x**

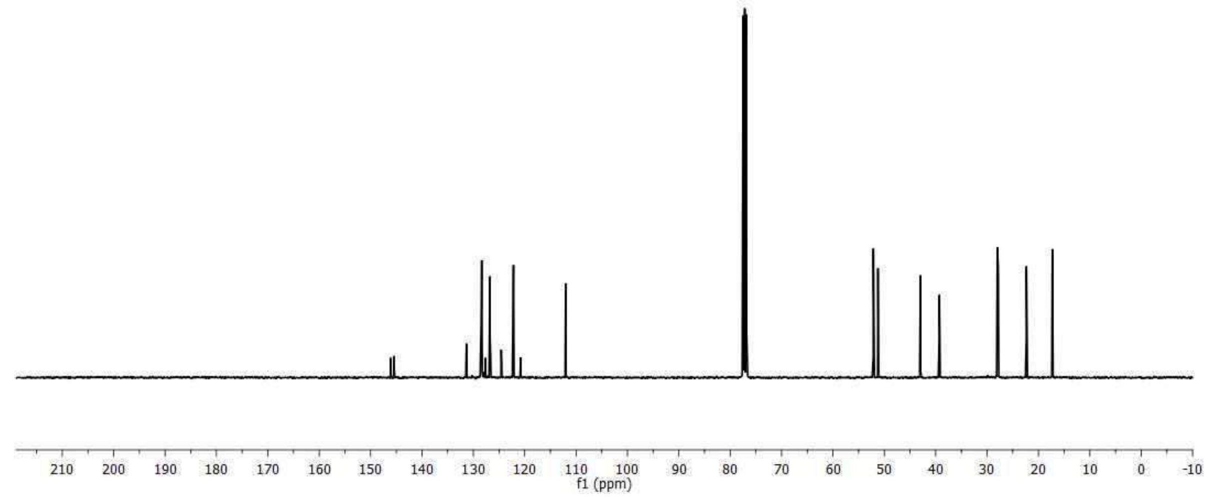

^{1}H NMR, 500 MHz, $CDCl_3$ **5a**

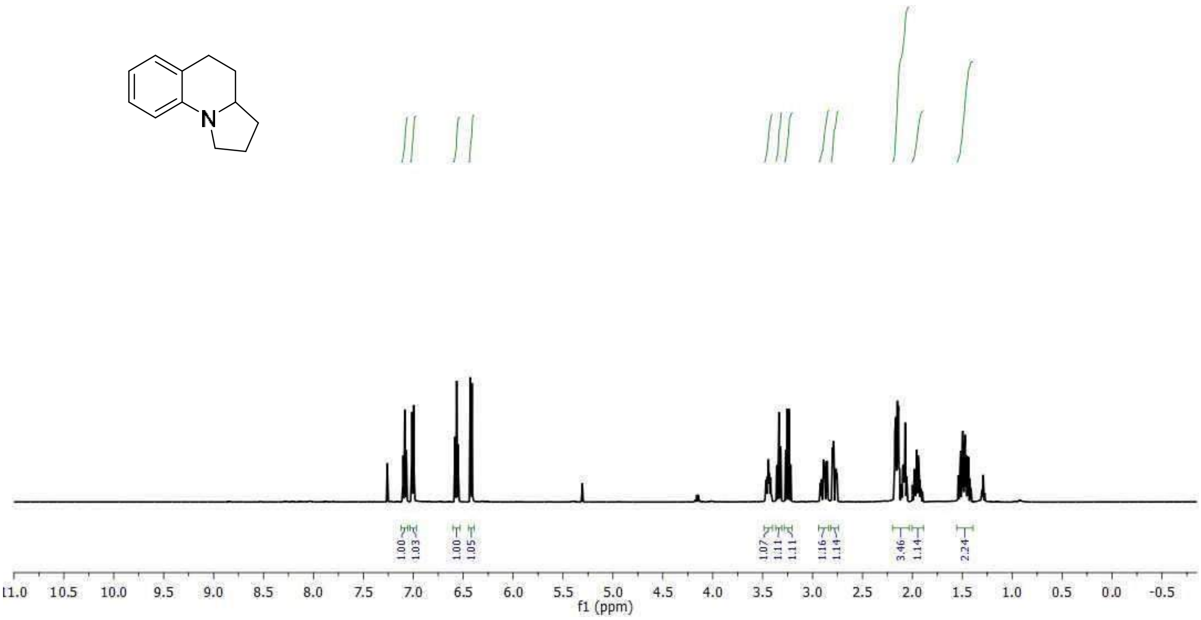

^{13}C NMR, 125 MHz, $CDCl_3$ **5a**

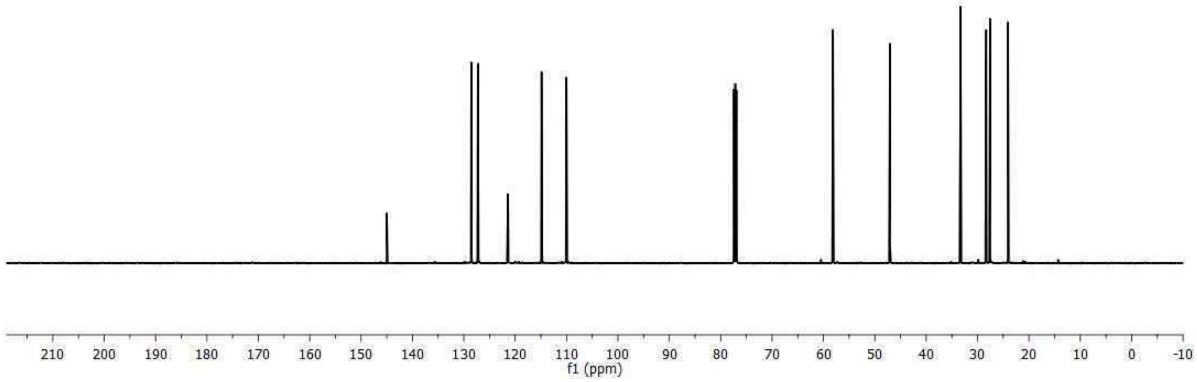

^{1}H NMR, 500 MHz, $CDCl_3$ **5b**

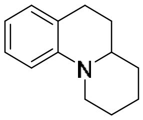

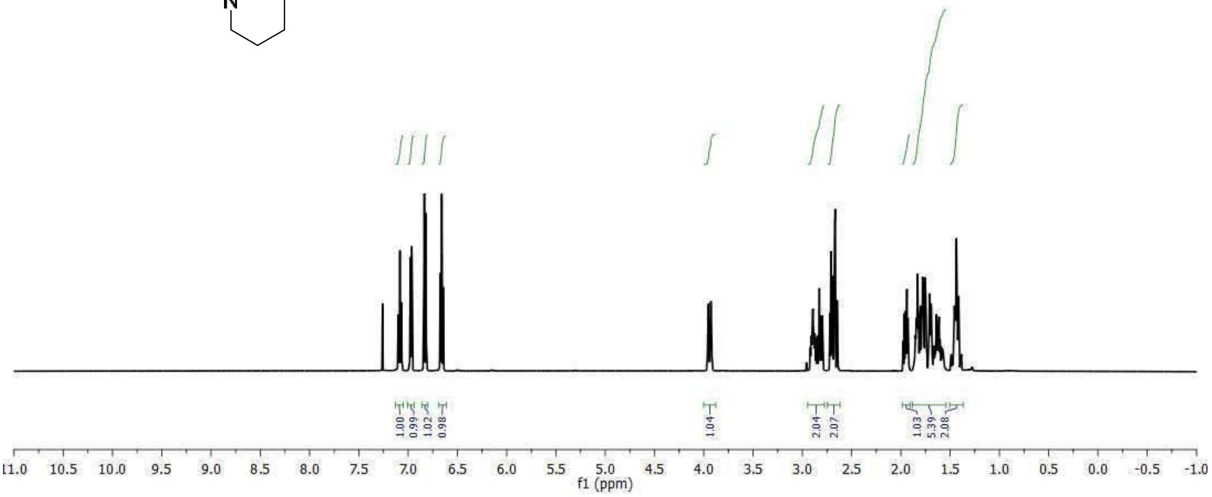

^{13}C NMR, 125 MHz, $CDCl_3$ **5b**

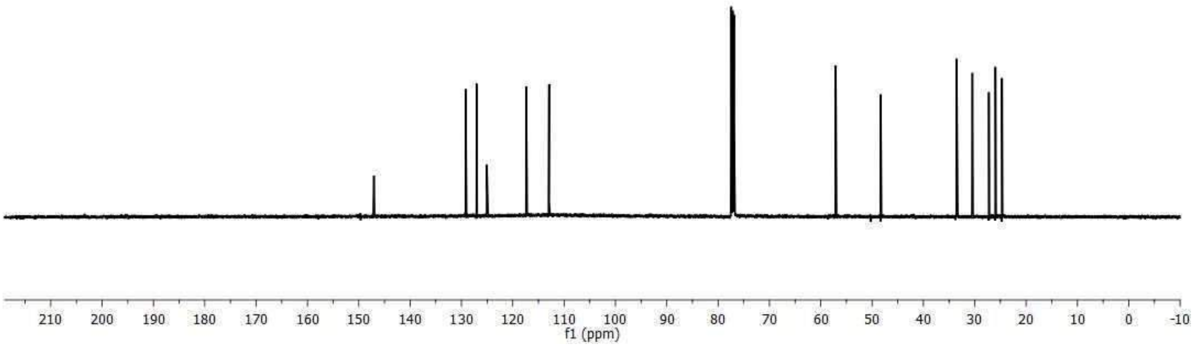

^{1}H NMR, 500 MHz, $CDCl_3$ **7**

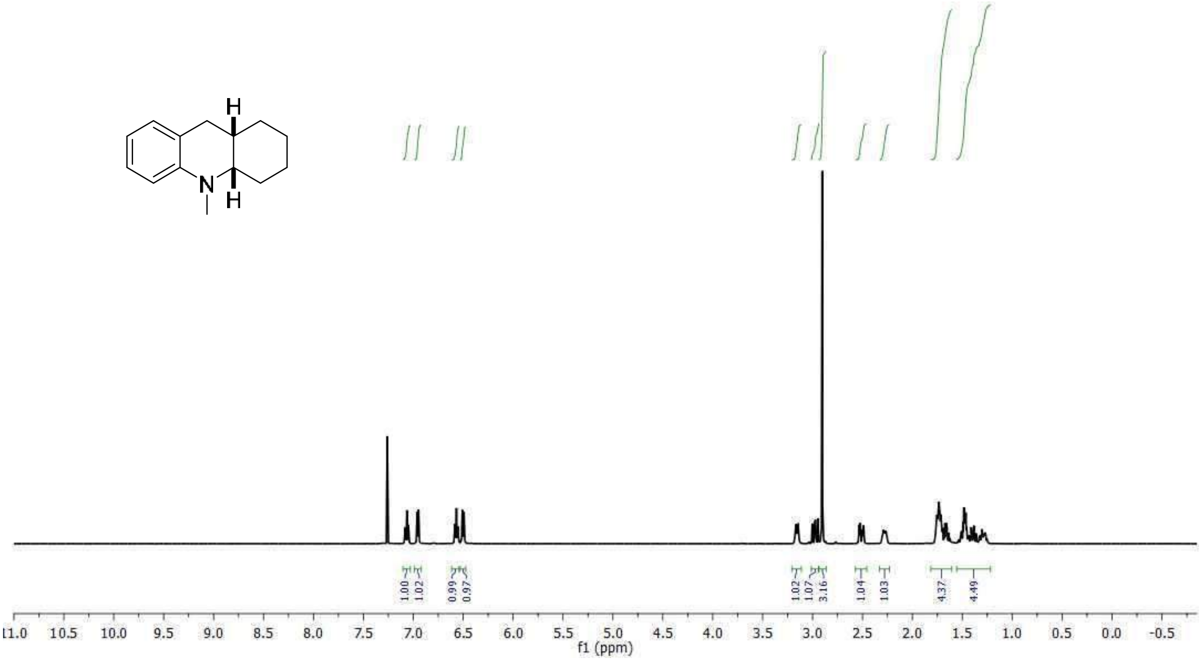

^{13}C NMR, 125 MHz, $CDCl_3$ **7**

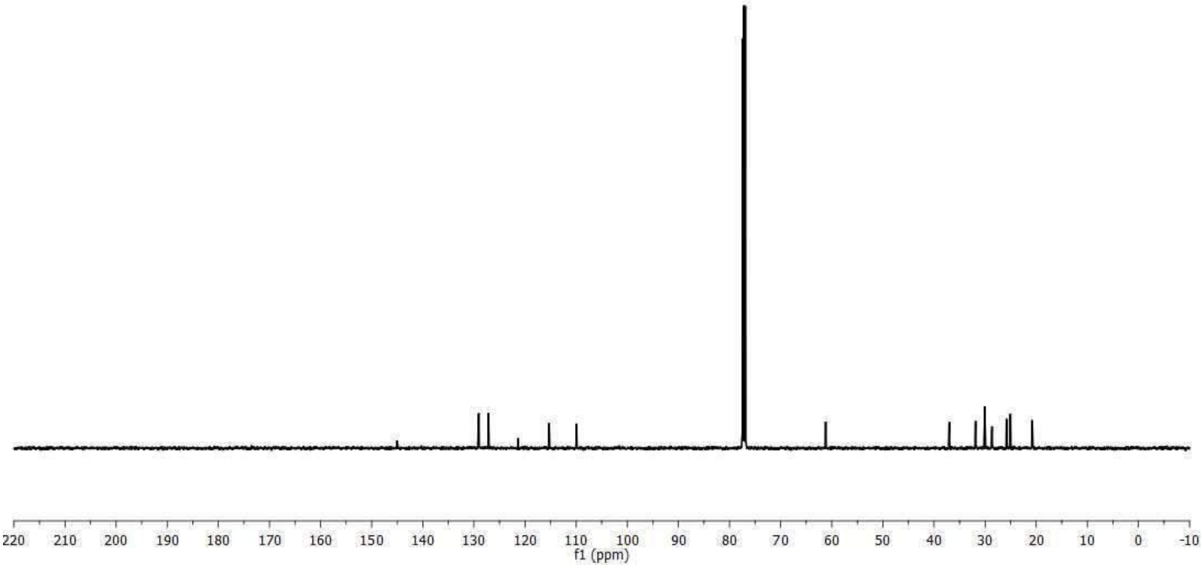

^{1}H NMR, 400 MHz, $CDCl_3$ **9**

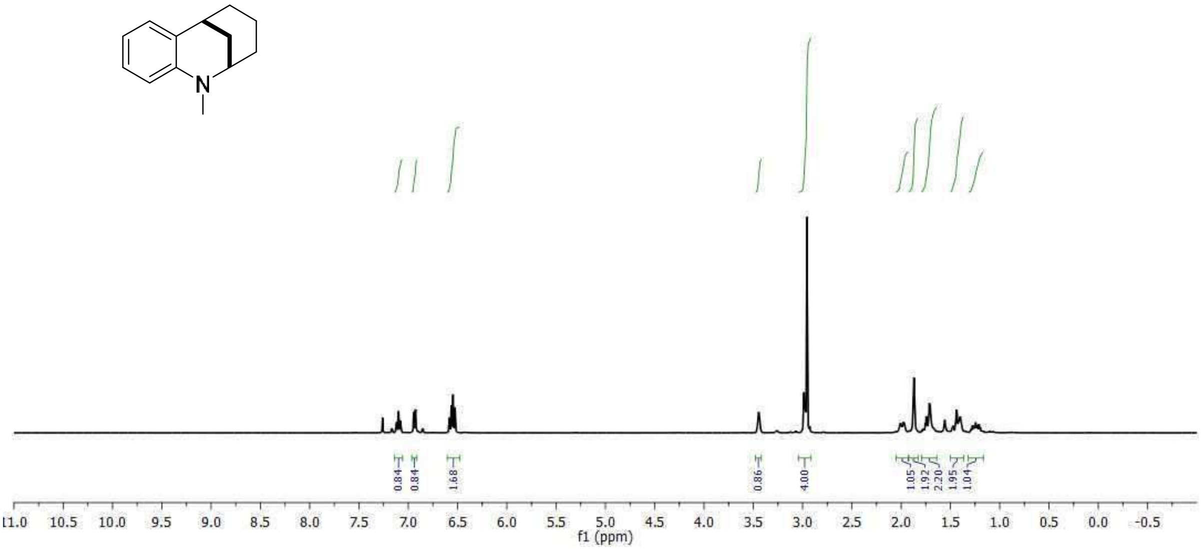

^{13}C NMR, 100 MHz, $CDCl_3$ **9**

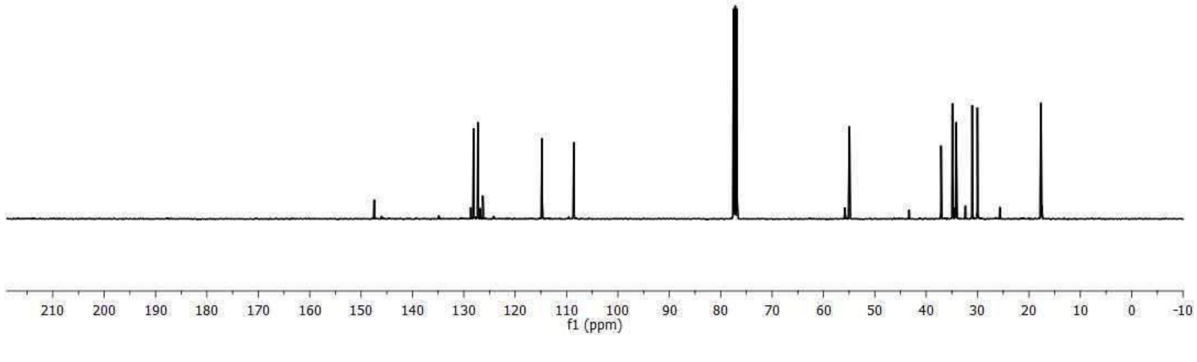

^{1}H NMR, 500 MHz, $CDCl_3$ **12a**

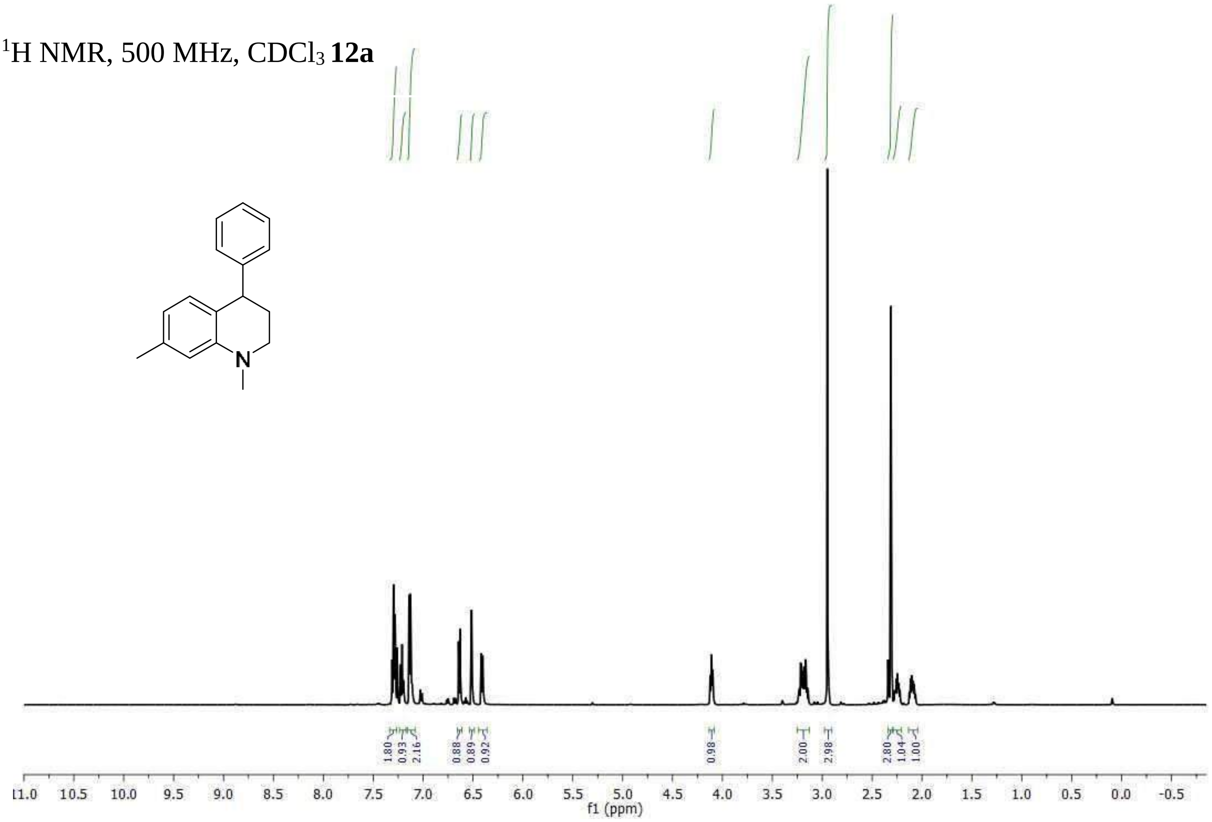

^{13}C NMR, 125 MHz, $CDCl_3$ **12a**

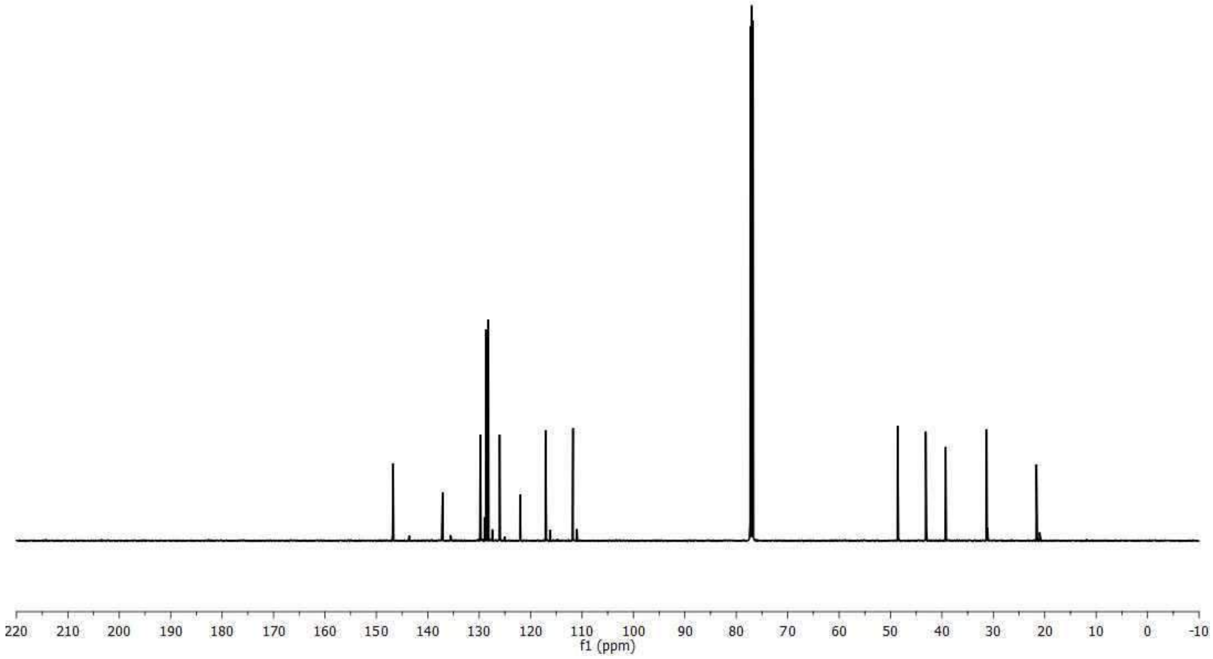

^{1}H NMR, 500 MHz, $CDCl_3$ **13a**

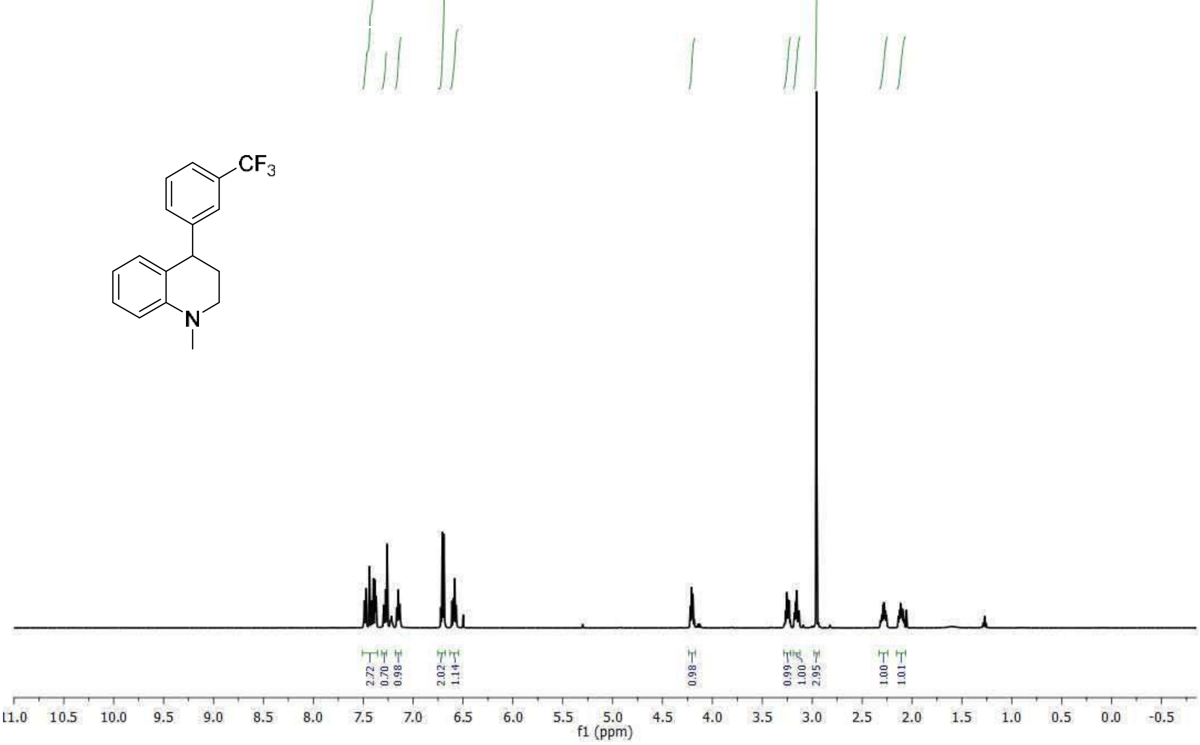

^{13}C NMR, 100 MHz, $CDCl_3$ **13a**

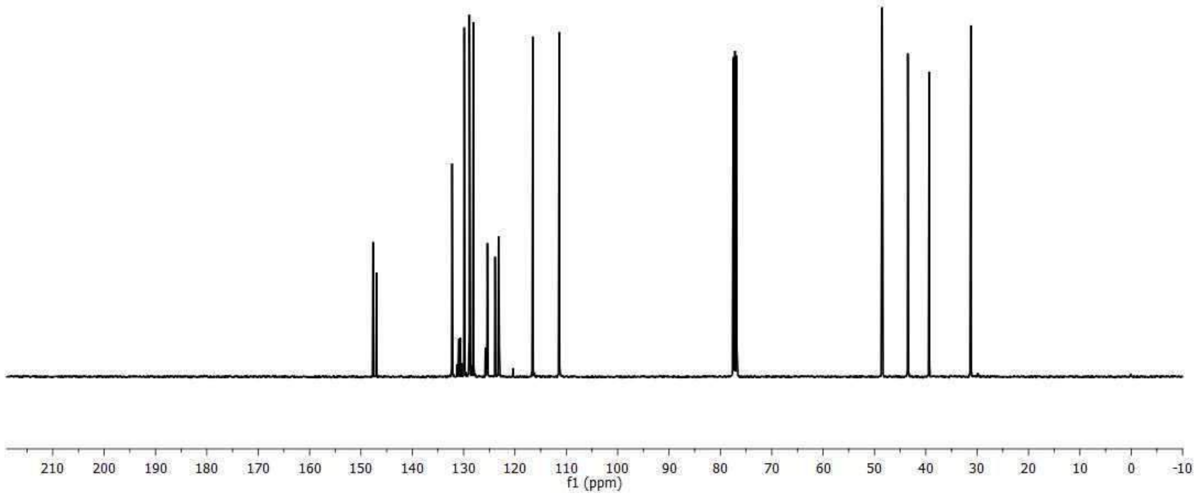

^{1}H NMR, 400 MHz, $CDCl_3$ **15**

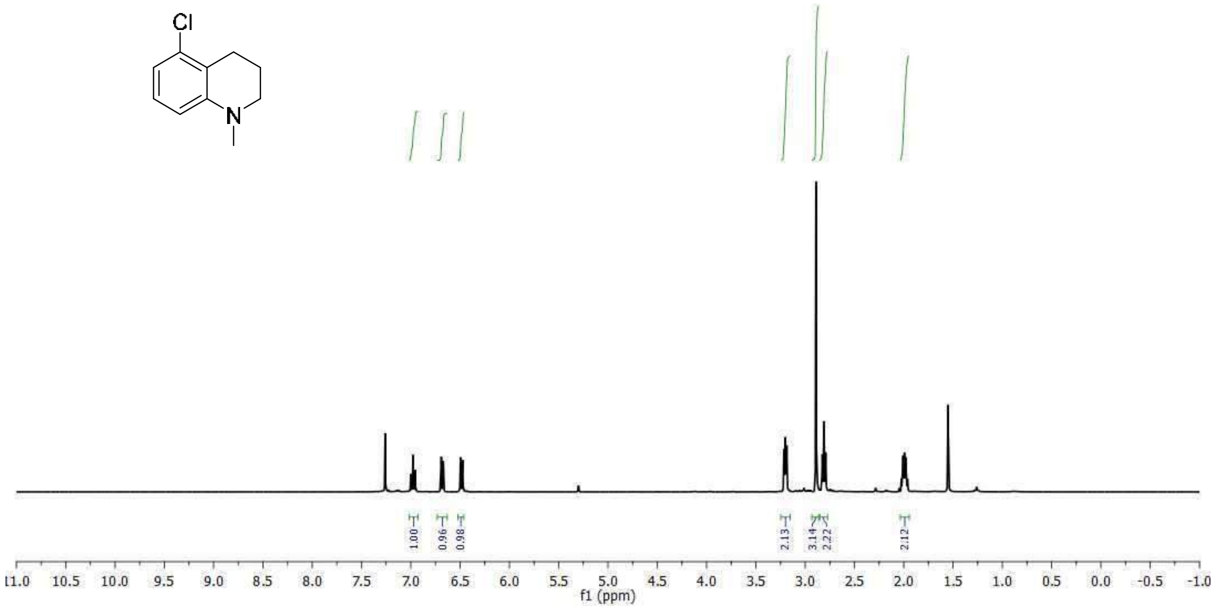

^{13}C NMR, 100 MHz, $CDCl_3$ **15**

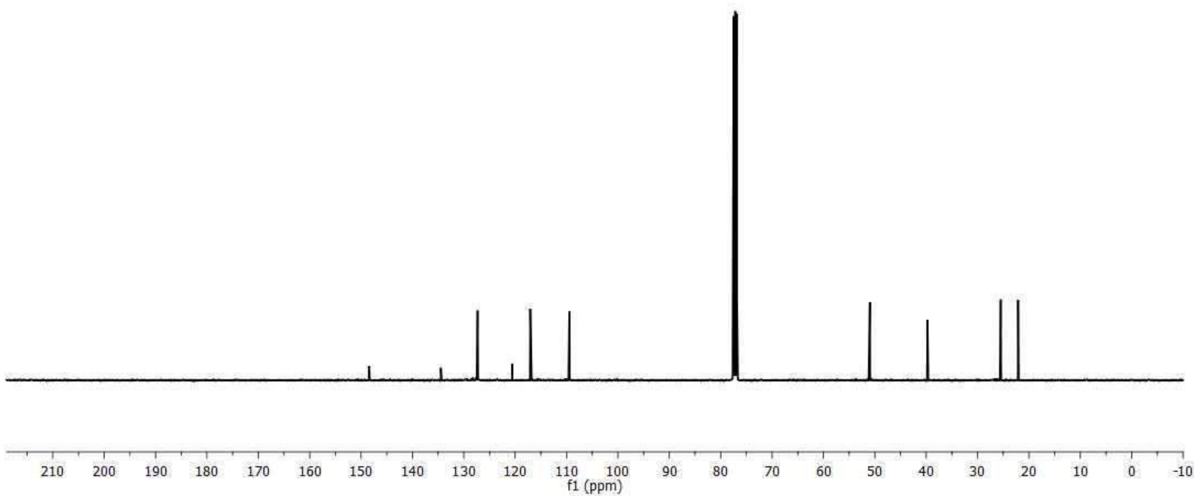

^{1}H NMR, 400 MHz, $CDCl_3$ **17**

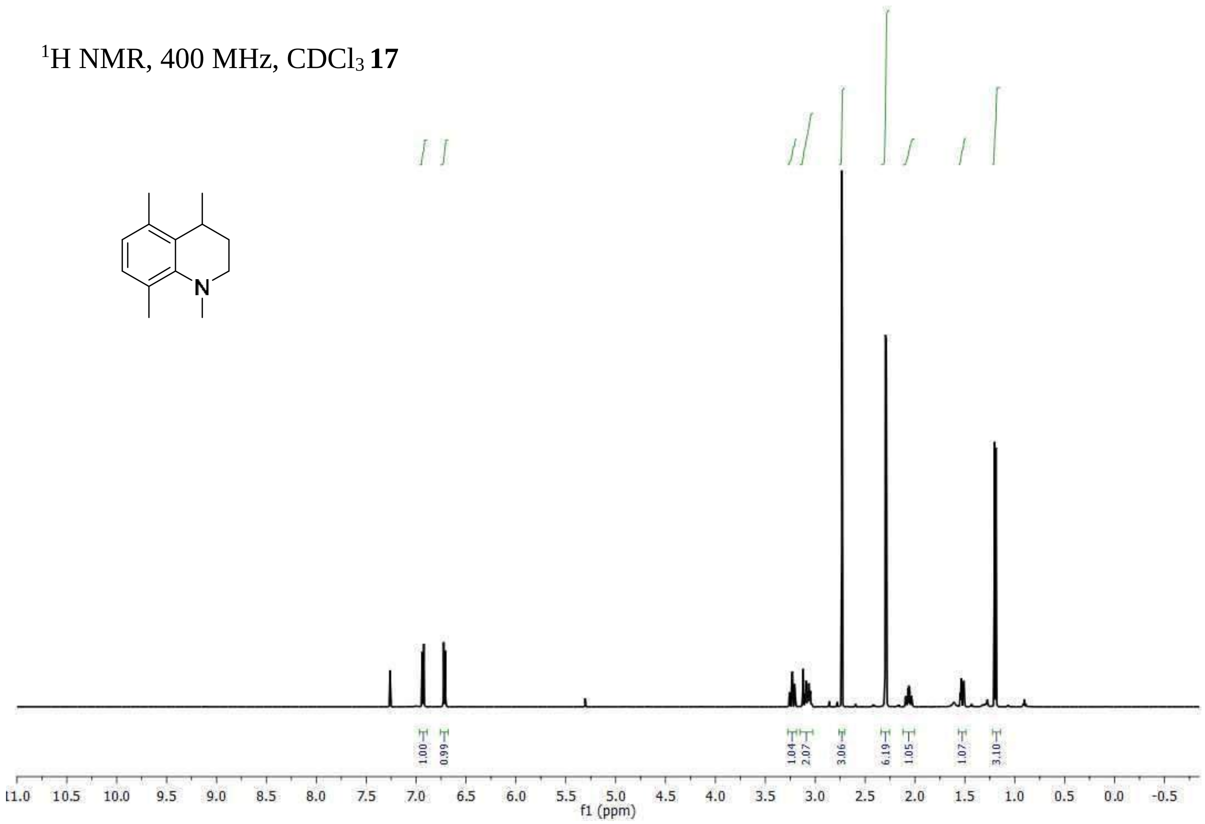

^{13}C NMR, 100 MHz, $CDCl_3$ **17**

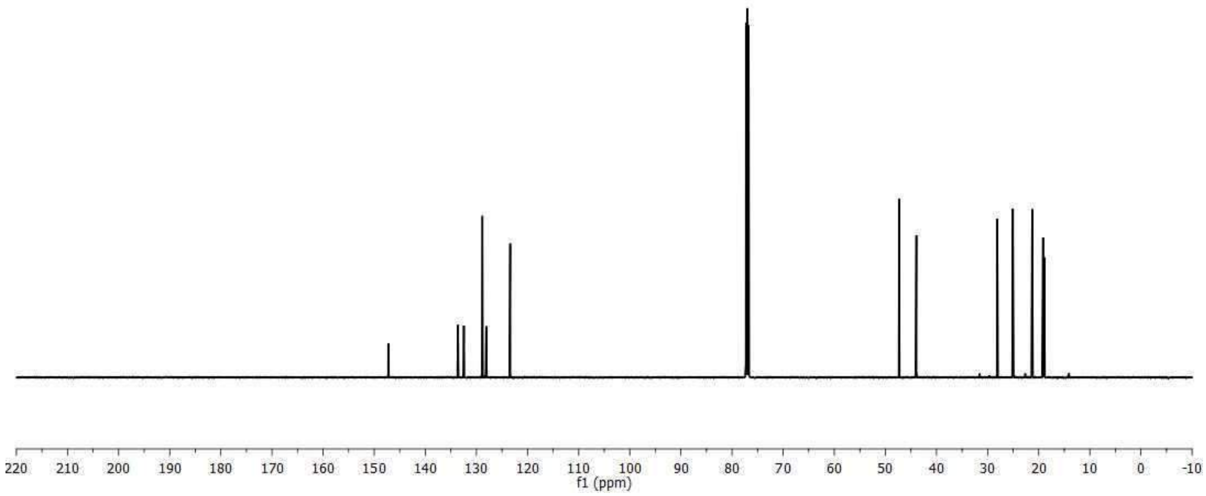

1.9 NMR/HPLC traces for asymmetric synthesis of angustureine 11

^{1}H NMR spectrum of Ellman addition product

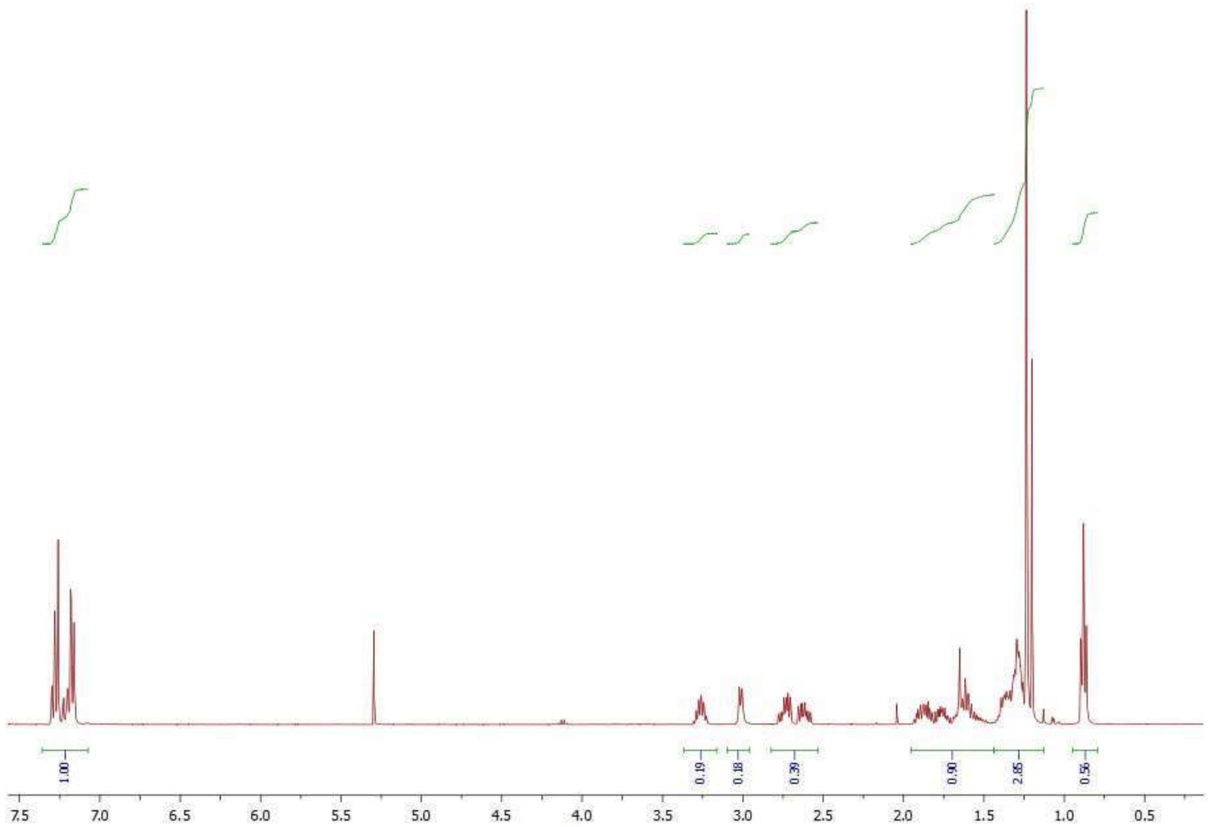

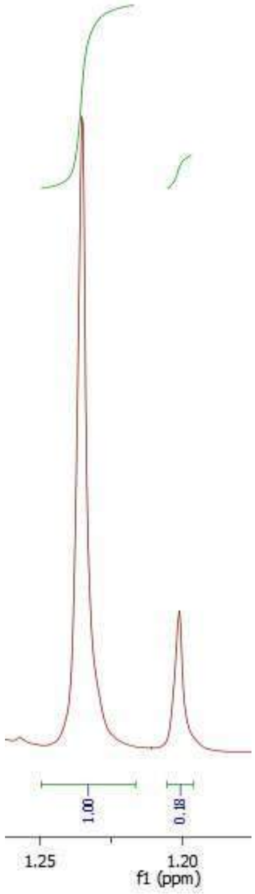

HPLC Traces angustureine

The enantiomeric excess was determined was determined by HPLC using a chiral phase column: CHIRALPAK® AD-H column; flowrate 0.5 mL min^{-1}; UV 254 nm; eluent hexane 100%; *R*t (1) = 15.88 min (*R*)-enantiomer, (2) = 18.16 min (*S*)-enantiomer.

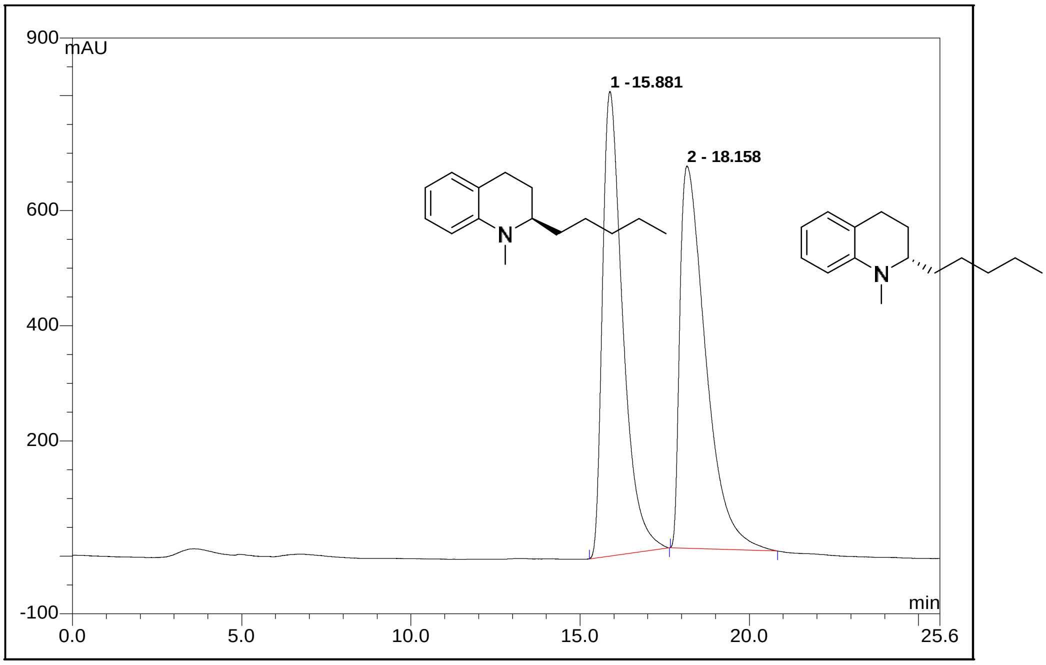

No.	Ret.Time min	Height mAU	Area mAU*min	Rel.Area %
1	16.19	833.560	595.843	83.92
2	18.78	150.482	114.180	16.08
Total:		984.042	710.023	100.00

1.10 DFT Calculations

Description of theoretical method

Electronic structure calculations using the Gaussian 09 suite of programs [*Frisch et al.*, *Gaussian 09, Revision A.1*, Gaussian, Inc., Wallingford CT] were used to map the stationary points on the electronic potential energy surface (PES) for the cyclization of the aminyl radical, and then for the cyclization of the protonated aminium radical. Molecular geometries were first optimised using hybrid density functional theory, which includes some exact Hartree-Fock exchange. The B3LYP method was used together with the 6-311+G(2d,p) triple zeta basis set. This large, flexible basis set has both polarization and diffuse functions added to the atoms. The resulting rotational constants and vibrational frequencies were then used to compute the enthalpy (*H*) and Gibbs free energy (*G*) at 298 K. The calculations were performed in the presence of the solvent, dichloromethane, using the Polarizable Continuum Model which places the solute in a cavity within the solvent reaction field [Tomasi *et al.*, Chem. Rev., 2005, 105, 2999-3093; Frisch *et al. J. Chem. Phys.*, **2010**, *132*, 114110].

Table S1. Molecular geometries for the neutral aminyl radical
(element followed by Cartesian co-ordinates in Ångstroms)

**Initial structure $C_6H_5(CH_2)_3$-N-CH_3**	Colour code: grey = C; white = H; blue = N
C,0,2.489152686,-1.2205951357,0.8198530258<br>C,0,1.450289745,-0.3663533042,1.1834879555<br>C,0,1.0303609562,0.6607565162,0.3352990069<br>C, 1.6868106012,0.8123028642,-0.8908732585<br>C, 2.7243983666,-0.0372516,-1.2594471985<br>C, 3.1294420981,-1.0599416858,-0.4044974158<br>H, 2.798318414,-2.0098484205,1.4954562134<br>H, 0.9612546153,-0.4980900332,2.142867192<br>H, 1.3861936513,1.6089219553,-1.5634549233<br>H, 3.2210622901,0.1013839815,-2.2128808573<br>H, 3.9389453447,-1.7215404374,-0.6892795763<br>C, -0.1033304184,1.5866213551,0.7207271062<br>C, -1.4063741555,1.3611323062,-0.06872355<br>H, 0.2132964764,2.6222146966,0.5655930437<br>H, -0.306565557,1.4821892703,1.7907151212<br>C, -2.0610298393,0.0088919907,0.1994793744<br>H, -2.1164889432,2.1519295714,0.1898673485<br>H, -1.2103344929,1.4544950491,-1.1408034092<br>H, -1.365502325,-0.8102732735,-0.0466354202<br>H, -2.2713239136,-0.1062147267,1.27659146<br>N, -3.2745108519,-0.1681028978,-0.5563096359<br>C, -3.9464649354,-1.4014803606,-0.2434239393<br>H, -4.8426090244,-1.5159428901,-0.8538031217<br>H, -4.2361221051,-1.4332062198,0.817807738<br>H, -3.2891226834,-2.2677765716,-0.4115122795	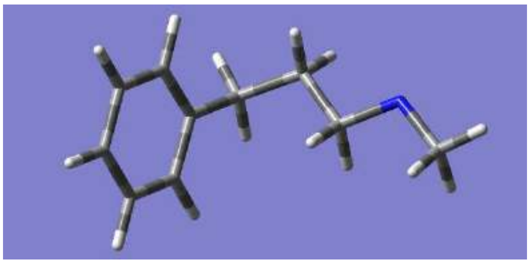

$C_6H_5(CH_2)_3$-N-CH_3 after *ortho*-substitution

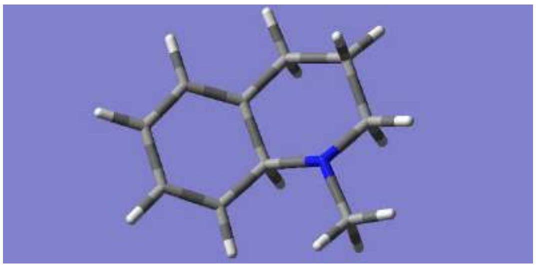

C, -1.266285407,1.3874729265,0.2933128401
C-0.0898884053,0.4714639753,0.4834995996
C, -0.4319703798,-0.9809937804,0.2330475517
C,-1.6803230247,-1.3599200681,-0.1660283386
C,-2.7293622834,-0.4316113696,-0.3433189956
C,-2.4917142308,0.9389253351,-0.0924738268
H,-1.1297220751,2.4376744388,0.5157306064
H,0.2308214418,0.5615352123,1.547860823
H,-1.8733284842,-2.4125337563,-0.3501572069
H,-3.7080329117,-0.768324155,-0.6602008221
H,-3.3077890194,1.645595619,-0.1992153247
C,0.6960396014,-1.9474098744,0.4167689101
C,1.9213308725,-1.4822113724,-0.3841759793
H,0.3926139264,-2.9559358366,0.127654374
H,0.9720765316,-1.985226145,1.4801620762
C,2.2341261227,-0.0230927831,-0.0772407312
H,2.7909987443,-2.0994767275,-0.1432195182
H,1.7243243321,-1.5924374766,-1.4543725949
H,2.5523698721,0.0699865589,0.9791085708
H,3.0708774765,0.3163112788,-0.6920080774
N,1.0933164504,0.8587551271,-0.3541181693
C,1.4862082744,2.2507437104,-0.1474601036
H,0.7101758494,2.9304938571,-0.4956628815
H,2.3881599685,2.4522128214,-0.7271516191
H,1.7029667573,2.4798804844,0.9108598375

$C_6H_5(CH_2)_3$-N-CH_3 after*spiro*-substitution

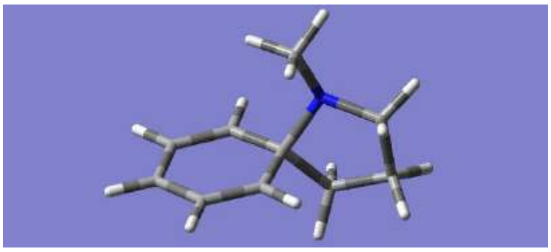

C,-1.897696519,0.3701463743,1.3547666867
C,-0.5518982861,0.2282168881,1.1954952381
C,0.0769139202,-0.3279535679,-0.0591873289
C,-0.959685983,-0.6684207877,-1.0900268066
C,-2.2973302435,-0.5214735893,-0.8833063945
C,-2.801242396,-0.0051118534,0.3340970505
H,-2.2875783389,0.7792501812,2.2811058969
H,0.1153722209,0.5231555227,1.99803754
H,-0.5843507152,-1.0485263997,-2.0340691421
H,-2.993120446,-0.7970529022,-1.6686783046
H,-3.8677320934,0.1127355656,0.4773050873
C,0.9732869781,-1.5607537462,0.2761777472
C,2.3331339679,-0.9686471813,0.702882013
H,1.0826539034,-2.1579397918,-0.6310163263
H,0.5174959707,-2.1912956532,1.039412886
C,2.316803048,0.4815305167,0.1786632763
H,2.4534866592,-0.984834118,1.7867476983
H,3.1575201717,-1.5430917351,0.2795892975
H,2.2910148334,1.1976609365,1.0155459966
H,3.196164612,0.7284734639,-0.4218042306
N,1.113308249,0.5758537863,-0.6534418861

C,0.6980468662,1.9400229852,-0.9386330683<br>H,-0.2035135054,1.9341622533,-1.5532785189<br>H,1.487247211,2.4442669191,-1.5014219049<br>H,0.4958189147,2.5339039328,-0.0329985027	

Table S2. Molecular geometries for the protonated aminium radical
(element followed by Cartesian co-ordinates in Ångstroms)

**$C_6H_5(CH_2)_3$-NH^+-CH_3**<br><br>C,2.7024629869,-1.1812426064,0.6921792741<br>C,1.5954956801,-0.4734917197,1.1523766212<br>C,1.0702406918,0.5941100563,0.4168276319<br>C,1.6889278001,0.9403922285,-0.7913168743<br>C,2.7932022387,0.2361928588,-1.2527840024<br>C,3.3029759121,-0.8298627955,-0.5123765759<br>H,3.0971718275,-2.0032507107,1.2772238573<br>H,1.1396396862,-0.7465049684,2.0978994029<br>H,1.307933268,1.7757566706,-1.3690816168<br>H,3.2631852758,0.5212929163,-2.1864272329<br>H,4.166777615,-1.3768671034,-0.870266933<br>C,-0.1360959111,1.3562539354,0.9017020978<br>C,-1.4124776663,1.1219520537,0.069110281<br>H,0.0631432615,2.4314686231,0.8530873693<br>H,-0.3359160891,1.1191102137,1.9487455266<br>C,-1.8730438226,-0.3631207415,0.1935429386<br>H,-2.2144374958,1.7719207185,0.4213816089<br>H,-1.2281898783,1.3514647243,-0.9817375303<br>H,-1.0515505352,-1.0013914952,-0.1488444289<br>H,-2.1283680936,-0.5978714034,1.2255663683<br>N,-3.0067369226,-0.6053944552,-0.6348269296<br>C,-4.356006026,-0.7438438216,-0.1707630637<br>H,-5.0405264058,-0.2813552074,-0.8823476872<br>H,-4.4577282279,-0.3141469674,0.8229690144<br>H,-4.6013131314,-1.8153159206,-0.1217117553<br>H,-2.8488570382,-0.6722120828,-1.6381853619	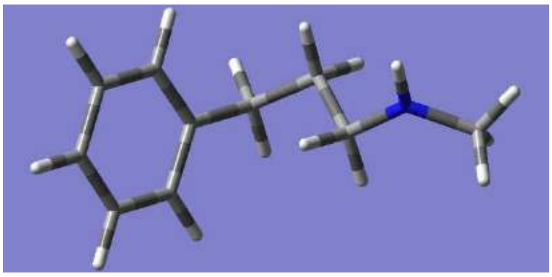
**$C_6H_5(CH_2)_3$-NH^+-CH_3 after *ortho*-substitution**<br><br>C,-1.299853668,1.3711336927,0.3868265167<br>C,-0.1502988958,0.4398329851,0.5719967598<br>C,-0.4526461778,-1.0009226009,0.2738338036<br>C,-1.6777748116,-1.3620482518,-0.2082554817<br>C,-2.7110139569,-0.424619645,-0.4097460793<br>C,-2.4982767027,0.933874359,-0.0871155386<br>H,-1.1791212865,2.4015069755,0.6926527332<br>H,0.2746973002,0.547335841,1.5785025065<br>H,-1.8622949686,-2.4066691031,-0.4331577399<br>H,-3.6691299735,-0.7484603185,-0.7932628683<br>H,-3.3140657744,1.6386741197,-0.1948609846<br>C,0.6806017643,-1.959418109,0.472774426<br>C,1.898345289,-1.5153766877,-0.3550653981<br>H,0.3806402161,-2.9694255435,0.1928497028<br>H,0.9609742789,-1.9848390911,1.5331469384<br>C,2.2590499768,-0.0626621573,-0.0908074354<br>H,2.7711887224,-2.1241839787,-0.1121641105<br>H,1.6963803795,-1.657903022,-1.420368871	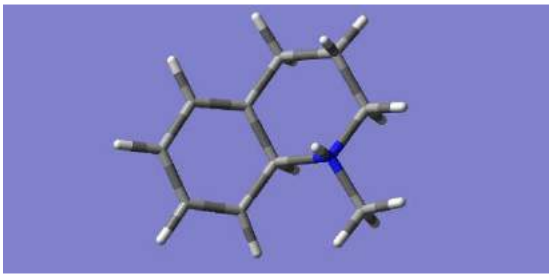

H,2.5658940895,0.0973235452,0.9436993803<br>H,3.0619000545,0.2768261156,-0.7435666002<br>N,1.0868003786,0.8628866855,-0.3257029171<br>C,1.4889955327,2.2903859308,-0.1331151762<br>H,0.6810655485,2.940424689,-0.4526718434<br>H,2.373164034,2.4813137424,-0.7361163537<br>H,1.7137441276,2.4490261942,0.9190383219<br>H,0.7828645233,0.7504666326,-1.2960806913	
**$C_6H_5(CH_2)_3$-NH^+-CH_3 *spiro*-substitution**<br><br>C,-1.3991437771,2.653843031,0.2957827336<br>C,-0.5176762489,1.6950058233,0.6948202443<br>C,-0.1577460213,0.5380426881,-0.1692074691<br>C,-0.9007212992,0.5158400667,-1.4641482607<br>C,-1.7654503751,1.5034019063,-1.8241682268<br>C,-2.0248807734,2.596145667,-0.9678663296<br>H,-1.6394571165,3.4664187305,0.9708592867<br>H,-0.0788273099,1.735797047,1.6843575229<br>H,-0.7431731575,-0.3383136676,-2.114601599<br>H,-2.2814142396,1.4376251341,-2.7744178531<br>H,-2.7166601376,3.3711810349,-1.2690012314<br>C,-0.1929211882,-0.818332627,0.5962378928<br>C,1.0989794493,-1.5742983323,0.2672555774<br>H,-1.0873509652,-1.3757624173,0.3235120721<br>H,-0.2447489058,-0.6172001127,1.6670878175<br>C,2.1413983101,-0.4734402718,0.1865673701<br>H,1.3511552201,-2.3106364879,1.0289290392<br>H,1.0196885606,-2.0982936265,-0.6876799276<br>H,2.3911905719,-0.0750483208,1.1696812412<br>H,3.058077189,-0.7434705571,-0.3348990771<br>N,1.4474980215,0.6154080445,-0.5791432357<br>C,2.0908552443,1.9548532155,-0.4937403049<br>H,1.5289626417,2.6586739056,-1.1024406792<br>H,3.1081222978,1.8686868986,-0.8710899154<br>H,2.1064209497,2.2837808461,0.5407271885<br>H,1.4574903094,0.3455212719,-1.5636667567	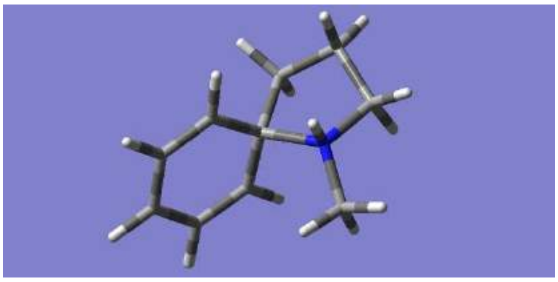
**TS1 between $C_6H_5(CH_2)_3$-NH^+-CH_3 and intermediate I1**<br><br>C,2.2324255605,-1.3886997732,0.7118225015<br>C,1.3664792808,-0.4260182696,1.2277046935<br>C,0.9375860909,0.6475982173,0.4450835461<br>C,1.4034823517,0.7371621849,-0.8714691008<br>C,2.2682121723,-0.2208731043,-1.3901395447<br>C,2.6840065274,-1.2900436891,-0.5996283249<br>H,2.556610567,-2.2112789579,1.3382549525<br>H,1.0337298523,-0.5044240754,2.2571838465<br>H,1.0994704478,1.5724176481,-1.4936928108<br>H,2.6246027049,-0.1288609193,-2.4093691104	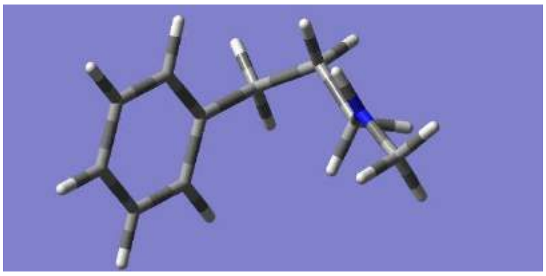

H,3.3600199263,-2.0348775898,-1.0018704069<br>C,-0.0079815131,1.6891361002,1.0019630789<br>C,-1.4046645939,1.6847764301,0.351781862<br>H,0.4154675934,2.6869005226,0.8603326774<br>H,-0.1141731447,1.5482656963,2.080415894<br>C,-2.1879324625,0.3863269685,0.5738735568<br>H,-1.9875782103,2.5133630828,0.755996214<br>H,-1.3096328714,1.8793558044,-0.7200808034<br>H,-1.6345817987,-0.3395991175,1.193612317<br>H,-3.1232182477,0.5456423882,1.1289305164<br>N,-2.5414410843,-0.3082655392,-0.6282735826<br>C,-3.3453835333,-1.4930787644,-0.668397171<br>H,-4.1986283347,-1.3173588588,-1.3330283679<br>H,-3.6801813046,-1.7601789276,0.329085939<br>H,-2.756341812,-2.3041382737,-1.1125556569<br>H,-2.190445164,0.0507938165,-1.515594715	
**Intermediate I1**<br><br>C,1.8700311514,-1.6132825118,0.1203355513<br>C,1.2226406486,-0.4623478998,0.541817735<br>C,0.6477591707,0.4212432388,-0.3850783958<br>C,0.7690878398,0.1191113075,-1.7580205123<br>C,1.4161589272,-1.0334656909,-2.1798384869<br>C,1.9650531944,-1.906113641,-1.2425157851<br>H,2.305832646,-2.2856983978,0.8489396455<br>H,1.1571009575,-0.2372699511,1.5999261902<br>H,0.3658474362,0.8050853441,-2.4944717475<br>H,1.5016207084,-1.2483741564,-3.2377525427<br>H,2.4735081152,-2.8047309032,-1.5692757407<br>C,-0.0374947516,1.6810389261,0.0712148556<br>C,-1.5243526794,1.7543085857,-0.3227997578<br>H,0.4645589025,2.5501361348,-0.3662200653<br>H,0.0567287396,1.7749687008,1.1548002927<br>C,-2.308723552,0.5321252458,0.1650097996<br>H,-1.9801969608,2.6453754155,0.111556166<br>H,-1.6340160747,1.8449427549,-1.406398471<br>H,-2.2158905737,0.386083178,1.2410621987<br>H,-3.3745476946,0.6830050942,-0.0654228346<br>N,-1.8885410526,-0.677274412,-0.4942299649<br>C,-1.8708449269,-1.9759883389,0.1231396229<br>H,-2.8243665837,-2.4847681407,-0.0720021186<br>H,-1.7255067737,-1.8788773695,1.1956641355<br>H,-1.0751223391,-2.5776486844,-0.3202303377<br>H,-1.8898969748,-0.6647133987,-1.510267922	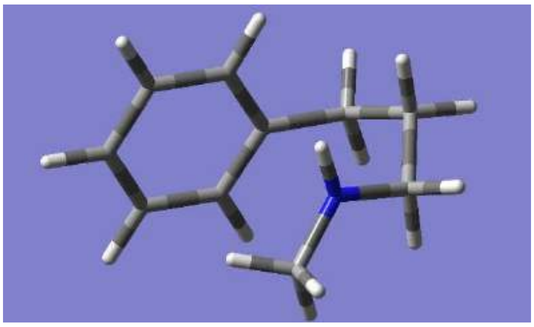
**TS2 from I1 to *ortho*- $C_6H_5(CH_2)_3$-NH^+-CH_3**<br><br>C,-1.7591715548,0.8136341193,0.4103828445<br>C,-0.5428230067,0.0981344285,0.6929041392<br>C,-0.2912308398,-1.1495381685,-0.0053059624<br>C,-1.1631968954,-1.5482234063,-0.9947692988	

C,-2.3060485791,-0.7960040023,-1.2973040837<br>C,-2.5981881003,0.3817120879,-0.5833946514<br>H,-2.0064087655,1.6849310901,1.0025366633<br>H,-0.1691526194,0.1694241854,1.7104377486<br>H,-0.9644450325,-2.4609246661,-1.5440160045<br>H,-2.9777958962,-1.1311583326,-2.0771867946<br>H,-3.5081546386,0.9269630964,-0.8000924406<br>C,1.0016529138,-1.8485804361,0.2831470994<br>C,2.1900095384,-0.9954673608,-0.2126764635<br>H,1.0259377412,-2.823259383,-0.2050468185<br>H,1.1057399685,-2.0157010543,1.3599787107<br>C,2.1958916514,0.414411274,0.3634934406<br>H,3.1284019158,-1.4760580451,0.0698618126<br>H,2.1757477881,-0.9479616652,-1.3054411564<br>H,2.2864923013,0.3983579651,1.4515288695<br>H,3.0427479213,0.9850807219,-0.0290335119<br>N,0.958414749,1.1649761562,0.0439487463<br>C,0.9107588952,2.5263395506,0.6010673641<br>H,-0.0047418679,3.0181808804,0.2823854053<br>H,1.7697404257,3.0972802962,0.2438859888<br>H,0.9435847675,2.4718078543,1.688035302<br>H,0.7914452188,1.184133814,-0.9609479487	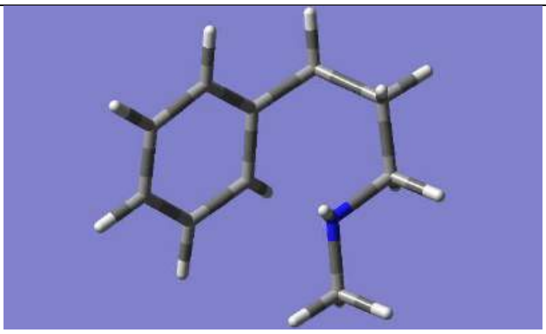
**TS3**<br>C,-2.1138199963,0.4547621408,0.9992078754<br>C,-0.8123660665,0.0833540769,1.1894127317<br>C,-0.0334865368,-0.5621003766,0.1314305673<br>C,-0.8011562101,-0.9133048286,-1.0723646754<br>C,-2.0988629108,-0.5205368717,-1.235300756<br>C,-2.7710393677,0.1852379664,-0.2170955368<br>H,-2.6553485703,0.9397124246,1.8021108108<br>H,-0.3274348465,0.2584681826,2.1419381198<br>H,-0.3063464655,-1.4970817065,-1.840148105<br>H,-2.628369888,-0.781328057,-2.1432960111<br>H,-3.8001116238,0.4890716375,-0.3565087<br>C,1.0068498176,-1.5918146496,0.593150336<br>C,2.3142455979,-1.3694836552,-0.1738770028<br>H,0.6107375067,-2.5966422555,0.4437892014<br>H,1.180321279,-1.4646877629,1.6632329443<br>C,2.4976608783,0.1394222825,-0.1709209249<br>H,3.1544351163,-1.873940001,0.3019708642<br>H,2.2435072292,-1.7452692897,-1.1975674474<br>H,2.7637121134,0.5056710111,0.8207464908<br>H,3.2346089666,0.4992575895,-0.8897944039<br>N,1.1625218837,0.6967559257,-0.5099432985<br>C,0.923367897,2.0945128133,-0.0980525503<br>H,-0.0817944123,2.3891598291,-0.3892750316<br>H,1.649940435,2.7397412073,-0.5925834723<br>H,1.0364853805,2.1790694344,0.9796559212<br>H,1.0137751834,0.6236470028,-1.5152624168	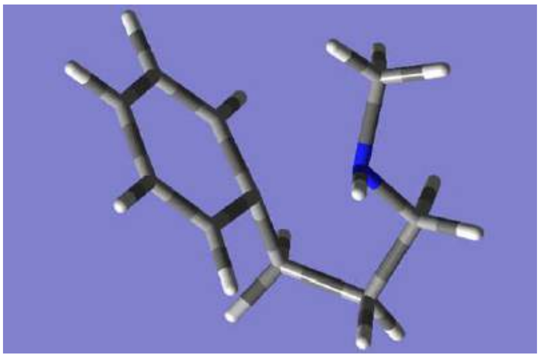

Table S3. Enthalpy and Gibbs free energy changes along the potential energy surfaces

	ΔH^o(298 K) / kJ mol^{-1}	ΔG^o(298 K) / kJ mol^{-1}
$C_6H_5(CH_2)_3$-N-$CH_3 \rightarrow$ *ortho*	36.5	54.2
$C_6H_5(CH_2)_3$-N-$CH_3 \rightarrow$ *spiro*	53.5	67.3
$C_6H_5(CH_2)_3$-NH^+-$CH_3 \rightarrow$ *ortho*	6.9	23.1
$C_6H_5(CH_2)_3$-NH^+-$CH_3 \rightarrow$ *spiro*	29.1	43.5

1.11 Bibliography

1 R. Cadoni, A. Porcheddu, G. Giacomelli and L. De Luca, *Org. Lett.*, 2012, **14**, 5014–5017.

2 A. E. Strom and J. F. Hartwig, *J. Org. Chem.*, 2013, **78**, 8909–8914.

3 S. Li, K. Huang, J. Zhang, W. Wu and X. Zhang, *Org. Lett.*, 2013, **15**, 3078–3081.

4 R. Itooka, Y. Iguchi and N. Miyaura, *J. Org. Chem.*, 2003, **68**, 6000–6004.

5 T. O. Vieira, M. J. Green and H. Alper, *Org. Lett.*, 2006, **8**, 6143–6145.

6 A. R. Maguire, N. R. Buckley, P. O'Leary and G. Ferguson, *J. Chem. Soc. Perkin Trans. 1*, 1998, 4077–4092.

7 P. Prediger, A. R. da Silva and C. R. D. Correia, *Tetrahedron*, 2014, **70**, 3333–3341.

8 D. G. Pintori and M. F. Greaney, *Org. Lett.*, 2011, **13**, 5713–5715.

9 Y. Li, Y.-Y. Hu and S.-L. Zhang, *Chem. Commun.*, 2013, **49**, 10635–10637.

10 H. Takakura, R. Kojima, M. Kamiya, E. Kobayashi, T. Komatsu, T. Ueno, T. Terai, K. Hanaoka, T. Nagano and Y. Urano, *J. Am. Chem. Soc.*, 2015, **137**, 4010–4013.

11 B. R. Henke, T. G. Consler, N. Go, R. L. Hale, D. R. Hohman, S. a. Jones, A. T. Lu, L. B. Moore, J. T. Moore, L. a. Orband-Miller, R. G. Robinett, J. Shearin, P. K. Spearing, E. L. Stewart, P. S. Turnbull, S. L. Weaver, S. P. Williams, G. B. Wisely and M. H. Lambert, *J. Med. Chem.*, 2002, **45**, 5492–5505.

12 M. A. Bigi and M. C. White, *J. Am. Chem. Soc.*, 2013, **135**, 7831–7834.

13 A. Millet and O. Baudoin, *Org. Lett.*, 2014, **16**, 3998–4000.

14 M. Vellakkaran, M. M. S. Andappan and N. Kommu, *Green Chem.*, 2014, **16**, 2788.

15 R. Shang, Z. Huang, X. Xiao, X. Lu, Y. Fu and L. Liu, *Adv. Synth. Catal.*, 2012, 2465–2472.

16 J. R. DeBergh, K. M. Spivey and J. M. Ready, *J. Am. Chem. Soc.*, 2008, **130**, 7828–7829.

17 Q. Lei, Y. Wei, D. Talwar, C. Wang, D. Xue and J. Xiao, *Chem. Eur. J.*, 2013, **19**, 4021–4029.

18 Z. Huang and G. Dong, *J. Am. Chem. Soc.*, 2013, **135**, 17747–50.

19 X. Wang, T. Pei, X. Han and R. A. Widenhoefer, *Org. Lett.*, 2003, **5**, 2699–2701.

20 X. Jiang, C. Wang, Y. Wei, D. Xue, Z. Liu and J. Xiao, *Chem. Eur. J.*, 2014, **20**, 58–63.

21 Y. Zhao, S. W. Foo and S. Saito, *Angew. Chem. Int. Ed.*, 2011, **50**, 3006–3009.

22 A. O'Byrne and P. Evans, *Tetrahedron*, 2008, **64**, 8067–8072.

23 N. Kurono, E. Honda, F. Komatsu, K. Orito and M. Tokuda, *Tetrahedron*, 2004, **60**, 1791–1801.

24 R. Omar-Amrani, A. Thomas, E. Brenner, R. Schneider and Y. Fort, *Org. Lett.*, 2003, **5**, 2311–4.

25 D. W. Wang, X. B. Wang, D. S. Wang, S. M. Lu, Y. G. Zhou and Y. X. Li, *J. Org. Chem.*, 2009, **74**, 2780–2787.
